# CRISPR/Cas9-mediated genome editing efficiently creates specific mutations at multiple loci using one sgRNA in *Brassica napus*

**DOI:** 10.1038/s41598-017-07871-9

**Published:** 2017-08-08

**Authors:** Hong Yang, Jia-Jing Wu, Ting Tang, Ke-De Liu, Cheng Dai

**Affiliations:** 1 College of Plant Science & Technology, Huazhong Agricultural University, Wuhan, 430070 China; 2 National Key Laboratory of Crop Genetic Improvement, Huazhong Agricultural University, Wuhan, 430070 China

## Abstract

CRISPR/Cas9 is a valuable tool for both basic and applied research that has been widely applied to different plant species. Nonetheless, a systematical assessment of the efficiency of this method is not available for the allotetraploid *Brassica napus*—an important oilseed crop. In this study, we examined the mutation efficiency of the CRISPR/Cas9 method for 12 genes and also determined the pattern, specificity and heritability of these gene modifications in *B*. *napus*. The average mutation frequency for a single-gene targeted sgRNA in the T0 generation is 65.3%. For paralogous genes located in conserved regions that were targeted by sgRNAs, we observed mutation frequencies that ranged from 27.6% to 96.6%. Homozygotes were readily found in T0 plants. A total of 48.2% of the gene mutations, including homozygotes, bi-alleles, and heterozygotes were stably inherited as classic Mendelian alleles in the next generation (T1) without any new mutations or reversions. Moreover, no mutation was found in the putative off-target sites among the examined T0 plants. Collectively, our results demonstrate that CRISPR/Cas9 is an efficient tool for creating targeted genome modifications at multiple loci that are stable and inheritable in *B*. *napus*. These findings open many doors for biotechnological applications in oilseed crops.

## Introduction

*Brassica* species, particularly canola varieties, are cultivated worldwide for edible oil, animal feed, and biodiesel because of their high nutritional value and a high-energy output yield per hectare^1,2^. Development of new oilseed cultivars using traditional cross-breeding strategies is time-consuming and complicated because they are allotetraploids^3–5^. Therefore, new breeding technologies that can introduce one or a few traits into an elite background would appear useful for developing new cultivars of oilseed rape. In the last few decades, both mutagenesis and genetic transformation of particular DNA fragments have been extensively used to create new cultivars^6^. Compared to screening for natural mutations, these strategies greatly facilitate the breeding processes^6^. However, these methods have shortcomings. For instance, traditional mutagenesis strategies introduce random mutations that can only be removed using laborious and time-consuming selection strategies. Additionally, health and environmental concerns are associated with genetic transformation^7^. Therefore, seeking new ways to edit genes of interest without either random mutagenesis or transgenes becomes imperative.

Thus far, three genome-editing tools have been well developed: Zinc-Finger Nucleases (ZFNs), Transcription Activator-Like Effector Nucleases (TALENs) and Clustered Regularly Interspaced Palindromic Repeat (CRISPR)-associated protein 9 system (CRISPR/Cas9)^8^. Some of the principles for these tools are similar, such as generating site-specific double-strand breaks (DSBs) in the genome followed by error-prone DNA repair. There are two endogenous DNA repair pathways, namely homology-dependent repair (HDR) and non-homologous end-joining (NHEJ)^9^. NHEJ is the predominant mechanism in somatic plant cells^10,11^, which frequently causes short insertions or deletions (indels) around the DSBs^12^. ZFNs and TALENs were developed and applied in plants before CRISPR/Cas9^13–17^. One disadvantage of ZFNs and TALENs relative to CRISPR/Cas9 is that the plasmids required for ZFNs and TALENs are difficult to construct^18^. Partially because of this reason, the CRISPR/Cas9 system has been rapidly and widely applied for genome editing in both animals and plants^19–21^. Briefly, genome editing using CRISPR/Cas9 utilizes a 20-bp guide RNA sequence (sgRNA or gRNA) that uses base pairing to direct the Cas9 nuclease to the target site. Cas9 cuts the target site to generate a DSB. Mutations are introduced during the DNA repair process. Additionally, CRISPR/Cas9 is more precise and efficient than ZFNs and TALENs^18^. Moreover, the CRISPR/Cas9 system can edit multiple target sites by using multiple sgRNAs encoded in a single CRISPR array, thereby facilitating the rapid genetic analyses of complex regulatory circuits^21^. Due to these distinct advantages, the CRISPR/Cas9 technology has greatly accelerated both forward and reverse genetics in plants^22^. Besides gene deletions, CRISPR/Cas9 is useful for inserting specific DNA fragment into target sites and specifically altering the transcriptional activity of genes by fusing transcriptional activation or repression domains to an inactivated Cas9^23,24^.

The characteristics of CRISPR/Cas9-induced gene mutations have been carefully described in a small number of plant species. Mutation frequencies have ranged from 2.7% to 100% and are largely dependent on the promoter used to drive the expression of *Cas9*^9^. In *Arabidopsis* and *Camelina sativa*, mutations mostly occur in somatic cells, thus resulting in no homozygous or bi-allelic mutations in the T1 generation when *Cas9* is driven by either the *CaMV 35S* promoter or the *PcUbi4-2* promoter from *Petroselinum crispum*, respectively^25,26^. In contrast, the frequency goes up to 8.3% in the T1 generation when *Cas9* is driven by an egg-specific promoter^27^. However, for other plant species that require tissue culture for gene transformation, such as tomato and rice, the percentage of homozygous and bi-allelic mutants was much higher in the T0 generation when the *35S* promoter was used to drive Cas9^28–30^. These results indicate that both the transformation methods and the promoter activity influence the homozygous mutation frequency^9^. With regard to the genetic characteristics, mutations induced by CRISPR/Cas9 are stably inherited without generating novel mutations, and the segregation pattern in the descendants follow the classical Mendelian model in plants^27,30^.

*Brassica napus* (AACC), a young allotetraploid species, was derived from the hybridization of two diploid species *Brassica rapa* (AA) and *Brassica oleracea* <7500 years ago^31^. Hence, a majority of genes in *Brassica napus* (AACC) are multiple-copy genes that exhibit high sequence similarity, which hinders gene function studies. Although the CRISPR/Cas9 system was employed in *Brassica oleracea* and canola to generate mutants^32,33^, detailed observations on mutation patterns and genetic characteristics of CRISPR/Cas9-induced mutations in *B*. *napus* still require careful analysis. Here, we show that CRISPR/Cas9 could specifically and efficiently induce targeted mutations at one locus or multiple loci in the T0 generation of *B*. *napus* and that the mutations were stably inherited into the progeny. Only one mutated plant without the transgene was found among all of the T0 lines. This finding may be explained by the transient expression of *Cas9* or the loss of T-DNA during the regeneration of callus. No off-target mutations were identified in the CRISPR/Cas9 transgenic lines, indicating that the mutagenesis mediated by CRISPR/Cas9 is highly specific in *B*. *napus*. This study uncovers the genetic features of CRISPR/Cas9 in *B*. *napus* and indicates that CRISPR/Cas9 is a powerful tool for oilseed rape improvement by targeted gene modification.

## Results

### CRISPR/Cas9 sgRNA design and vector construction

To apply CRISPR/Cas9 in *B*. *napus*, the sgRNA-Cas9 vectors from a previous report were used^34^. The sgRNAs were designed using the online tool CRISPR-P (http://cbi.hzau.edu.cn/cgi-bin/CRISPR)^35^. In one construct, two sgRNAs for each gene with a high score were selected and their expression was driven by the Arabidopsis *U6-26* and *U6-29* promoter, respectively (Supplemental Fig. S1A). The expression of *Cas9* was driven by the *CaMV 35S* promoter. We planned to test a total of 12 *B*. *napus* genes involved in the regulation of plant development with diverse functions that belong to 4 gene families (Supplemental Table S1, and Supplemental Fig. S2 to S5). *BnaA9*.*RGA*, *BnaC9*.*RGA*, *BnaA6*.*RGA*, and *BnaC7*.*RGA* are paralogous genes of the *BnaRGA* family, orthologs of *Arabidopsis REPRESSOR OF GA1-3* (*RGA*) gene, which acts as a master repressor in gibberellic signaling. Thus, loss-of-function mutations will cause phenotypes that mimick high level of GA^36,37^. The *BnaFUL* family contains three paralogs: *BnaA9*.*FUL*, *BnaC2*.*FUL*, and *BnaC7*.*FUL*, which are the orthologs of Arabidopsis *FRUITFULL* (*FUL*) regulating silique dehiscence during flower development^38–40^. *BnaA2*.*DA2*.*1*, *BnaA2*.*DA2*.*2*, *BnaC6*.*DA2*, *BnaC5*.*DA1* and *BnaA6*.*DA1*, are paralogous genes of the *BnaDA2* and *BnaDA1* family, respectively, which are orthologs of *Arabidopsis DA2* and *DA1* (*DA: LARGE IN CHINESE*). The *da2* and *da1* mutants exhibit increases in organ size, consistent with these two genes serving as negative regulators of organ size^41,42^. Because *BnaA2*.*DA2*.*1* (*BnaA02g18880D*) and *BnaA2*.*DA2*.*2* (*BnaA02g18890D*) are adjacent genes with extremely high sequence identity (98.22%), no sgRNAs could be designed to distinguish between them. Therefore we considered *BnaA2*.*DA2*.*1* and *BnaA2*.*DA2*.*2* as one gene in our study, named *BnaA2*.*DA2*. To target one gene, two sgRNAs at gene-specific regions were designed. To target the paralogous genes of one gene family, two sgRNAs at the conserved regions were designed. We made a total of 10 constructs. Seven target one gene (Table 1) and 3 target the paralogs in one gene family (Table 2). All of these constructs were used to transform oilseed callus following standard procedures (Supplemental Fig. S1B).

**Table 1:** Percentage of mutated plants in the T0 generation induced by single-gene targeted sgRNAs.

Target Gene	sgRNA	No. of Plants examined	No. of plants with mutations	Mutation rate (%)	Homozygous mutations	
					Number	Rate (%)
*BnaA9*.*RGA*	sgRNA1	30	24	80.0	0	0
	sgRNA2		21	70.0	0	0
*BnaC9*.*RGA*	sgRNA1	29	29	100.0	0	0
	sgRNA2		29	100.0	0	0
*BnaA6*.*RGA*	sgRNA1	30	19	63.3	0	0
	sgRNA2		21	70.0	0	0
*BnaC7*.*RGA*	sgRNA1	19	1	5.3	0	0
	sgRNA2		8	42.1	0	0
*BnaA2*.*DA2*	sgRNA1	41	37	90.2	1	2.4
	sgRNA2		18	43.9	0	0
*BnaA6*.*DA1*	sgRNA1	64	41	64.1	0	0
	sgRNA2		26	40.6	4	6.25
*BnaC5*.*DA1*	sgRNA1	20	8	40.0	0	0
	sgRNA2		20	100.0	0	0
Total (average)		233		(65.3)	5	(0.6)

**Table 2:** Percentage of mutated sites in the T0 generation induced by multiple-gene targeted sgRNAs.

Gene family	Target Gene	sgRNA	No. of Plants examined	No. of plants with mutations	Mutation rate (%)	Homozygous mutations	% of homozygous mutations	% of plants mutated at different target genes
*BnaRGA*	*BnaA9*.*RGA*	sgRNA1	29	28	96.6	3	10.3	One gene is mutated: 0 Two genes are mutated: 3.4 Three genes are mutated: 6.9 Four genes are mutated: 86.2
		sgRNA2		8	27.6	0	0	
	*BnaC9*.*RGA*	sgRNA1	29	25	86.2	0	0	
		sgRNA2		9	31	0	0	
	*BnaA6*.*RGA*	sgRNA1	29	28	96.6	6	20.7	
		sgRNA2		10	34.5	0	0	
	*BnaC7*.*RGA*	sgRNA1	29	27	93.1	5	17.2	
		sgRNA2		12	41.4	0	0	
*BnaDA2*	*BnaA2*.*DA2*	sgRNA1	17	7	41.2	0	0	One gene is mutated: 35.3 ﻿Two genes are mutated: 47.1
		sgRNA2		8	47.1	1	5.9	
	*BnaC6*.*DA2*	sgRNA1	17	6	35.3	0	0	
		sgRNA2		14	82.4	1	5.9	
*BnaFUL*	*BnaA9*.*FUL*	sgRNA1	21	16	76.2	0	0	One gene is mutated: 0 Two genes are mutated: 22.2 Three genes are mutated: 55.6
		sgRNA2		18	85.7	4	19	
	*BnaC2*.*FUL*	sgRNA1	21	16	76.2	0	0	
		sgRNA2		17	81	4	19	
	*Bna﻿C7*.*FUL*	sgRNA1	21	16	76.2	0	0	
		sgRNA2		18	85.7	3	14.3	
Total			67					

### Mutation efficiency of CRISPR/Cas9 for single-gene targeted sgRNAs in *B*. *napus*

First, we evaluated the mutation efficiency of CRISPR/Cas9 for single-gene targeted sgRNAs in *B*. *napus*. The two sgRNAs used for one gene usually have different mutation rates^28,30^. Therefore, using two sgRNAs for each gene assured a high mutation rate. Because the paralogous genes have high sequence similarity in the allotetraploid *B*. *napus*, we confirmed the specificity of the primers for genotyping by direct sequencing (data not shown). A total of 233 Cas9-positive T0 transgenic lines for the 7 constructs were identified (Table 1). To accurately calculate the mutation rate, each target site was directly sequenced from the transgenic plants. We manually checked the sequencing chromatograms for each line. We concluded that mutations had been successfully introduced when the sequencing chromatograms indicated a nucleotide change (insertion, deletion or substitution) or multiple traces (overlapping peaks) at the sgRNA target sites (Supplemental Fig. S1C). Among the sgRNAs, the mutation rate varied from 5.3% to 100.0% (Table 1 and Supplemental Table S2). The average mutation rate was 65.3% (Table 1), which is comparable to other plant species^9^. Among the plants transformed with 7 different constructs, no homologous mutations were found for 5 constructs. The percentage of homologous mutations was 2.4% and 6.25%, respectively, for only two sgRNA target sites (BnaA2.DA2-sgRNA2 and BnaA6.DA1-sgRNA2) (Table 1).

### Mutation efficiency of CRISPR/Cas9 for multiple-gene targeted sgRNAs in *B*. *napus*

Simultaneously mutating paralogous genes in one gene family is important in allotetraploids, such as *B*. *napus*. To test the efficiency of simultaneous gene mutations in *B*. *napus*, two sgRNAs targeting the conserved sequences among gene family members were designed for three gene families (*BnaRGA*, *BnaDA2* and *BnaFUL*) (Table 2 and Supplemental Table 2), and a total of 67 Cas9-positive transgenic lines were created. The mutations were first detected using the T7 endonuclease I (T7E1) assay from 8 independent transgenic lines of *BnaRGA-sgRNA* (Supplemental Fig. S6). In the T7E1 assay, DNA fragments with mutations were digested by the T7E1 enzyme, whereas DNA fragments without mutations were not digested. A high mutation rate of 87.5% (7/8) occurred at the target sites of *BnaA9*.*RGA*, *BnaC7*.*RGA* and *BnaC9*.*RGA* (Supplemental Fig. S6). The DNA fragment from one line (L43) was not digested by T7E1, which is consistent with an intact target site. The results were further verified by Sanger sequencing, indicating that the T7E1 assay works well for *Brassica* (Supplemental Table S3).

We used Sanger sequencing of PCR amplicons to analyze transgenic plants when the T7E1 assay couldn’t distinguish a homozygous mutation from a non-mutation^28^. The mutation frequency at each target site for sgRNA-mediate multiple-gene targeted mutagenesis ranged from 27.6% to 96.6% (Table 2, and Supplemental Table S2), which was similar to sgRNA-mediated single-gene targeted mutagenesis (Table 2 vs. Table 1). Double, triple or quadruple mutations were readily detected, accounting for 3.4% to 86.2% of the mutants (Table 2). These data indicate that these sgRNAs efficiently target more than one locus in *B*. *napus*. A proportion of the homozygous mutations (5.9% to 20.7%) could also be identified in the T0 plants (Table 2). The homozygous mutation rate was a bit higher than we observed for the single-gene targeted sgRNAs, which may be related to the targeting efficiency of different sgRNAs^9^.

Previous studies indicate that GC content may influence the efficiency of sgRNAs and that higher GC content is usually associated with higher mutation frequencies^43–45^. In this study, the GC content was indeed positively correlated with mutation frequencies for the two sgRNAs targeting the same gene (Supplemental Table S2). However, we observed that this rule was occasionally not followed, such as in the case of *BnaC7*.*RGA-sgRNA* and *BnaDA2-sgRNA* (Supplemental Table S2).

### Variety and frequency of mutations caused by CRISPR/Cas9

**Figure 1:**
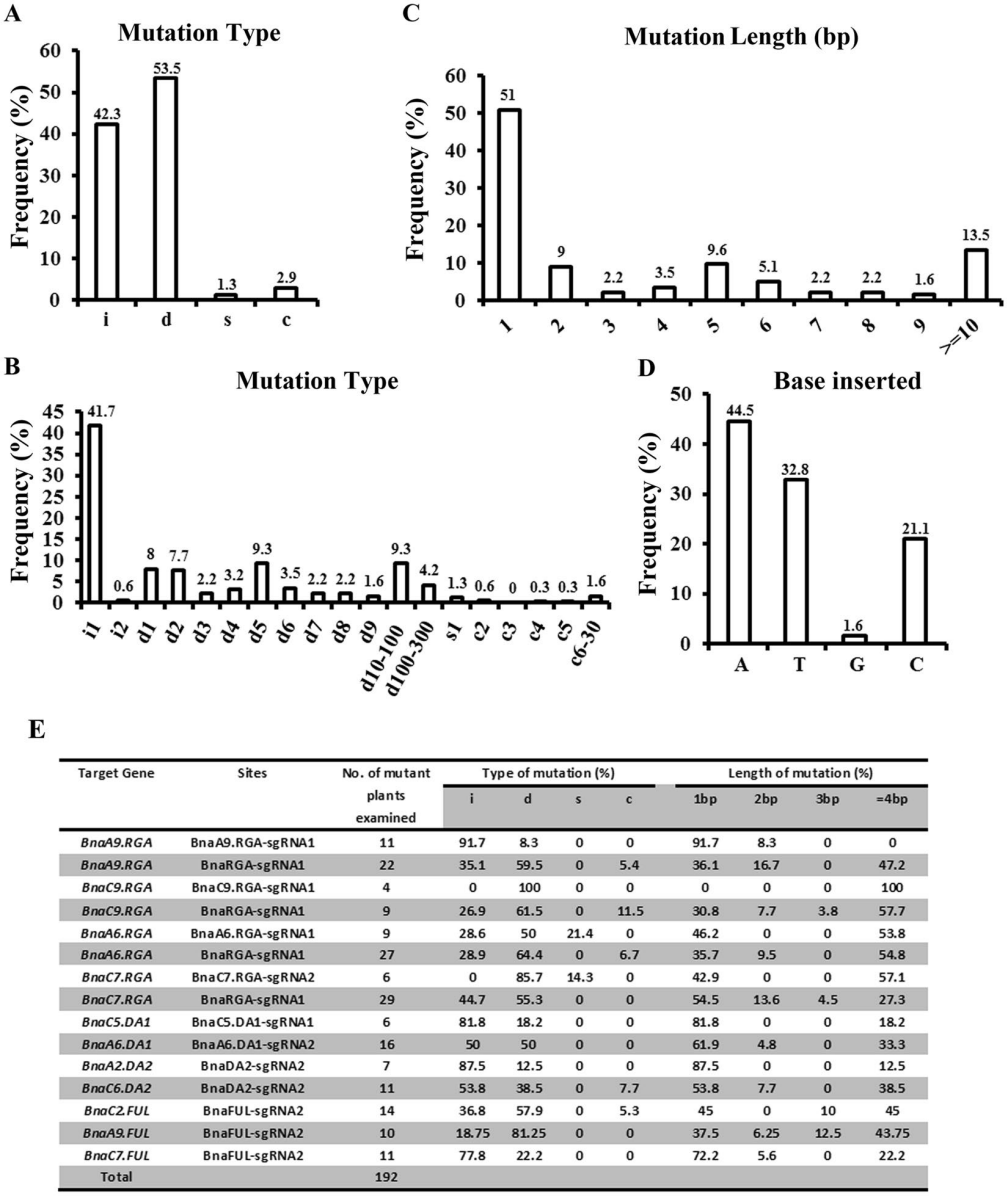
Frequency of CRISPR/Cas9-induced mutation types. (**A**,**B**) Frequency of each mutation type for all of the mutations induced by the 10 constructs in the T0 generation. i: insertion; d: deletion; s: substitution; c: combined mutation. d#, number of base pairs (bp) deleted from the target site; i#, number of bp inserted at target site, c#, number of bp combined mutations. (**C**) Frequency of different mutation lengths regardless of the mutation types using the data from (**A**). (**D**) Percentage of different bases for the 1-bp insertion (i1 in (**B**)). (**E**) Detailed characterization of the different mutation types. Notes: i: insertion, d: deletion, s: substitution, c: combined mutation (more than one mutation type in one allele).

Next, the sequencing data from all of the target sites was combined and analyzed to determine the mutation types and frequencies induced by CRISPR/Cas9 in *B*. *napus*. In total, the sequencing results of 422 PCR amplicons were analyzed by the decoding website DSDecode (http://dsdecode.scgene.com/)^46^. Part of the results were further confirmed by TA cloning and sequencing. The results are summarized in Fig. 1, and more details are provided in Supplemental Data S1. Several types of mutations were observed: deletions, insertions, substitutions, and combined mutations (i.e., more than one mutation type in one allele). Among all of the types of mutations, 53.5% were deletions, 42.3% were insertions, 2.9% were combined mutations, and 1.3% were substitutions (Fig. 1A and B). The mutations were predominantly short nucleotide changes (≤3-bp) (62.2%) (Fig. 1C), a majority of which (41.7%) were one nucleotide insertions (Fig. 1B). The length of deletions ranged from one bp to hundreds of bp, and 4.2% of mutations exhibited a >100-bp deletion. The longest deletion was 270 bp (Supplemental Data S1). These long fragment deletions are caused by the simultaneous repairing of two DSBs generated by two sgRNAs in one construct in our system^33^.

The cleavage site of Cas9 is usually 3-bp upstream of the PAM sequence^47^. Our results demonstrated that 91.3% of the mutations indeed occurred at this position. Additionally, 0.7%, 1.4%, 4.3% and 2.2% of the mutations occurred at the 1^st^, 2^nd^, 4^th^ and 5^th^ base from the PAM site, respectively (Supplemental Fig. S7A). In more detail, all of the 1-bp insertions (100%) and most of the 1-bp deletions (87.5%) were located 3-bp upstream of the PAM site (Supplemental Fig. S7B). When the base composition of the 1-bp insertions was examined, most of them were A (44.5%) or T (32.8%) insertions. Unexpectedly, the percentage of C insertions was 21.1%, which is much higher than was observed in rice (7.6%)^30^ and tomato (9.3%)^28^ (Fig. 1D).

We further compared the mutation types for each target gene. For example, all *BnaC9*.*RGA-sgRNA1* mutations were deletions. Mutations in *BnaA9*.*RGA-sgRNA1* were predominantly insertions (91.7%). The *BnaA6*.*RGA-sgRNA1* induced substitutions at a frequency of 21.4%, which is much higher than other sgRNAs (Fig. 1E). In general, short indels (≤3-bp) were most abundant. However, the deletion length was more than 4-bp in the entire *BnaC9*.*RGA-sgRNA1* target sites (Fig. 1E). We also observed different types of mutations for different loci targeted by the same sgRNA. For example, *BnaFUL-sgRNA2* induced 81.25% deletions in *BnaA9*.*FUL* and 77.8% insertions in *BnaC7*.*FUL* (Fig. 1E). Although there were a limited number of mutations analyzed for some targets, these results provide strong evidence that the types of mutations vary at distinct target sites.

### Genotypes of CRISPR/Cas9 mutants in the T0 generation

There are two alleles for each gene, both of which might be mutated by CRISPR/Cas9. Thus, CRISPR/Cas9 could produce five genotypes: homozygote (the two alleles have the same mutation), bi-allele (the two alleles have different mutations), heterozygote (only one allele is mutated), chimera (more than two different mutations exist), and WT-type (no mutation). To estimate the proportion of each genotype among the T0 mutants, we randomly chose 117 single-gene targeted and 66 multiple-gene targeted sgRNA lines containing *Cas9* insertions to analyze the mutations of each targeted site by Sanger sequencing. A total of 425 PCR amplicons were decoded by the DSDecode website. Additionally, 14 PCR amplicons were analyzed by inserting them into a TA vector and sequencing 10 individual clones for each amplicon. We obtained results from 439 amplicons. The genotype data are summarized in Table 3.

**Table 3:** Genotypes of T0 transgenic plants. ‘—’: the sequencing results were not well decoded by DSDecode.

Target gene	Sites	No. of examined plants	Genotype				
			Homozygote	Heterozygote	Bi-allele	Chimera	WT
*BnaA9*.*RGA*	BnaA9.RGA-sgRNA1	20		8 (40.0%)	1 (5.0%)	6 (30%)	5 (25.0%)
	BnaA9.RGA-sgRNA2	—					
*BnaA9*.*RGA*	BnaRGA-sgRNA1	23	3 (13.0%)	1 (4.3%)	13 (56.5%)	5 (21.8%)	1 (4.3%)
	BnaRGA-sgRNA2	8		5 (62.5%)	2 (25.0%)		1 (12.5%)
*BnaC9*.*RGA*	BnaC9.RGA-sgRNA1	4		4 (100.0%)			
	BnaC9.RGA-sgRNA2	5		5 (100.0%)			
*BnaC9*.*RGA*	BnaRGA-sgRNA1	11		1 (9.1%)	4 (36.4%)	5 (45.5%)	1 (9.1%)
	BnaRGA-sgRNA2	4		2 (50.0%)	1 (25.0%)		1 (25.0%)
*BnaA6*.*RGA*	BnaA6.RGA-sgRNA1	25		4 (16.0%)	3 (12.0%)	10 (40.0%)	8 (32.0%)
	BnaA6.RGA-sgRNA2	4			3 (75.0%)	1 (25.0%)	
*BnaA6*.*RGA*	BnaRGA-sgRNA1	29	6 (20.7%)	2 (6.9%)	14 (48.3%)	6 (20.7%)	1 (3.4%)
	BnaRGA-sgRNA2	8		1 (12.5%)	1 (12.5%)	5 (62.5%)	1 (12.5%)
*BnaC7*.*RGA*	BnaC7.RGA-sgRNA1	19		1 (5.3%)			18 (94.7%)
	BnaC7.RGA-sgRNA2	19		3 (15.8%)	1 (5.3%)	4 (21.1%)	11 (57.9%)
*BnaC7*.*RGA*	BnaRGA-sgRNA1	30	5 (16.7%)	3 (10.0%)	16 (53.3%)	5 (16.7%)	1 (3.3%)
	BnaRGA-sgRNA2	11		8 (72.7%)		3 (27.3%)	
*BnaC5*.*DA1*	BnaC5.DA1-sgRNA1	20		3 (15.0%)	3 (15.0%)	4 (20.0%)	10 (50.0%)
	BnaC5.DA1-sgRNA2	17				17 (100.0%)	
*BnaA6*.*DA1*	BnaA6.DA1-sgRNA1	—					
	BnaA6.DA1-sgRNA2	29	4 (13.8%)	4 (13.8%)	8 (27.6%)	5 (17.2%)	8 (27.6%)
*BnaA2*.*DA2*	BnaDA2-sgRNA1	—					
	BnaDA2-sgRNA2	17	1 (5.9%)	5 (29.4%)	1 (5.9%)	2 (11.8%)	8 (47.1%)
*BnaC6*.*DA2*	BnaDA2-sgRNA1	16	1 (6.25%)	1 (6.25%)		7 (43.75%)	7 (43.75%)
	BnaDA2-sgRNA2	16	1 (6.25%)	3 (18.75%)	2 (12.5%)	8 (50.0%)	2 (12.5%)
*BnaC2*.*FUL*	BnaFUL-sgRNA1	15	1 (6.7%)	1 (6.7%)		9 (60.0%)	4 (26.6%)
	BnaFUL-sgRNA2	16	4 (25.0%)	3 (18.75%)	5 (31.25%)	2 (12.5%)	2 (12.5%)
*BnaA9*.*FUL*	BnaFUL-sgRNA1	19	1 (5.3%)	1 (5.3%)		13 (68.4%)	4 (21.0%)
	BnaFUL-sgRNA2	19	4 (21.1%)	1 (5.3%)	5 (26.3%)	7 (36.8%)	2 (10.5%)
*BnaC7*.*FUL*	BnaFUL-sgRNA1	17				13 (76.5%)	4 (23.5%)
	BnaFUL-sgRNA2	18	3 (16.7%)	1 (5.6%)	7 (38.9%)	3 (16.7%)	4 (22.2%)
Total		439	34 (7.7%)	71 (16.1%)	90 (20.5%)	140 (31.9%)	104 (23.7%)

**Figure 2:**
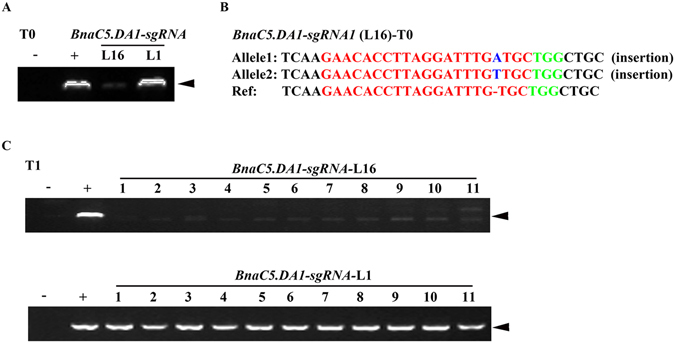
Genotyping of the *BnaC5*.*DA1-sgRNA*-L16 plants in the T0 and T1 generation. (**A**) Cropped gel image showing the PCR products derived from *Cas9* in *BnaC5*.*DA1-sgRNA*-*L16* in the T0 generation. (**B**) The genotype of *BnaC5*.*DA1-sgRNA*-*L16* in the T0 generation. The PAM sequence is indicated with green. The sgRNA is indicated with red. Mutation sites are indicated with blue. (**C**) Cropped gel image showing the PCR products of *Cas9* in different progeny from *BnaC5*.*DA1-sgRNA*-*L16* in the T1 generation. +:﻿ *pKSE401* was used as a positive control; −: gDNA of WT was used as a negative control. *BnaC5*.*DA1-sgRNA-L1* (T0 and T1) was used as a positive control for the *Cas9* insertion. The arrowheads indicate the position of the amplicons.

Based on the genotyping results, 7.7% (34/439) sites were homozygous and 20.5% (90/439) sites were bi-allelic. Thus, a total of 28.2% had defects in both alleles (Table 3). Among all the T0 homozygotes, 70.6% (24/34) of them carried a 1-bp insertion (i1) or a 1-bp deletion (d1), indicating that these two mutation types happened at a high frequency in T0 homozygous plants. For the bi-allelic mutations, the predominant type carried a combination of i1ai1b mutations (a and b indicate two different nucleotides) (19.4%), followed by i1d1 (i: insertion, d: deletion) (12.0%) (Supplemental Fig. S8). The frequency of heterozygotes and chimeras was 16.1% (71/439) and 31.9% (140/439), respectively. No mutations were found in 23.7% (104/439) of the sites. Interestingly, we identified one line called *BnaC5*.*DA1-sgRNA1*-L16 that has an A/T insertion at the expected target site without the Cas9 insertion (Fig. 2A and B, Supplemental Fig. S9), which was confirmed in the next generation (Fig. 2C, Supplemental Table S3). Based on these data, we suggest that it is possible to get T-DNA-free plants in the first generation by regenerating oilseed callus.

Previous studies showed that different CRISPR/Cas9-induced mutations could arise from different tissues^27,29^. To test this possibility in *B*. *napus*, we genotyped 6 single-gene targeted and 9 multiple-gene targeted sgRNA lines with mixed tissues of leaves, shoots, and flower buds. A total of 21 PCR amplicons were used for the sequencing analysis. For homozygous and bi-allelic lines, the same mutations were detected in leaves and mixed tissues (Supplemental Tables S3 and S4). However, we found that 36.4% (4/11) of heterozygous and chimeric plants had new mutations in shoots and flower buds (Supplemental Tables S3 and S4), indicating that the wild type alleles from these plants were further mutated by CRISPR/Cas9.

**Figure 3:**
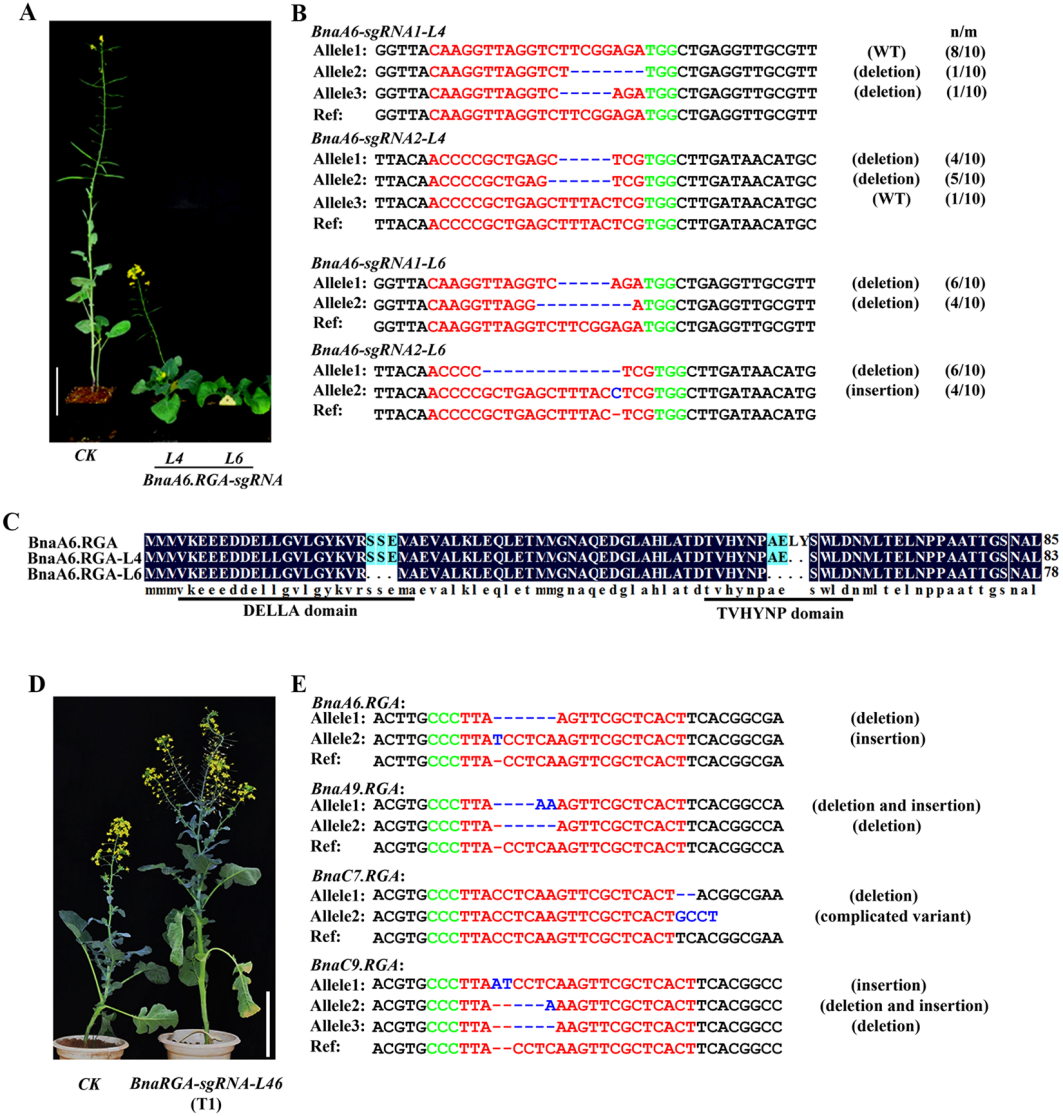
Phenotype and genotype of a *BnaA6*.*RGA-sgRNA* single mutant (T0) and a *BnaRGA-sgRNA* quadruple mutant (T1). (**A**) Morphology of *BnaA6*.*RGA-sgRNA* transgenic plants at the same age. L4 and L6: two individual T0 transgenic lines. CK: *BnaA6*.*DA1-sgRNA* (L10). Bar = 15 cm. (**B**) Genotype of *BnaA6*.*RGA-sgRNA* lines (L4 and L6) in the T0 generation. (**C**) Amino acid sequence alignment of the target sites derived from WT and two *BnaA6*.*RGA-sgRNA* transgenic lines (L4 and L6). The DELLA and TVHYNP domains are underlined. The amino acid deletion in the DELLA domain is caused by sgRNA1. The amino acid deletion in the TVHYNP domain is caused by sgRNA2. (**D**) Image showing the morphology of the quadruple mutant induced by *BnaRGA-sgRNA* (L46-T1) at the same age as the control. CK: WT plant. Bar = 15 cm. (**E**) Genotype of the quadruple mutant isolated from *BnaRGA-sgRNA-L46* in the T1 generation. The PAM sequence is indicated with green. The sgRNA is indicated with red. The mutation sites are indictaed with blue. For the term n/m, m indicates the number of clones examined, and n indicates the number of clones showing the indicated genotype.

When 3 bp or multiples of 3 bp are deleted from an exon without affecting the reading frame, a few amino acids may be deleted from the middle of a protein, which may alter its biochemical properties. *BnaA6*.*RGA* encodes a DELLA protein that negatively regulates GA signaling in many plant species. It is reported that deletion of the conserved TVHYNP domain resulted in a dwarf phenotype and insensitivity to exogenous GA^36,37^. Among the transgenic lines of *BnaA6*.*RGA-sgRNA*, two T0 lines (T0-L4 and T0-L6) were dwarf (Fig. 3A), and the phenotype was inherited in the T1 generation (Supplemental Fig. S10). According to the sequencing results, the T0-L4 and T0-L6 plants have a 6- and 12-nt deletion at the target sites, respectively (Fig. 3B, Supplemental Table S3), that causes a 2- and 4- amino acid deletion in the TVHYNP domain (Fig. 3C).

### CRISPR/Cas9-induced mutations are stable and inheritable in *B*. *napus*

*B*. *napus* is an allotetraploid crop^31^. Given the high ploidy level, it is interesting to test whether the mutations caused by CRISPR/Cas9 are stable and inheritable in *B*. *napus*. A total of 323 T1 plants derived from the T0 (Table 3) were examined for the genotypes at the target sites (Table 4; Supplemental Table S3). Due to the large number of samples, sequencing results from the entire set of DNA amplicons were directly decoded by DSDecode. Therefore, only homozygotes, wild type, bi-alleles and heterozygotes were clearly identifiable. We could not accurately decode chimeras using this approach. Thus, chimeras are indicated with an ‘h’ for heterogeneous (Table 4). The T1 progeny of 3 T0 homozygotes were still homozygous with the same mutations, indicating that the mutations in these transgenic lines were stable (Table 4). If the bi-allele and heterozygous genotypes are inherited normally, the segregation ratio is expected to be 1xx:2xy:1yy. However, unexpected segregation ratios of 0:2:13, 3:12:0, 0:20:0 and 11:1:2 were observed among the T1 progeny of BnaDA2-sgRNA2-L12 (targeted to *BnaA2.DA2*), BnaDA2-sgRNA1-L20 (targeted to *BnaC6.DA2*), BnaRGA-sgRNA1-L27 (targeted to *BnaA9.RGA*) and BnaC5.DA1-sgRNA-L1, respectively (Table 4), which might be due to unequal inheritance frequencies of the two alleles. New mutation types were identified in the T1 generation for heterozygotes. One possible reason is that Cas9 could be still functional at the non-mutated allele at the targeted region. As expected, the T1 segregation patterns of T0 chimeras were more diverse and less predictable (Table 4). Irrespective of bi-allelic, heterozygous or chimeric mutants, homozygotes were found in T1 generation (Table 4). Unexpectedly, no T1 descendants of WT showed any novel mutations even in the presence of the Cas9 transgene (Table 4), indicating that the CRISPR/Cas9 was not functional in these transgenic lines, possibly due to lower expression of Cas9 and/or the guide RNA.

**Table 4:** Segregation patterns of CRISPR/Cas9-induced mutations in the T1generation.

Target gene	sgRNA	Line	T0			T1		
			Zygosity^◐^	Genotype	Cas9^§^	Segregation ratio	P value	Cas9^§^
*BnaA6*.*RGA*	BnaRGA-sgRNA1	L40	Homozygote	d1d1	+	8 d1d1		7(+), 1(−)
*BnaA6*.*RGA*	BnaRGA-sgRNA1	L44	Homozygote	i1i1	+	17 i1i1		12(+), 5(−)
*BnaA9*.*RGA*	BnaRGA-sgRNA1	L44	Homozygote	d6d6	+	17 d6d6		12(+), 5(−)
*BnaA6*.*RGA*	BnaA6.RGA-sgRNA1	L5	Bi-allele	d5, d6	+	9 d5d5: 9 d5d6:2 d6d6^※^	0.08*	16(+), 4(−)
*BnaA6*.*RGA*	BnaA6.RGA-sgRNA1	L6	Bi-allele	d5, d9	+	4 d5d5: 5 d5d9:0 d9d9^※^	0.02*	7(+), 2(−)
*BnaA6*.*RGA*	BnaRGA-sgRNA1	L46	Bi-allele	i1, d5	+	6 i1i1: 9 i1d5: 2 d5d5	0.38	15(+), 2(−)
*BnaA9*.*RGA*	BnaRGA-sgRNA1	L27	Bi-allele	d1, d5	+	0 d1d1:20 d1d5:0 d5d5	3.1E-07*	16(+), 4(−)
*BnaC7*.*RGA*	BnaRGA-sgRNA1	L38	Bi-allele	i1, d2	+	3 i1i1: 8 i1d2: 4 d2d2	0.90	12(+), 3(−)
*BnaC5*.*DA1*	BnaC5.DA1-sgRNA1	L1	Bi-allele	i1a, i1b	+	11 i1ai1a:1 i1ai1b: 2 i1bi1b	2.3E-07*	14(+)
*BnaC5*.*DA1*	BnaC5.DA1-sgRNA1	L16	Bi-allele	i1a, i1b	−	4 i1ai1a: 8 i1ai1b: 4 i1bi1b	1.0	16(−)
*BnaA2*.*DA2*	BnaDA2-sgRNA2	L12	Bi-allele	i1, d4	+	0 i1i1: 2 i1d4: 13 d4d4	0.04*	15(+)
*BnaC6*.*DA2*	BnaDA2-sgRNA2	L20	Bi-allele	i1a, i1b	+	3 i1ai1a:12 i1ai1b: 0 i1bi1b	1.8E-05*	13(+), 2(−)
*BnaA9*.*FUL*	BnaFUL-sgRNA2	L15	Bi-allele	i1, d1	+	5 i1i1: 10 i1d1: 1 d1d1	0.008*	13(+), 3(−)
*BnaC7*.*FUL*	BnaFUL-sgRNA2	L23	Bi-allele	i2, d5	+	3 i2i2: 8 i2d5: 5 d5d5	0.77	15(+), 1(−)
*BnaA6*.*RGA*	BnaRGA-sgRNA1	L33	Heterozygote	i1, WT	+	3 i1ai1a: 7 i1ai1b: 2 i1bi1b: 1 i1aWT	0.31	12(+), 1(−)
*BnaA9*.*RGA*	BnaRGA-sgRNA1	L40	Heterozygote	d22, WT	+	7 i1i1: 2 d22d22: 7 h^※^		15(+), 1(−)
*BnaC7*.*RGA*	BnaRGA-sgRNA1	L40	Heterozygote	i1a, WT	+	5 i1ai1a: 9 i1ai1b: 2 i1bi1b	0.31	15(+), 1(−)
*BnaA6*.*DA1*	BnaA6.DA1-sgRNA2	L6	Heterozygote	i1, WT	+	3 i1i1: 10 i1WT: 2 WTWT	0.90	15(+)
*BnaA6*.*DA1*	BnaA6.DA1-sgRNA2	L24	Heterozygote	d1, WT	+	4 d1d1: 6 d1WT: 3 WTWT		13(+)
*BnaA2*.*DA2*	BnaDA2-sgRNA2	L1	Heterozygote	i1, WT	+	11 i1ai1b: 5 WTWT	0.06*	12(+), 4(−)
*BnaA2*.*DA2*	BnaDA2-sgRNA2	L20	Heterozygote	i1, WT	+	3 i1ai1a: 6 i1aWT: 4 i1ai1b: 2 WTWT		13(+), 2(−)
*BnaC6*.*DA2*	BnaDA2-sgRNA2	L1	Heterozygote	i1, WT	+	0 i1i1: 10 i1WT: 6 WTWT	0.31	12(+), 4(−)
*BnaA6*.*RGA*	BnaA6.RGA-sgRNA2	L4	Chimera	d6, d5, WT	+	3d5d5: 16 h^※^		15(+), 4(−)
*BnaC9*.*RGA*	BnaRGA-sgRNA1	L27	Chimera	d1, d5, d33, d36	+	2d1 h:10 d5 h: 2d33 h: 5d36 h^※^		15(+), 4(−)
*BnaC9*.*RGA*	BnaRGA-sgRNA1	L40	Chimera	i1, d5, WT	+	16 d19h^※^		15(+), 1(−)
*BnaC6*.*DA2*	BnaDA2-sgRNA1	L12	Chimera	d4, h	+	5 d4d4: 2 d4h: 2 d7h: 3 d3d4: 1 WTWT: 2 h^※^		15(+)
*BnaA9*.*RGA*	BnaRGA-sgRNA1	L43	WT	WT^♢^	+	10 WT WT		10(+)
*BnaA6*.*DA1*	BnaA6.DA1-sgRNA2	L15	WT	WT^♢^	+	10 WT WT		10(+)
*BnaA6*.*DA1*	BnaA6.DA1-sgRNA2	L25	WT	WT^♢^	+	10 WT WT		10(+)

◐The zygosity of the homozygote, bi-allele, and heterozygote in T0 plants. ^♢^WT, no mutations were identified. ^§^Presence of Cas9 sequence: +, Cas9 positive; −, Cas9 negative; n.d., Not determined. ^※^More data are needed to fully explain the T1 genotypes. d#, # of bp deleted from a target site; d#a, same number of deletion at one site; d#b, same number of deletion at other sites; i#, # of bp inserted at target site; i#a, same number of insertion at one site; i#b, same number of insertion of different nucleotide at the same site; c#, combined mutation; h, heterogeneous, more than one sequence detected in the sample. χ^2^ test *P value < 0.1.

Because a majority of genes have multiple paralogs with functional redundancy in *B*. *napus* (allotetraploid), single gene knockouts probably will not show obvious phenotypes^48^. In this study, the T1 quadruple mutant of *BnaRGA* grew longer stems than control plants (Fig. 3D and Supplemental Fig. S11). Genotyping results indicated that the four paralogs of *BnaRGA* could be efficiently knocked out with homozygous or bi-allelic mutations in the T0 generation (Fig. 3E), and inherited in the T1 generation (Supplemental Table S3). Together, these data make the case that CRISPR/Cas9 has great advantages for gene function studies in *B*. *napus*.

Genetically manipulated materials without T-DNA insertions are largely favored for crop improvement and should be more public acceptable. Indeed, the T-DNA insertions in 76.2% of the T0 lines did not cosegregate with the CRISPR/Cas9-induced mutations and were therefore removed in the next generation. The average value for T1 progeny lacking the Cas9 transgene was 10.9% when analyzed using Cas9-specific primers (Supplemental Fig. S12, Supplemental Table S5). The homozygote and bi-allele genotypes were stably passed to subsequent generations regardless of whether the T-DNA was present. These data indicate that CRISPR/Cas9 is an effective tool for the improvement of *B*. *napus*.

### No off-targets were discovered in *B*. *napus*

Low-frequency examples of off-target cleavage have been reported for CRISPR/Cas9 in plants^28,30^. To detect the off-target events in oilseed plants, potential off-target loci following PAM sequences that are highly homologous to the sgRNAs of *BnaA9*.*RGA*, *BnaC9*.*RGA*, *BnaA6*.*RGA* and *BnaC7*.*RGA* were predicted using the online tool CRISPR-P (http://cbi.hzau.edu.cn/cgi-bin/CRISPR)^35^. At least three of the most likely off-target sites for each sgRNA were examined in a total of 50 randomly selected T0 and T1 plants using gene-specific primers (Supplemental Table S6). Previous reports have indicated that the 12 nucleotides of “seed sequence” located in the target site and adjoining the PAM are critical for recognition specificity and cleavage efficiency of Cas9^30,49,50^. In the off-target sequences, there were 1 to 3 mismatches in the ‘seed sequence’. No mutations were found in the putative off-target sites (Table 5), indicating that CRISPR/Cas9-mediated mutagenesis is specific in oilseed plants.

**Table 5:** Detection of mutations at the putative CRISPR/Cas9 off-target sites in the T0 and T1 generation.

Target	Putative off-target sites	Putative off-target locus	Putative off-target sequence	No. of mismatch bases	No. of plants examined	No. of mutations
*BnaA9*.*RGA-sgRNA1*	OFF1	chrC09_random:+1591592	GAGGTCGTC**A**GAGATGGC**G**GAGG	2	50	0
	OFF2	chrC07:-27532718	**C**AGGTCGTC**G**GAGATGGC**T**GAGG	3	50	0
	OFF3	chrC09_random:+1591705	ACCCGTCGGAGCT**T**TACTCGTGG	1	50	0
*BnaC9*.*RGA-sgRNA1*	OFF1	chrA09:−11644254	CAAGGTGAGGTCGTC**C**GAGATGG	1	50	0
	OFF2	chrA09:−11644135	ACCCGTCGGAGCT**C**TACTCGTGG	1	50	0
	OFF3	chrA06:+23009274	ACCC**CG**C**T**GAGCTTTACTCGTGG	3	50	0
*BnaA6*.*RGA-sgRNA1*	OFF1	chrC09:+5730399	CAAGGT**A**AGGTC**G**TCGGAGATGG	2	50	0
	OFF2	chrC07:−33809295	CAAGGT**A**AGGTC**G**TCGGAGATGG	2	50	0
	OFF3	chrC07:−27532724	CAAGGT**C**AGGTC**G**TCGGAGATGG	2	50	0
	OFF4	chrC09_random:+1591705	ACCC**GT**C**G**GAGCTTTACTCGTGG	3	50	0
*BnaC7*.*RGA-sgRNA1*	OFF1	chrC07:−33809289	**A**AGGTCGTCGGAGATGGC**G**GAGG	2	50	0
	OFF2	chrC09:+5730405	**A**AGGTCGTCGGAGATGGC**G**GAGG	2	50	0
	OFF3	chrA06:+23009161	**T**AGGTC**T**TCGGAGATGGCTGAGG	2	50	0

The PAM motif (NGG) is indicated by underlines; mismatched bases are shown in red color.

## Discussion

### Highly efficient target gene mutagenesis by CRISPR/Cas9 in *B*. *napus*

Compared to traditional mutagenesis strategies, CRISPR/Cas9 targeted genome editing is precise and efficient. Therefore, CRISPR/Cas9 is extremely useful for gene function studies and crop improvement^25,30,51,52^. In this work, to systematically assess the application of CRISPR/Cas9 in *B*. *napus*, 12 genes were selected for targeted mutagenesis. The mutation frequency ranged from 5.3% to 100%. Mutation frequencies are similar in most of plants. Based on these data, we suggest that variations in genome size do not significantly influence the efficiency of targeted genome editing mediated by the CRISPR/Cas9 system^9^. Indeed, we found that CRISPR/Cas9 efficiently created homozygous and bi-allelic mutations that were stably maintained during plant regeneration in *B*. *napus*, which has a high ploidy level. CRISPR/Cas9 induced these two genotypes not only in one target gene, but also in several paralogous genes without any reduction of efficiency (Tables 3 and 4). Due to a high ploidy level, paralogs in one gene family with functional redundancy usually exist in *B*. *napus*. Therefore, one-gene knockouts do not lead to phenotypes in *B*. *napus*. For example, we observed no significant differences in the single-gene knockout mutant of *BnaA6*.*RGA-sgRNA* and *BnaA9*.*RGA-sgRNA* relative to WT plants (Supplemental Fig. S13). In contrast, we observed significantly longer stems in the quadruple mutant of *BnaRGA-sgRNA* relative to WT plants (Fig. 3D and Supplemental Fig. S11). Our results demonstrate that using sgRNAs derived from conserved regions, CRISPR/Cas9 can simultaneously knockout a group of paralogous genes. Thus, CRISPR/Cas9 is able to create ideal materials for functional studies in oilseed rape.

Based on a comparison of T0 and T1 generation plants (Table 4), it is clear that the mutations of homozygotes and most of the bi-alleles are stably inherited regardless of whether T-DNA is present. However, heterozygotes retain the wild type alleles (Table 4) that have the potential to be mutated. Thus, new mutation types could arise from different tissues in both T0 and T1 plants probably due to cell-autonomous mutagenesis in those tissues (Supplemental Table S3), consistent with other reports^28,30^. In the meantime, the wild type alleles in the heterozygotes and chimeras could also be mutated as plants are propagated (Table 4). It is noticeable that no new mutations were generated for the WT plants when the T0 plants were propagated to the T1 generation (Table 4), which is inconsistent with the high mutagenesis efficiency of CRISPR/Cas9. We assume that the expression level of CRISPR/Cas9 is low because of position effects, post-transcriptional gene silencing, or the Cas9 protein is somehow inactivated^9,30^.

### Cas9 and sgRNAs affect the genome editing efficiency

One construct expressing two sgRNAs for each targeted gene assured a high mutation rate per gene^9^. A comparison of mutation frequencies obtained using this strategy indicated that sgRNAs with higher GC contents are usually associated with higher mutation frequencies, with the exception of *BnaC7*.*RGA-sgRNA* and *BnaDA2-sgRNA* (Supplemental Table S2). Our findings indicate that GC content may influence the efficiency of sgRNA, which is consistent with previous work^43–45^. Unexpectedly, the mutation rate of each gene is usually determined by the sgRNA with the higher mutation rate. In other words, sgRNAs that induce higher mutation rates appear to compensate for sgRNAs that induce lower mutations rates. The expression level and pattern of *Cas9* and *sgRNAs* driven by different promoters have a major influence on genome editing efficiency^9^. In *Arabidopsis* and maize, tissue specific or plant endogenous promoters that drive Cas9 expression greatly increased the mutation frequency compared to constitutive promoters, such as the *CaMV 35S promoter*^27,53–56^. The results from soybean and liverwort demonstrated that mutation frequencies could be increased 2 to 7-fold when the intrinsic *U6* promoter is used to drive sgRNA expression, compared to the *Arabidopsis U6* promoter^57,58^. In contrast, high mutation frequencies were observed in *B*. *napus* when using either constitutive promoters or the *Arabidopsis U6* promoter. Although these promoters work well for generating mutations in *B*. *napus*, particular sites were edited at a low frequency (e.g., BnaC7.RGA-sgRNA1, 5.3%). Tissue-specific promoters or more active promoters may increase the frequency of mutagenesis by increasing the expression level of *Cas9* and *sgRNAs* during plant regeneration.

### Potential T-DNA free mutants were generated by CRISPR/Cas9 in *B*. *napus*

T-DNA-free mutants could be generated by self-crossing or backcrossing in the T1 generation, which provides reliable material for crop improvement^28,30,58^. Indeed, 11.3% of the T1 mutant plants lacked the Cas9 transgene (Supplemental Table S5). Additionally, we found one Cas9-negative T0 line—*BnaC5*.*DA1-sgRNA-L16*—with a mutation at the target site in both the T0 and T1 generations (Fig. 2 and Table 4). One possible explanation for this finding is that the CRISPR/Cas9 cassette was lost during cell division or that transient expression of *Cas9* is responsible for this mutation. Three pairs of primers were used to confirm the loss of the T-DNA insert (Supplemental Fig. S12). However, whole genome resequencing would be a perfect approach to rule out the presence of any T-DNA fragments^59^. For some lines, very few or no plants lost the CRISPR/Cas9 cassette, which may be due to multiple CRISPR/Cas9 cassette insertions in the genome. In this case, increasing the population size or a cross with WT is needed to remove the transgenes. Recently, two approaches have been developed for generating Cas9-free mutants in one generation. One strategy involves delivering the Cas9-sgRNA ribonucleoprotein complex or the CRISPR-Cas9 DNA/RNA into plant cells using particle bombardment or protoplast transfection^60,61^. The other strategy uses fluorescent proteins as markers to facilitate the selection of transgene-free plants^62^. These reports provide new strategies screen and isolate Cas9 free material and speed up molecular breeding in the future.

In summary, we demonstrated that the CRISPR/Cas9 is a highly efficient tool for genome editing in *B*. *napus*. We found that sgRNAs derived from conserved sequences could simultaneously induce homozygous or bi-allelic mutations at multiple loci. Therefore, CRISPR/Cas9 provides a powerful tool for studying gene function in oilseed and the fastest method for the breeding of polyploid crops. Moreover, the targeted gene modification mediated by CRISPR/Cas9 is safer for both human health and the environment because it is possible to remove the foreign DNA following the mutagenesis of the target DNA. Thus, CRISPR/Cas9 is useful for both basic and applied research in *B*. *napus*.

## Material and Methods

### Plant materials and growth conditions

The *B*. *napus* variety *Westar* was transformed with *Agrobaterium*. The transgenic lines and wild-type plants were grown in a greenhouse at 22 °C, 70% relative humidity, and in a photoperiod containing16 h of light/8 h of dark. Mature seeds were collected from T0 plants and germinated for 7 days at 22 °C, on petri dishes, in a photoperiod containing 16 h of light/8 h of dark. The seedlings were then transferred to soil and grown in the greenhouse.

### Plant transformation

The procedure for *Agrobaterium*-mediated transformation was carried out as previously described^63^. Briefly, the explants were incubated in *Agrobaterium*-infection buffer (MS salts 4.43 g/L; sucrose 30 g/L; acetosyringone 100 mM; pH 5.8–5.9) for 20 min, then transferred to M1 medium plates (MS salts 4.43 g/L; sucrose 30 g/L; acetosyringone 100 mM; mannitol 18 g/L; 2,4-D 1 mg/L; kinetin 0.3 mg/mL; pH 5.8–5.9) in the dark, for 48 h. Afterwards, the explants were transferred to M2 medium plates (MS salts 4.43 g/L; sucrose 30 g/L; acetosyringone 100 mM; mannitol 18 g/L; AgNO_3_ 4 mg/L; 2,4-D 1 mg/L; kinetin 0.3 mg/mL; Timentin 270 mg/L; pH 5.8–5.9), with proper antibiotics to select for transgenic callus. The calli were transferred to M3 medium plates (MS salts 4.43 g/L; glucose 10 g/L; xylose 0.25 g/L; zeatin 2 mg/L; IAA 0.1 mg/L; Timentin 270 mg/L; pH5.8–5.9) and then transferred to M4 medium (MS salts 2.22 g/L; sucrose 10 g/L; IBA 0.5 mg/L; Timentin 135 mg/L; pH5.8–5.9) to allow the shoots and roots to regenerate, respectively. We tested for T-DNA insertions using *Cas9*-specific primers, *NPTII*-specific primers, and *pKSE401*-specific primers for all of the T0 transgenic lines (Table S6). The positive plants were transferred to soil for further analysis.

### Vector construction

The sgRNA-Cas9 plant expression vectors were constructed as previously described with minor modifications^34^. The target sgRNA sequences were designed using the web server CRISPR-P (http://cbi.hzau.edu.cn/cgi-bin/CRISPR)^35^, and then the sequences were further analyzed using the CRISPR Primer Designer software^50^. Using *pCBC-DT1T2* as the template, two *AtU6 promoter-sgRNA-AtU6 terminator* cassettes were amplified by PCR using the primers listed in Table S8. Then the PCR fragments were inserted into *pKSE401* by Golden Gate Assembly^64^, and confirmed by Sanger sequencing. The *pKSE401-sgRNA* vectors were used for plant transformation.

### Genotyping and T7E1 assay

To analyze the mutations caused by CRISPR/Cas9, genomic DNA was extracted from each transgenic plant using the CTAB method (Molecular Cloning, 3rd edition). The flanking sequence around the CRISPR target sites was amplified by PCR using gene-specific primers (Supplemental Table S6). First, each PCR amplicon generated from WT genomic DNA was sub-cloned into the pGEM-T easy vector (A3600, Promega, USA) to confirm the primer specificity by Sanger sequencing. Then, most of the amplicons were directly sequenced to analyze the mutations. For the complex mutations, the amplicons were first sub-cloned into the pGEM-T easy vector, and about 10 clones of each amplicon were individually sequenced by Sanger sequencing.

The T7E1 assay was carried out as previously described with minor modifications^27^. 300 ng of purified PCR products were denatured and annealed in a thermocycler using the following program: 5 min at 95 °C, 95 to 85 °C using a ramp rate of −2 °C/sec, 85 to 25 °C using a ramp rate of −0.2 °C/sec. The annealed PCR products were digested with T7E1 nuclease (M0302S, New England Biolabs) which specifically cleaves DNA with mismatches at 37 °C for 1 h. Digested PCR products were analyzed using agarose gel electrophoresis containing ethidium bromide.

### Off-target analysis

The potential off-target sites were predicted using CRISPR-P (http://cbi.hzau.edu.cn/cgi-bin/CRISPR). The top-ranking potential off-target sites containing fewer than 3-bp mismatches in the 12-bp seed sequence were selected for validation. The genomic DNA sequences surrounding the potential off-target sites were amplified by PCR using gene-specific primers (Supplemental Table S6). PCR products were analyzed by direct sequencing.

### Sequencing chromatogram decoding

The online tool DSDecode (http://dsdecode.scgene.com/)^46^ was used for chromatogram decoding. Sequence files (xxx.abi) and the reference gene sequences were uploaded to the server where they were decoded. Subsequently, the results were compared to the reference sequence to ensure that the cleavage site is in the target region of sgRNA.

## Electronic supplementary material

Supplementary Information
