# Author Correction: CRISPR/Cas9-mediated genome editing efficiently creates specific mutations at multiple loci using one sgRNA in *Brassica napus*

**DOI:** 10.1038/s41598-018-23161-4

**Published:** 2018-03-15

**Authors:** Hong Yang, Jia-Jing Wu, Ting Tang, Ke-De Liu, Cheng Dai

**Affiliations:** 1 College of Plant Science & Technology, Huazhong Agricultural University, Wuhan, 430070 China; 2 National Key Laboratory of Crop Genetic Improvement, Huazhong Agricultural University, Wuhan, 430070 China

Correction to: *Scientific Reports* 10.1038/s41598-017-07871-9, published online 08 August 2017

The Acknowledgements section in this Article is incomplete.

“This study was supported by the National Key Research and Development Program of China (2016YFD0101007) to Dr. Ke-de Liu, the Natural Science Foundation of Hubei Province (36116006), and the Scientific & Technological Self-innovation Foundation of Huazhong Agricultural University (0900206305) to Dr. Cheng Dai. We are grateful to Dr. Qijun Chen for kindly providing pKSE401 and pCBC-DT1T2. We thank Dr. Chunying Kang, Dr. Robert M. Larkin and Dr. Liang Guo (Huazhong Agricultural University, China) for helpful discussion and critical reading of the manuscript.”

should read:

“This study was supported by the National Key Research and Development Program of China (2016YFD0101007) to Dr. Ke-de Liu, the Fundamental Research Funds for the Central Universities (No. 2662015BQ031) and the Natural Science Foundation of Hubei Province (No. 2016CFB141) to Dr. Cheng Dai. We are grateful to Dr. Qijun Chen for kindly providing pKSE401 and pCBC-DT1T2. We thank Dr. Chunying Kang, Dr. Robert M. Larkin and Dr. Liang Guo (Huazhong Agricultural University, China) for helpful discussion and critical reading of the manuscript.”

