# Supplementary material for: CRISPR/Cas9-mediated genome editing efficiently creates specific mutations at multiple loci using one sgRNA in Brassica napus
Source: Sci Rep. 2017 Aug 8;7:7489. doi: 10.1038/s41598-017-07871-9 (PMC5548805; doi:10.1038/s41598-017-07871-9)
Supplement: Supplementary file 1 — Supplementary Information [file 41598_2017_7871_MOESM1_ESM.pdf]

Title page

CRISPR/Cas9-mediated genome editing efficiently creates specific mutations at multiple loci using one sgRNA in *Brassica napus*

Hong Yang[1], Jia-Jing Wu[1], Ting Tang[1], Ke-De Liu[2]*, Cheng Dai[1]*

1. College of Plant Science & Technology, Huazhong Agricultural University, Wuhan 430070, China

2. National Key Laboratory of Crop Genetic Improvement, Huazhong Agricultural University, Wuhan 430070, China

Supplemental Information

Table of contents

Supplemental figure legends.

Figure S1. Vector design for CRISPR/Cas9 mediated genome editing and plant transformation workflow in *B. napus*.

Figure S2. The genomic sequence alignment and sgRNA locations of *BnaRGA* family genes

Figure S3. The genomic sequence alignment and sgRNA locations of *BnaDA1* family genes.

Figure S4. The genomic sequence alignment and sgRNA locations of *BnaDA2* family genes

Figure S5. The genomic sequence alignment and sgRNA locations of *BnaFUL* family genes

Figure S6. Detection of target mutations by the T7 endonuclease I (T7E1) assay.

Figure S7. Frequency of cleavage sites upstream of the PAM sequence.

Figure S8. Frequency of each mutation type for bi-allelic mutations.

Figure S9. Gel images showing the PCR products of *Cas9* obtained from *BnaC5.DA1-sgRNA-L16* in the T0 and T1 generations.

Figure S10. Phenotypes of *BnaA6.RGA-sgRNA* transgenic plants in the T1 generation.

Figure S11. Stem length of the *BnaRGA-sgRNA* quadruple mutant plants in the T1 generation.

Figure S12. Image showing the PCR products from *Cas9*, *NPTII* and *pKSE401* derived from different progeny of BnaRGA-L44 in the T1 generation.

Figure S13. The stem length of *BnaA6.RGA-sgRNA* and *BnaA9.RGA-sgRNA* transgenic plants in the T1 generation.

Supplemental Table S1. Selected target genes in *B. napus* for assessing CRISPR/Cas9.

Supplemental Table S2. Summary of GC content and mutation rates for each sgRNA.

Supplemental Table S3. Sequencing results from the T0 and T1 plants.

Supplemental Table S4. Dissection of the genotypes in leaves and mixed tissues (leaf, stem, and flower bud) in the T0 generation.

Supplemental Table S5. Percentage of T1 plants with or without the Cas9 transgene.

Supplemental Table S6. The list of primers used in this study.

Supplemental Data S1. Decoding results from the T0 plants.

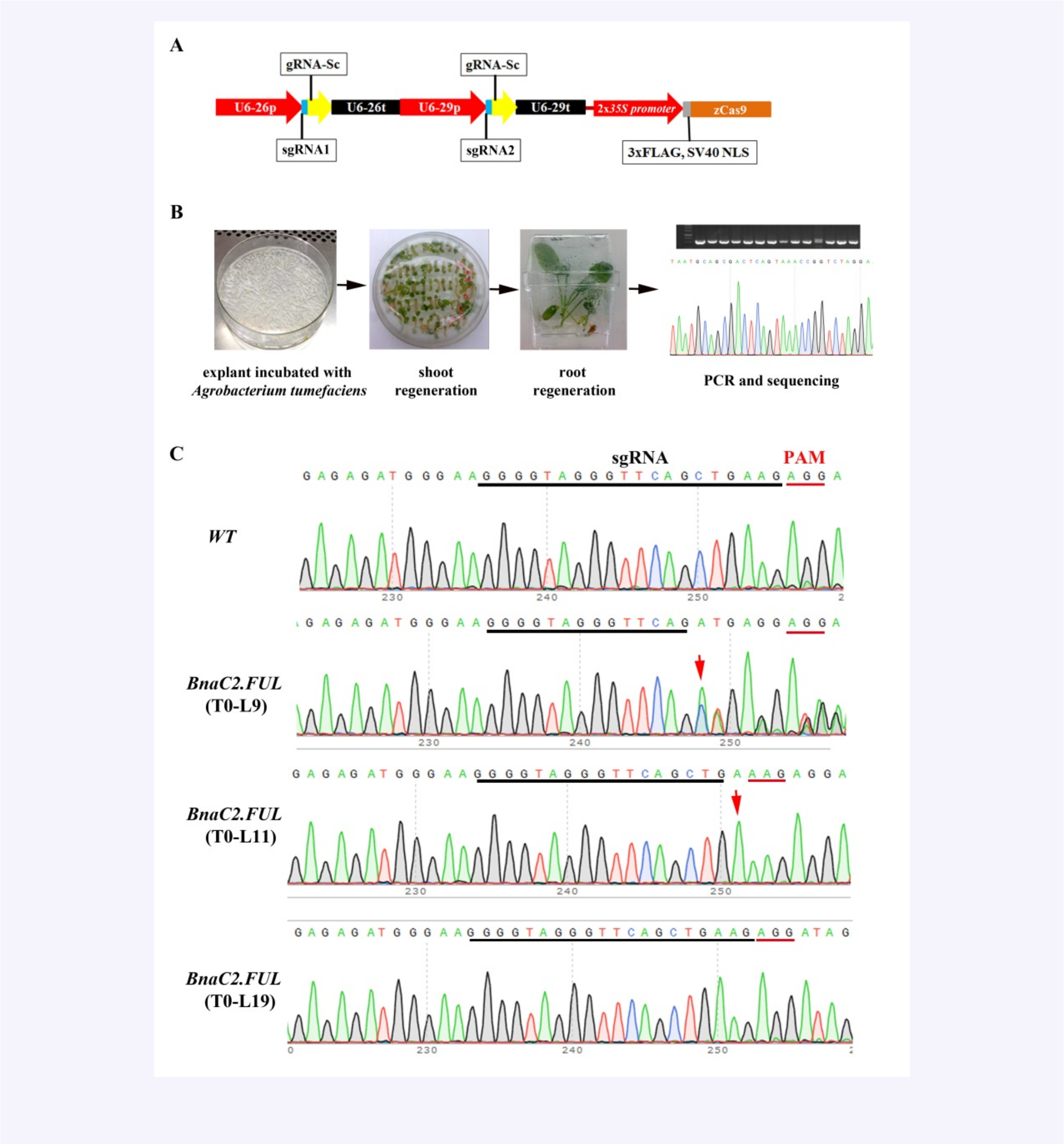

Figure S1. Vector design for CRISPR/Cas9 mediated genome editing and workflow of plant transformation in *B. napus*.

(A) Diagram of the Cas9/gRNA constructs[33]. The expression of the full-length sequence of *Zea mays* codon-optimized Cas9 protein is driven by the 2x*35S* promoter with a 3xFLAG tag and the SV40 nuclear localization signal (NLS) at the N terminus. *U6-26p* and *U6-29p* are two *Arabidopsis U6* gene promoters that drive the expression of the two sgRNAs. U6-26t and U6-29t are the *U6* gene terminators from *Arabidopsis*. gRNA-Sc: gRNA scaffold.

(B) Workflow of *B. napus* transformation. The diagram illustrates the procedure from

the *Agrobacterium tumefaciens* infection of the explant tissues to the regeneration of the plant. The DNA was extracted from Cas9-positive lines for PCR and sequencing. (C) Examples of sequencing chromatograms from WT and the *BnaC2.FUL-sgRNA* (T0) transgenic plants L9, L11, and L19. The arrowhead indicates overlapping peaks (L9) or an "A" insertion (L11). No mutation was discovered for L19. The black and red lines indicate the sgRNA and PAM sequence, respectively.

Figure S2: The genomic sequence alignment and sgRNA locations for the *BnaRGA* family genes. The gene specific sgRNA targets are indicated with red. The paralogous gene specific sgRNA targets are indictaed with light blue. The PAM motif (NGG) is indicated with a grey box. The start codons are indicated with a yellow box, and the stop codons are indicated with a blue box.

```
BnaA9.RGA      -------GAACTAAATCAGAGACATGAAGAGAGATCTCCATCAGTTTCAAGGTCCTCCTGATACTAGATTCCCCAACCAC---GGGACAG
BnaC9.RGA      -------GAACTAAATCAGAGACATGAAGAGAGATCTCCATCAGTTTCAAGGTCCTCCTGATACTAGATTCCCCAACCAC---GGGACAG
BnaA6.RGA      ACTATATCTTAACCAGATCAGAAATGAAGAGGGATCTTCATCAGTTCCAAGGTCCCAACCACGGGACATCAATCGCCGGTTCTTCCACTT
BnaC7.RGA      ACTA---TCTAACCAGATCAGAAATGAAGAGAGATCTTCATCAGTTCCAAGGTCCCAACCACGGGACATCAATCGCCGGTTCTTCCACTT
                             *        ******** ***** ******** ********     *    * **    *  *         **

BnaA9.RGA      CGAACACCGGTTCCTCTTCTAAAGACAAGATGATGATGGTCAAGGAAGAAGAAGACGGCGGAAACATGGATGAGCTTCTCGCTGTGTTGG
BnaC9.RGA      CGAACACCGGTTCCTCTTCTAAAGACAAGATGATGAAGGTTAAGGAAGAAGAAGACGGCGGAAACATGGATGAGCTTCTCGCTGTGTTGG
BnaA6.RGA      CTTCCCCTGCGGTGTTTGGTAAAGACAAGATGATGATGGTCAAAGA------------AGAAGAAGACGACGAGCTTCTAGGAGTCTTGG
BnaC7.RGA      CTTCCCCGGCGGTTTTTGGTAAAGACAAGATGATGATGGTTAAGGA------------AGAAGAAGACGACGAGCTTCTAGGAGTCTTGG
               *   * * *     * *  ***************** *** ** **             * * *    ** ******** *  ** ****

BnaA9.RGA      GTTACAAGGTGAGGTCGTCCGAGATGGCCGAGGTTGCGTTGAAGCTAGAGCAGTTAGAGACCATGATGGGTAACGTTCAAGAAGACGGTT
BnaC9.RGA      GTTACAAGGTGAGGTCGTCAGAGATGGCGGAGGTTGCGTTGAAGCTAGAGCAGTTAGAGACCATGATGGGTAACGTTCAAGAAGATGGTT
BnaA6.RGA      GTTACAAGGTTAGGTCTTCGGAGATGGCTGAGGTTGCGTTGAAACTCGAGCAGCTTGAGACGATGATGGGTAACGCTCAAGAAGACGGTT
BnaC7.RGA      GTTACAAGGTCAGGTCGTCGGAGATGGCTGAGGTTGCGTTGAAACTCGAGCAGCTTGAGACAATGATGGGTAATGTTCAAGAAGACGGTT
               ********** ***** ** ******** ************** ** ****** * ***** *********** * ********* ****

BnaA9.RGA      TATCCAATCTCGCGACGGATACTGTTCACTACAACCCGTCGGAGCTCTACTCGTGGCTTGATAACATGCTCACGGAGTTTAACCCGCCGC
BnaC9.RGA      TATCTAATCTCGCGACGGATACTGTTCACTACAACCCGTCGGAGCTTTACTCGTGGCTTGATAACATGCTCACGGAGTTTAACCCGCCGC
BnaA6.RGA      TAGCTCACCTCGCGACGGATACTGTTCATTACAACCCCGCTGAGCTTTACTCGTGGCTTGATAACATGCTCACGGAGCTTAACCCACCCG
BnaC7.RGA      TAGCTCATCTCGCGACGGATACTGTTCATTACAACCCTGCTGAGCTCTACTCTTGGCTTGATAACATGCTCACGGAGCTTAACCCACCCG
               ** *  * ******************** ********  * ***** ***** ************************ ******* **

BnaA9.RGA      CGCCGGAGATAAACAACTCGTTTCTCGCCGGAGCTG------------------------------------GTGGTTCCGATTACGACC
BnaC9.RGA      CGCCGGAGATAAACAACTCGTTTCTCGCCGGAGCTG------------------------------------GTGGTTCCGATTACGACC
BnaA6.RGA      CTGCAACGACCGGATCTAACGCTTTGAACC---CGGAGATTAATAATAATAATAATAACTCGTTTTTCAC---------CGGAGGCGACC
BnaC7.RGA      CTGCGTCCGGGTCTAGTGGTGTGAATCAGATTCTTCCTTCCCCGGAGGTTAATAATAACTCGTTCTTCGCCGGAGGCGGCGATTACGACC
               *  *                                                                           **    *****
```

```
BnaA9.RGA   TCAAAGCCATTCCTGGAAACGCGATTTACGCGAGATCCGACCAGTTCGCGATAGATTCTTCCTCCTCGTCGAACCAAGCTGGTGATAACA
BnaC9.RGA   TCAAAGCCATTCCTGGAAACGCGATTTACGCGAGATCCAACCAGTTCGCGATAGATTCTTCCTCCTCGTCGAACCAAGCTGGTGATAACA
BnaA6.RGA   TCAAAGCGATTCCTGGAAACGCGGTTTGTCGCAGATCTAATCAGTTCGCGTTTGCG---------------------------GTTGATT
BnaC7.RGA   TCAAAGCTATTCCCGGAAGCGCGATTTGCAGGGGATCTAATCAGT------TCGCC---------------------------GTCGATT
            ******* ***** **** **** ***      ****  * ****      * *                             *   *

BnaA9.RGA   GTCAGAGCACCAAGCGTCTTAAGTCATGCTCGAGCCCTGATTCGTTGGTTACAGGAACAACGGTAACAACCACAACCACTGAGTCAACTC
BnaC9.RGA   GCCAGAGCACCAAGCGTTTGAAGTCATGCTCGAGCCCTGATTCGTTGGTTACAGGAACAACGGTAACAACCACAACTACTGAGTCA----
BnaA6.RGA   CGTCGAGTAATAAGCGTTTGAAACCGTCCTCGAGCCCTGATTCGATGGTTACATCTCCATCACCTGCTGG---AGTTATAGGAACGACGG
BnaC7.RGA   CGTTGAATAATAAACGGTTGAAACCATGCTCGAGCCCTGATTCGATGGTTACATCTCCATCACCTGCTGG---AGTTATAGGAACGACGG
                **  *  ** **  * **  * * **************** ********    ** *     *      *   *  *   *

BnaA9.RGA   GGTCTGTAGGCCTCGCAGCTGAGTCAACTCGGTCCATGGTCCTGGTGGACTCGCAAGAGAACGGTGTGCGTCTGGTCCACGCGCTCATGG
BnaC9.RGA   --------------------------ACTCGGTCCATGGTCCTGGTGGACTCGCAAGAGAACGGTGTGCGCCTAGTCCACGCGCTCATGG
BnaA6.RGA   TTACAA------CCGTGACCGAGTCAACTCGTCCTTTAATCCTGGTCGACTCGCAGGACAACGGAGTGCGTCTAGTCCACGCGCTTATGG
BnaC7.RGA   TTACCACAACAACCGCGAGCGAGTCAACTCGTCCTTTAATCCTGGTCGACTCGCAGGACAACGGCGTGCGTCTAGTCCACGCGCTTATGG
                                      *****  *  *  ******* ******** ** ***** ***** ** *********** ****

BnaA9.RGA   CCTGCGCGGAAGCAATCCAGAACAACGATTTGTCCATAGCGGAAGCTCTGGTGAAGCAGATTGGATTCTTGGCCGTGTCTCAAGCCGGAG
BnaC9.RGA   CCTGCGCGGAAGCTATCCAGAACAACGATTTGTCTATAGCGGAAGCTCTCGTCAAGCAGATTGGATTCTTAGCCGTGTCTCAAGCCGGAG
BnaA6.RGA   CCTGCGCTGAAGCCGTGCAGAGCAGCAACTTGACTCTAGCGGAGGCTCTCGTTAAGCAGATTGGTTTCTTGGCCGTCTCTCAAGCCGGAG
BnaC7.RGA   CCTGTGCGGAAGCCGTGCAGAGCAGCAACTTGACTCTAGCGGAGGCTCTCGTTAAGCAGATTGGGTTCTTGGCCGTGTCTCAAGCCGGAG
            **** ** *****  * **** ** * * *** *  ******* ***** ** *********** ***** ***** *************

BnaA9.RGA   CCATGAGGAAAGTGGCGACGTACTTCGCCGAAGCCCTCGCGCGGCGGATCTACCGCCTCTCTCCGCCGCAGACTCAGATCGATCACTCTC
BnaC9.RGA   CCATGAGGAAAGTGGCGACGTACTTCGCCGAAGCTCTCGCGCGGCGGATCTACCGCCTCTCTCCGCCGCAGACTCAGATCGATCACTCTC
BnaA6.RGA   CCATGAGGAAAGTCGCCACGTACTTCGCCGAAGCTCTCGCGCGGAGGATCTACCGCCTCTCTCCGCCGCAGACGCAGATCGATCACTCTT
BnaC7.RGA   CCATGAGGAAGGTCGCCACGTACTTCGCCGAAGCTCTCGCGCGGCGGATCTACCGCCTCTCTCCGCCGCAGACTCAGATTGACCATTCTC
            ********** ** ** ***************** ********* **************************** ***** ** ** ***

BnaA9.RGA   TCTCCGACACGCTCCAGATGCACTTCTACGAGACGTGCCCTTACCTCAAGTTCGCTCACTTCACGGCCAACCAAGCCATCCTCGAGGCTT
BnaC9.RGA   TCTCGGACACGCTCCAGATGCACTTCTACGAGACGTGCCCTTACCTCAAGTTCGCTCACTTCACGGCCAACCAAGCCATCCTCGAGGCTT
BnaA6.RGA   TATCCGATACTCTCCAGATGCACTTCTACGAGACTTGCCCTTACCTCAAGTTCGCTCACTTCACGGCGAATCAGGCGATTCTCGAGGCTT
BnaC7.RGA   TCTCCGATACTCTCCAGATGCACTTCTACGAGACGTGCCCTTACCTCAAGTTCGCTCACTTCACGGCGAATCAAGCCATCCTCGAGGCTT
            * ** ** ** *********************** ******************************** ** ** ** ** **********
```

```
BnaA9.RGA   TCGAAGGGAAGAAGAGAGTCCACGTCATCGACTTCTCCATGAATCAAGGCCTGCAATGGCCGGCGCTTATGCAAGCCCTTGCGTTGAGAG
BnaC9.RGA   TCGAAGGGAAGAAGAGAGTCCACGTCATCGACTTCTCCATGAATCAAGGCCTGCAATGGCCGGCGCTTATGCAAGCCCTTGCGTTGAGAG
BnaA6.RGA   TCGAAGGGAAGAAGAGAGTCCACGTCATCGATTTCTCGATGAACCAAGGGCTTCAGTGGCCCGCGCTTATGCAAGCCCTTGCGTTGAGGG
BnaC7.RGA   TCGAAGGGAAGAAGAGAGTCCACGTCATCGATTTCTCGATGAACCAAGGGCTTCAGTGGCCCGCGCTTATGCAAGCCCTCGCGTTGAGAG
            ******************************* ***** ***** ***** ** ** ***** ***************** ******** *

BnaA9.RGA   AAGGTGGTCCTCCTGTTTTCCGGTTAACCGGGATTGGTCCTCCGGCGGCGGATAACTCCGACCATCTCCATGAGGTTGGGTGTAAGTTAG
BnaC9.RGA   AAGGTGGTCCTCCTGTTTTCCGGTTAACCGGGATTGGTCCTCCGGCGGCGGATAACTCCGATCATCTCCATGAGGTTGGGTGTAAGTTAG
BnaA6.RGA   AAGGAGGTCCTCCGAGTTTCAGGTTAACCGGAATTGGTCCTCCCGCGGCGGATAACTCCGATCATCTCCATGAAGTTGGATGTAAGTTGG
BnaC7.RGA   AAGGAGGTCCTCCGAGTTTCAGGTTAACCGGAATTGGTCCTCCCGCGGCGGATAACTCCGATCATCTCCACGAAGTTGGATGTAAGTTGG
            **** ********   **** ********** *********** ***************** ******** ** ***** ******** *

BnaA9.RGA   CTCAGCTCGCGGAAGCGATTCACGTGGAGTTTGAGTATCGTGGCTTCGTGGCTAATAGCTTGGCCGATCTCGATGCTTCGATGCTTGAGC
BnaC9.RGA   CTCAGCTCGCGGAAGCGATTCACGTCGAGTTTGAGTATCGTGGCTTCGTGGCTAATAGCTTGGCCGATCTCGATGCTTCCATGCTTGAGC
BnaA6.RGA   CTCAGCTCGCGGAGGCGATTCACGTCGAGTTTGAGTATCGTGGCTTTGTTGCTAATAGCTTAGCTGATCTTGATGCCTCGATGCTTGAGC
BnaC7.RGA   CTCAGCTCGCAGAGGCGATTCACGTCGAGTTTGAGTACCGTGGCTTTGTTGCTAACAGCTTAGCCGATCTCGACGCGTCGATGCTTGAGC
            ********** ** *********** *********** ******** ** ***** ***** ** ***** ** ** ** **********

BnaA9.RGA   TTAGACCGAGTGAGATCGAAGCTGTGGCGGTTAACTCTGTTTTCGAGCTACACAAGCTCCTTGGCCGTACCGGTGGGATAGAGAAAGTTC
BnaC9.RGA   TTAGACCGAGTGAGATCGAAGCTGTGGCGGTTAACTCTGTTTTCGAGCTACACAAGCTCCTTGGCCGTACCGGTGGGATAGAGAAAGTTC
BnaA6.RGA   TTAGACCGAGTGAAACCGAAGCTGTGGCGGTTAACTCTGTTTTCGAGCTCCACAAGCTCCTAGGCCGTACCGGTGGGATAGAGAAAGTCT
BnaC7.RGA   TTAGACCGAGCGAAACCGAATCTGTGGCGGTTAACTCCGTTTTCGAGCTCCACAAGCTCCTAGGCCGTCCCGGTGGGATAGAGAAAGTCT
            ********** ** * **** **************** *********** *********** ****** *******************

BnaA9.RGA   TCGGCGTGGTGAAACAGATTAAACCGGTGATTTTCACGGTGGTTGAGCAAGAATCGAGTCATAACGGTCCGGATTTCTTAGACCGGTTTA
BnaC9.RGA   TCGGCGTGGTGAAACAGATTAAACCGGTGATTTTCACGGTGGTTGAGCAAGAATCGAATCATAACGGTCCGGTTTTCCTAGACCGGTTTA
BnaA6.RGA   TCGGCGTTGTGAAACAGATTAAACCGGTGATTTTCACGGTTGTTGAGCAAGAATCGAATCATAACGGTCCGGTTTTCTTAGACCGGTTTA
BnaC7.RGA   TCGGCGTTGTGAAACAGATTAAACCGGTGATTTTCACGGTGGTTGAGCAAGAATCGAATCATAACGGTCCGGTTTTCTTAGACCGGTTTA
            ******* ******************************** **************** ************** **** ************

BnaA9.RGA   CTGAATCGCTGCATTACTATTCGACGTTGTTTGATTCCTTGGAAGGTGTTCCCAGTAGCCAAGACAAGGTTATGTCGGAAGTTTACTTAG
BnaC9.RGA   CTGAGTCGCTGCATTACTATTCGACACTGTTTGATTCTTTGGAAGGTGTTCCCAGTAGCCAAGACAAAGTCATGTCGGAAGTTTACTTAG
BnaA6.RGA   CTGAATCGCTGCATTATTATTCGACGTTGTTTGATTCCTTGGAAGGTGCTCCGAGTAGCCAAGATAAAGTTATGTCGGAAGTTTATTTAG
BnaC7.RGA   CTGAATCGCTGCATTACTATTCGACCTTGTTTGATTCCTTGGAAGGAGCTCCGAGTAGCCAAGACAAAGTCATGTCGGAAGTTTACTTAG
            **** *********** ********  ********** ******** * *** *********** ** ** ************** ****
```

```
BnaA9.RGA       GGAAACAGATTTGCAATCTCGTGGCTTGTGAAGGACCAGACCGGGTGGAGAGACACGAGACGCTGAGTCAATGGGCTAACCGGTTCGGTA
BnaC9.RGA       GGAAACAGATTTGCAATCTCGTGGCTTGTGAAGGACCAGACCGGGTGGAGAGACACGAGACGCTGAGTCAATGGGCTAACCGGTTCGGTA
BnaA6.RGA       GGAAACAGATTTGCAATCTGGTGGCTTGCGAAGGTCCGGACCGTGTTGAGAGACATGAGACGCTGAGTCAATGGTCGAACCGGTTCGGTT
BnaC7.RGA       GGAAACAGATTTGCAATCTGGTGGCTTGCGAAGGTCCGGACCGTGTCGAGAGACATGAGACGCTGAGTCAATGGTCGAACCGGTTCGGTT
                ******************* ******** ***** ** ***** ** ******** ****************** * ************

BnaA9.RGA       CGTCCGGTTTTGCGCCGGCACATCTCGGGTCTAACGCGTTTAAGCAAGCGAGTATGCTTTTGGCTTTGTTTAACGGCGGCGAAGGTTATC
BnaC9.RGA       CGTCCGGTTTTGCGCCGGCACATCTCGGGTCTAACGCGTTTAAGCAAGCGAGTATGCTTTTGGCTTTGTTTAACGGCGGCGAAGGTTATC
BnaA6.RGA       CGTCCGGTTTTGCGCCGGCGCATCTCGGGTCTAACGCGTTTAAGCAAGCGAGTACGCTTTTGGCTTTGTTTAATGGAGGCGAAGGTTATC
BnaC7.RGA       CGTCCGGTTTTGCGCCGGCGCATCTCGGGTCTAACGCGTTTAAGCAAGCGAGTACGCTTTTGGCTTTGTTTAATGGAGGCGAAGGTTATC
                ******************* ********************************** ****************** ** *************

BnaA9.RGA       GTGTGGAGGAGAATAATGGGTGCTTGATGTTGGGTTGGCATACTCGGCCGCTGATAACAACCTCCGCTTGGAAGCTCTCGGCGGCGCACT
BnaC9.RGA       TTGTGGAGGAGAATAATGGGTGTTTGATGTTGGGTTGGCATACTCGACCTCTGGTAACAACCTCCGCTTGGAAGCTATTGGCGGCGCACT
BnaA6.RGA       GTGTGGAGAAGAATAATGGGTGTTTGATGTTGAGTTGGCACACTCGACCGCTCATAACCACCTCCGCTTGGAAGCTCTCGGCGGTGCACT
BnaC7.RGA       GTGTGGAGGAGAATAATGGGTGTTTGATGTTGAGCTGGCACACACGACCGCTCATAACAACCTCCGCTTGGAAGCTCTCGGCGGTGCACT
                 ******* ************* ********* * ***** ** ** ** **  **** ***************** * ***** *****

BnaA9.RGA       GAGTGGACACGGAGACTAGGTTGGTGGTGGTGTGGTGGTGGGTTGAG-------------------------------------------
BnaC9.RGA       GAGTGGACACGGAGACTAGGTTGGTGGCGGTAT---GGTGGGTTGAC-------------------------------------------
BnaA6.RGA       GAGTTGGCTCGATGTCGACGATGGTGGTATTGGTGATTTGACTCGAAGTCGACTGTGGTTGTGTGGTGAGTTGACTCGCAGCCGACGGTG
BnaC7.RGA       GAGTTGGCTCGATGTCGACGATGGTGGTATTGGTGATTTGAGTCGAAGTCGACTGTGGTTGTGTGGTGAGTTGACTCGCAGCCGACGGTG
                **** * * **  * * * * ******   *       **  * **
```

Figure S3: The genomic sequence alignment and sgRNA locations for the *BnaDA1* gene family. The gene specific sgRNA targets are indicated with red. The PAM motif (NGG) is indicated with a grey box. The start codons are indicated with a yellow box, and the stop codons are indicated with a blue box.

```
BnaC5.DA1    ATGAGCAGATAAGAGTTATTAGGAAGACTATGGGTTGGTTTAACAAGATCTTCAAAGGCTCTACCCAAAGGTTCCGGCTTGGGAATGACC
BnaA6.DA1    TAGAGTTATTAGGAAGAAGAGAGGTTACTATGGGTTGGTTTAACAAGATCTTCAAAGGCTCTACCCAAAGGTTCCGGCTTGGGAATGACC
               ***    ** **   *    *   ****************************************************************

BnaC5.DA1    ATGACCACAATGGCTATTACCAGAGTTATCCACATGATGAGCCTAGTGCTGATACTGATCCTGA------TCCTGATCCTGATGAAACTC
BnaA6.DA1    ATGACCACAATGGCTATTACCAGAGTTATCCACATGATGAGCCTAGTGCTGATACTGATCCTGATCCTGATCCTGATCCTGATGAAACTC
             ****************************************************************      ********************
BnaC5.DA1    ATACTCAGGAACCATCTACCTCTGAGGTTACTATAACTGTCTTTACATATCTCTGGCTGCTTGTACTGTTGCTTCAACATTTTTTTGTTT
BnaA6.DA1    ATACTCAGGAACCATCTACCTCTGAGGTTACTATAACTCTCTTTACATATCTCTGGTTT---GTACTATTGCTTCAACATTTTGTTGTTT
             ************************************** ***************** *    ***** *************** ******

BnaC5.DA1    CCCTTTACTAGGAGGATACATCCGGCCAGGAGAATGAAGACATTGACCGTGCAATCGCATTGTCTCTTATAGAAAACAGTCAAGGACATA
BnaA6.DA1    CCCTTTACTAGGAGGATACATCCGGCCAGGAAAACGAAGACATAGATCGTGCAATCGCATTGTCTCTTATAGAAAACAGTCAAGGACAGA
             ******************************* ** ******** ** ***************************************** *

BnaC5.DA1    CTAACACAG---GCGCCGGTGAGTCCTTTTTCCTTGCCAAACTAGAAAGAAATATGAATTATGAAACTCGGTTTGTTACATTTAACAGAA
BnaA6.DA1    CTAATAATACATGCGCTGGTGAGTCCCTTTTCCTTGCCAA----ACTAGAAATATGAATTATGAAACTCGGTTTGTTACATTTAAAAGAA
             **** *      **** ********* *************       ************************************** ****

BnaC5.DA1    TAGTGAACGCAGGGAAGTACGCAATGGTGGATGAAGATGAGCAGCTTGCTAGAGCCATACAAGAGAGCATGGTAGTTGGGAATACACCGC
BnaA6.DA1    TAGCCAACGCAGGGAAGTACGCAATGGTGGATGAAGATGAGCAACTTGCTAGAGCCATACAAGAGAGCATGGTAGTTGGGAATACACCGC
             ***  ************************************** **********************************************

BnaC5.DA1    GTCAGAAGCATGGAAGCAGTTATGATATTGGGAACGCATATGGGTCTGGAGACGTATACGGGAATGGACATATGCATGGAGGTGGAAATG
BnaA6.DA1    GTCAGAAGCATGGAAGTAGTTATGATATTGGGAATGCATATGGGGCTGGAGACGTTTACGGGAATGGACATATGCATGGAGGTGGAAATG
             **************** ***************** ********* ********** **********************************
```

```
BnaC5.DA1    TTTATGCCAATGGAGACATTTATTATCCAAGACCTACTGCTTTTCCTATGGATTTCAGGTTCACTTTTGATACTCAATTAATCATCTGTA
BnaA6.DA1    TATATGCCAATGGAGATATTTATTATCCAAGACCTACTGCTTTCCCAATGGATTTCAGGTTCACTTT-GATACTCAATCAATCATCTGTA
             * ************** ************************** ** ******************** ********** ***********

BnaC5.DA1    GCCTGTTTAACTTGGTTGAGATGTGTTAAATAACTT-------ATCAAGAACACCTTAGGATTTGTGCTGGCTGCAATATGGAGATTGGG
BnaA6.DA1    GCCTGTTTGTTAAGTTTCTTTCCAGTTAAGTAACTCACCAACAACGTGTCACTACCTAGGATTTGTGCTGGCTGCAATATGGAGATTGGA
             ********     * **       ***** *****        *      **  * *********************************

BnaC5.DA1    CATGGAAGATATCTGAATTGCTTGAATGCACTGTGGCATCCGGAATGTTTTCGATGCTATGGCTGTAGGCACCCCATTTCTGAGTACGAG
BnaA6.DA1    CATGGAAGATATCTGAATTGCTTGAATGCACTATGGCATCCAGAATGTTTTCGATGTTATGGCTGTAGGCATCCCATCTCTGAGTACGAG
             ******************************** ******** ************** ************** ***** ************

BnaC5.DA1    GTGAAATCAAGCTTTCTCATTCTTTCTATTGTAGTTAACCTTTGATGTAATGAATAACATGTTTTCCTTTTTTTTCTTAATAGTTCTCAA
BnaA6.DA1    GTGAAGTCAAGCTTTCTTATTCTTTTGATTGTAGATAACCTTCAAAATTAAC--------GCATAACATGTTTTCCTTTATAGTTCTCAA
             ***** *********** *******  ******* *******  *  * *          *  *  * * **** *** ***********

BnaC5.DA1    CATCTGGGAACTACCCTTTTCACAAAGCTTGTTATAGGGAGAGATACCATCCAAAATGTGATGTCTGCAGCCTCTTTGTATGTAAATCTT
BnaA6.DA1    CGTCTGGGAACTACCCTTTTCACAAAGCTTGTTATAGGGAGAGATACCATCCAAAATGTGATGTCTGCAGCCTCTTTGTATGTAAATCTT
             * ****************************************************************************************

BnaC5.DA1    TAGTCTTTTTTTTCCATCATTAAAGTGCCTATTTATGCGTTGTTTCCTTCGGATAGTGCACTTATTATATCTTCTGTTGATTTTCTTGTT
BnaA6.DA1    TAGCCTTTTTTT--CATTTTTAAAGTGCCTATATATGCCTTGTTTCCTTCGGATATTGCACTTATCTTCTGTTGAT--------------
             *** ********  ***  ************* ***** **************** *********  * * **

BnaC5.DA1    TTCGAATGTAAATTTGTTGTGACAGATTCCAACAAACCATGCTGGTCTTATTGAATATAGGGCACATCCTTTTTGGGTCCAGAAGTACTG
BnaA6.DA1    TTTCTTGTTTCGAATGTTGTGACAGATTCCAACAAACCATGCTGGTCTTATTGAATATAGGGCACATCCTTTTTGGGTCCAGAAGTATTG
             **      *     ************************************************************************* **
BnaC5.DA1    CCCTTCTCACGAACACGATGCTACCCCAAGATGTTGCAGTTGCGAAAGAATGGAGGTGAGTTTTTCTCCCCAAAATGTGTCCCACAACAA
BnaA6.DA1    CCCTTCTCACGAACACGATGCTACCCCAAGATGTTGCAGTTGCGAAAGAATGGAGGTTGAGTTTTTCTCCCCCTAAGTGTCCTACAACAA
             *********************************************************    ****   ***   * ****** *******

BnaC5.DA1    ACATCTGCTCAAAGTCGAAATGTCAACTTTCGTTTTTTTTTTCTTTGTAGCCACGGAATACAGGATATGTTGAACTTAACGATGGACGGA
BnaA6.DA1    ACATCTTCCCAAAGTCAATACTA-ATTTGCCAGCTTTCGTTTATGTGCAGCCACGCAATACAGGATATGTTGAACTTAACGATGGACGGA
             ****** * ******* * *    *  *  *   ***  *** * ** ******* **********************************
```

```
BnaC5.DA1    AACTTTGCCTGGAGTGTCTGGACTCAGCAGTCATGGACACTTTTCAATGCCAACCTCTGTATCTGCAGATACAAGAATTCTATGAAGGGC
BnaA6.DA1    AACTTTGCCTTGAATGTCTGGACTCAGCGGTGATGGACACTTTTCAATGCCAACCTCTGTATCTGCAGATACAAGAATTCTACGAAGGTC
             ********** ** ************** ** ************************************************** ***** *

BnaC5.DA1    TTTTCATGAAGGTAGAGCAGGACGTTCCACTTCTTTTAGTTGAGAGGCAAGCACTCAACGAAGCCAGAGAAGGTGAAAAGAATGTGAGTA
BnaA6.DA1    TTTTCATGAAGGTTGAGCAGGACGTTCCACTTCTTTTAGTTGAGAGGCAAGCACTCAACGAAGCCAGAGAAGGTGAAAAGAATGTGAGTA
             ************* ****************************************************************************

BnaC5.DA1    GCAAAAAGAAAAACACAATTATACTTCAGTACATATATCACCTTTTTTCA--------CAAGTGTTTTAAGCTTTTCATTGTGTGCTTCC
BnaA6.DA1    AACAACATACAGTTGTCAAGTAATTTATTTGAATATATCACATTTATTTTTGTTTTATCCGAAGTGTTTTAACTTTTGGTTGTGTTCTTC
                ** * * *      *    * **   *  ********* *** **          *    ** **     ***   *       * *

BnaC5.DA1    TTAGGGTCACTATCACATGCCAGAGACGAGAGGACTCTGCCTTTCAGAAGAACAAACTGTTAGCACTGTGAGAAAGAGATCGAAGCATGG
BnaA6.DA1    TTAGGGTCACTATCACATGCCAGAGACAAGAGGACTCTGCCTTTCAGAAGAACAAACTGTTAGCACTGTAAGAAAGAGATCGAAGCATGG
             *************************** ***************************************** ********************
BnaC5.DA1    CACAGGAAACTGGGCTGGGAATATGATTACAGAGCCTTACAAGTTAACACGTCAATGCGAGGTTACTGCCATTCTCATCTTGTTTGGGCT
BnaA6.DA1    CACAGGAAACTGGGCTGGGAATATGATTACAGAGCCTTACAAGTTAACACGTCAATGCGAGGTCACTGCCATTCTCATCTTGTTTGGGCT
             *************************************************************** **************************

BnaC5.DA1    CCCTAGGCTACTCACCGGTTCGATTCTAGCTCATGAGATGATGCACGCGTGGATGCGGCTCAAGGGTGAGTTTCTTAGTTCACTGCTTCT
BnaA6.DA1    CCCTAGGCTACTCACCGGTTCGATTCTAGCTCATGAGATGATGCACGCGTGGATGCGGCTCAAGGGTGAGTTTCTTAGTTCACTGCTTCT
             ******************************************************************************************

BnaC5.DA1    CTTTT-TTTCACATTGTTGAATCTCTATTGTGGTCTTGAAAAGGATTCCGGACGCTGAGCCAAGACGTTGAAGAAGGAATATGTCAAGTA
BnaA6.DA1    CTTTTTTTTCACATTGTTGAATCTCTATTGTGGTCTTGAAAAGGATTCCGGACGCTGAGCCAAGACGTTGAAGAAGGAATATGTCAAGTG
             ***** ***********************************************************************************

BnaC5.DA1    ATGGCTCATAAGTGGTTGGAAGCAGAGTTAGCTGCTGGTTCAAGAAACAGCAATGTTGCATCATCATCATCTTCTTCTTCTGGAGGATTG
BnaA6.DA1    ATGGCTCATAAGTGGTTGGAAGCAGAGTTAGCTGCTGGTTCAAGAAACAGCAATGTTGCGTCATCTTC------ATCTTCTAGAGGAGTG
             *********************************************************** ***** **       ****** ***** **

BnaC5.DA1    AAGAAGGGACCAAGATCGCAATACGAGAGGAAGCTTGGTGAGTTTTTCAAGCACCAAATCGAGTCTGATGCTTCTCCGGTTTATGGAGAC
BnaA6.DA1    AAGAAGGGACCAAGATCGCAGTACGAGAGGAAGCTTGGTGAGTTTTTCAAGCACCAAATCGAGTCTGATGCTTCTCCGGTTTATGGAGAC
             ******************** *********************************************************************
```

```
BnaC5.DA1   GGGTTCAGGGCTGGGAGGTTAGCGGTTAACAAGTATGGTTTGCCGAAAACACTTGAGCATATACATATGACCGGTAGATTCCCGGTTTAA
BnaA6.DA1   GGGTTCAGGGCTGGGAGGTTAGCGGTTAACAAGTATGGTTTGCCAAAAACACTTGAGCATATACAGATGACCGGTAGATTCCCGGTTTAA
            ******************************************** ******************** ************************
```

Figure S4: Genomic sequence alignment and sgRNA locations for the *BnaDA2* gene family. The gene specific sgRNA targets are indicated with red. The paralogous gene specific sgRNA targets are indicated with light blue. The PAM motif (NGG) is indicated with a grey box. The start codons are indicated with a yellow box. The stop codons are indicated with a blue box.

```
BnaC6.DA2     TGTTTTTATAGTCTCTGCCCTTAAAGTAAGTAGGAGAAAAGATTAGAACTTTTGGTGAGAGAGAGATGGGTAATAAGCTGGTGGGGAGGA
BnaA2.DA2.1   -----------------------------------------------------------------ATGGGTAATAAGCTAGTGGGAAGGA
BnaA2.DA2.2   -----------------------------------------------------------------ATGGGTAATAAGCTAGTGGGAAGGA
                                                                               ************** ***** ****

BnaC6.DA2     AGAGGCAAGTGGTGGAAGAAAGGTACACAAAGCCACAAGGCTTGTATGTGAATACAGATGTCGACATTAAGAAGCTTAGGAAACTCATTG
BnaA2.DA2.1   GGAGACAAGCGGTGGAAGAAAAGTATACAAAGCCACAAGGTTTGTATCTGAACAAAGATGTCGACATTAAAAAGCTTAGGAAACTGATTC
BnaA2.DA2.2   GGAGACAAGCGGTGGAAGAAAAGTATACAAAGCCACAAGGTTTGTATCTGAACAAAGATGTCGACATTAAAAAGCTTAGGAAACTGATTC
               *** **** *********** *** ************** ****** **** * *************** ************** ***

BnaC6.DA2     TTGAGTCTAAGCTCGCTCCTTGCTACCCTGGGGACGACGAGAGCTGCCACGAGCTCGAGGAATGCCCCATTTGCTTCCTGGTTTGTGAAT
BnaA2.DA2.1   TTGAGTCTAAGCTTGCTCCTTGCTACCCTGGAGACGATGAAGGCTGTCATGAACTCGAAGAATGCCCCATTTGCTTTCTGGTTTGTGTTC
BnaA2.DA2.2   TTGAGTCTAAGCTTGCTCCTTGCTACCCTGGAGACGATGAAGGCTGTCATGAACTCGAAGAATGCCCCATTTGCTTTCTGGTTTGTGTTC
              ************* ***************** ***** **  **** ** ** ***** ***************** **********

BnaC6.DA2     TTTATCAAGTCTCTGGATTTTCTAGTGTCATTGTTGTGG-----ACCTAACTTAGTGATGTTTTTTTTTTTTTTAGTTCTATCCTAGCCT
BnaA2.DA2.1   TCTTTTTCAAGAATGCATAGCAATCTCAT-TCATTGTAGATCTAACTCTGATGTTTCTTCTTGTGTGTGTTTCTAGTACTATCCTAGCCT
BnaA2.DA2.2   TCTTTTTCAAGAATGCATAGCAATCTCAT-TCATTGTAGATCTAACTCTGATGTTTCTTCTTGTGTGTGTTTCTAGTACTATCCTAGCCT
              * * *        ** **       *    *  **** *     **     *   *  * ** * * * *** **** ************

BnaC6.DA2     CAATAGATCAAGATGTTGCATGAAAAGCATTTGTACAGGTGAAAAATTCATCAGATTTTTTTTTTAAAGCTTCTTTATGTTATTTCATGT
BnaA2.DA2.1   CAATAGGTCAAGATGTTGCATGAAAAGCATTTGTACAGGTGAAGTATATCTATCTCATCAGCTTTTGCT------TATTAGCTTCTTGTT
BnaA2.DA2.2   CAATAGGTCAAGATGTTGCATGAAAAGCATTTGTACAGGTGAAGTATATCTATCTCATCAGCTTTTGCT------TATTAGCTTCTTGTT
              ****** ************************************  **   *      *    ***          ***    **     *

BnaC6.DA2     TTTAAAATGGCGATTTGTTTTGGTTTTTGGTTGCAGAGTGTTTTTTGCAAATGAAGAATCCTAACTCAGCTCGGCCTACTCAGTATCCTT
BnaA2.DA2.1   TCCACTGTTTTGATTTGTTTTATAATTTGTTTGCAGAGTGTTTTTTGCAAATGAAGAATCCTAATTCAGCTCGGCCTACTCAGTATCCTT
BnaA2.DA2.2   TCCACTGTTTTGATTTGTTTTATAATTTGTTTGCAGAGTGTTTTTTGCAAATGAAGAATCCTAATTCAGCTCGGCCTACTCAGTATCCTT
              *  *   *   **********    **** ********************************** *************************
```

```
BnaC6.DA2       AAATTTTTTAATCCTCTGCATTTACTTGTAGGGAGTTGACTTTTATTATTAGTTTAGATGAATGGGTCTTCTAGTGCATGTGCTCCTTTA
BnaA2.DA2.1     TAAACTCAATCATGATACTCATAATCAACAAGGAGCTGGTCTAGTG-----GCTAAAACTCTTAGATTATCTAGTCGGTAT---------
BnaA2.DA2.2     TAAACTCAATCATGATACTCATAATCAACAAGGAGCTGGTCTAGTG-----GCTAAAACTCTTAGATTATCTAGTCGGTAT---------
                 **  *               * *     * **** **   *         * * * *    * * *  ******   * *

BnaC6.DA2       ACATTATGAGGTGCCCCTTTTGTAAAACACCAAACTATGCTGTTGAGTACCGTGGAGTAAAGACCAAGGAGGAAAAGGGCATGGAACAAG
BnaA2.DA2.1     ---CTAAGGAGTCATAGGTTCATGTCCCTCCAGAACCTC--------------CTAGTTAATGAAAAATAATTCATGAGCCTTTAGGGAC
BnaA2.DA2.2     ---CTAAGGAGTCATAGGTTCATGTCCCTCCAGAACCTC--------------CTAGTTAATGAAAAATAATTCATGAGCCTTTAGGGAC
                    ** *  **      **  *    * *** *   *                 *** **    **  *    * * ** *  *   *

BnaC6.DA2       TTGTAA-GATATAAATAAAT-----TATTCATTAGTAAACGTTTCTCCAATGTTTCAAGAAACGCTAGTTAGGCTCTTTAGAGAGGATTA
BnaA2.DA2.1     TTGTTTTTTTATTAGTACTTTGATGTCCTCCTTAACATTATGAGGTGCCCCTTTTGTAAAACACCCAACTATGCTGTTGAGTACCGTGGA
BnaA2.DA2.2     TTGTTTTTTTATTAGTACTTTGATGTCCTCCTTAACATTATGAGGTGCCCCTTTTGTAAAACACCCAACTATGCTGTTGAGTACCGTGGA
                ****     *** * **  *     *  ** ***  *        * *    ***  * **   * *  ** *** ** **    *   *

BnaC6.DA2       TTGATTAGGCCGGCGTCTAGACCGATTTTTTATTTTTTAAATATAGGTTAAGAAAAATCGTTTTGTTTAAACCTGATTTGCGAATGGAAT
BnaA2.DA2.1     GTAAAGAGCAAGGAGGAGAAGGGCATTGAG-----------------------CAAGTTGTA-----------AGATTTGGAAAT-----
BnaA2.DA2.2     GTAAAGAGCAAGGAGGAGAAGGGCATTGAG-----------------------CAAGTTGTA-----------AGATTTGGAAAT-----
                 * *  **   ** *   *     ***                           ** * **             ******  ***

BnaC6.DA2       GGCGCCGCCTAAACTGATTTTTAGAACATTGCGTTTCTCTATAAAGCT--------TTGGTTGAATAATATGTAATGTATAACCTTTTTT
BnaA2.DA2.1     ---------------GATCTTTCACTGTTACCTTTACTCAGTCAAGGTTGATGTAATGACTTTGTTTATATG-TAATGTTCCTTTTTTAT
BnaA2.DA2.2     ---------------GATCTTTCACTGTTACCTTTACTCAGTCAAGGTTGATGTAATGACTTTGTTTATATG-TAATGTTCCTTTTTTAT
                               *** ***      *  * ** ***  * *** *        *   **   * *****  *    *    **** *

BnaC6.DA2       GTAGGAAGAGCAAAGGGTTATAGAAGCGAAAATAAGGATGAGGCAGAAAGAAATGGAGGATGATGAAGAGAAAATGCAGAAACGTATGGA
BnaA2.DA2.1     AAAGGAGGAGCAACTGGTTATAGAAGCCAAGATAAGGATGAGGCAGAAAGAAATGCAGGATGATGAAGAGAAAATGCAGAAACGTTTGGA
BnaA2.DA2.2     AAAGGAGGAGCAACTGGTTATAGAAGCCAAGATAAGGATGAGGCAGAAAGAAATGCAGGATGATGAAGAGAAAATGCAGAAACGTTTGGA
                  **** ******  ************ ** ************************ ***************************** ****

BnaC6.DA2       ATCATCATGTTCCTCTAGCGCAAGCGCCATGACTGGTGAAATGGAATACGGTTCTGCTTCAGGTAAGGTTCTTTCTTTCTTTCAGCTAAT
BnaA2.DA2.1     ATCA---TGTTCCTCTAGCACAAGCGCAGTGACTGGTGAAATTGAATATGGCTCAGCTTCAGGTCAGTCTTTTTTTCTTACTTCAGTGTC
BnaA2.DA2.2     ATCA---TGTTCCTCTAGCACAAGCGCAGTGACTGGTGAAATTGAATATGGCTCAGCTTCAGGTCAGTCTTTTTTTCTTACTTCAGTGTC
                ****   ************ *******  ************* ***** ** ** ********* **  * *** * *   *    *
```

```
BnaC6.DA2       TCTCCTGATTAGTAACATATATGGCTTTTGACAGGCTTTCATTTTATGTGCCTTGTTGGTTTTGCAGCAATATCTTATAATGATCCCATG
BnaA2.DA2.1     AAATCTTGGTAT---CACAAA-----GGTGGTGGTCTT---CATTTTTGTTTGCCTTGGGTTTGCAGCTATGTCTTATAATTCTCCTATG
BnaA2.DA2.2     AAATCTTGGTAT---CACAAA-----GGTGGTGGTCTT---CATTTTTGTTTGCCTTGGGTTTGCAGCTATGTCTTATAATTCTCCTATG
                    **   **    ** * *       **   * ***     ** *        **** ******** ** *********  *** ***

BnaC6.DA2       GAAGACGCTGAAAGTGCTTCATCACAGAACGTGTCAGTTGCTAGACAGCGCTCCCGCCGCCCTCAAGG--AAACAGGTCTCTGTCTTTTT
BnaA2.DA2.1     GAAGATGCGGAAACTGCTTCATCTCAGAACACGTCAGTTAGACAGAGAGGAAACAGGTCTGTCTTTCTACTTGGCTGCTCTTTATTTTGT
BnaA2.DA2.2     GAAGATGCGGAAACTGCTTCATCTCAGAACACGTCAGTTAGACAGAGAGGAAACAGGTCTGTCTTTCTACTTGGCTGCTCTTTATTTTGT
                ***** ** **** ********* ******  *******          *   * *                    *    *   *** *

BnaC6.DA2       CTTTGTTAAGTTCTTCTATGGAGTAATGTATTAGTTGTAAGCATGTAGTAGTGTTATT-------------------------GCTTATT
BnaA2.DA2.1     CTTTCTCAAGTTCTGCAATGAAGTAATGTATTAGTGGTAAAGCATGATGTGTAATGAGATCATCCAGTGTTTTGGGGGAGCTAAGTACGT
BnaA2.DA2.2     CTTTCTCAAGTTCTGCAATGAAGTAATGTATTAGTGGTAAAGCATGATGTGTAATGAGATCATCCAGTGTTTTGGGGGAGCTAAGTACGT
                **** * ******* * *** ************** ****      *   **  *                              *   *

BnaC6.DA2       GGACTTGGTCT-----TGTT-TTGGGCATAGTTTTCACTTAGGGAGTGAAGTGAGTAACCTTTGTGATTTTATGTTACAAGTGGAAAGTT
BnaA2.DA2.1     AGACTTGGTTTTGTTTTGTTTTGGGGAACACTGTATCATTTAGGACTCAAGTGCACCACT-----GTGCTTCTATTACAAGTG-GATGTT
BnaA2.DA2.2     AGACTTGGTTTTGTTTTGTTTTGGGGAACACTGTATCATTTAGGACTCAAGTGCACCACT-----GTGCTTCTATTACAAGTG-GATGTT
                 ******** *     **** * *** * * * *    **  *** * *****    **      *   ** * *********  * ***

BnaC6.DA2       GTGAGAGTTTGTTATGTTTCCATGTCTCTAGCTTCAGTTTAATCTCATTTTCAGA--------------------------------CAT
BnaA2.DA2.1     TTGAGGTTAAAGTATGCTTCCATGTCTCCAACAACAATTGAATCTCATCAATGTGCATTGATGTAACTTTAATTTGAGGTGATTCAGTAT
BnaA2.DA2.2     TTGAGGTTAAAGTATGCTTCCATGTCTCCAACAACAATTGAATCTCATCAATGTGCATTGATGTAACTTTAATTTGAGGTGATTCAGTAT
                 ****  *    **** *********** * *  ** ** ********                                        **

BnaC6.DA2       CTTACTTCTGATTTGTGTGGAAACTCTTCAGGGATGATGAGGCTGAAGCTGACCTTGAAGAGTTGATGGTCATGGAAGCAATATGGCTCT
BnaA2.DA2.1     TGGACTTTATATATGTGATTTTTTTCTGAAAACTCTTCAGGGATGATGTTGACCTTGAAGAATTAATGGTTATGGAAGCAATATGGCTCT
BnaA2.DA2.2     TGGACTTTATATATGTGATTTTTTTCTGAAAACTCTTCAGGGATGATGTTGACCTTGAAGAATTAATGGTTATGGAAGCAATATGGCTCT
                   ****   ** ****       ***  *          ** *** * ************ ** ***** *******************

BnaC6.DA2       CCATGCAGGTAAAACTTTTATGTTTAGTTACTTGTCTTAGCTCTAATGACAAA--------TTTTTACATTTCTCATTGTCTTTTTTTTC
BnaA2.DA2.1     CCATTCAGGTAATAATTATTTGTTTATTTAGTTATTTTTATACCTCTTATGAAATGAAAAAAAATATCAATTC--TCCATGGCTTTTTTC
BnaA2.DA2.2     CCATTCAGGTAATAATTATTTGTTTATTTAGTTATTTTTATACCTCTTATGAAATGAAAAAAAATATCAATTC--TCCATGGCTTTTTTC
                **** ******* * ** * ****** *** ** * **    *   * *  **           *  ** ***      *   *******
```

```
BnaC6.DA2       ACTTTTGTCTAAACAGGAAACAGGGATGCAGAGAGATTCAGGTGGTGGGGGAGTGACACCTTTTAGGCAGTATGTATCAGAAGAAGATCA
BnaA2.DA2.1     ACTTTGTTTCAAACAGGAAACAGAGATGGAGAGAAGTTCAGGTAGTGGAGAGACTGCACCTTGTAGTCAGTATGTATCAGAAGAAGAACC
BnaA2.DA2.2     ACTTTGTTTCAAACAGGAAACAGAGATGGAGAGAAGTTCAGGTAGTGGAGAGACTGCACCTTGTAGTCAGTATGTATCAGAAGAAGAACC
                *****  *  ************* **** *****  ******* **** *      ****** *** ******************** *

BnaC6.DA2       TAGTTATGCAGAACCAGCAACCCCGTCTTCATCATCTGGTGGACTTGCTTGTGCAATCTCTGCACTTGCTGAGCAACGCCAGCAAATAAT
BnaA2.DA2.1     AGCAA---------------------------------TGCCGTCGTCTTGTGCAATCTCTGCACT---TGCTGAACGCCAGCAAATGGT
BnaA2.DA2.2     AGCAA---------------------------------TGCCGTCGTCTTGTGCAATCTCTGCACT---TGCTGAACGCCAGCAAATGGT
                                                  *******************   **   *************

BnaC6.DA2       TGGAGAATCCTCTAATCACAATCACAACGTCAACGTTGCTTCATACAGTATGCTTCCTGGCAACTGCGACAGTTACTACGACATAGAACA
BnaA2.DA2.1     TGGTGAATCCTCCAATCACAATCACAACGTCAACGTTTCTTCATACACTATGCTTCCTGGCAACTGCGACAGTTACTACGACGTAGAACA
BnaA2.DA2.2     TGGTGAATCCTCCAATCACAATCACAACGTCAACGTTTCTTCATACACTATGCTTCCTGGCAACTGCGACAGTTACTACGACGTAGAACA
                *** ******** ************************ ********* *********************************** ******

BnaC6.DA2       AGACGCAGATGACATTGACCACTATCATCATTATAATCACTACCAAAACAACACCGAGATGGGAGAAACAGGGAGCAACAGCTCTTACAT
BnaA2.DA2.1     AGAAGTAGACGACATTGACCACCACCATAATCAT------TACCATAACAACACTGAGATGGGAGAAACAGGGAGCAGCAGCTATGTAAG
BnaA2.DA2.2     AGAAGTAGACGACATTGACCACCACCATAATCAT------TACCATAACAACACTGAGATGGGAGAAACAGGGAGCAGCAGCTATGTAAG
                *** * *** ************ * *** ** **      ***** ******** ********************** ***** *   *

BnaC6.DA2       GAACGGCGGCGAGAGCTACCACAACTTTCCTCTTCCACCACCTCCTCCTCTGGTGATTGCTCCAGAGAGTTTTGAGGAGCAGATGATGAT
BnaA2.DA2.1     CTCGTACATGAACGGAGAGACCTTCCACAACTTTCCTCCTCCTCCTCCTATGGTCATTGTTCCAGATAGTTTTGAAGAGCAGATGATGAT
BnaA2.DA2.2     CTCGTACATGAACGGAGAGGGCTTCCACAACTTTCCTCCTCCTCCTCCTATGGTCATTGTTCCAGAGAGTTTTGAAGAGCAGATGATGAT
                      *    *  *  *   *  *       **** ** ********* **** **** ****** ******** **************

BnaC6.DA2       GGCTATGGCTGTGTCTTTGGCAGAGGTTCATGCCACGACCACCACAAGCGCACCAACTGAAGTTACTTGGCAATAAGTAAGTAGGAGGGC
BnaA2.DA2.1     GGCTATGGCTGTGTCGTTGGCAGAGGTTCATGCCACGACCACAAGT---GCACCAACTGAAGTTAGTTGGCAATAAAATAGTGTAGTAAT
BnaA2.DA2.2     GGCTATGGCTGTGTCTTTGGCAGAGGTTCATGCCACGACCACAAGT---GCACCAACTGAAGTTAGTTGGCAATAAAATAGTGTAGTAAT
                *************** ************************** *     **************** **********   ***
```

Figure S5: Genomic sequence alignment and sgRNA locations for the *BnaFUL* family genes. The paralogous gene-specific sgRNA targets are indicated with light blue. The PAM motif (NGG) is indicated with a grey box. The start codons are indicated with a yellow box. The stop codons are indicated with a blue box.

```
BnaA9.FUL   --------------------------------------------------------ATGGGAAGGGGTAGGGTTCAGCTGAAGAGGATAG
BnaC7.FUL   AAGGTTGTCGTTTCTCTCTCTTTTTCTTGAGCTTTTGAGGAGAGAGAGAGAGAGACATGGGAAGGGGTAGGGTTCAGCTGAAGAGGATAG
BnaC2.FUL   -----------TTCTCTCTCTTGTTCTTGAGG------TTTTGAAGAGAAAGAGAGATGGGAAGGGGTAGGGTTCAGCTGAAGAGGATAG
                                                                    **********************************

BnaA9.FUL   AGAACAAGATCAATAGGCAAGTTACTTTCTCAAAGAGAAGGTCTGGTTTGCTCAAGAAAGCTCATGAGATCTCTGTTCTCTGCGATGCTG
BnaC7.FUL   AAAACAAGATCAATAGGCAAGTTACTTTCTCTAAGAGAAGGTCTGGTTTGCTCAAGAAAGCTCATGAGATCTCTGTTCTCTGCGATGCTG
BnaC2.FUL   AGAATAAGATAAATAGGCAAGTTACTTTCTCAAAGAGAAGGTCTGGTTTGCTCAAGAAAGCTCATGAGATCTCTGTTCTCTGCGATGCTG
            * ** ***** ******************** **********************************************************

BnaA9.FUL   AGGTTGCTCTCGTTGTCTTCTCTTCCAAAGGCAAACTCTTCGAATATTCCACTGACTCTAGCATGGAAAGGATACTTGAGCGATATGATC
BnaC7.FUL   AGGTTGCCCTCGTCATCTTCTCTTCCAAAGGCAAACTCTTCGAATATTCCACCGACTCTTGCATGGAAAGGATACTTGAACGCTACGATC
BnaC2.FUL   AGGTTGCTCTCATCGTCTTCTCTTCCAAAGGCAAACTCTTCGAATATTCCACAGACTCTTGCATGGAAAGCATACTTGAACGCTATGATC
            ******* *** *  ************************************* ****** ********** ******** ** ** ****

BnaA9.FUL   GCTATTTATATTCAGACAAACAACTTGTTGGCCGAGACATTTCACAAAGTGAAAATTGGGTTCTAGAGCATGCTAAGCTCAAGGCAAGAG
BnaC7.FUL   GCTATTTGTATTCGGACAAACAACTTGTAGGCCGAGAGATTTCACAGAGTGAAAATTGGGTTCTTGAACATGCTAAGCTCAAGGCTAGAA
BnaC2.FUL   GCTATTTGTACTCAGATAAACAACTTGTTGGCAGAGACATTTCACAGATTGAAAATTGGGTTCTAGAACATGCCAAGCTCAAGGCAAGAG
            ******* ** ** ** *********** *** **** ******** * *************** ** ***** *********** *** 

BnaA9.FUL   TTGAGGTACTTGAAAAGAATAAAAGGAATTTTATGGGGGAAGATCTTGATTCCTTGAGCATAAAGGAGCTTCAAAGCTTGGAGCATCAGC
BnaC7.FUL   TTGAGGTACTCGAGAAGAACAAAAGGAATTTTATGGGGGAAGATCTTGATTCGTTGAGCTTAAAGGAGCTCCAAAGCTTGGAACATCAGC
BnaC2.FUL   TTGAGGTACTTGAGAAGAACAAAAGGAATTTTATGGGGGAAGAACTTGAATCTTTGAGTTTAAAGGACCTTCAAAGCCTGGAGCATCAGC
            ********** ** ***** *********************** ***** ** *****  ******* ** ****** **** *******

BnaA9.FUL   TCGACGCTGCTATCAAGAGCATTAGGTCAAGAAAGAACCAAGCTATGTTCGAATCCATATCAGCGCTCCAGAAGAAGGATAAGGCCTTGC
BnaC7.FUL   TCCATGCTGCTATCAAGAGCATTAGGTCAAGAAAGAACCAAGCTATGTTCGAATCCATATCGGCGCTCCAAAAGAAGGATAAGGCCTTGC
BnaC2.FUL   TCGATGCTGCTATCAAAAGCATTAGGTCAAGAAAGAACCAAGCTATGTTCGAATCCATATCAGCGCTCCAGAAGAAGGATAAGGCCTTGC
            ** * *********** ******************************************** ******** *******************
```

```
BnaA9.FUL   AAGATCACAATAATACGCTTCTCAAAAAGATTAAGGAGAAGGAAAAGGAGAAGAACACGGGTCAGCAAGAAGGACAATTAATCCAATGCT
BnaC7.FUL   AAGATCACAACAATGCGCTTCTCAAAAAGATTAAGGAGAGG------GAGAAGAACACGGTTCAGCAAGGAGGACAACTAATCCAATGCT
BnaC2.FUL   AAGATCACAATAATACGCTTCTCAAAAAGATTAAGGAGAGG------GAGAAGAAAACGGGTCATCAAGAAGGACAATTAAACCATTGCT
            ********** *** ************************ *      ******** **** *** **** ******* *** *** ****

BnaA9.FUL   CCAACAATTCTTCAGTTCTTCAGCCCCAGTACTGCGTAACCGCCTCCAGAGATGGCCTTGTGGAGAGAGTTGTGGGAGAGAACGGCGGTG
BnaC7.FUL   CCAACAGTTCTTCAATTCTTCAGCCCCAATACTGCTTAACCTCCTCCAGAGATGGCTTCGTGGGGAGGGTTGGGGAAGAGAACGGCGTAG
BnaC2.FUL   CCAACAATTCTTCAATTGTTCAGCCCCAGTACTGTTTAACCTCCTCCAGAGATGGCTTTGTGGGGAGAGTTGGGGGAGAGAATGGTGGAG
            ****** ******* ** ********** *****  ***** ************** * **** *** **** ** ****** ** *  *

BnaA9.FUL   CATCGTCGTTGATTGAACCAAACTCTCTTCTTCCAGCTTGGATGCTACGTTCAAATGAGTAAAATTACCTAAGTAT------GAT-----
BnaC7.FUL   CATCATCACTGGCGGAACCAAACTCTCTTCTTCCGGCTTGGATGTTACGCCCTACCACGAATGAATAG----------------------
BnaC2.FUL   CATCGTCATTGACGGAACCAAACTCTCTTCTTCCGGCTTGGATGTTACGCCCTACGACGAATGAATAGAATTATCTATATATTATAAGAT
            **** **  **   ******************** ********* ****  * *    * *  * **
```

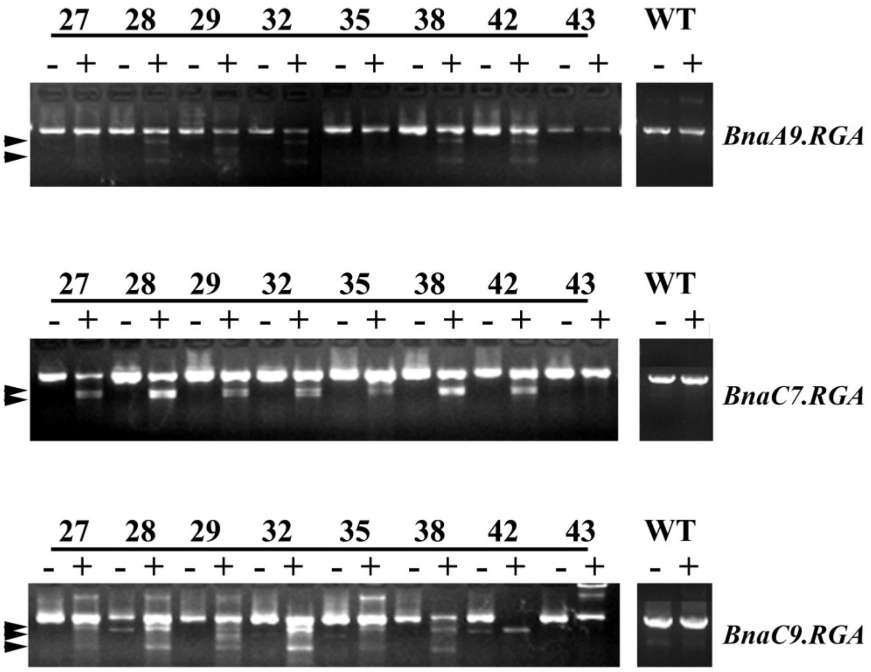

Figure S6. Detection of target mutations using the T7 endonuclease I (T7E1) assay. From the top to the bottom, the gel images show the T7E1-digested products of *BnaA9.RGA*, *BnaC9.RGA,* and *BnaC7.RGA*, respectively. Arrows indicate the position of the products cleaved by T7E1. +: with T7E1 digestion. −: without T7E1 digestion. The numbers indicate the different transgenic lines of *BnaRGA-sgRNA* in the T0 generation.

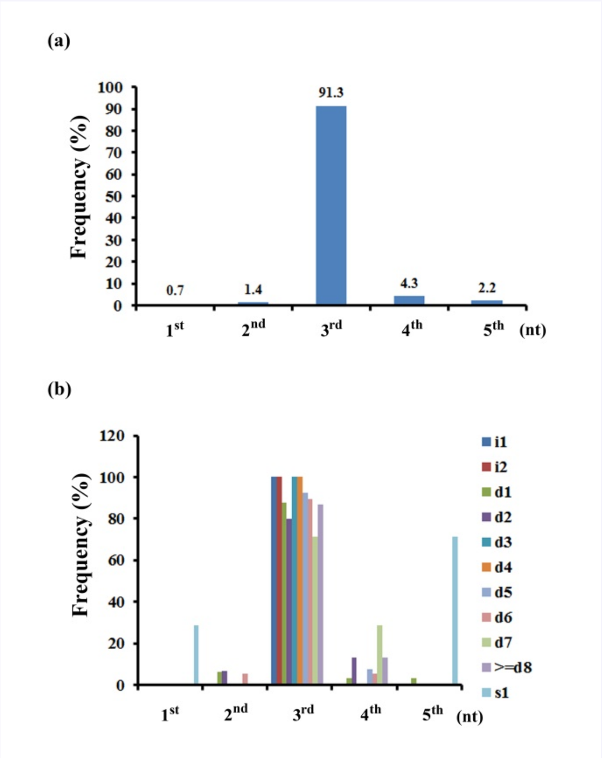

Figure S7. The frequency of cleavage sites upstream of the PAM sequence.

(a) Bar graph shows the frequency of cleavage sites upstream of the PAM sequence among all of the mutations.

(b) Bar graph shows the frequency of cleavage sites upstream of the PAM sequence among all of the mutations classified by different mutation types. i: insertion; d: deletion; s: substitution. The d number indicates the number of bp deleted from the target site. The i number indicates the number of bp inserted at the target site. The s number indicates the number of substituted bp.

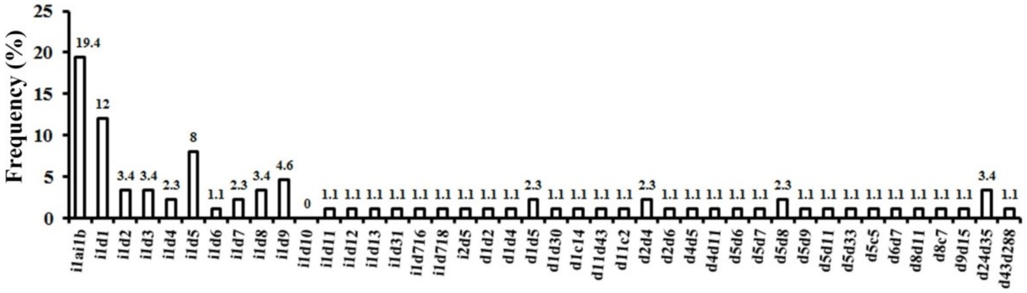

Figure S8. Frequency of each mutation type for bi-allelic mutations.

The bar graph shows the frequency of each mutation type for all bi-allelic mutations detected in the T0 generation, i: insertion; d: deletion. The d number, indicates the number of bp deleted from the target site. The i number indicates the number of bp inserted at the target site.

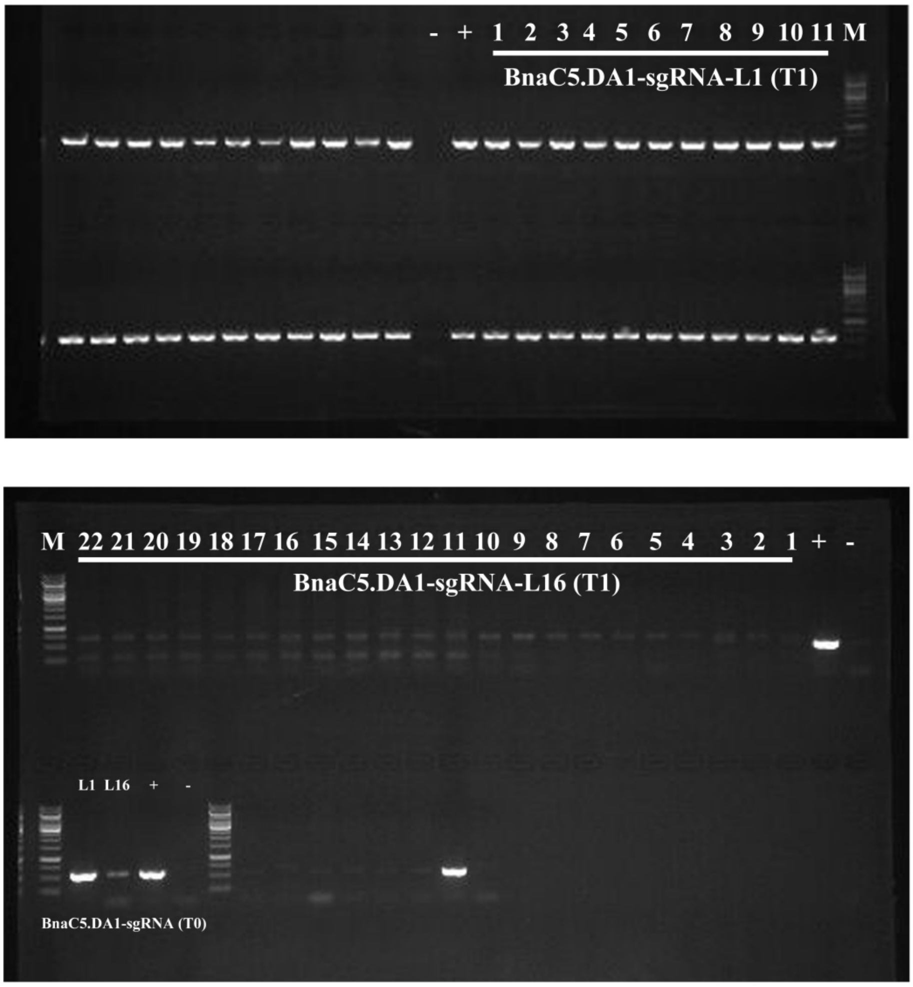

Figure S9. Original image shows the PCR products of Cas9 in different progeny of *BnaC5.DA1-sgRNA-L16* in the T0 and T1 generations.

The original gel image shows the PCR products of *Cas9* in B*naC5.DA1-sgRNA-L16* in the T0 and T1 generations.

+: pKSE401 was used as the positive control. -: gDNA of WT was used as the negative control. *BnaC5.DA1-sgRNA-L1* (T0 and T1) was used as the positive control with the *Cas9* insertion.

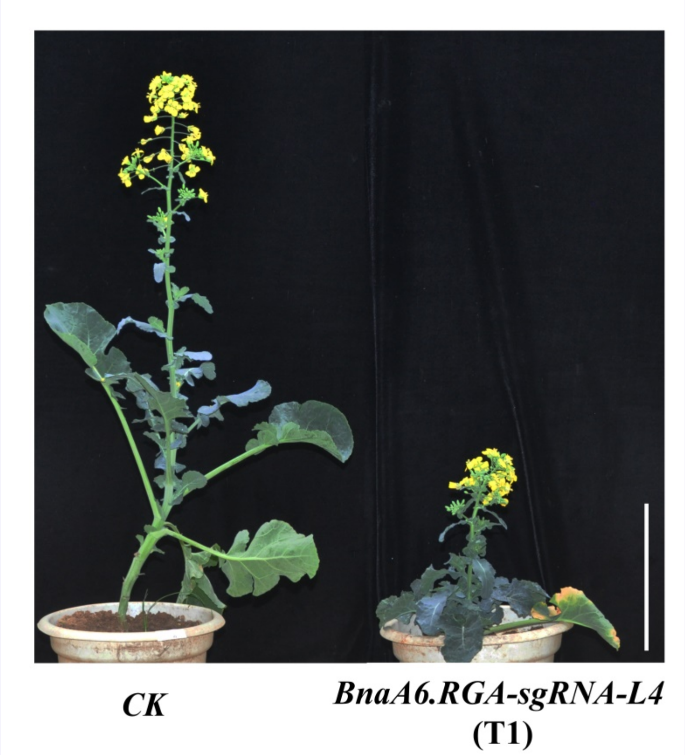

Figure S10. Phenotype of *BnaA6.RGA-sgRNA* transgenic plants in the T1 generation. An image shows the morphology of the phenotype induced by *BnaA6.RGA-sgRNA* (L4-T1) at the same age as the control. CK: WT plant. Bar = 15cm.

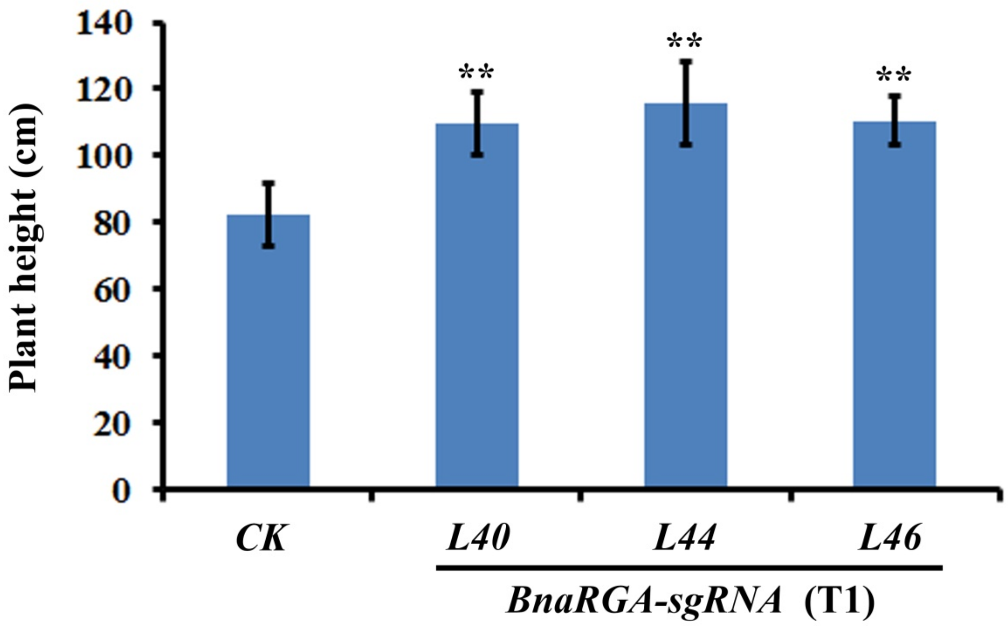

Figure S11. Stem length of the *BnaRGA-sgRNA* quadruple mutant T1 plants.

The Bar graph shows the stem length of T1 quadruple mutants induced by *BnaRGA-sgRNA* (L40, L44 and L46) and control plants at 125 DAS (Days After Sowing). Error bars represent SD (n=15-20). **: $p < 0.01$, Heteroscedastic t-test.

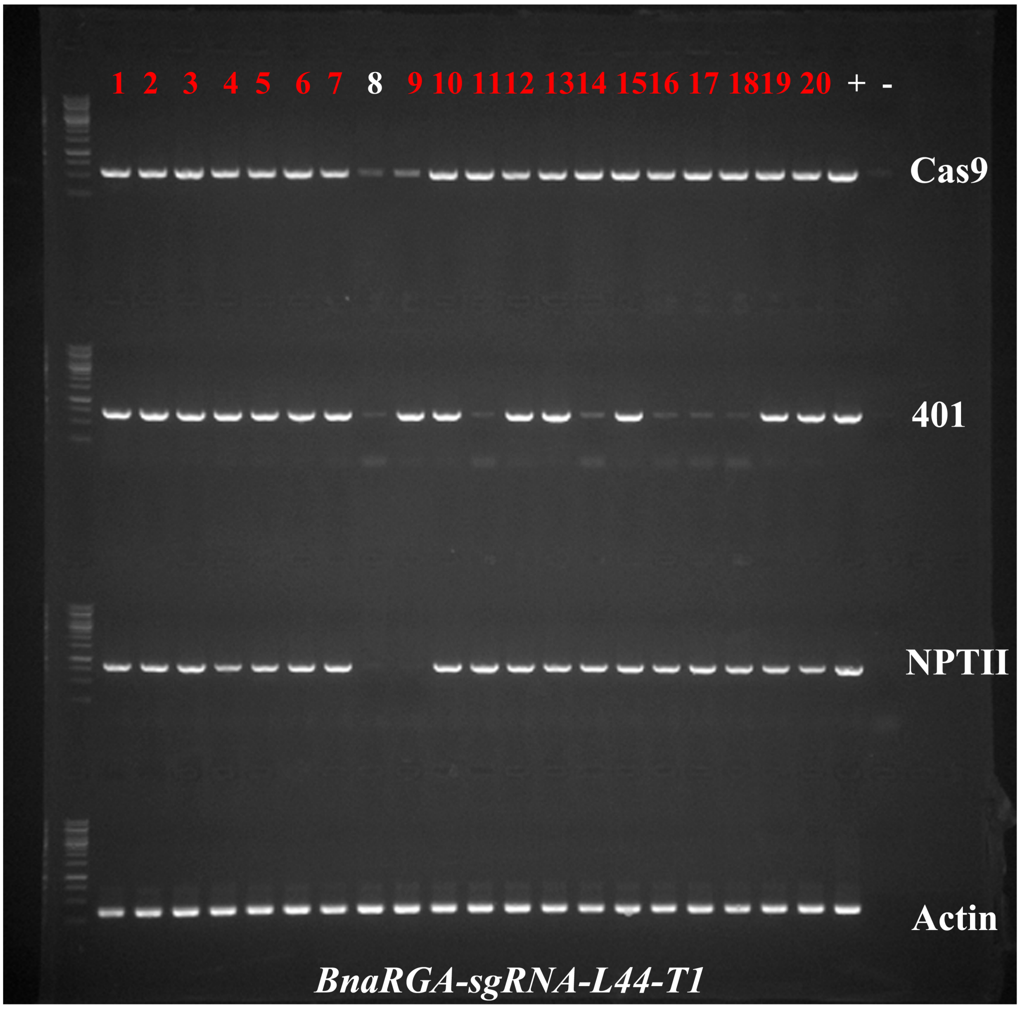

Figure S12. Image showing the PCR products from *Cas9*, *NPTII* and *pKSE401* in different progeny of BnaRGA-L44 in the T1 generation.

The original gel image shows PCR products from *Cas9*, *NPTII* and *pKSE401* in BnaRGA-sgRNA-L44 from the T1 generation. The red label indicates the transgenic lines containing the vector fragment.

+: pKSE401 was used as the positive control; -: gDNA from *Westar* was used as the negative control for *Cas9*, *NPTII* and *pKSE401*. ddH$_2$O was used as the negative control for *Actin1*. The *B. napus* actin1 gene was used as the PCR internal control.

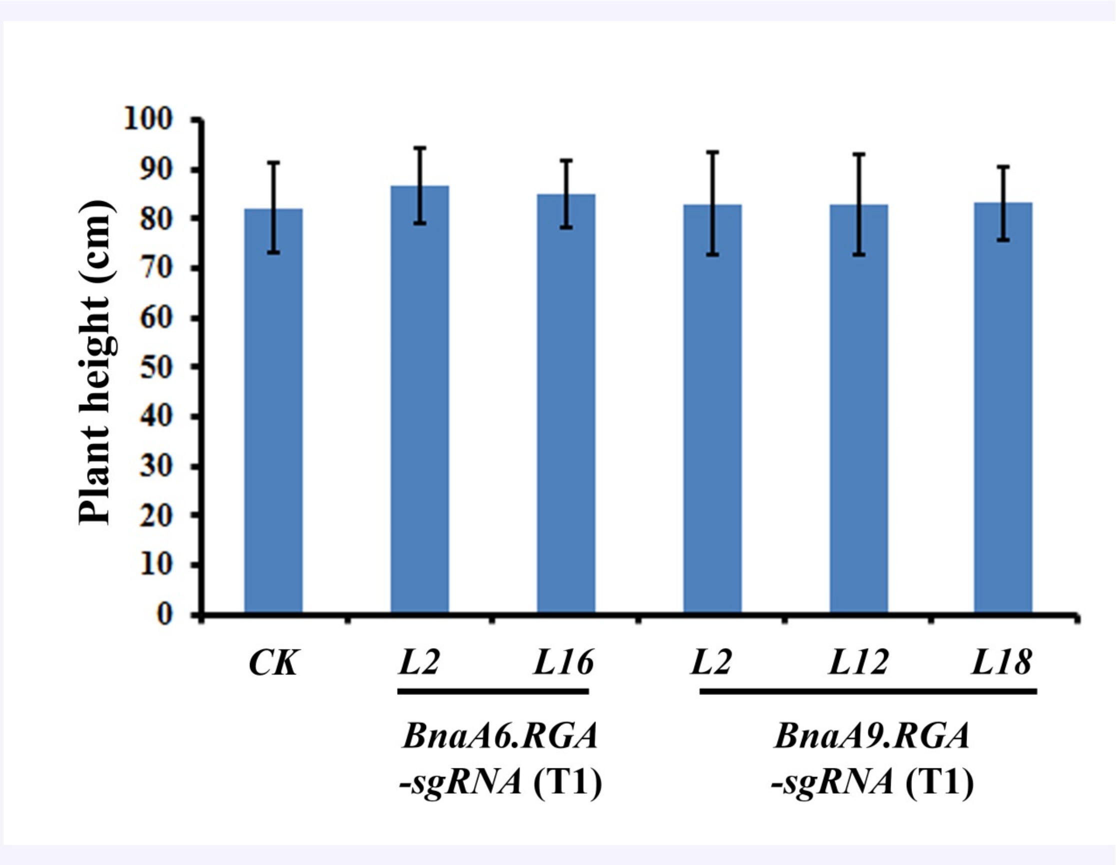

Figure S13. The stem length of *BnaA6.RGA-sgRNA* and *BnaA9.RGA-sgRNA* transgenic plants in the T1 generation.

The bar graph shows the stem length of T1 mutants induced by *BnaA6.RGA-sgRNA* (L2 and L16) and *BnaA9.RGA-sgRNA* (L2, L12 and L18). The control plants at 125 DAS (Days After Sowing). Error bars represent SD (n=15-20). Heteroscedastic t-test analysis showed no significant difference.

Supplemental Table S1. Selected target genes in *B. napus* used to assess CRISPR/Cas9.

Target Gene Family	Gene Identifier	Gene Product	Expected knock-out phenotype
BnaRGA	BnaA09g18700D	Arabidopsis RGA homolog	Versatile phenotypes due to enhanced GA signaling, such as longer stems, etc.
	BnaC09g52270D	Arabidopsis RGA homolog	
	BnaA06g34810D	Arabidopsis RGA homolog	
	BnaC07g20900D	Arabidopsis RGA homolog	
BnaDA2	BnaA02g18880D	Arabidopsis DA2 homolog	Increase in organ size
	BnaA02g18890D	Arabidopsis DA2 homolog	
	BnaC06g38900D	Arabidopsis DA2 homolog	
BnaDA1	BnaA06g13370D	Arabidopsis DA1 homolog	Increase in organ size
	BnaC05g14930D	Arabidopsis DA1 homolog	
BnaFUL	BnaC07g49790D	Arabidopsis FUL homolog	Failure of dehiscence, premature rupture of the carpel valves
	BnaA09g05500D	Arabidopsis FUL homolog	
	BnaC02g41870D	Arabidopsis FUL homolog	

Supplemental Table S2. Summary of the sgRNAs (%GC content and mutation rates). The PAM motif (NGG) is indicated with a grey box.

Target genes	Target sequence (PAM in gray)	GC content	Mutation rate
BnaA9.RGA	**sgRNA1: GAGGTCGTCCGAGATGGCCGAGG**	**70%**	**80.0%**
	**sgRNA2: ACCCGTCGGAGCTCTACTCGTGG**	**65%**	**70.0%**
BnaC9.RGA	**sgRNA1: CAAGGTGAGGTCGTCAGAGATGG**	**55%**	**100.0%**
	**sgRNA2: ACCCGTCGGAGCTTTACTCGTGG**	**60%**	**100.0%**
BnaA6.RGA	**sgRNA1: CAAGGTTAGGTCTTCGGAGATGG**	**50%**	**63.3%**
	**sgRNA2: ACCCCGCTGAGCTTTACTCGTGG**	**60%**	**70.0%**
BnaC7.RGA	**sgRNA1: CAGGTCGTCGGAGATGGCTGAGG**	**65%**	**5.3%**
	**sgRNA2: ACCCTGCTGAGCTCTACTCTTGG**	**55%**	**42.1%**
BnaA2.DA2	**sgRNA1: GCACAAGACGACGGCATTGCTGG**	**60%**	**90.2%**
	**sgRNA2: GGGAGCTAAGTACGTAGACTTGG**	**50%**	**43.9%**
BnaC5.DA1	**sgRNA1: GAACACCTTAGGATTTGTGCTGG**	**45%**	**40.0%**
	**sgRNA2: CGATGGACGGAAACTTTGCCTGG**	**55%**	**100%**
BnaA6.DA1	**sgRNA1: TATGTGCAGCCACGCAATACAGG**	**50%**	**64.1%**
	**sgRNA2: GAAGAGAGGTTACTATGGGTTGG**	**45%**	**40.6%**
BnaRGA	**sgRNA1: AGTGAGCGAACTTGAGGTAAGGG**	**45%**	***BnaA9.RGA*:96.6%, *BnaC9.RGA*:86.2%, *BnaA6.RGA*:96.6%, *BnaC7.RGA*:93.1%**
	**sgRNA2: GATTAAACCGGTGATTTTCACGG**	**35%**	***BnaA9.RGA*:27.6%, *BnaC9.RGA*:31.0%, *BnaA6.RGA*:34.5%, *BnaC7.RGA*:41.4%**
BnaFUL	**sgRNA1: ATATTCGAAGAGTTTGCCTTTGG**	**35%**	***BnaA9.FUL*:76.2%, *BnaC2.FUL*: 76.2%, *BnaC7.FUL*:76.2%**
	**sgRNA2: GGGGTAGGGTTCAGCTGAAGAGG**	**60%**	***BnaA9.FUL*:85.7%, *BnaC2.FUL*:81.0%, *BnaC7.FUL*:85.7%**
BnaDA2	**sgRNA1:TAGTAACTGTCGCAGTTGCCAGG**	**50%**	***BnaA2.DA2*:41.2%, *BnaC6.DA2*: 35.3%**
	**sgRNA2: TGCATGAAAAGCATTTGTACAGG**	**35%**	***BnaA2.DA2*:47.1%, *BnaC6.DA2*: 82.4%**

Supplemental Table S3. Sequencing results from the T0 and T1 plants.

In the T0 generation, DNA was extracted from a leaf for most plants and also from mixed tissues for some plants. The DNA was amplified using PCR and sequenced. The sequences were uploaded to the following website: http://dsdecode.scgene.com/ for sequence decoding. For the PCR amplicons that contained multiple mixed sequences that could not be decoded, the DNA was cloned into a TA vector, and each clone was individually sequenced. The genotypes were classified as heterozygous, homozygous mutants, or WT. In the T1 generation, DNA was extracted and PCR amplified, and then sequenced without cloning. M/N: N refers to the number of T1 plants examined, and M refers to the number of plants showing a particular genotype. WT indicates a wild-type sequence with no mutations detected. The d number indicates the number of bp deleted from the target site. The d number a, indicates the same number of deletion at one site. The d number b indicates the same number of deletions at other sites. The i number indicates the number of bp inserted at a target site. The i number followed with "a" indicates the same number of insertions at one site. The i number followed with "b" indicates the same number of insertions of different nucleotide at the same site. h indicates heterogeneous (i.e., more than one sequence detected in the sample). The name of transgenic lines is indicated with a bold font. The sgRNAs are indicated with red. The mutations are indicated with yellow. The PAM motif (NGG) is indicated with a grey box.

BnaA6.RGA, BnaRGA-sgRNA1**，T0-line40**

T0-leaf:

```
d1:   TTCTACGAGACTTGCCCTTAC-TCAAGTTCGCTCACTTCACGGCGAATCAGGCGATTCTCGAGGCTTTCG
Ref:  TTCTACGAGACTTGCCCTTACCTCAAGTTCGCTCACTTCACGGCGAATCAGGCGATTCTCGAGGCTTTCG
```

T0-mixed tissues:

```
d1:   TTCTACGAGACTTGCCCTTAC-TCAAGTTCGCTCACTTCACGGCGAATCAGGCGATTCTCGAGGCTTTCG
Ref:  TTCTACGAGACTTGCCCTTACCTCAAGTTCGCTCACTTCACGGCGAATCAGGCGATTCTCGAGGCTTTCG
```

T1: Distribution of plants with different genotypes (M/N)

```
d1:   TTCTACGAGACTTGCCCTTAC-TCAAGTTCGCTCACTTCACGGCGAATCAGGCGATTCTCGAGGCTTTCG
Ref:  TTCTACGAGACTTGCCCTTACCTCAAGTTCGCTCACTTCACGGCGAATCAGGCGATTCTCGAGGCTTTCG
```

d1d1 (8/8)

BnaA6.RGA, BnaRGA-sgRNA1**，T0-line44**

T0-leaf:

```
i1:   TTCTACGAGACTTGCCCTTAACCTCAAGTTCGCTCACTTCACGGCGAATCAGGCGAT
Ref:  TTCTACGAGACTTGCCCTTA-CCTCAAGTTCGCTCACTTCACGGCGAATCAGGCGAT
```

T0-mixed tissues:

```
i1:   TTCTACGAGACTTGCCCTTAACCTCAAGTTCGCTCACTTCACGGCGAATCAGGCGAT
Ref:  TTCTACGAGACTTGCCCTTA-CCTCAAGTTCGCTCACTTCACGGCGAATCAGGCGAT
```

T1: Distribution of plants with different genotypes (M/N)

```
i1:   TTCTACGAGACTTGCCCTTAACCTCAAGTTCGCTCACTTCACGGCGAATCAGGCGAT
Ref:  TTCTACGAGACTTGCCCTTA-CCTCAAGTTCGCTCACTTCACGGCGAATCAGGCGAT
```

i1i1	(17/17)

BnaA9.RGA, BnaRGA-sgRNA1**，T0-line44**

T0-leaf:

d6:   GCACTTCTACGAGACGTGCCCTTA------GTTCGCTCACTTCACGGCCAACCAAGCCATCCTCGAGG

Ref: GCACTTCTACGAGACGTGCCCTTACCTCAAGTTCGCTCACTTCACGGCCAACCAAGCCATCCTCG

T0-mixed tissues:

d6:   GCACTTCTACGAGACGTGCCCTTA------GTTCGCTCACTTCACGGCCAACCAAGCCATCCTCGAGG

Ref: GCACTTCTACGAGACGTGCCCTTACCTCAAGTTCGCTCACTTCACGGCCAACCAAGCCATCCTCG

T1: Distribution of plants with different genotypes (M/N)

d6:   GCACTTCTACGAGACGTGCCCTTA------GTTCGCTCACTTCACGGCCAACCAAGCCATCCTCGAGG

Ref: GCACTTCTACGAGACGTGCCCTTACCTCAAGTTCGCTCACTTCACGGCCAACCAAGCCATCCTCG

d6d6 (17/17)

***BnaA6.RGA, BnaA6.RGA-sgRNA1*，T0-line5**

T0-leaf:

d5: ATACTGTTCATTACAACCCCGCTGAGC-----TCGTGGCTTGATAACATGCTCACGGAGCTTAA

d6: ATACTGTTCATTACAACCCCGCTGAG------TCGTGGCTTGATAACATGCTCACGGAGC

Ref: ATACTGTTCATTACAACCCCGCTGAGCTTTACTCGTGGCTTGATAACATGCTCACGGAGC

T0-mixed tissues:

d5: ATACTGTTCATTACAACCCCGCTGAGC-----TCGTGGCTTGATAACATGCTCACGGAGCTTAA

d6: ATACTGTTCATTACAACCCCGCTGAG------TCGTGGCTTGATAACATGCTCACGGAGC

Ref: ATACTGTTCATTACAACCCCGCTGAGCTTTACTCGTGGCTTGATAACATGCTCACGGAGC

T1: Distribution of plants with different genotypes (M/N)

d5: ATACTGTTCATTACAACCCCGCTGAGC-----TCGTGGCTTGATAACATGCTCACGGAGCTTAA

d6: ATACTGTTCATTACAACCCCGCTGAG------TCGTGGCTTGATAACATGCTCACGGAGC

Ref: ATACTGTTCATTACAACCCCGCTGAGCTTTACTCGTGGCTTGATAACATGCTCACGGAGC

d5d6 (9/20)

d5: ATACTGTTCATTACAACCCCGCTGAGC-----TCGTGGCTTGATAACATGCTCACGGAGCTTAA

Ref: ATACTGTTCATTACAACCCCGCTGAGCTTTACTCGTGGCTTGATAACATGCTCACGGAGC

d5d5 (9/20)

d6: ATACTGTTCATTACAACCCCGCTGAG------TCGTGGCTTGATAACATGCTCACGGAGC

Ref: ATACTGTTCATTACAACCCCGCTGAGCTTTACTCGTGGCTTGATAACATGCTCACGGAGC

d6d6 (2/20)

***BnaA6.RGA, BnaA6.RGA-sgRNA1*，T0-line6**

T0-leaf:

```
d9:   TCTTGGGTTACAAGGTTAGG---------ATGGCTGAGGTTGCGTTGAAACTCGAGCAGCTTGAGACGAT
d5:   TCTTGGGTTACAAGGTTAGGTC-----AGATGGCTGAGGTTGCGTTGAAACTCGAGCAGCTTGAGACGAT
Ref:  TCTTGGGTTACAAGGTTAGGTCTTCGGAGATGGCTGAGGTTGCGTTGAAACTCGAGCAGCTTGAGACGAT
```

T0-mixed tissues:

```
d9:   TCTTGGGTTACAAGGTTAGG---------ATGGCTGAGGTTGCGTTGAAACTCGAGCAGCTTGAGACGAT
d5:   TCTTGGGTTACAAGGTTAGGTC-----AGATGGCTGAGGTTGCGTTGAAACTCGAGCAGCTTGAGACGAT
Ref:  TCTTGGGTTACAAGGTTAGGTCTTCGGAGATGGCTGAGGTTGCGTTGAAACTCGAGCAGCTTGAGACGAT
```

T1: Distribution of plants with different genotypes (M/N)

```
d9:   TCTTGGGTTACAAGGTTAGG---------ATGGCTGAGGTTGCGTTGAAACTCGAGCAGCTTGAGACGAT
d5:   TCTTGGGTTACAAGGTTAGGTC-----AGATGGCTGAGGTTGCGTTGAAACTCGAGCAGCTTGAGACGAT
Ref:  TCTTGGGTTACAAGGTTAGGTCTTCGGAGATGGCTGAGGTTGCGTTGAAACTCGAGCAGCTTGAGACGAT
d5d9                                                                          (5/9)

d5:   TCTTGGGTTACAAGGTTAGGTC-----AGATGGCTGAGGTTGCGTTGAAACTCGAGCAGCTTGAGACGAT
Ref:  TCTTGGGTTACAAGGTTAGGTCTTCGGAGATGGCTGAGGTTGCGTTGAAACTCGAGCAGCTTGAGACGAT
d5d5                                                                          (4/9)
```

BnaA6.RGA, BnaRGA-sgRNA1**，T0-line46**

T0-leaf:

```
d5:   TTCTACGAGACTTGCCCTTA------AGTTCGCTCACTTCACGGCGAATCAGGCGATTCTCGAGGCTTTCG
i1:   TTCTACGAGACTTGCCCTTATCCTCAAGTTCGCTCACTTCACGGCGAATCAGGCGATTCTCGAGGCTTTCG
Ref:  TTCTACGAGACTTGCCCTTA-CCTCAAGTTCGCTCACTTCACGGCGAATCAGGCGATTCTCGAGGCTTTCG
```

T0-mixed tissues:

```
d5:   TTCTACGAGACTTGCCCTTA------AGTTCGCTCACTTCACGGCGAATCAGGCGATTCTCGAGGCTTTCG
i1:   TTCTACGAGACTTGCCCTTATCCTCAAGTTCGCTCACTTCACGGCGAATCAGGCGATTCTCGAGGCTTTCG
Ref:  TTCTACGAGACTTGCCCTTA-CCTCAAGTTCGCTCACTTCACGGCGAATCAGGCGATTCTCGAGGCTTTCG
```

T1: Distribution of plants with different genotypes (M/N)

```
d5:   TTCTACGAGACTTGCCCTTA------AGTTCGCTCACTTCACGGCGAATCAGGCGATTCTCGAGGCTTTCG
i1:   TTCTACGAGACTTGCCCTTATCCTCAAGTTCGCTCACTTCACGGCGAATCAGGCGATTCTCGAGGCTTTCG
Ref:  TTCTACGAGACTTGCCCTTA-CCTCAAGTTCGCTCACTTCACGGCGAATCAGGCGATTCTCGAGGCTTTCG
i1d5                                                                          (9/17)
```

```
d5:   TTCTACGAGACTTGCCCTTA------AGTTCGCTCACTTCACGGCGAATCAGGCGATTCTCGAGGCTTTCG
Ref:  TTCTACGAGACTTGCCCTTA-CCTCAAGTTCGCTCACTTCACGGCGAATCAGGCGATTCTCGAGGCTTTCG
d5d5                                                                          (2/17)
```

```
i1:   TTCTACGAGACTTGCCCTTATCCTCAAGTTCGCTCACTTCACGGCGAATCAGGCGATTCTCGAGGCTTTCG
Ref:  TTCTACGAGACTTGCCCTTA-CCTCAAGTTCGCTCACTTCACGGCGAATCAGGCGATTCTCGAGGCTTTCG
i1i1                                                                          (6/17)
```

***BnaA9.RGA, BnaRGA-sgRNA1*，T0-line27**

T0-leaf:

d5: ACTTCTACGAGACGTGCCCT-----CAAGTTCGCTCACTTCACGGCCAACCAAGCCATCCTCGAGGCTTT

d1: ACTTCTACGAGACGTGCCCTT-CCTCAAGTTCGCTCACTTCACGGCCAACCAAGCCATCCTCGAGGCTTT

Ref: ACTTCTACGAGACGTGCCCTTACCTCAAGTTCGCTCACTTCACGGCCAACCAAGCCATCCTCGAGGCTTT

T0-mixed tissues:

d5: ACTTCTACGAGACGTGCCCT-----CAAGTTCGCTCACTTCACGGCCAACCAAGCCATCCTCGAGGCTTT

d1: ACTTCTACGAGACGTGCCCTT-CCTCAAGTTCGCTCACTTCACGGCCAACCAAGCCATCCTCGAGGCTTT

Ref: ACTTCTACGAGACGTGCCCTTACCTCAAGTTCGCTCACTTCACGGCCAACCAAGCCATCCTCGAGGCTTT

T1: Distribution of plants with different genotypes (M/N)

d5: ACTTCTACGAGACGTGCCCT-----CAAGTTCGCTCACTTCACGGCCAACCAAGCCATCCTCGAGGCTTT

d1: ACTTCTACGAGACGTGCCCTT-CCTCAAGTTCGCTCACTTCACGGCCAACCAAGCCATCCTCGAGGCTTT

Ref: ACTTCTACGAGACGTGCCCTTACCTCAAGTTCGCTCACTTCACGGCCAACCAAGCCATCCTCGAGGCTTT

d5d1 (20/20)

***BnaC7.RGA, BnaRGA-sgRNA1*，T0-line38**

T0-leaf:

d2:   TTCTACGAGACGTGCCCTTA---TTCGCTCACTTCACGGCGAATCAAGCCATCCTCGAGGCTTTCG

i1:   TTCTACGAGACGTGCCCTTAACCTCAAGTTCGCTCACTTCACGGCGAATCAAGCCATCCTCGAGGCTTTCG

Ref: TTCTACGAGACGTGCCCTTA-CCTCAAGTTCGCTCACTTCACGGCGAATCAAGCCATCCTCGAGGCTTTCG

T0-mixed tissues:

d2:   TTCTACGAGACGTGCCCTTA---TTCGCTCACTTCACGGCGAATCAAGCCATCCTCGAGGCTTTCG

i1:   TTCTACGAGACGTGCCCTTAACCTCAAGTTCGCTCACTTCACGGCGAATCAAGCCATCCTCGAGGCTTTCG

Ref: TTCTACGAGACGTGCCCTTA-CCTCAAGTTCGCTCACTTCACGGCGAATCAAGCCATCCTCGAGGCTTTCG

T1: Distribution of plants with different genotypes (M/N)

d2:   TTCTACGAGACGTGCCCTTA---TTCGCTCACTTCACGGCGAATCAAGCCATCCTCGAGGCTTTCG

i1:   TTCTACGAGACGTGCCCTTAACCTCAAGTTCGCTCACTTCACGGCGAATCAAGCCATCCTCGAGGCTTTCG

Ref: TTCTACGAGACGTGCCCTTA-CCTCAAGTTCGCTCACTTCACGGCGAATCAAGCCATCCTCGAGGCTTTCG

i1d2 (8/15)

i1:   TTCTACGAGACGTGCCCTTAACCTCAAGTTCGCTCACTTCACGGCGAATCAAGCCATCCTCGAGGCTTTCG

Ref: TTCTACGAGACGTGCCCTTA-CCTCAAGTTCGCTCACTTCACGGCGAATCAAGCCATCCTCGAGGCTTTCG

i1i1 (3/15)

d2:   TTCTACGAGACGTGCCCTTA---TTCGCTCACTTCACGGCGAATCAAGCCATCCTCGAGGCTTTCG

Ref: TTCTACGAGACGTGCCCTTA-CCTCAAGTTCGCTCACTTCACGGCGAATCAAGCCATCCTCGAGGCTTTCG

d2d2 (4/15)

***BnaC5.DA1, BnaC5.DA1-sgRNA1*，T0-line1**

T0-leaf:

i1a: GTTAAATAACTTATCAAGAACACCTTAGGATTTGATGCTGGCTGCAATATGGAGATTGGGCATGGAAGA

i1b: GTTAAATAACTTATCAAGAACACCTTAGGATTTGTTGCTGGCTGCAATATGGAGATTGGGCATGGAAGA

Ref: GTTAAATAACTTATCAAGAACACCTTAGGATTTG-TGCTGGCTGCAATATGGAGATTGGGCATGGAAGA

T0-mixed tissues:

i1a: GTTAAATAACTTATCAAGAACACCTTAGGATTTGATGCTGGCTGCAATATGGAGATTGGGCATGGAAGA

i1b: GTTAAATAACTTATCAAGAACACCTTAGGATTTGTTGCTGGCTGCAATATGGAGATTGGGCATGGAAGA

Ref: GTTAAATAACTTATCAAGAACACCTTAGGATTTG-TGCTGGCTGCAATATGGAGATTGGGCATGGAAGA

T1: Distribution of plants with different genotypes (M/N)

i1a: GTTAAATAACTTATCAAGAACACCTTAGGATTTGATGCTGGCTGCAATATGGAGATTGGGCATGGAAGA

i1b: GTTAAATAACTTATCAAGAACACCTTAGGATTTGTTGCTGGCTGCAATATGGAGATTGGGCATGGAAGA

Ref: GTTAAATAACTTATCAAGAACACCTTAGGATTTG-TGCTGGCTGCAATATGGAGATTGGGCATGGAAGA

i1ai1b (1/14)

i1a: GTTAAATAACTTATCAAGAACACCTTAGGATTTGATGCTGGCTGCAATATGGAGATTGGGCATGGAAGA

Ref: GTTAAATAACTTATCAAGAACACCTTAGGATTTG-TGCTGGCTGCAATATGGAGATTGGGCATGGAAGA

i1ai1a (11/14)

i1b: GTTAAATAACTTATCAAGAACACCTTAGGATTTGTTGCTGGCTGCAATATGGAGATTGGGCATGGAAGA

Ref: GTTAAATAACTTATCAAGAACACCTTAGGATTTG-TGCTGGCTGCAATATGGAGATTGGGCATGGAAGA

i1bi1b (2/14)

BnaC5.DA1, BnaC5.DA1-sgRNA1**，T0-line16**

T0-leaf:

i1a: GTTAAATAACTTATCAAGAACACCTTAGGATTTGATGCTGGCTGCAATATGGAGATTGGGCATGGAAGA

i1b: GTTAAATAACTTATCAAGAACACCTTAGGATTTGTTGCTGGCTGCAATATGGAGATTGGGCATGGAAGA

Ref: GTTAAATAACTTATCAAGAACACCTTAGGATTTG-TGCTGGCTGCAATATGGAGATTGGGCATGGAAGA

T0-mixed tissues:

i1a: GTTAAATAACTTATCAAGAACACCTTAGGATTTGATGCTGGCTGCAATATGGAGATTGGGCATGGAAGA

i1b: GTTAAATAACTTATCAAGAACACCTTAGGATTTGTTGCTGGCTGCAATATGGAGATTGGGCATGGAAGA

Ref: GTTAAATAACTTATCAAGAACACCTTAGGATTTG-TGCTGGCTGCAATATGGAGATTGGGCATGGAAGA

T1: Distribution of plants with different genotypes (M/N)

i1a: GTTAAATAACTTATCAAGAACACCTTAGGATTTGATGCTGGCTGCAATATGGAGATTGGGCATGGAAGA

i1b: GTTAAATAACTTATCAAGAACACCTTAGGATTTGTTGCTGGCTGCAATATGGAGATTGGGCATGGAAGA

Ref: GTTAAATAACTTATCAAGAACACCTTAGGATTTG-TGCTGGCTGCAATATGGAGATTGGGCATGGAAGA

i1ai1b (8/16)

i1a: GTTAAATAACTTATCAAGAACACCTTAGGATTTGATGCTGGCTGCAATATGGAGATTGGGCATGGAAGA

Ref: GTTAAATAACTTATCAAGAACACCTTAGGATTTG-TGCTGGCTGCAATATGGAGATTGGGCATGGAAGA

i1ai1a (4/16)

i1b: GTTAAATAACTTATCAAGAACACCTTAGGATTTGTTGCTGGCTGCAATATGGAGATTGGGCATGGAAGA

Ref: GTTAAATAACTTATCAAGAACACCTTAGGATTTG-TGCTGGCTGCAATATGGAGATTGGGCATGGAAGA

i1bi1b (4/16)

BnaA2.DA2, BnaDA2-sgRNA2**，T0-line12**

T0-leaf:

d4: ATAGATATACTTCACCTGTA-----TGCTTTTCATGCAACATCTTGACCTATTGAGGCTAGGATAGTACTA

i1: ATAGATATACTTCACCTGTAACAAATGCTTTTCATGCAACATCTTGACCTATTGAGGCTAGGATAGTACTA

Ref: ATAGATATACTTCACCTGTA-CAAATGCTTTTCATGCAACATCTTGACCTATTGAGGCTAGGATAGTACTA

T0-mixed tissues:

d4: ATAGATATACTTCACCTGTA-----TGCTTTTCATGCAACATCTTGACCTATTGAGGCTAGGATAGTACTA

i1: ATAGATATACTTCACCTGTAACAAATGCTTTTCATGCAACATCTTGACCTATTGAGGCTAGGATAGTACTA

Ref: ATAGATATACTTCACCTGTA-CAAATGCTTTTCATGCAACATCTTGACCTATTGAGGCTAGGATAGTACTA

T1: Distribution of plants with different genotypes (M/N)

d4: ATAGATATACTTCACCTGTA-----TGCTTTTCATGCAACATCTTGACCTATTGAGGCTAGGATAGTACTA

i1: ATAGATATACTTCACCTGTAACAAATGCTTTTCATGCAACATCTTGACCTATTGAGGCTAGGATAGTACTA

Ref: ATAGATATACTTCACCTGTA-CAAATGCTTTTCATGCAACATCTTGACCTATTGAGGCTAGGATAGTACTA

i1d4 (2/15)

d4: ATAGATATACTTCACCTGTA-----TGCTTTTCATGCAACATCTTGACCTATTGAGGCTAGGATAGTACTA

Ref: ATAGATATACTTCACCTGTA-CAAATGCTTTTCATGCAACATCTTGACCTATTGAGGCTAGGATAGTACTA

d4d4 (13/15)

***BnaC6.DA2, BnaDA2-sgRNA2*，T0-line20**

T0-leaf:

i1a: TGATGAATTTTTCACCTGTAACAAATGCTTTTCATGCAACATCTTGATCTATTGAGGC

i1b: TGATGAATTTTTCACCTGTACCAAATGCTTTTCATGCAACATCTTGATCTATTGAGGC

Ref: TGATGAATTTTTCACCTGTA-CAAATGCTTTTCATGCAACATCTTGATCTATTGAGGC

T0-mixed tissues:

i1a: TGATGAATTTTTCACCTGTAACAAATGCTTTTCATGCAACATCTTGATCTATTGAGGC

i1b: TGATGAATTTTTCACCTGTACCAAATGCTTTTCATGCAACATCTTGATCTATTGAGGC

Ref: TGATGAATTTTTCACCTGTA-CAAATGCTTTTCATGCAACATCTTGATCTATTGAGGC

**T1:**	Distribution of plants with different genotypes (M/N)
i1a: TGATGAATTTTTCACCTGTAACAAATGCTTTTCATGCAACATCTTGATCTATTGAGGC<br>i1b: TGATGAATTTTTCACCTGTACCAAATGCTTTTCATGCAACATCTTGATCTATTGAGGC<br>Ref: TGATGAATTTTTCACCTGTA-CAAATGCTTTTCATGCAACATCTTGATCTATTGAGGC<br>i1ai1b	(12/15)
i1a: TGATGAATTTTTCACCTGTAACAAATGCTTTTCATGCAACATCTTGATCTATTGAGGC<br>Ref: TGATGAATTTTTCACCTGTA-CAAATGCTTTTCATGCAACATCTTGATCTATTGAGGC<br>i1ai1a	(3/15)

***BnaA9.FUL, BnaFUL-sgRNA2*，T0-line15**

T0-leaf:

```
d1:  GGAAGGGGTAGGGTTCAGCT--AAGAGGATAGAGAAC
i1:  GGAAGGGGTAGGGTTCAGCTGTAAGAGGATAGAGA
Ref: GGAAGGGGTAGGGTTCAGCTG-AAGAGGATAGAGAACAAGATCAAT
```

T0-mixed tissues:

```
d1:  GGAAGGGGTAGGGTTCAGCT--AAGAGGATAGAGAAC
i1:  GGAAGGGGTAGGGTTCAGCTGTAAGAGGATAGAGA
Ref: GGAAGGGGTAGGGTTCAGCTG-AAGAGGATAGAGAACAAGATCAAT
```

T1: Distribution of plants with different genotypes (M/N)

```
d1:  GGAAGGGGTAGGGTTCAGCT--AAGAGGATAGAGAAC
i1:  GGAAGGGGTAGGGTTCAGCTGTAAGAGGATAGAGA
Ref: GGAAGGGGTAGGGTTCAGCTG-AAGAGGATAGAGAACAAGATCAAT
i1d1
```

(10/16)

```
i1:  GGAAGGGGTAGGGTTCAGCTGTAAGAGGATAGAGA
Ref: GGAAGGGGTAGGGTTCAGCTG-AAGAGGATAGAGAACAAGATCAAT
i1i1
```

(5/16)

```
d1:  GGAAGGGGTAGGGTTCAGCT--AAGAGGATAGAGAAC
Ref: GGAAGGGGTAGGGTTCAGCTG-AAGAGGATAGAGAACAAGATCAAT
d1d1
```

(1/16)

BnaC7.FUL, BnaFUL-sgRNA2**，T0-line23**

T0-leaf:

```
d5:  ATGGGAAGGGGTAGGGTTCA-------AGAGGATAGAAAACAAGA
i2:  ATGGGAAGGGGTAGGGTTCAGCTGAAAAGAGGATAGAAAACAAGATCAATAGGCAA
Ref: ATGGGAAGGGGTAGGGTTCAGCTG--AAGAGGATAGAAAACAAGATCAATAGGCAAGTTACTTTCTCTAAGA
```

T0-mixed tissues:

```
d5:  ATGGGAAGGGGTAGGGTTCA-------AGAGGATAGAAAACAAGA
i2:  ATGGGAAGGGGTAGGGTTCAGCTGAAAAGAGGATAGAAAACAAGATCAATAGGCAA
Ref: ATGGGAAGGGGTAGGGTTCAGCTG--AAGAGGATAGAAAACAAGATCAATAGGCAAGTTACTTTCTCTAAGA
```

T1: Distribution of plants with different genotypes (M/N)

```
d5:  ATGGGAAGGGGTAGGGTTCA-------AGAGGATAGAAAACAAGA
i2:  ATGGGAAGGGGTAGGGTTCAGCTGAAAAGAGGATAGAAAACAAGATCAATAGGCAA
Ref: ATGGGAAGGGGTAGGGTTCAGCTG--AAGAGGATAGAAAACAAGATCAATAGGCAAGTTACTTTCTCTAAGA
i2d5                                                                        (8/16)
```

```
i2:   ATGGGAAGGGGTAGGGTTCAGCTGAAAAGAGGATAGAAAACAAGATCAATAGGCAA
Ref:  ATGGGAAGGGGTAGGGTTCAGCTG--AAGAGGATAGAAAACAAGATCAATAGGCAAGTTACTTTCTCTAAGA
i2d2                                                                        (3/16)
```

```
d5:   ATGGGAAGGGGTAGGGTTCA-------AGAGGATAGAAAACAAGA
Ref:  ATGGGAAGGGGTAGGGTTCAGCTG--AAGAGGATAGAAAACAAGATCAATAGGCAAGTTACTTTCTCTAAGA
d5d5                                                                        (5/16)
```

BnaA6.RGA, BnaRGA-sgRNA1，**T0-line33**

T0-leaf:

```
i1a: CTACGAGACTTGCCCTTACCCTCAAGTTCGCTCACTTCACGGCGAATCAGGCGATTCTCGAGGCTTTCG
WT:  CTACGAGACTTGCCCTTA-CCTCAAGTTCGCTCACTTCACGGCGAATCAGGCGATTCTCGAGGCTTTCG
Ref: CTACGAGACTTGCCCTTA-CCTCAAGTTCGCTCACTTCACGGCGAATCAGGCGATTCTCGAGGCTTTCG
```

T0-mixed tissues:

```
i1a: CTACGAGACTTGCCCTTACCCTCAAGTTCGCTCACTTCACGGCGAATCAGGCGATTCTCGAGGCTTTCG
i1b: CTACGAGACTTGCCCTTAACCTCAAGTTCGCTCACTTCACGGCGAATCAGGCGATTCTCGAGGCTTTCG
WT:  CTACGAGACTTGCCCTTA-CCTCAAGTTCGCTCACTTCACGGCGAATCAGGCGATTCTCGAGGCTTTCG
Ref: CTACGAGACTTGCCCTTA-CCTCAAGTTCGCTCACTTCACGGCGAATCAGGCGATTCTCGAGGCTTTCG
```

T1: Distribution of plants with different genotypes (M/N)

```
i1a: CTACGAGACTTGCCCTTACCCTCAAGTTCGCTCACTTCACGGCGAATCAGGCGATTCTCGAGGCTTTCG
Ref: CTACGAGACTTGCCCTTA-CCTCAAGTTCGCTCACTTCACGGCGAATCAGGCGATTCTCGAGGCTTTCG
i1ai1a
```

(3/13)

```
i1a: CTACGAGACTTGCCCTTACCCTCAAGTTCGCTCACTTCACGGCGAATCAGGCGATTCTCGAGGCTTTCG
i1b: CTACGAGACTTGCCCTTAACCTCAAGTTCGCTCACTTCACGGCGAATCAGGCGATTCTCGAGGCTTTCG
Ref: CTACGAGACTTGCCCTTA-CCTCAAGTTCGCTCACTTCACGGCGAATCAGGCGATTCTCGAGGCTTTCG
i1ai1b
```

(7/13)

```
i1b: CTACGAGACTTGCCCTTAACCTCAAGTTCGCTCACTTCACGGCGAATCAGGCGATTCTCGAGGCTTTCG
Ref: CTACGAGACTTGCCCTTA-CCTCAAGTTCGCTCACTTCACGGCGAATCAGGCGATTCTCGAGGCTTTCG
i1bi1b
```

(2/13)

i1a: CTACGAGACTTGCCCTTACCCTCAAGTTCGCTCACTTCACGGCGAATCAGGCGATTCTCGAGGCTTTCG

WT: CTACGAGACTTGCCCTTA-CCTCAAGTTCGCTCACTTCACGGCGAATCAGGCGATTCTCGAGGCTTTCG

Ref: CTACGAGACTTGCCCTTA-CCTCAAGTTCGCTCACTTCACGGCGAATCAGGCGATTCTCGAGGCTTTCG

i1aWT (1/13)

BnaA9.RGA, BnaRGA-sgRNA1**，T0-line40**

T0-leaf:

d22: CTCCGACACGCTCCAGATGC-----------------------CCTCAAGTTCGCTCACTTCACGGCCAACCAAGC

WT: GCACTTCTACGAGACGTGCCCTTA-CCTCAAGTTCGCTCACTTCACGGCCAACCAAGCCATCCTCGAGGCTT

Ref: GCACTTCTACGAGACGTGCCCTTA-CCTCAAGTTCGCTCACTTCACGGCCAACCAAGCCATCCTCGAGGCTT

T0-mixed tissues:

d22: CTCCGACACGCTCCAGATGC-----------------------CCTCAAGTTCGCTCACTTCACGGCCAACCAAGC

i1: GCACTTCTACGAGACGTGCCCTTAACCTCAAGTTCGCTCACTTCACGGCCAACCAAGCCATCCTCGAGGCTT

WT: GCACTTCTACGAGACGTGCCCTTA-CCTCAAGTTCGCTCACTTCACGGCCAACCAAGCCATCCTCGAGGCTT

Ref: GCACTTCTACGAGACGTGCCCTTA-CCTCAAGTTCGCTCACTTCACGGCCAACCAAGCCATCCTCGAGGCTT

T1: Distribution of plants with different genotypes (M/N)

i1: GCACTTCTACGAGACGTGCCCTTAACCTCAAGTTCGCTCACTTCACGGCCAACCAAGCCATCCTCGAGGCTT

Ref: GCACTTCTACGAGACGTGCCCTTA-CCTCAAGTTCGCTCACTTCACGGCCAACCAAGCCATCCTCGAGGCTT

i1i1 (7/13)

d22: CTCCGACACGCTCCAGATGC-----------------------CCTCAAGTTCGCTCACTTCACGGCCAACCAAGC

Ref: GCACTTCTACGAGACGTGCCCTTA-CCTCAAGTTCGCTCACTTCACGGCCAACCAAGCCATCCTCGAGGCTT

d22d22 (2/13)

h: GCACTTCTACGAGACGTGCCCTTA

Ref: GCACTTCTACGAGACGTGCCCTTACCTCAAGTTCGCTCACTTCACGGCCAACCAAGCCATCCTCGAGGCTT

h (7/13)

BnaC7.RGA, BnaRGA-sgRNA1**，T0-line40**

T0-leaf:

i1: TGCACTTCTACGAGACGTGCCCTTACCCTCAAGTTCGCTCACTTCACGGCGAATCAAGCCAT

WT: TGCACTTCTACGAGACGTGCCCTTA-CCTCAAGTTCGCTCACTTCACGGCGAATCAAGCCAT

Ref: TGCACTTCTACGAGACGTGCCCTTA-CCTCAAGTTCGCTCACTTCACGGCGAATCAAGCCAT

T0-mixed tissues:

i1: TGCACTTCTACGAGACGTGCCCTTACCCTCAAGTTCGCTCACTTCACGGCGAATCAAGCCAT

WT: TGCACTTCTACGAGACGTGCCCTTA-CCTCAAGTTCGCTCACTTCACGGCGAATCAAGCCAT

Ref: TGCACTTCTACGAGACGTGCCCTTA-CCTCAAGTTCGCTCACTTCACGGCGAATCAAGCCAT

T1:	Distribution of plants with different genotypes (M/N)
i1a: TGCACTTCTACGAGACGTGCCCTTACCCTCAAGTTCGCTCACTTCACGGCGAATCAAGCCAT<br>Ref: TGCACTTCTACGAGACGTGCCCTTA-CCTCAAGTTCGCTCACTTCACGGCGAATCAAGCCAT<br>i1ai1a	(5/16)
i1b: TGCACTTCTACGAGACGTGCCCTTATCCTCAAGTTCGCTCACTTCACGGCGAATCAAGCCAT<br>Ref: TGCACTTCTACGAGACGTGCCCTTA-CCTCAAGTTCGCTCACTTCACGGCGAATCAAGCCAT<br>i1bi1b	(2/16)
i1a: TGCACTTCTACGAGACGTGCCCTTACCCTCAAGTTCGCTCACTTCACGGCGAATCAAGCCAT<br>i1b: TGCACTTCTACGAGACGTGCCCTTATCCTCAAGTTCGCTCACTTCACGGCGAATCAAGCCAT<br>Ref: TGCACTTCTACGAGACGTGCCCTTA-CCTCAAGTTCGCTCACTTCACGGCGAATCAAGCCAT<br>i1ai1b	(9/16)

***BnaA6.DA1, BnaA6.DA1-sgRNA2*，T0-line6**

T0-leaf:

```
WT:   TAAGTTCAACATATCCTGTA-TTGCGTGGCTGCACATAAACGAAAGCTGGCAAATTAGTATTGACTTTGGG
i1:   TAAGTTCAACATATCCTGTAATTGCGTGGCTGCACATAAACGAAAGCTGGCAAATTAGTATTGACTTTGGG
Ref:  TAAGTTCAACATATCCTGTA-TTGCGTGGCTGCACATAAACGAAAGCTGGCAAATTAGTATTGACTTTGGG
```

T0-mixed tissues:

```
WT:   TAAGTTCAACATATCCTGTA-TTGCGTGGCTGCACATAAACGAAAGCTGGCAAATTAGTATTGACTTTGGG
i1:   TAAGTTCAACATATCCTGTAATTGCGTGGCTGCACATAAACGAAAGCTGGCAAATTAGTATTGACTTTGGG
Ref:  TAAGTTCAACATATCCTGTA-TTGCGTGGCTGCACATAAACGAAAGCTGGCAAATTAGTATTGACTTTGGG
```

T1: Distribution of plants with different genotypes (M/N)

```
WT:   TAAGTTCAACATATCCTGTA-TTGCGTGGCTGCACATAAACGAAAGCTGGCAAATTAGTATTGACTTTGGG
i1:   TAAGTTCAACATATCCTGTAATTGCGTGGCTGCACATAAACGAAAGCTGGCAAATTAGTATTGACTTTGGG
Ref:  TAAGTTCAACATATCCTGTA-TTGCGTGGCTGCACATAAACGAAAGCTGGCAAATTAGTATTGACTTTGGG
i1WT                                                                          (10/15)

i1:   TAAGTTCAACATATCCTGTAATTGCGTGGCTGCACATAAACGAAAGCTGGCAAATTAGTATTGACTTTGGG
Ref:  TAAGTTCAACATATCCTGTA-TTGCGTGGCTGCACATAAACGAAAGCTGGCAAATTAGTATTGACTTTGGG
i1i1                                                                          (3/16)

WT:   TAAGTTCAACATATCCTGTA-TTGCGTGGCTGCACATAAACGAAAGCTGGCAAATTAGTATTGACTTTGGG
Ref:  TAAGTTCAACATATCCTGTA-TTGCGTGGCTGCACATAAACGAAAGCTGGCAAATTAGTATTGACTTTGGG
WTWT                                                                          (2/16)
```

***BnaA6.DA1, BnaA6.DA1-sgRNA2*，T0-line24**

T0-leaf:

WT: AAGTTCAACATATCCTGTATTGCGTGGCTGCACATAAACGAAAGCTGGCAAATTAGTATTGACTTTGGGA

d1: AAGTTCAACATATCCTGTA-TGCGTGGCTGCACATAAACGAAAGCTGGCAAATTAGTATTGACTTTGGGA

Ref: AAGTTCAACATATCCTGTATTGCGTGGCTGCACATAAACGAAAGCTGGCAAATTAGTATTGACTTTGGGA

T0-mixed tissues:

WT: AAGTTCAACATATCCTGTATTGCGTGGCTGCACATAAACGAAAGCTGGCAAATTAGTATTGACTTTGGGA

d1: AAGTTCAACATATCCTGTA-TGCGTGGCTGCACATAAACGAAAGCTGGCAAATTAGTATTGACTTTGGGA

Ref: AAGTTCAACATATCCTGTATTGCGTGGCTGCACATAAACGAAAGCTGGCAAATTAGTATTGACTTTGGGA

T1: Distribution of plants with different genotypes (M/N)

d1: AAGTTCAACATATCCTGTA-TGCGTGGCTGCACATAAACGAAAGCTGGCAAATTAGTATTGACTTTGGGA

Ref: AAGTTCAACATATCCTGTATTGCGTGGCTGCACATAAACGAAAGCTGGCAAATTAGTATTGACTTTGGGA

d1d1 (4/13)

WT: AAGTTCAACATATCCTGTATTGCGTGGCTGCACATAAACGAAAGCTGGCAAATTAGTATTGACTTTGGGA

d1: AAGTTCAACATATCCTGTA-TGCGTGGCTGCACATAAACGAAAGCTGGCAAATTAGTATTGACTTTGGGA

Ref: AAGTTCAACATATCCTGTATTGCGTGGCTGCACATAAACGAAAGCTGGCAAATTAGTATTGACTTTGGGA

d1WT (6/13)

WT: AAGTTCAACATATCCTGTATTGCGTGGCTGCACATAAACGAAAGCTGGCAAATTAGTATTGACTTTGGGA

Ref: AAGTTCAACATATCCTGTATTGCGTGGCTGCACATAAACGAAAGCTGGCAAATTAGTATTGACTTTGGGA

WTWT (3/13)

***BnaA2.DA2, BnaDA2-sgRNA2*，T0-line1**

T0-leaf:

WT: ATAGATATACTTCACCTGTA-CAAATGCTTTTCATGCAACATCTTGACCTATTGAGGCTAGGATAGTACTA

i1:   ATAGATATACTTCACCTGTAACAAATGCTTTTCATGCAACATCTTGACCTATTGAGGCTAGGATAGTACTA

Ref: ATAGATATACTTCACCTGTA-CAAATGCTTTTCATGCAACATCTTGACCTATTGAGGCTAGGATAGTACTA

T0-mixed tissues:

WT: ATAGATATACTTCACCTGTA-CAAATGCTTTTCATGCAACATCTTGACCTATTGAGGCTAGGATAGTACTA

i1a: ATAGATATACTTCACCTGTAACAAATGCTTTTCATGCAACATCTTGACCTATTGAGGCTAGGATAGTACTA

i1b: ATAGATATACTTCACCTGTATCAAATGCTTTTCATGCAACATCTTGACCTATTGAGGCTAGGATAGTACTA

Ref: ATAGATATACTTCACCTGTA-CAAATGCTTTTCATGCAACATCTTGACCTATTGAGGCTAGGATAGTACTA

T1: Distribution of plants with different genotypes (M/N)

i1a: ATAGATATACTTCACCTGTAACAAATGCTTTTCATGCAACATCTTGACCTATTGAGGCTAGGATAGTACTA

i1b: ATAGATATACTTCACCTGTATCAAATGCTTTTCATGCAACATCTTGACCTATTGAGGCTAGGATAGTACTA

Ref: ATAGATATACTTCACCTGTA-CAAATGCTTTTCATGCAACATCTTGACCTATTGAGGCTAGGATAGTACTA

i1ai1b (11/16)

WT: ATAGATATACTTCACCTGTA-CAAATGCTTTTCATGCAACATCTTGACCTATTGAGGCTAGGATAGTACTA

Ref: ATAGATATACTTCACCTGTA-CAAATGCTTTTCATGCAACATCTTGACCTATTGAGGCTAGGATAGTACTA

WTWT (5/16)

***BnaA2.DA2, BnaDA2-sgRNA2*，T0-line20**

T0-leaf:

```
WT:   ATAGATATACTTCACCTGTA-CAAATGCTTTTCATGCAACATCTTGACCTATTGAGGCTAGGATAGTACTA
i1a:  ATAGATATACTTCACCTGTAACAAATGCTTTTCATGCAACATCTTGACCTATTGAGGCTAGGATAGTACTA
Ref:  ATAGATATACTTCACCTGTA-CAAATGCTTTTCATGCAACATCTTGACCTATTGAGGCTAGGATAGTACTA
```

T0-mixed tissues:

```
WT:   ATAGATATACTTCACCTGTA-CAAATGCTTTTCATGCAACATCTTGACCTATTGAGGCTAGGATAGTACTA
i1a:  ATAGATATACTTCACCTGTAACAAATGCTTTTCATGCAACATCTTGACCTATTGAGGCTAGGATAGTACTA
i1b:  ATAGATATACTTCACCTGTATCAAATGCTTTTCATGCAACATCTTGACCTATTGAGGCTAGGATAGTACTA
Ref:  ATAGATATACTTCACCTGTA-CAAATGCTTTTCATGCAACATCTTGACCTATTGAGGCTAGGATAGTACTA
```

T1: Distribution of plants with different genotypes (M/N)

```
i1a:  ATAGATATACTTCACCTGTAACAAATGCTTTTCATGCAACATCTTGACCTATTGAGGCTAGGATAGTACTA
Ref:  ATAGATATACTTCACCTGTA-CAAATGCTTTTCATGCAACATCTTGACCTATTGAGGCTAGGATAGTACTA
i1ai1a                                                                          (3/15)

WT:   ATAGATATACTTCACCTGTA-CAAATGCTTTTCATGCAACATCTTGACCTATTGAGGCTAGGATAGTACTA
i1a:  ATAGATATACTTCACCTGTAACAAATGCTTTTCATGCAACATCTTGACCTATTGAGGCTAGGATAGTACTA
Ref:  ATAGATATACTTCACCTGTA-CAAATGCTTTTCATGCAACATCTTGACCTATTGAGGCTAGGATAGTACTA
i1aWT                                                                           (6/15)

i1a:  ATAGATATACTTCACCTGTAACAAATGCTTTTCATGCAACATCTTGACCTATTGAGGCTAGGATAGTACTA
i1b:  ATAGATATACTTCACCTGTATCAAATGCTTTTCATGCAACATCTTGACCTATTGAGGCTAGGATAGTACTA
Ref:  ATAGATATACTTCACCTGTA-CAAATGCTTTTCATGCAACATCTTGACCTATTGAGGCTAGGATAGTACTA
i1ai1b                                                                          (4/15)
```

WT: ATAGATATACTTCACCTGTACAAATGCTTTTCATGCAACATCTTGACCTATTGAGGCTAGGATAGTACTA

Ref: ATAGATATACTTCACCTGTACAAATGCTTTTCATGCAACATCTTGACCTATTGAGGCTAGGATAGTACTA

WTWT (2/15)

***BnaC6.DA2, BnaDA2-sgRNA2*，T0-line1**

T0-leaf:

```
WT:   TCGTAGTAACTGTCGCAGTT-GCCAGGAAGCATACT
i1:   TCGTAGTAACTGTCGCAGTTTGCCAGGAAGCATAC
Ref:  TCGTAGTAACTGTCGCAGTT-GCCAGGAAGCATACT
```

T0-mixed tissues:

```
WT:   TCGTAGTAACTGTCGCAGTT-GCCAGGAAGCATACT
i1:   TCGTAGTAACTGTCGCAGTTTGCCAGGAAGCATAC
Ref:  TCGTAGTAACTGTCGCAGTT-GCCAGGAAGCATACT
```

T1: Distribution of plants of different genotypes (M/N)

```
WT:    TCGTAGTAACTGTCGCAGTT-GCCAGGAAGCATACT
i1:    TCGTAGTAACTGTCGCAGTTTGCCAGGAAGCATAC
Ref:   TCGTAGTAACTGTCGCAGTT-GCCAGGAAGCATACT
i1WT                                          (10/16)
```

```
WT:    TCGTAGTAACTGTCGCAGTT-GCCAGGAAGCATACT
Ref:   TCGTAGTAACTGTCGCAGTT-GCCAGGAAGCATACT
WTWT                                          (6/13)
```

***BnaA6.RGA, BnaA6.RGA-sgRNA2*，T0-line4**

T0-leaf:

d5: TTCATTACAACCCCGCTGAGC-----TCGTGGCTTGATAACATGCTCACGGA

d6: TTCATTACAACCCCGCTGAG------TCGTGGCTTGATAACATGCTCACGGA

WT: TTCATTACAACCCCGCTGAGCTTTACTCGTGGCTTGATAACATGCTCACGGA

Ref: TTCATTACAACCCCGCTGAGCTTTACTCGTGGCTTGATAACATGCTCACGGA

T0-mixed tissues:

d5: TTCATTACAACCCCGCTGAGC-----TCGTGGCTTGATAACATGCTCACGGA

d6: TTCATTACAACCCCGCTGAG------TCGTGGCTTGATAACATGCTCACGGA

WT: TTCATTACAACCCCGCTGAGCTTTACTCGTGGCTTGATAACATGCTCACGGA

Ref: TTCATTACAACCCCGCTGAGCTTTACTCGTGGCTTGATAACATGCTCACGGA

T1: Distribution of plants with different genotypes (M/N)

d5: TTCATTACAACCCCGCTGAGC-----TCGTGGCTTGATAACATGCTCACGGA

Ref: TTCATTACAACCCCGCTGAGCTTTACTCGTGGCTTGATAACATGCTCACGGA

d5d5 (3/19)

h: TTCATTACAACCCCGCTGAG

Ref: TTCATTACAACCCCGCTGAGCTTTACTCGTGGCTTGATAACATGCTCACGGA

h (16/19)

***BnaC9.RGA, BnaRGA-sgRNA1*，T0-line27**

T0-leaf:

d5: CACTTCTACGAGACGTGCCCT-----CAAGTTCGCTCACTTCACGGCCAACCAAGCCATCCTCGAGGCTTT

d1: CACTTCTACGAGACGTGCCCTT-CCTCAAGTTCGCTCACTTCACGGCCAACCAAGCCATCCTCGAGGCTTT

d36: CACTTCTACGAGACGTGCCCTTACCT------------------------------------CGAGGCTTTCGAAGGGAAG

d33: CACTCTCCCTTCGAAAGCCC---------------------------------AGCCATCCTCGAGGCTTTCGAAGGGAAG

Ref: CACTTCTACGAGACGTGCCCTTACCTCAAGTTCGCTCACTTCACGGCCAACCAAGCCATCCTCGAAGACGTGGGGAA

T0-mixed tissues:

d5: CACTTCTACGAGACGTGCCCT-----CAAGTTCGCTCACTTCACGGCCAACCAAGCCATCCTCGAGGCTTT

d1: CACTTCTACGAGACGTGCCCTT-CCTCAAGTTCGCTCACTTCACGGCCAACCAAGCCATCCTCGAGGCTTT

d36: CACTTCTACGAGACGTGCCCTTACCT------------------------------------CGAGGCTTTCGAAGGGAAG

d33: CACTCTCCCTTCGAAAGCCC---------------------------------AGCCATCCTCGAGGCTTTCGAAGGGAAG

Ref: CACTTCTACGAGACGTGCCCTTACCTCAAGTTCGCTCACTTCACGGCCAACCAAGCCATCCTCGAAGACGTGGGGAA

T1: Distribution of plants with different genotypes (M/N)

d36: CACGCTCCAGATGCACTTCTACGAGACGTGCCCTTACCT------------------------------------CGAGGCTTTCGAAGGGAAG

h: CACGCTCCAGATGCACTTCTACGAGACGTGCCCTTACCT

Ref: CACGCTCCAGATGCACTTCTACGAGACGTGCCCTTACCTCAAGTTCGCTCACTTCACGGCCAACCAAGCCATCC

d36h (5/19)

d1: CACTTCTACGAGACGTGCCCTT-CCTCAAGTTCGCTCACTTCACGGCCAACCAAGCCATCCTCGAGGCTTT

h: CACGCTCCAGATGCACTTCTACGAGACGTGCCCTTACCT

Ref: CACGCTCCAGATGCACTTCTACGAGACGTGCCCTTACCTCAAGTTCGCTCACTTCACGGCCAACCAAGCCATCC

d1h (2/19)

```
d5:   CACTTCTACGAGACGTGCCCT-----CAAGTTCGCTCACTTCACGGCCAACCAAGCCATCCTCGAGGCTTT
h:    CACGCTCCAGATGCACTTCTACGAGACGTGCCCTTACCT
Ref:  CACGCTCCAGATGCACTTCTACGAGACGTGCCCTTACCTCAAGTTCGCTCACTTCACGGCCAACCAAGCCATCC
d5h                                                                              (10/19)

d33:  CACTCTCCCTTCGAAAGCCC---------------------------------AGCCATCCTCGAGGCTTTCGAAGGGAAG
h:    CACGCTCCAGATGCACTTCTACGAGACGTGCCCTTACCT
Ref:  CACGCTCCAGATGCACTTCTACGAGACGTGCCCTTACCTCAAGTTCGCTCACTTCACGGCCAACCAAGCCATCC
d33h                                                                             (2/19)
```

***BnaC9.RGA, BnaRGA-sgRNA1*，T0-line40**

T0-leaf:

```
i1:   CTACGAGACGTGCCCTTACCCTCAAGTTCGCTCACTTCACGGCCAACCAA
d5:   CTACGAGACGTGCCCTTA------GTTCGCTCACTTCACTTCACGGCCAACCAA
WT:   CTACGAGACGTGCCCTTA-CCTCAAGTTCGCTCACTTCACGGCCAACCAA
Ref:  CTACGAGACGTGCCCTTA-CCTCAAGTTCGCTCACTTCACGGCCAACCAA
```

T0-mixed tissues:

```
i1:   CTACGAGACGTGCCCTTACCCTCAAGTTCGCTCACTTCACGGCCAACCAA
d5:   CTACGAGACGTGCCCTTA------GTTCGCTCACTTCACTTCACGGCCAACCAA
WT:   CTACGAGACGTGCCCTTA-CCTCAAGTTCGCTCACTTCACGGCCAACCAA
d19:  CTACGAGACGTGCCCTTA--------------------ACG
Ref:  CTACGAGACGTGCCCTTA-CCTCAAGTTCGCTCACTTCACGGCCAACCAA
```

T1: Distribution of plants with different genotypes (M/N)

```
d19:  TCTACGAGACGTGCCCTTA--------------------ACG
h:    TCTACGAGACGTGCCCTTAGCC
Ref:  TCTACGAGACGTGCCCTTACCTCAAGTTCGCTCACTTCACGGCCAACCAAGCCATCCTCGAGGCTTTCG
d19h                                                                        (16/16)
```

***BnaC6.DA2, BnaDA2-sgRNA2*，T0-line12**

T0-leaf:

d4: CATACAGTATGCTTCCTGGC----GCGACAGTTACTACGACATAGAACAAGACGCAGA

h: CATACAGTATGCTTCCTGGC

Ref: CATACAGTATGCTTCCTGGCAACTGCGACAGTTACTACGACATAGAACAAGACGCAGATGACATTGACCA

T0-mixed tissues:

i1:   CTACGAGACGTGCCCTTACCCTCAAGTTCGCTCACTTCACGGCCAACCAA

d5:   CTACGAGACGTGCCCTTA------GTTCGCTCACTTCACTTCACGGCCAACCAA

WT: CTACGAGACGTGCCCTTA-CCTCAAGTTCGCTCACTTCACGGCCAACCAA

d19: CTACGAGACGTGCCCTTA-------------------ACG

Ref: CTACGAGACGTGCCCTTA-CCTCAAGTTCGCTCACTTCACGGCCAACCAA

**T1:**	Distribution of plants with different genotypes (M/N)
d19: TCTACGAGACGTGCCCTTA-------------------ACG	
h: TCTACGAGACGTGCCCTTAGCC	
Ref: TCTACGAGACGTGCCCTTACCTCAAGTTCGCTCACTTCACGGCCAACCAAGCCATCCTCGAGGCTTTCG	
d19h	(16/16)

***BnaA9.RGA, BnaRGA-sgRNA1*，T0-line43**

T0-leaf:

WT: CTCCGACACGCTCCAGATGCACTTCTACGAGACGTGCCCTTACCTCAAGTTCGCTCACTTCACGGCCAAC

Ref: CTCCGACACGCTCCAGATGCACTTCTACGAGACGTGCCCTTACCTCAAGTTCGCTCACTTCACGGCCAAC

T0-mixed tissues:

WT: CTCCGACACGCTCCAGATGCACTTCTACGAGACGTGCCCTTACCTCAAGTTCGCTCACTTCACGGCCAAC

Ref: CTCCGACACGCTCCAGATGCACTTCTACGAGACGTGCCCTTACCTCAAGTTCGCTCACTTCACGGCCAAC

T1: Distribution of plants with different genotypes (M/N)

WT: CTCCGACACGCTCCAGATGCACTTCTACGAGACGTGCCCTTACCTCAAGTTCGCTCACTTCACGGCCAAC

Ref: CTCCGACACGCTCCAGATGCACTTCTACGAGACGTGCCCTTACCTCAAGTTCGCTCACTTCACGGCCAAC

WTWT (10/10)

***BnaA6.DA1, BnaA6.DA1-sgRNA2*，T0-line15**

T0-leaf:

WT: CTAATTTGCCAGCTTTCGTTTATGTGCAGCCACGCAATACAGGATATGTTGAACTTAACGATGGACGG

Ref: CTAATTTGCCAGCTTTCGTTTATGTGCAGCCACGCAATACAGGATATGTTGAACTTAACGATGGACGG

T0-mixed tissues:

WT: CTAATTTGCCAGCTTTCGTTTATGTGCAGCCACGCAATACAGGATATGTTGAACTTAACGATGGACGG

Ref: CTAATTTGCCAGCTTTCGTTTATGTGCAGCCACGCAATACAGGATATGTTGAACTTAACGATGGACGG

T1: Distribution of plants with different genotypes (M/N)

WT: CTAATTTGCCAGCTTTCGTTTATGTGCAGCCACGCAATACAGGATATGTTGAACTTAACGATGGACGG

Ref: CTAATTTGCCAGCTTTCGTTTATGTGCAGCCACGCAATACAGGATATGTTGAACTTAACGATGGACGG

WTWT (10/10)

***BnaA6.DA1*, *BnaA6.DA1-sgRNA2*，T0-line25**

T0-leaf:

WT: CTAATTTGCCAGCTTTCGTTTATGTGCAGCCACGCAATACAGGATATGTTGAACTTAACGATGGACGGA

Ref: CTAATTTGCCAGCTTTCGTTTATGTGCAGCCACGCAATACAGGATATGTTGAACTTAACGATGGACGGA

T0-mixed tissues:

WT: CTAATTTGCCAGCTTTCGTTTATGTGCAGCCACGCAATACAGGATATGTTGAACTTAACGATGGACGGA

Ref: CTAATTTGCCAGCTTTCGTTTATGTGCAGCCACGCAATACAGGATATGTTGAACTTAACGATGGACGGA

T1: Distribution of plants with different genotypes (M/N)

WT: CTAATTTGCCAGCTTTCGTTTATGTGCAGCCACGCAATACAGGATATGTTGAACTTAACGATGGACGGA

Ref: CTAATTTGCCAGCTTTCGTTTATGTGCAGCCACGCAATACAGGATATGTTGAACTTAACGATGGACGGA

WTWT (10/10)

***BnaC9.RGA, BnaRGA-sgRNA1*，T0-line46**

T0-leaf:

```
i2:   AGATGCACTTCTACGAGACGTGCCCTTAATCCTCAAGTTCGCTCACTTCACGGCCAACCAAGCCATCC
c3:   AGATGCACTTCTACGAGACGTGCCCTTA-----AAAGTTCGCTCACTTCACGGCCAACCAAGCCATCCT
d4:   AGATGCACTTCTACGAGACGTGCCCTTA------AAGTTCGCTCACTTCACGGCCAACCAAGCCATCCTCG
Ref: AGATGCACTTCTACGAGACGTGCCCTTA--CCTCAAGTTCGCTCACTTCACGGCCAACCAAGCCATCCTCG
```

T0-mixed tissues:

```
i2:   AGATGCACTTCTACGAGACGTGCCCTTAATCCTCAAGTTCGCTCACTTCACGGCCAACCAAGCCATCC
c3:   AGATGCACTTCTACGAGACGTGCCCTTA-----AAAGTTCGCTCACTTCACGGCCAACCAAGCCATCCT
d4:   AGATGCACTTCTACGAGACGTGCCCTTA------AAGTTCGCTCACTTCACGGCCAACCAAGCCATCCTCG
Ref: AGATGCACTTCTACGAGACGTGCCCTTA--CCTCAAGTTCGCTCACTTCACGGCCAACCAAGCCATCCTCG
```

***BnaC9.RGA, BnaRGA-sgRNA1*，T0-line29**

T0-leaf:

```
i1:   CAGATGCACTTCTACGAGACGTGCCCTTAACCTCAAGTTCGCTCACTTCACGGCCAACCAA
c6:   CAGATGCACTTCTACGAGACGTGCCCTTA------TTTCGCTCACTTCACGGCCAACCAA
d6:   CAGATGCACTTCTACGAGACGTGCCCTTA-------TTCGCTCACTTCACGGCCAACCAA
c9:   CAGAGGCACATCTATGAGACGTGCC-------CCAA-TTCGCTCACTTCACGGCCAACCAA
Ref:  CAGATGCACTTCTACGAGACGTGCCCTTA-CCTCAAGTTCGCTCACTTCACGGCCAACCAA
```

T0-mixed tissues:

```
i1:   CAGATGCACTTCTACGAGACGTGCCCTTAACCTCAAGTTCGCTCACTTCACGGCCAACCAA
c6:   CAGATGCACTTCTACGAGACGTGCCCTTA------TTTCGCTCACTTCACGGCCAACCAA
d6:   CAGATGCACTTCTACGAGACGTGCCCTTA-------TTCGCTCACTTCACGGCCAACCAA
c9:   CAGAGGCACATCTATGAGACGTGCC-------CCAA-TTCGCTCACTTCACGGCCAACCAA
Ref:  CAGATGCACTTCTACGAGACGTGCCCTTA-CCTCAAGTTCGCTCACTTCACGGCCAACCAA
```

Supplemental Table S4. Genotypes in leaves and mixed tissues (leaf, stem, and flower bud) among the T0 plants.

				**Genotype**	
**Target gene**	**sgRNA**	**Line**	**Zygosity◐**	**Leaf**	**Mixed tissues**
BnaA6.RGA	BnaRGA-sgRNA1	T0-40	Homozygote	d1d1	d1d1
BnaA6.RGA	BnaRGA-sgRNA1	T0-44	Homozygote	i1i1	i1i1
BnaA9.RGA	BnaRGA-sgRNA1	T0-44	Homozygote	d6d6	d6d6
BnaA6.RGA	BnaA6.RGA-sgRNA1	T0-5	Bi-allele	d5,d6	d5,d6
BnaA9.RGA	BnaRGA-sgRNA1	T0-27	Bi-allele	i1,d5	i1,d5
BnaA6.RGA	BnaRGA-sgRNA1	T0-46	Bi-allele	i1,d5	i1,d5
BnaA6.RGA	BnaA6.RGA-sgRNA1	T0-6	Bi-allele	d5,d9	d5,d9
BnaC7.RGA	BnaRGA-sgRNA1	T0-38	Bi-allele	i1,d2	i1,d2
BnaC5.DA1	BnaC5.DA1-sgRNA1	T0-16	Bi-allele	i1a,i1b	i1a,i1b
BnaA6.RGA	BnaRGA-sgRNA1	T0-33	Heterozygote	i1,WT	i1a,i1b,WT
BnaA6.DA1	BnaA6.DA1-sgRNA2	T0-6	Heterozygote	i1,WT	i1,WT
BnaA6.DA1	BnaA6.DA1-sgRNA2	T0-24	Heterozygote	d1,WT	d1,WT
BnaC7.RGA	BnaRGA-sgRNA1	T0-40	Heterozygote	i1a,WT	i1a,WT
BnaA9.RGA	BnaRGA-sgRNA1	T0-40	Heterozygote	d22,WT	d22, i1,WT
BnaA2.DA2	BnaDA2-sgRNA2	T0-20	Heterozygote	i1, WT	i1a, i1b, WT
BnaC9.RGA	BnaRGA-sgRNA1	T0-29	Chimera	i1, d6, c6, c9	i1, d6, c6, c9
BnaC9.RGA	BnaRGA-sgRNA1	T0-40	Chimera	i1,d5, WT	i1,d5, d19, WT
BnaC9.RGA	BnaRGA-sgRNA1	T0-27	Chimera	d1,d5,d33,d36	d1,d5,d33,d36
BnaC9.RGA	BnaRGA-sgRNA1	T0-46	Chimera	i2, d4, c4	i2, d4, c4
BnaA6.RGA	BnaRGA-sgRNA1	T0-4	Chimera	d7,d5,WT	d7,d5,WT
BnaA9.RGA	BnaRGA-sgRNA1	T0-43	WT◊	WT	WT

◐: the zygosity of the homozygote, bi-allele, and heterozygote in the T0 plants. ◇: WT, no mutations were identified.

d number indicates the number of bp deleted from a target site. d number a indicates the same number of bp were deleted at one site. d number b indicates the same number of bp were deleted at other sites. i number indicates the number of bp inserted at a target site. i number a indicates the same number of insertions at one site. i number b indicates the same number of different nucleotides were inserted at the same site. c number indicates a combined mutation. h indicates heterogeneous (i.e., more than one sequence detected in the sample).

Supplemental Table S5. Percentage of T1 plants with or without the Cas9 transgene.

Target Gene	Line	No. of plants examined	Cas9 positive plants	Cas9 positive rate	Cas9 negative plants
BnaRGA	L27	30	26	86.70%	1, 4, 5, 12
BnaRGA	L33	30	29	96.70%	20
BnaRGA	L38	28	25	89.30%	13, 25, 26
BnaRGA	L40	38	37	97.40%	14
BnaRGA	L44	30	26	86.67%	8, 24, 27, 30
BnaRGA	L46	30	28	93.30%	11, 13
BnaA6.RGA	L4	24	11	45.80%	8, 13-24
BnaA6.RGA	L5	30	19	63.30%	4, 5, 9, 11, 13, 16, 20, 22, 23, 27, 30
BnaA6.RGA	L6	10	8	80.00%	8, 9
BnaA6.DA1	L6	30	30	100%	
BnaA6.DA1	L15	29	28	96.60%	9
BnaA6.DA1	L24	34	34	100%	
BnaDA2	L1	25	19	76%	1, 2, 11, 14, 20, 24
BnaDA2	L12	19	19	100%	
BnaDA2	L20	26	24	92.3%	4, 14
BnaC5.DA1	L1	30	30	100.0%	
BnaFUL	L2	8	7	87.5%	6
BnaFUL	L12	12	9	75.0%	4, 5, 11
BnaFUL	L15	29	26	89.7%	3, 11, 23
BnaFUL	L16	19	19	100.0%	
BnaFUL	L23	19	18	94.7%	6
Total		530	471	88.14%	58 (10.9%)

Supplemental Table S6: The list of primers used in this study.

Primer name	Primer sequence (5' to 3')	Purpose of the primers
BnC5.DA1-DT1-BsF	ATATATGGTCTCGATTGGATGGACGGAAACTTTGCCGTT	Making BnaC5.DA1 CRISPR construct
BnC5.DA1-DT1-F0	TGGATGGACGGAAACTTTGCCGTTTTAGAGCTAGAAATAGC	Making BnaC5.DA1 CRISPR construct
BnC5.DA1-DT2-R0	AACGCACAAATCCTAAGGTGTTCAATCTCTTAGTCGACTCTAC	Making BnaC5.DA1 CRISPR construct
BnC5.DA1-DT2-BsR	ATTATTGGTCTCGAAACGCACAAATCCTAAGGTGTTCAA	Making BnaC5.DA1 CRISPR construct
BnA6.DA1-DT1-BsF	ATATATGGTCTCGATTGATGTGCAGCCACGCAATACGTT	Making BnaA6.DA1 CRISPR construct
BnA6.DA1-DT1-F0	TGATGTGCAGCCACGCAATACGTTTTAGAGCTAGAAATAGC	Making BnaA6.DA1 CRISPR construct
BnA6.DA1-DT2-R0	AACACCCATAGTAACCTCTCTTCAATCTCTTAGTCGACTCTAC	Making BnaA6.DA1 CRISPR construct
BnA6.DA1-DT2-BsR	ATTATTGGTCTCGAAACACCCATAGTAACCTCTCTTCAA	Making BnaA6.DA1 CRISPR construct
BnA2.DA2-DT1-BsF	ATATATGGTCTCGATTGGGAGCTAAGTACGTAGACTGTT	Making BnaA2.DA2 CRISPR construct
BnA2.DA2-DT1-F0	TGGGAGCTAAGTACGTAGACTGTTTTAGAGCTAGAAATAGC	Making BnaA2.DA2 CRISPR construct
BnA2.DA2-DT2-R0	AACGCAATGCCGTCGTCTTGTGCAATCTCTTAGTCGACTCTAC	Making BnaA2.DA2 CRISPR construct
BnA2.DA2-DT2-BsR	ATTATTGGTCTCGAAACGCAATGCCGTCGTCTTGTGCAA	Making BnaA2.DA2 CRISPR construct
BnC7.RGA-DT1-BsF	ATATATGGTCTCGATTGCCCTGCTGAGCTCTACTCTGTT	Making BnaC7.RGA CRISPR construct
BnC7.RGA-DT1-F0	TGCCCTGCTGAGCTCTACTCTGTTTTAGAGCTAGAAATAGC	Making BnaC7.RGA CRISPR construct
BnC7.RGA-DT2-R0	AACCAGCCATCTCCGACGACCTCAATCTCTTAGTCGACTCTAC	Making BnaC7.RGA CRISPR construct
BnC7.RGA-DT2-BsR	ATTATTGGTCTCGAAACCAGCCATCTCCGACGACCTCAA	Making BnaC7.RGA CRISPR construct
BnA9.RGA-DT1-BsF	ATATATGGTCTCGATTGAGGTCGTCCGAGATGGCCGGTT	Making BnaA9.RGA CRISPR construct
BnA9.RGA-DT1-F0	TGAGGTCGTCCGAGATGGCCGGTTTTAGAGCTAGAAATAGC	Making BnaA9.RGA CRISPR construct
BnA9.RGA-DT2-R0	AACCGAGTAGAGCTCCGACGGGCAATCTCTTAGTCGACTCTAC	Making BnaA9.RGA CRISPR construct
BnA9.RGA-DT2-BsR	ATTATTGGTCTCGAAACCGAGTAGAGCTCCGACGGGCAA	Making BnaA9.RGA CRISPR construct

Primer name	Primer sequence (5'-3')	Purpose of the primers
BnC9.RGA-DT1-BsF	ATATATGGTCTCGATTGCCCGTCGGAGCTTTACTCGGTT	Making BnaC9.RGA CRISPR construct
BnC9.RGA-DT1-F0	TGCCCGTCGGAGCTTTACTCGGTTTTAGAGCTAGAAATAGC	Making BnaC9.RGA CRISPR construct
BnC9.RGA-DT2-R0	AACTCTCTGACGACCTCACCTTCAATCTCTTAGTCGACTCTAC	Making BnaC9.RGA CRISPR construct
BnC9.RGA-DT2-BsR	ATTATTGGTCTCGAAACTCTCTGACGACCTCACCTTCAA	Making BnaC9.RGA CRISPR construct
BnA6.RGA-DT1-BsF	ATATATGGTCTCGATTGCCCCGCTGAGCTTTACTCGGTT	Making BnaA6.RGA CRISPR construct
BnA6.RGA-DT1-F0	TGCCCCGCTGAGCTTTACTCGGTTTTAGAGCTAGAAATAGC	Making BnaA6.RGA CRISPR construct
BnA6.RGA-DT2-R0	AACTCTCCGAAGACCTAACCTTCAATCTCTTAGTCGACTCTAC	Making BnaA6.RGA CRISPR construct
BnA6.RGA-DT2-BsR	ATTATTGGTCTCGAAACTCTCCGAAGACCTAACCTTCA	Making BnaA6.RGA CRISPR construct
BnRGA-DT1-BsF	ATATATGGTCTCGATTGGTGAGCGAACTTGAGGTAAGTT	Making RGA homolog CRISPR construct
BnRGA-DT1-F0	TGGTGAGCGAACTTGAGGTAAGTTTTAGAGCTAGAAATAGC	Making RGA homolog CRISPR construct
BnRGA-DT2-R0	AACTGAAAATCACCGGTTTAATCAATCTCTTAGTCGACTCTAC	Making RGA homolog CRISPR construct
BnRGA-DT2-BsR	ATTATTGGTCTCGAAACTGAAAATCACCGGTTTAATCAA	Making RGA homolog CRISPR construct
BnDA2-DT1-BsF	ATATATGGTCTCGATTGAGTAACTGTCGCAGTTGCCGTT	Making DA2 homolog CRISPR construct
BnDA2-DT1-F0	TGAGTAACTGTCGCAGTTGCCGTTTTAGAGCTAGAAATAGC	Making DA2 homolog CRISPR construct
BnDA2-DT2-R0	AACGTACAAATGCTTTTCATGCCAATCTCTTAGTCGACTCTAC	Making DA2 homolog CRISPR construct
BnDA2-DT2-BsR	ATTATTGGTCTCGAAACGTACAAATGCTTTTCATGCCAA	Making DA2 homolog CRISPR construct
BnFUL-DT1-BsF	ATATATGGTCTCGATTGTATTCGAAGAGTTTGCCTTGTT	Making FUL homolog CRISPR construct
BnFUL-DT1-F0	TGTATTCGAAGAGTTTGCCTTGTTTTAGAGCTAGAAATAGC	Making FUL homolog CRISPR construct
BnFUL-DT2-R0	AACCTTCAGCTGAACCCTACCCCAATCTCTTAGTCGACTCTAC	Making FUL homolog CRISPR construct
BnFUL-DT2-BsR	ATTATTGGTCTCGAAACCTTCAGCTGAACCCTACCCCAA	Making FUL homolog CRISPR construct
BnA9.RGA-H-F	GGCCTCGCAGCTGAGTCAACTCG	Checking BnRGA gene editing for A9
BnC9.RGA-H-F	ACAACTACTGAGTCAACTCGGTCCATGGTCC	Checking BnRGA gene editing for C9
BnC7.RGA-H-F	AATCAGATTCTTCCTTCCCCGG	Checking BnRGA gene editing for C7

Primer name	Primer sequence (5’-3’)	Purpose of the primers
BnA6.RGA-H-F	CAACGACCGGATCTAACGCTTTG	Checking BnRGA gene editing for A6
BnRGA-H-R	GATTGCAAATCTGTTTCCCTAA	Checking BnRGA gene editing
BnA9.RGA-S-F	TCCAAGATCAGAGCCCAAA	Checking BnaA9.RGA gene editing
BnA9.RGA-S-R	GAGGCCTACAGACCGAGTT	Checking BnaA9.RGA gene editing
BnC9.RGA-S-F	GAAAGCAAACCCTAGATCCAAGATC	Checking BnaC9.RGA gene editing
BnC9.RGA-S-R	GGACCGAGTTGACTCAGTAGTTGT	Checking BnaC9.RGA gene editing
BnA6.RGA-S-F	TCGCAGTGAAAAACAAAGCAATCC	Checking BnaA6.RGA gene editing
BnA6.RGA-S-R	GGTTCAAAGCGTTAGATCCGGTCG	Checking BnaA6.RGA gene editing
BnC7.RGA-S-F	TGAAGTGGTAGTCGCAGTGAA	Checking BnaC7.RGA gene editing
BnC7.RGA-S-R	TCTGATTCACACCACTAGACCCGG	Checking BnaC7.RGA gene editing
BnFUL-A9-F	AGGGTTGTCATTTCTCTCTCATGTTCTTGAAGA	Checking BnFUL gene editing for A9
BnFUL-A9-R	GTATATGATCGTACAAACAAATATATGTACACTTTA	Checking BnFUL gene editing for A9
BnFUL-C2-F	TTCTCTCTCTTGTTCTTGAGGTTTT	Checking BnFUL gene editing for C2
BnFUL-C2-R	CTTCACCCTAATAACCTCAATTAATC	Checking BnFUL gene editing for C2
BnFUL-C7-F	AAGGTTGTCGTTTCTCTCTCTTTTTCTTGAGCTTTT	Checking BnFUL gene editing for C7
BnFUL-C7-R	CAAGCAACACATATATAAACATACAAAAGCGCATTT	Checking BnFUL gene editing for C7
BnC5.DA1-F1	ATACACCGCGTCAGAAGCA	Checking BnaC5.DA1 gene editing
BnC5.DA1-R1	GAATCTGTCACAACAAATTTACATTCGA	Checking BnaC5.DA1 gene editing
BnC5.DA1-F2	TCGAATGTAAATTTGTTGTGACAGATTC	Checking BnaC5.DA1 gene editing
BnC5.DA1-R2	TGTGTTTTTCTTTTTGCTACTCACA	Checking BnaC5.DA1 gene editing
BnA6.DA1-F1	CAGAGATCCTACATTGAACTATGAGCAG	Checking BnaA6.DA1 gene editing
BnA6.DA1-R1	TGGCTTCGTTGAGTGCTTGC	Checking BnaA6.DA1 gene editing
BnA6.DA1-F2	CTCACCAACAACGTGTCACTAC	Checking BnaA6.DA1 gene editing

**Primer name**	**Primer sequence (5'-3')**	**Purpose of the primers**
BnA6.DA1-R2	GTAGTGACACGTTGTTGGTGAG	Checking BnaA6.DA1 gene editing
BnA2.DA2-H-F1	CCAGCAATGCCGTCGTCTTGTG	Checking BnaDA2 gene editing for A2
BnA2.DA2-H-R1	GAAGCTTTTGAACACAACCCAACAATTTTC	Checking BnaDA2 gene editing for A2
BnA2.DA2-H-F2	GCAATCTCATTCATTGTAGATCTAACTCTG	Checking BnaDA2 gene editing for A2
BnA2.DA2-H-R2	CATCAACCTTGACTGAGTAAAGGTAACA	Checking BnaDA2 gene editing for A2
BnC6.DA2-H-F1	CATCATCTGGTGGACTTGCT	Checking BnaDA2 gene editing for C6
BnC6.DA2-H-R1	CTTGTCTATGACCAAACAAAAACACAC	Checking BnaDA2 gene editing for C6
BnC6.DA2-H-F2	TTCTAGTGTCATTGTTGTGGACCTAAC	Checking BnaDA2 gene editing for C6
BnC6.DA2-H-R2	GAGCCTAACTAGCGTTTCTTGAAAC	Checking BnaDA2 gene editing for C6
BnA2.DA2-S-F1	TGTCTTTCTACTTGGCTGC	Checking BnaA2.DA2 gene editing
BnA2.DA2-S-R1	GGAGAATTGATATTTTTTTCATTTCATAAGAGGTA	Checking BnaA2.DA2 gene editing
BnA2.DA2-S-F2	GAGGTGATTCAGTATTGGACTT	Checking BnaA2.DA2 gene editing
BnA2.DA2-S-R2	TCATGTACGAGCTTACATAGCT	Checking BnaA2.DA2 gene editing
BnA6/C7-OFF-F	ATGATGATGCTGAAGGAAGAAGA	Checking BnRGA off-target
BnA6/C7-OFF-C9R	TCCCTCTCGGAAAATCTCTACATCT	Checking BnRGA off-target
BnA6/C7-OFF-C7R	TCTCGGCAGATCCGGTAAAA	Checking BnRGA off-target
BnA9.RGA-S-F	TCCAAGATCAGAGCCCAAA	Checking BnRGA off-target
BnA9.RGA-S-R	GAGGCCTACAGACCGAGTT	Checking BnRGA off-target
BnC9.RGA-S-F	GAAAGCAAACCCTAGATCCAAGATC	Checking BnRGA off-target
BnC9.RGA-S-R	GGACCGAGTTGACTCAGTAGTTGT	Checking BnRGA off-target
BnA6.RGA-S-F	TCGCAGTGAAAAACAAAGCAATCC	Checking BnRGA off-target
BnA6.RGA-S-R	GGTTCAAAGCGTTAGATCCGGTCG	Checking BnRGA off-target
BnC7.RGA-S-F	TGAAGTGGTAGTCGCAGTGAA	Checking BnRGA off-target

Primer name	Primer sequence (5’-3’)	Purpose of the primers
BnC7.RGA-S-R	TCTGATTCACACCACTAGACCCGG	Checking BnRGA off-target
Cas9-570-F	AGACCGTGAAGGTTGTGGAC	Checking the positive transgenic lines
Cas9-570-R	TAGTGATCTGCCGTGTCTCG	Checking the positive transgenic lines
Actin-1F	ATGTGATGTGGATATCAGGAAGGAT	Internal control
Actin-1R	ACGGTCCAGATTCGTCATACTCA	Internal control
NPTII-F	ATGGGGATTGAACAAGATGGAT	Checking NPTII gene in transgenic lines
NPTII-R	CAGAAGAACTCGTCAAGAAGGCG	Checking NPTII gene in transgenic lines
401-F	TGTCCCAGGATTAGAATGATTAGGC	Checking pKSE401 fragment in transgenic lines
401-R	GTCAGGCTGCAGTAGTTTCCATTAA	Checking pKSE401 fragment in transgenic lines

Supplemental Data S1. Decoding results from T0 plants.

In the T0 generation, DNA was extracted from leaves. The DNA sequences around the target sites were amplified using PCR and then sequenced. The sequencing results were decoded using the following website: http://dsdecode.scgene.com/. For the PCR amplicons that contained mixed sequences that could not be decoded, the DNA was cloned into a TA vector, and each clone was individually sequenced. The genotype could be defined as homozygous, heterozygous, bi-allele, chimera mutants or WT. Allele number and the genotype were identified. # indicates the number of genotypes that were identified. WT indicates a wild-type sequence with no mutation detected. Deletion indicates a deletion mutation was detected. Insertion indicates that an insertion mutation was detected. Substitution indicates that a substitution mutation was detected. Complicated variant indicates combined mutations with more than two different genotypes that could not be identified. Ref indicates the reference sequence. The names of the transgenic lines are indicated with a bold font. The sgRNA targets are indicated with red. The mutations are indicated with yellow. The PAM motif (NGG) is indicated with a grey box.

BnaA9.RGA

L1 *BnaA9.RGA*-sgRNA1

Allele: TGTTGGGTTACAAGGTGAGGTCGTCCGAGATGGCCGAGGTTGCGTTGAAGCTAGAGCAGTTAGAGACCATGATGGG (WT)

Ref: TGTTGGGTTACAAGGTGAGGTCGTCCGAGATGGCCGAGGTTGCGTTGAAGCTAGAGCAGTTAGAGACCATGATGGG

L2 *BnaA9.RGA*-sgRNA1

Allele1: TGGGTTACAAGGTGAGGTCGTCCGAGATGG-CCGAGGTTGCGTTGAAGCTAGAGCAGTTAGAGACCATGATGG (WT)

Allele2: TGGGTTACAAGGTGAGGTCGTCCGAGATGGACCGAGGTTGCGTTGAAGCTAGAGCAGTTAGAGACCATGATGGG (insertion)

Ref: TGGGTTACAAGGTGAGGTCGTCCGAGATGG-CCGAGGTTGCGTTGAAGCTAGAGCAGTTAGAGACCATGATGGGT

L3 *BnaA9.RGA*-sgRNA1

Allele1: TGGGTTACAAGGTGAGGTCGTCCGAGATGG-CCGAGGTTGCGTTGAAGCTAGAGCAGTTAGAGACCATGATGG (WT)

Allele2: TGGGTTACAAGGTGAGGTCGTCCGAGATGGACCGAGGTTGCGTTGAAGCTAGAGCAGTTAGAGACCATGATGGG (insertion)

Ref: TGGGTTACAAGGTGAGGTCGTCCGAGATGG-CCGAGGTTGCGTTGAAGCTAGAGCAGTTAGAGACCATGATGGGT

L4 *BnaA9.RGA*-sgRNA1

Allele1: TGGGTTACAAGGTGAGGTCGTCCGAGATGG-CCGAGGTTGCGTTGAAGCTAGAGCAGTTAGAGACCATGATGG (WT)

Allele2: TGGGTTACAAGGTGAGGTCGTCCGAGATGGACCGAGGTTGCGTTGAAGCTAGAGCAGTTAGAGACCATGATGGG (insertion)

Ref: TGGGTTACAAGGTGAGGTCGTCCGAGATGG-CCGAGGTTGCGTTGAAGCTAGAGCAGTTAGAGACCATGATGGGT

L5 *BnaA9.RGA*-sgRNA1

Allele1: TGGGTTACAAGGTGAGGTCGTCCGAGATGG-CCGAGGTTGCGTTGAAGCTAGAGCAGTTAGAGACCATGATGG (WT)

Allele2: TGGGTTACAAGGTGAGGTCGTCCGAGATGGACCGAGGTTGCGTTGAAGCTAGAGCAGTTAGAGACCATGATGGG (insertion)

Ref: TGGGTTACAAGGTGAGGTCGTCCGAGATGG-CCGAGGTTGCGTTGAAGCTAGAGCAGTTAGAGACCATGATGGGT

L6 *BnaA9.RGA*-sgRNA1

Allele1: TGTTGGGTTACAAGGTGAGGTCGTCCGAGATGGCCGAGGTTGCGTTGAAGCTAGAGCAGTTAGAGACCATGATGGG (WT)

Allele2: TGTTGGGTTACAAGGTGAGGTCGTCCGAGATGGACCAAGG (complicated variant)

Ref: TGTTGGGTTACAAGGTGAGGTCGTCCGAGATGGCCGAGGTTGCGTTGAAGCTAGAGCAGTTAGAGACCATGATGGG

L7 *BnaA9.RGA*-sgRNA1

Allele1: TGGGTTACAAGGTGAGGTCGTCCGAGATGG-CCGAGGTTGCGTTGAAGCTAGAGCAGTTAGAGACCATGATGG (WT)

Allele2: TGGGTTACAAGGTGAGGTCGTCCGAGATGGACCGAGGTTGCGTTGAAGCTAGAGCAGTTAGAGACCATGATGGG (insertion)

Ref: TGGGTTACAAGGTGAGGTCGTCCGAGATGG-CCGAGGTTGCGTTGAAGCTAGAGCAGTTAGAGACCATGATGGGT

L8 *BnaA9.RGA*-sgRNA1

Allele: TGTTGGGTTACAAGGTGAGGTCGTCCGAGATGGCCGAGGTTGCGTTGAAGCTAGAGCAGTTAGAGACCATGATGGG (WT)

Ref: TGTTGGGTTACAAGGTGAGGTCGTCCGAGATGGCCGAGGTTGCGTTGAAGCTAGAGCAGTTAGAGACCATGATGGG

L9 *BnaA9.RGA*-sgRNA1

Allele1: TGTTGGGTTACAAGGTGAGGTCGTCCGAGATGGCCGAGGTTGCGTTGAAGCTAGAGCAGTTAGAGACCATGATGGG (WT)

Allele2: TGTTGGGTTACAAGGTGAGGTCGTCCGAGATGGACCAAGG (complicated variant)

Ref: TGTTGGGTTACAAGGTGAGGTCGTCCGAGATGGCCGAGGTTGCGTTGAAGCTAGAGCAGTTAGAGACCATGATGGG

L10 *BnaA9.RGA*-sgRNA1

Allele1: TGGGTTACAAGGTGAGGTCGTCCGAGATGGCCCGAGGTTGCGTTGAAAGCTAGAGCAGTTAGAGACCATGATGG (insertion)

Allele2: TGGGTTACAAGGTGAGGTCGTCCGAGATGGACCGAGGTTGCGTTGAAGCTAGAGCAGTTAGAGACCATGATGGG (insertion)

Allele3: TGGGTTACAAGGTGAGGTCGTCCGAGATGGTCCGAGGTTGCGTTGAAGCTAGAGCAGTTAGAGACCATGATG (insertion)

Ref: TGGGTTACAAGGTGAGGTCGTCCGAGATGG-CCGAGGTTGCGTTGAAGCTAGAGCAGTTAGAGACCATGATGGGT

L11 *BnaA9.RGA*-sgRNA1

Allele1: TGGGTTACAAGGTGAGGTCGTCCGAGATGGCCCGAGGTTGCGTTGAAAGCTAGAGCAGTTAGAGACCATGATGG (insertion)

Allele2: TGGGTTACAAGGTGAGGTCGTCCGAGATGGACCGAGGTTGCGTTGAAGCTAGAGCAGTTAGAGACCATGATGGG (insertion)

Allele3: TGGGTTACAAGGTGAGGTCGTCCGAGATGGTCCGAGGTTGCGTTGAAGCTAGAGCAGTTAGAGACCATGATG (insertion)

Ref: TGGGTTACAAGGTGAGGTCGTCCGAGATGG-CCGAGGTTGCGTTGAAGCTAGAGCAGTTAGAGACCATGATGGGT

L12 *BnaA9.RGA*-sgRNA1

Allele1: TGGGTTACAAGGTGAGGTCGTCCGAGATGG-CCGAGGTTGCGTTGAAGCTAGAGCAGTTAGAGACCATGATGG (WT)

Allele2: TGGGTTACAAGGTGAGGTCGTCCGAGATGGACCGAGGTTGCGTTGAAGCTAGAGCAGTTAGAGACCATGATGGG (insertion)

Ref: TGGGTTACAAGGTGAGGTCGTCCGAGATGG-CCGAGGTTGCGTTGAAGCTAGAGCAGTTAGAGACCATGATGGGT

L13 *BnaA9.RGA*-sgRNA1

Allele: TGTTGGGTTACAAGGTGAGGTCGTCCGAGATGGCCGAGGTTGCGTTGAAGCTAGAGCAGTTAGAGACCATGATGGG (WT)

Ref: TGTTGGGTTACAAGGTGAGGTCGTCCGAGATGGCCGAGGTTGCGTTGAAGCTAGAGCAGTTAGAGACCATGATGGG

L14 *BnaA9.RGA*-sgRNA1

Allele: TGTTGGGTTACAAGGTGAGGTCGTCCGAGATGGCCGAGGTTGCGTTGAAGCTAGAGCAGTTAGAGACCATGATGGG (WT)

Ref: TGTTGGGTTACAAGGTGAGGTCGTCCGAGATGGCCGAGGTTGCGTTGAAGCTAGAGCAGTTAGAGACCATGATGGG

L15 *BnaA9.RGA*-sgRNA1

Allele1: TGTTGGGTTACAAGGTGAGGTCGTCCGAGATGGCCGAGGTTGCGTTGAAGCTAGAGCAGTTAGAGACCATGATGGG (WT)

Allele2: TGTTGGGTTACAAGGTGAGGTCGTCCGAGATGGACCAAGG (complicated variant)

Ref: TGTTGGGTTACAAGGTGAGGTCGTCCGAGATGGCCGAGGTTGCGTTGAAGCTAGAGCAGTTAGAGACCATGATGGG

L16 *BnaA9.RGA*-sgRNA1

Allele1: TGGGTTACAAGGTGAGGTCGTCCGAGAT---CCGAGGTTGCGTTGAAGCTAGAGCAGTTAGAGACCATGATGGGT (deletion)

Allele2: TGGGTTACAAGGTGAGGTCGTCCGAGATGGTCCGAGGTTGCGTTGAAGCTAGAGCAGTTAGAGACCATGATGGGT (insertion)

Ref: TGGGTTACAAGGTGAGGTCGTCCGAGATGG-CCGAGGTTGCGTTGAAGCTAGAGCAGTTAGAGACCATGATGGGT

L17 *BnaA9.RGA*-sgRNA1

Allele1: TGGGTTACAAGGTGAGGTCGTCCGAGATGG-CCGAGGTTGCGTTGAAGCTAGAGCAGTTAGAGACCATGATGG (WT)

Allele2: TGGGTTACAAGGTGAGGTCGTCCGAGATGGACCGAGGTTGCGTTGAAGCTAGAGCAGTTAGAGACCATGATGGG (insertion)

Ref: TGGGTTACAAGGTGAGGTCGTCCGAGATGG-CCGAGGTTGCGTTGAAGCTAGAGCAGTTAGAGACCATGATGGGT

L18 *BnaA9.RGA*-sgRNA1

Allele1: TGTTGGGTTACAAGGTGAGGTCGTCCGAGATGGCCGAGGTTGCGTTGAAGCTAGAGCAGTTAGAGACCATGATGGG (WT)

Allele2: TGTTGGGTTACAAGGTGAGGTCGTCCGAGATGGACCAAGG (complicated variant)

Ref: TGTTGGGTTACAAGGTGAGGTCGTCCGAGATGGCCGAGGTTGCGTTGAAGCTAGAGCAGTTAGAGACCATGATGGG

L21 *BnaA9.RGA*-sgRNA1

Allele1: TGGGTTACAAGGTGAGGTCGTCCGAGATGG-CCGAGGTTGCGTTGAAGCTAGAGCAGTTAGAGACCATGATGG (WT)

Allele2: TGGGTTACAAGGTGAGGTCGTCCGAGATGGACCGAGGTTGCGTTGAAGCTAGAGCAGTTAGAGACCATGATGGG (insertion)

Ref: TGGGTTACAAGGTGAGGTCGTCCGAGATGG-CCGAGGTTGCGTTGAAGCTAGAGCAGTTAGAGACCATGATGGGT

L22 *BnaA9.RGA*-sgRNA1

Allele: TGTTGGGTTACAAGGTGAGGTCGTCCGAGATGGCCGAGGTTGCGTTGAAGCTAGAGCAGTTAGAGACCATGATGGG (WT)

Ref: TGTTGGGTTACAAGGTGAGGTCGTCCGAGATGGCCGAGGTTGCGTTGAAGCTAGAGCAGTTAGAGACCATGATGGG

L27 *BnaRGA*-sgRNA1

Allele1: ACTTCTACGAGACGTGCCCT-----CAAGTTCGCTCACTTCACGGCCAACCAAGCCATCCTCGAGGCTTT (deletion)

Allele2: ACTTCTACGAGACGTGCCCTT-CCTCAAGTTCGCTCACTTCACGGCCAACCAAGCCATCCTCGAGGCTTT (deletion)

Ref: ACTTCTACGAGACGTGCCCTTACCTCAAGTTCGCTCACTTCACGGCCAACCAAGCCATCCTCGAGGCTTT

L28 *BnaRGA*-sgRNA2

Allele1: GGTGGGATAGAGAAAGTTCTCGGCGTGGTGAAACAGATTAAACCGGTGATTTTCACGGTGGTTGAGCAAG (WT)

Allele2: GGTGGGATAGAGAAAGTTCTCGGCGTGGTGAAACAGATTAAACCGGTGATT-TCACGGTGGTTGAGCAAG (deletion)

Ref: GGTGGGATAGAGAAAGTTCTCGGCGTGGTGAAACAGATTAAACCGGTGATTTTCACGGTGGTTGAGCAAG

L29 *BnaRGA*-sgRNA1

Allele1: TTCTACGAGACGTGCCCTTA--------TTCGCTCACTTCACGGCCAACCAAGCCATCCTCGAGGCTTTCG (deletion)

Allele2: TTCTACGAGACGTGCCCTTAACCTCAAGTTCGCTCACTTCACGGCCAACCAAGCCATCCTCGAGGCTTTCG (insertion)

Ref: TTCTACGAGACGTGCCCTTA-CCTCAAGTTCGCTCACTTCACGGCCAACCAAGCCATCCTCGAGGCTTTCG

L30 *BnaRGA*-sgRNA2

Allele1: GTGGGATAGAGAAAGTTCTCGGCGTGGTGAAACAGATTAAACCGGTGATT-TCACGGTGGTTGAGCAAGA (deletion)

Allele2: GTGGGATAGAGAAAGTTCTCGGCGTGGTGAAACAGATTAAACCGGTGA--TTCACGGTGGTTGAGCAAGA (deletion)

Ref: GTGGGATAGAGAAAGTTCTCGGCGTGGTGAAACAGATTAAACCGGTGATTTTCACGGTGGTTGAGCAAGA

L31 *BnaRGA*-sgRNA1

Allele1: TGCACTTCTACGAGACGTGCCCTTAA--------TTCCCTCACTTCACGGCCAACCAAGCCATCCTCG (deletion and insertion)

Allele2: TGCACTTCTACGAGACGTGCCCTTA--------ATTCGCTCACTTCACGGCCAACCAAGCCATCCTCGAGGCTTTCG (deletion)

Allele3: TGCACTTCTACGAGACGTGCCCTTAACCTCAAGTTCGCTCACTTCACGGCCAACCAAGCCATCCTCGAGGCT (insertion)

Allele4: TGCACTTCTACGAGACGTGCCCTTACCCTCAAGTTCGCTCACTTCACGGCCAACCAAGCCATCCTCGAG (insertion)

Ref: TGCACTTCTACGAGACGTGCCCTTA-CCTCAAGTTCGCTCACTTCACGGCCAACCAAGCCATCCTCGAGGCTTTCG

L31 *BnaRGA*-sgRNA2

Allele1: TCTCGGCGTGGTGAAACAGATTAAACCGG-----TTCACGGTGGTTGAGCAAGAATCGAGTCATAACGGTCC (deletion)

Allele2: TCTCGGCGTGGTGAAACAGATTAAACCGGTGATTTTCACGGTGGTTGAGCAAGAATCGAGTCATAACGG (WT)

Ref: TCTCGGCGTGGTGAAACAGATTAAACCGGTGATTTTCACGGTGGTTGAGCAAGAATCGAGTCATAACGG

L32 *BnaRGA*-sgRNA2

Allele1: TGGGATAGAGAAAGTTCTCGGCGTGGTGAAACAGATTAAACCGGTGATTTTCACGGTGGTTGAGCA (WT)

Allele2: TGGGATAGAGAAAGTTCTCGGCGTGGTGAAACAGATTAAACCGGTGATT-TCACGGTGGTTGAGCA (deletion)

Ref: TGGGATAGAGAAAGTTCTCGGCGTGGTGAAACAGATTAAACCGGTGATTTTCACGGTGGTTGAGCAAG

L33 *BnaRGA*-sgRNA1

Allele1: ACTTCTACGAGACGTGCCCT--CCTCAAGTTCGCTCACTTCACGGCCAACCAAGCCATCCTCGAGGCTTT (deletion)

Allele2: ACTTCTACGAGACGTGCCCT----TCAAGTTCGCTCACTTCACGGCCAACCAAGCCATCCTCGAGGCTTT (deletion)

Ref: ACTTCTACGAGACGTGCCCTTACCTCAAGTTCGCTCACTTCACGGCCAACCAAGCCATCCTCGAGGCTTT

L34 *BnaRGA*-sgRNA1

Allele1: ACTTCTACGAGACGTGCCCT--CCTCAAGTTCGCTCACTTCACGGCCAACCAAGCCATCCTCGAGGCTTT (deletion)

Allele2: ACTTCTACGAGACGTGCCCT----TCAAGTTCGCTCACTTCACGGCCAACCAAGCCATCCTCGAGGCTTT (deletion)

Ref: ACTTCTACGAGACGTGCCCTTACCTCAAGTTCGCTCACTTCACGGCCAACCAAGCCATCCTCGAGGCTTT

L35 *BnaRGA*-sgRNA1

Allele1: ACTTCTACGAGACGTGCCCTTACCTCAAGTTCGCTCACTTCACGGCCAACCAAGCCATCCTCGAGGCTTT (WT)

Allele2: ACTTCTACGAGACGTGCCCTTAC (complicated variant)

Ref: ACTTCTACGAGACGTGCCCTTACCTCAAGTTCGCTCACTTCACGGCCAACCAAGCCATCCTCGAGGCTTT

L36 *BnaRGA*-sgRNA1

Allele1: TTCTACGAGACGTGCCCTTAACCTCAAGTTCGCTCATCACGGCCAACCAAGCCATCCTCGAGGCTTTCG (insertion)

Allele2: TTCTACGAGACGTGCCCTTA-----AAGTTCGCTCACTTCACGGCCAACCAAGCCATCCTCGAGGCTTTCG (deletion)

Ref: TTCTACGAGACGTGCCCTTA-CCTCAAGTTCGCTCACTTCACGGCCAACCAAGCCATCCTCGAGGCTTTCG

L37 *BnaRGA*-sgRNA1

Allele1: ACTTCTACGAGACGTGCCCT-----CAAGTTCGCTCACTTCACGGCCAACCAAGCCATCCTCGAGGCTTT (deletion)

Allele2: ACTTCTACGAGACGTGCCCT (complicated variant)

Ref: ACTTCTACGAGACGTGCCCTTACCTCAAGTTCGCTCACTTCACGGCCAACCAAGCCATCCTCGAGGCTTT

L37 *BnaRGA*-sgRNA2

Allele1: GTGGGATAGAGAAAGTTCTCGGCGTGGTGAAACAGATTAAACCGGTGATTTTCACGGTGGTTGAGCAAGA (WT)

Allele2: GTGGGATAGAGAAAGTTCTCGGCGTGGTGAAACAGATTAAACCGGTGAT--TCACGGTGGTTGAGCA (deletion)

Ref: GTGGGATAGAGAAAGTTCTCGGCGTGGTGAAACAGATTAAACCGGTGATTTTCACGGTGGTTGAGCAAGA

L38 *BnaRGA*-sgRNA1

Allele1: AGATGCACTTCTACGAGACG--------------AGTTCGCTCACTTCACGGCCAACCAAGCCATCCTCGA (deletion)

Allele2: AGATGCACTTCTACGAGACGTGCCCTTATCCTCAAGTTCGCTCACTTCACGGCCAACCAAGCCATCCTCGA (insertion)

Ref: AGATGCACTTCTACGAGACGTGCCCTTA-CCTCAAGTTCGCTCACTTCACGGCCAACCAAGCCATCCTCGA

L39 *BnaRGA*-sgRNA1

Allele1: TTCTACGAGACGTGCCCTTATCCTCAAGTTCGCTCACTTCACGGCCAACCAAGCCATCCTCGA (insertion)

Ref: TTCTACGAGACGTGCCCTTA-CCTCAAGTTCGCTCACTTCACGGCCAACCAAGCCATCCTCGA

L40 *BnaRGA*-sgRNA1

Allele1: CTCCGACACGCTCCAGATGCACTTCTACGAGACGTGCCCTTACCTCAAGTTCGCTCACTTCACGGCCAAC (WT)

Allele2: CTCCGACACGCTCCAGATGC----------------------CCTCAAGTTCGCTCACTTCACGGCCAAC (deletion)

Ref: CTCCGACACGCTCCAGATGCACTTCTACGAGACGTGCCCTTACCTCAAGTTCGCTCACTTCACGGCCAAC

L41 *BnaRGA*-sgRNA1

Allele1: ACTTCTACGAGACGTGCCCT------CAAGTTCGCTCACTTCACGGCCAACCAAGCCATCCTCGAGGCTTT (deletion)

Allele2: ACTTCTACGAGACGTGCCCTTATCCTCAAGTTCGCTCACTTCACGGCCAACCAAGCCATCCTCGAGGCTTT (insertion)

Ref: ACTTCTACGAGACGTGCCCTTA-CCTCAAGTTCGCTCACTTCACGGCCAACCAAGCCATCCTCGAGGCTTT

L42 *BnaRGA*-sgRNA1

Allele1: TTCTACGAGACGTGCCC--ACCTCAAGTTCGCTCACTTCACGGCCAACCAAGCCATCCTCGAGGCTT (deletion)

Allele2: TTCTACGAGACGTGCCCGCG (complicated variant)

Ref: TTCTACGAGACGTGCCCTTACCTCAAGTTCGCTCACTTCACGGCCAACCAAGCCATCCTCGAGGCTT

L43 *BnaRGA*-sgRNA1

Allele1: CTCCGACACGCTCCAGATGCACTTCTACGAGACGTGCCCTTACCTCAAGTTCGCTCACTTCACGGCCAAC (WT)

Ref: CTCCGACACGCTCCAGATGCACTTCTACGAGACGTGCCCTTACCTCAAGTTCGCTCACTTCACGGCCAAC

L44 *BnaRGA*-sgRNA1

Allele1: GCACTTCTACGAGACGTGCCCTTA------GTTCGCTCACTTCACGGCCAACCAAGCCATCCTCGAGG (deletion)

Ref: GCACTTCTACGAGACGTGCCCTTACCTCAAGTTCGCTCACTTCACGGCCAACCAAGCCATCCTCG

L45 *BnaRGA*-sgRNA1

Allele1: TTCTACGAGACGTGCCC--ACCTCAAGTTCGCTCACTTCACGGCCAACCAAGCCATCCTCGAGGCTT (deletion)

Allele2: TTCTACGAGACGTGCCCGCG (complicated variant)

Ref: TTCTACGAGACGTGCCCTTACCTCAAGTTCGCTCACTTCACGGCCAACCAAGCCATCCTCGAGGCTT

L45 *BnaRGA*-sgRNA2

Allele1:	GATAGAGAAAGTTCTCGGCGTGGTGAAACAGATTAAACCGGTGATTTTCACGGTGGTTGAGCAAGAATCG	(WT)
Allele2:	GATAGAGAAAGTTCTCGGCGTGGTGAAACAGATTAAACCGGTGATTT---CGGTGGTTGAGCAAGAATCG	(deletion)
Ref:	GATAGAGAAAGTTCTCGGCGTGGTGAAACAGATTAAACCGGTGATTTTCACGGTGGTTGAGCAAGAATCG	

L46 *BnaRGA*-sgRNA1

Allele1:	TTCTACGAGACGTGCCCTTA----AAAGTTCGCTCACTTCACGGCCAACCAAGCCATCCTCGAGGCTTT	(insertion and deletion)
Allele2:	TTCTACGAGACGTGCCCTTA------AGTTCGCTCACTTCACTTCACGGCCAACCAAGCCATCCTCGAGGCTTTCG	(deletion)
Ref:	TTCTACGAGACGTGCCCTTA-CCTCAAGTTCGCTCACTTCACGGCCAACCAAGCCATCCTCGAGGCTTTCG	

L46 *BnaRGA*-sgRNA2

Allele1:	TGGGATAGAGAAAGTTCTCGGCGTGGTGAAACAGATTAAACCGGTGATT--TCACGGTGGTTGAGCAAGAA	(deletion)
Allele2:	TGGGATAGAGAAAGTTCTCGGCGTGGTGAAACAGATTAAACCGGTGATTTGTCACGGTGGTTGAGCAAGAA	(insertion)
Ref:	TGGGATAGAGAAAGTTCTCGGCGTGGTGAAACAGATTAAACCGGTGATTT-TCACGGTGGTTGAGCAAGAA	

L47 *BnaRGA*-sgRNA1

Allele1:	TTCTACGAGACGTGCCC--ACCTCAAGTTCGCTCACTTCACGGCCAACCAAGCCATCCTCGAGGCTT	(deletion)
Allele2:	TTCTACGAGACGTGCCCGCG	(complicated variant)
Ref:	TTCTACGAGACGTGCCCTTACCTCAAGTTCGCTCACTTCACGGCCAACCAAGCCATCCTCGAGGCTT	

L48 *BnaRGA*-sgRNA1

Allele1:	TTCTACGAGACGTGCCC--ACCTCAAGTTCGCTCACTTCACGGCCAACCAAGCCATCCTCGAGGCTT	(deletion)
Allele2:	TTCTACGAGACGTGCCCGCG	(complicated variant)
Ref:	TTCTACGAGACGTGCCCTTACCTCAAGTTCGCTCACTTCACGGCCAACCAAGCCATCCTCGAGGCTT	

L49 *BnaRGA*-sgRNA1

Allele1:	GCACTTCTACGAGACGTGCC-----------TTCGCTCACTTCACGGCCAACCAAGCCATCCTCGAGGCT	(deletion)
Allele2:	GCACTTCTACGAGACGTGCCCTTACCCTCAAGTTCGCTCACTTCACGGCCAACCAAGCCATCCTCGAGGCT	(insertion)
Ref:	GCACTTCTACGAGACGTGCCCTTA-CCTCAAGTTCGCTCACTTCACGGCCAACCAAGCCATCCTCGAGGCT	

L50 *BnaRGA*-sgRNA1

Allele1: TTCTACGAGACGTGCCCTTA------AGTTCGCTCACTTCACGGCCAACCAAGCCATCCTCGAGGCTTTCG (deletion)

Allele2: TTCTACGAGACGTGCCCTTACCCTCAAGTTCGCTCTTCACGGCCAACCAAGCCATCCTCGAGGCTTTCG (insertion)

Ref: TTCTACGAGACGTGCCCTTA-CCTCAAGTTCGCTCACTTCACGGCCAACCAAGCCATCCTCGAGGCTTTCG

L51 *BnaRGA*-sgRNA1

Allele1: TTCTACGAGACGTGCCC--ACCTCAAGTTCGCTCACTTCACGGCCAACCAAGCCATCCTCGAGGCTT (deletion)

Allele2: TTCTACGAGACGTGCCCGCG (complicated variant)

Ref: TTCTACGAGACGTGCCCTTACCTCAAGTTCGCTCACTTCACGGCCAACCAAGCCATCCTCGAGGCTT

L52 *BnaRGA*-sgRNA1

Allele1: TTCTACGAGACGTGCCCTTA-CTCAAGTTCGCTCACTTCACGGCCAACCAAGCCATCCTCGAGGCTT (deletion)

Ref: TTCTACGAGACGTGCCCTTACCTCAAGTTCGCTCACTTCACGGCCAACCAAGCCATCCTCGAGGCTT

L53 *BnaRGA*-sgRNA1

Allele1: TTCTACGAGACGTGCCCTTATCCTCAAGTTCGCTCACTTCACGGCCAACCAAGCCATCCTCGAGGCTT (insertion)

Allele2: TTCTACGAGACGTGCCCTTACCCTCAAGTTCGCTCACTTCACGGCCAACCAAGCCATCCTCGAGGCTT (insertion)

Ref: TTCTACGAGACGTGCCCTTA-CCTCAAGTTCGCTCACTTCACGGCCAACCAAGCCATCCTCGAGGCTT

L54 *BnaRGA*-sgRNA1

Allele1: CTTCTACGAGACGTGCCCTT-----CAAGTTCGCTCACTTCACGGCCAACCAAGCCATCCTCGAGGCTTTC (deletion)

Allele2: CTTCTACGAGACGTGCCCTTAACCTCAAGTTCGCTCACTTCACGGCCAACCAAGCCATCCTCGAGGCTTTC (insertion)

Ref: CTTCTACGAGACGTGCCCTTA-CCTCAAGTTCGCTCACTTCACGGCCAACCAAGCCATCCTCGAGGCTTTC

L55 *BnaRGA*-sgRNA1

Allele1: TTCTACGAGACGTGCCCTTA------AGTTCGCTCACTTCACGGCCAACCAAGCCATCCTCGAGGCTTTCG (deletion)

Allele2: TTCTACGAGACGTGCCCTTACCCTCAAGTTCGCTCACTTCACGGCCAACCAAGCCATCCTCGAGGCTTTCG (insertion)

Ref: TTCTACGAGACGTGCCCTTA-CCTCAAGTTCGCTCACTTCACGGCCAACCAAGCCATCCTCGAGGCTTTCG

BnaC9.RGA

L1 *BnaC9.RGA*-sgRNA1

Allele1: ATGAGCTTCTCGCTGTGTTGGGTTACAAGGTGAGGTCGTCAG (deletion 118bp) CTCGTGGCTTGATAACATGC (deletion)

Allele2: ATGAGCTTCTCGCTGTGTTGGGTTACAAGGTGAGGTCGTCAGAGATGGCGGAGGTTGCGTTGAAGCTAGAGCAG (WT)

Ref: ATGAGCTTCTCGCTGTGTTGGGTTACAAGGTGAGGTCGTCAGAGATGGCGGAGGTTGCGTTGAAGCTAGAGCAG

L3 *BnaC9.RGA*-sgRNA1

Allele1: TGGATGAGCTTCTCG (deletion 152bp) TGGCTTGATAACATGCTCACGGA (deletion)

Allele2: TGGATGAGCTTCTCGCTGTGTTGGGTTACAAGGTGAGGTCGTCAGAGATGGCGGAGGTTGCGTTGAAGCTAGAGCAG (WT)

Ref: TGGATGAGCTTCTCGCTGTGTTGGGTTACAAGGTGAGGTCGTCAGAGATGGCGGAGGTTGCGTTGAAGCTAGAGCAG

L4 *BnaC9.RGA*-sgRNA1

Allele1: ATGAGCTTCTCGCTGTGTTGGGTTACAAGGTGAGGTCGTCAG (deletion 118bp) CTCGTGGCTTGATAACATGC (deletion)

Allele2: ATGAGCTTCTCGCTGTGTTGGGTTACAAGGTGAGGTCGTCAGAGATGGCGGAGGTTGCGTTGAAGCTAGAGCAG (WT)

Ref: ATGAGCTTCTCGCTGTGTTGGGTTACAAGGTGAGGTCGTCAGAGATGGCGGAGGTTGCGTTGAAGCTAGAGCAG

L5 *BnaC9.RGA*-sgRNA1

Allele1: ATGAGCTTCTCGCTGTGTTGGGTTACAAGGTGAGGTCGTCAG (deletion 118bp) CTCGTGGCTTGATAACATGC (deletion)

Allele2: ATGAGCTTCTCGCTGTGTTGGGTTACAAGGTGAGGTCGTCAGAGATGGCGGAGGTTGCGTTGAAGCTAGAGCAG (WT)

Ref: ATGAGCTTCTCGCTGTGTTGGGTTACAAGGTGAGGTCGTCAGAGATGGCGGAGGTTGCGTTGAAGCTAGAGCAG

L6 *BnaC9.RGA*-sgRNA2

Allele1: GATACTGTTCACTACAACCCGTCGGAGCTCTACTCGTGGCTTGATAACATGCTCACGGAGTTTAACCCGCCGC (substitution)

Allele2: GATACTGTTCACTACAACCCGTCGGAGCTTTACTCGTGGCTTGATAACATGCTCACGGAGTTTAACCCGCCGC (WT)

Ref: GATACTGTTCACTACAACCCGTCGGAGCTTTACTCGTGGCTTGATAACATGCTCACGGAGTTTAACCCGCCGC

L7 *BnaC9.RGA*-sgRNA2

Allele1: GATACTGTTCACTACAACCCGTCGGAGCTCTACTCGTGGCTTGATAACATGCTCACGGAGTTTAACCCGCCGC (substitution)

Allele2: GATACTGTTCACTACAACCCGTCGGAGCTTTACTCGTGGCTTGATAACATGCTCACGGAGTTTAACCCGCCGC (WT)

Ref: GATACTGTTCACTACAACCCGTCGGAGCTTTACTCGTGGCTTGATAACATGCTCACGGAGTTTAACCCGCCGC

L8 *BnaC9.RGA*-sgRNA2

Allele1: GATACTGTTCACTACAACCCGTCGGAGCTCTACTCGTGGCTTGATAACATGCTCACGGAGTTTAACCCGCCGC (substitution)

Allele2: GATACTGTTCACTACAACCCGTCGGAGCTTTACTCGTGGCTTGATAACATGCTCACGGAGTTTAACCCGCCGC (WT)

Ref: GATACTGTTCACTACAACCCGTCGGAGCTTTACTCGTGGCTTGATAACATGCTCACGGAGTTTAACCCGCCGC

L9 *BnaC9.RGA*-sgRNA2

Allele1: GATACTGTTCACTACAACCCGTCGGAGCTCTACTCGTGGCTTGATAACATGCTCACGGAGTTTAACCCGCCGC (substitution)

Allele2: GATACTGTTCACTACAACCCGTCGGAGCTTTACTCGTGGCTTGATAACATGCTCACGGAGTTTAACCCGCCGC (WT)

Ref: GATACTGTTCACTACAACCCGTCGGAGCTTTACTCGTGGCTTGATAACATGCTCACGGAGTTTAACCCGCCGC

L10 *BnaC9.RGA*-sgRNA2

Allele1: GATACTGTTCACTACAACCCGTCGGAGCTCTACTCGTGGCTTGATAACATGCTCACGGAGTTTAACCCGCCGC (substitution)

Allele2: GATACTGTTCACTACAACCCGTCGGAGCTTTACTCGTGGCTTGATAACATGCTCACGGAGTTTAACCCGCCGC (WT)

Ref: GATACTGTTCACTACAACCCGTCGGAGCTTTACTCGTGGCTTGATAACATGCTCACGGAGTTTAACCCGCCGC

L11 *BnaC9.RGA*-sgRNA2

Allele1: GATACTGTTCACTACAACCCGTCGGAGCTCTACTCGTGGCTTGATAACATGCTCACGGAGTTTAACCCGCCGC (substitution)

Allele2: GATACTGTTCACTACAACCCGTCGGAGCTTTACTCGTGGCTTGATAACATGCTCACGGAGTTTAACCCGCCGC (WT)

Ref: GATACTGTTCACTACAACCCGTCGGAGCTTTACTCGTGGCTTGATAACATGCTCACGGAGTTTAACCCGCCGC

L15 *BnaC9.RGA*-sgRNA2

Allele1: GATACTGTTCACTACAACCCGTCGGAGCTCTACTCGTGGCTTGATAACATGCTCACGGAGTTTAACCCGCCGC (substitution)

Allele2: GATACTGTTCACTACAACCCGTCGGAGCTTTACTCGTGGCTTGATAACATGCTCACGGAGTTTAACCCGCCGC (WT)

Ref: GATACTGTTCACTACAACCCGTCGGAGCTTTACTCGTGGCTTGATAACATGCTCACGGAGTTTAACCCGCCGC

L27 *BnaRGA*-sgRNA1

Allele1: CACTTCTACGAGACGTGCCCT-----CAAGTTCGCTCACTTCACGGCCAACCAAGCCATCCTCGAGGCTTT (deletion)

Allele2: CACTTCTACGAGACGTGCCCTT-CCTCAAGTTCGCTCACTTCACGGCCAACCAAGCCATCCTCGAGGCTTT (deletion)

Allele3: CACTTCTACGAGACGTGCCCTTACCT------------------------------------CGAGGCTTTCGAAGGGAAG (deletion)

Allele4: CACTCTCCCTTCGAAAGCCC---------------------------------AGCCATCCTCGAGGCTTTCGAAGGGAAG (deletion)

Ref: CACTTCTACGAGACGTGCCCTTACCTCAAGTTCGCTCACTTCACGGCCAACCAAGCCATCCTCGAAGACGTGGGGAA

L29 *BnaRGA*-sgRNA1

Allele1: CAGATGCACTTCTACGAGACGTGCCCTTAACCTCAAGTTCGCTCA (insertion)

Allele2: CAGATGCACTTCTACGAGACGTGCCCTTA------TTTCGCTCACTTCACGGCCAACCAA (deletion and insertion)

Allele3: CAGATGCACTTCTACGAGACGTGCCCTTA-------TTCGCTCACTTCACGGCCAACCAA (deletion)

Allele4: CAGAGGCACATCTATGAGACGTGCC-------CCAA-TTCGCTCACTTCACGGCCAACCAA (deletion and insertion)

Ref: CAGATGCACTTCTACGAGACGTGCCCTTA-CCTCAAGTTCGCTCACTTCACGGCCAACCAA

L31 *BnaRGA*-sgRNA1

Allele1: CAGATGCACTTCTACGAGACGTGCCCTTACCCTCAAGTTCGCTCACTTCACGGCCAACCA (insertion)

Allele2: TCCAGATGCACTTCTACGAGACGTGCCCTTA--CTCAAGTTCGCTCACTTCACGGCCAACC (deletion)

Allele3: CAGATGCACTTCTACGAGACGTGCCCTTAACCTCAAGTTCGCTCACTTCACGGCCAACCA (insertion)

Ref: CAGATGCACTTCTACGAGACGTGCCCTTA-CCTCAAGTTCGCTCACTTCACGGCCAACCA

L31 *BnaRGA*-sgRNA2:

Allele1: TGGGATAGAGAAAGTTCTCGGCGTGGTGAAACAGATTAAACCGG-----TTCACGGTGGT (deletion)

Allele2: TGGGATAGAGAAAGTTCTCGGCGTGGTGAAACAGATTAAAC-----------ACGGTGGT (deletion)

Ref: TGGGATAGAGAAAGTTCTCGGCGTGGTGAAACAGATTAAACCGGTGATTTTCACGGTGGT

L33 *BnaRGA*-sgRNA1

Allele1: CAGATGCACTTCTACGAGACGTGCCCTTA-----AGTTCGCTCACTTCACGGCCAACCAA (deletion)

Allele2: CAGATGCACTTCTACGAGACGTGCCCTTA--------TCGCTCACTTCACGGCCAACCAA (deletion)

Ref: CAGATGCACTTCTACGAGACGTGCCCTTACCTCAAGTTCGCTCACTTCACGGCCAACCAA

L38 *BnaRGA*-sgRNA1

Allele1:	CACTTCTACGAGACGTGCCCT------AAGTTCGCTCACTTCACGGCCAACCAA	(deletion)
Allele2:	CACTTCTACGAGACGTGCCCTT--CTCAAGTTCGCTCACTTCACGGCCAACCAA	(deletion)
Ref:	CACTTCTACGAGACGTGCCCTTACCTCAAGTTCGCTCACTTCACGGCCAACCAA	

L40 *BnaRGA*-sgRNA1

Allele1:	CTACGAGACGTGCCCTTACCCTCAAGTTCGCTCACTTCACGGCCAACCAA	(insertion)
Allele2:	CTACGAGACGTGCCCTTA------GTTCGCTCACTTCACTTCACGGCCAACCAA	(deletion)
Allele3:	CTACGAGACGTGCCCTTA-CCTCAAGTTCGCTCACTTCACGGCCAACCAA	(WT)
Ref:	CTACGAGACGTGCCCTTA-CCTCAAGTTCGCTCACTTCACGGCCAACCAA	

L46 *BnaRGA*-sgRNA1

Allele1:	AGATGCACTTCTACGAGACGTGCCCTTAATCCTCAAGTTCGCTCACTTCACGGCCAACCAAGCCATCC	(insertion)
Allele2:	AGATGCACTTCTACGAGACGTGCCCTTA-----AAAGTTCGCTCACTTCACGGCCAACCAAGCCATCCT	(deletion and insertion)
Allele3:	AGATGCACTTCTACGAGACGTGCCCTTA------AAGTTCGCTCACTTCACGGCCAACCAAGCCATCCTCG	(deletion)
Ref:	AGATGCACTTCTACGAGACGTGCCCTTA--CCTCAAGTTCGCTCACTTCACGGCCAACCAAGCCATCCTCG	

L46 *BnaRGA*-sgRNA2

Allele1:	CGGCGTGGTGAAACAGATTAAACCGGTGATT-TCACGGTGGTTGAGCAAGAATCGAGTCAT	(deletion)
Allele2:	CGGCGTGGTGAAACAGATTAAACCGGTGATTTTCACGGTGGTTGAGCAAGAGTCGAATCAT	(WT)
Ref:	CGGCGTGGTGAAACAGATTAAACCGGTGATTTTCACGGTGGTTGAGCAAGAATCGAATCA	

L55 *BnaRGA*-sgRNA1

Allele1:	CAGATGCCCTTCTACGAGACGTGCCCTTACCCTCAAGTTCGCTCACTTCACGGCCAACCA	(insertion)
Allele2:	CAGATGCACTTCTACGAGACGTGCCCTT----CAAGTTCGCTCACTTCACGGCCAACCAA	(deletion)
Ref:	CCAGATGCACTTCTACGAGACGTGCCCTTA-CCTCAAGTTCGCTCACTTCACGGCCA	

L44 *BnaRGA*-sgRNA1

Allele1: AGATGCACTTATACGAGACGTGCCCTTA-------GTTCGCTCACTTCACGGCCAACCAAGCCATCCT (deletion)

Allele2: AGATGCACTTCTACGAGACGTGCCCTTAACCTCAAGTTCGCTCACTTCACGGCCAACCAAGCCAT (insertion)

Ref: AGATGCACTTCTACGAGACGTGCCCTTA-CCTCAAGTTCGCTCACTTCACGGCCAACCAAGCCAT

L44 *BnaRGA*-sgRNA2

Allele1: TCTCGGCGTGGTGAAACAGATTAAACCGGGTGT---------GGTTGAGCAAGAGTCGAATCATAA (deletion)

Allele2: TCTCGGCGTGGTGAAACAGATTAAACCGGTGATTTTCACGGTGGTTGAGCAAGAATCGAGTC (WT)

Ref: TCTCGGCGTGGTGAAACAGATTAAACCGGTGATTTTCACGGTGGTTGAGCAAGAATCGAA

BnaC7.RGA

L1 *BnaC7.RGA*-sgRNA1

Allele1: TTGGGTTACAAGGTCAGGTCGTCGGAGATGGCTGAGGTTGCGTTGAAACTCGAG (WT)

Ref: TTGGGTTACAAGGTCAGGTCGTCGGAGATGGCTGAGGTTGCGTTGAAACTCGAG

L2 *BnaC7.RGA*-sgRNA2

Allele1: TTACAACCCTGCTGAGCTCTACTCTTGGCTTGATAACATGCTCACGGAGCTTAACCCACCCGCTG (WT)

Allele2: TTACAACCCTGCTGAGCTCT-----TGGCTTGATAACATGCTCACGGAGCTTAACCCACCCGCT (deletion)

Ref: TTACAACCCTGCTGAGCTCTACTCTTGGCTTGATAACATGCTCACGGAGCTTAACCCACCCGCTGCGTCC

L7 *BnaC7.RGA*-sgRNA2

Allele1: CATTACAACCCTGCTGAGCT----TCTTGGCTTGATAACATGCTCACGGAGCTTAACCCACCC (deletion)

Allele2: CATTACAACCCTGCTGAGCTCT-TCTTGGCTTGATAACATGCTCACGGAGCTTAACCCACCC (deletion)

Ref: CATTACAACCCTGCTGAGCTCTACTCTTGGCTTGATAACATGCTCACGGAGCTTAACCCACCCGCTGCGT

L8 *BnaC7.RGA*-sgRNA2

Allele1: TTGGGTTACAAGGTCAGGTCGTCGGAGATGGCTGAGGTTGCGTTGAAACTCGAG (WT)

Ref: TTGGGTTACAAGGTCAGGTCGTCGGAGATGGCTGAGGTTGCGTTGAAACTCGAG

L9 *BnaC7.RGA*-sgRNA1

Allele1: TTGGGTTACAAGGTCAGGTCGTCGGAGATGGCTGAGGTTGCGTTGAAACTCGAG (WT)

Ref: TTGGGTTACAAGGTCAGGTCGTCGGAGATGGCTGAGGTTGCGTTGAAACTCGAG

L10 *BnaC7.RGA*-sgRNA1

Allele1: TTGGGTTACAAGGTCAGGTCGTCGGAGATGGCTGAGGTTGCGTTGAAACTCGAG (WT)

Ref: TTGGGTTACAAGGTCAGGTCGTCGGAGATGGCTGAGGTTGCGTTGAAACTCGAG

L11 *BnaC7.RGA*-sgRNA1

Allele1: TTGGGTTACAAGGTCAGGTCGTCGGAGATGGCTGAGGTTGCGTTGAAACTCGAG (WT)

Ref: TTGGGTTACAAGGTCAGGTCGTCGGAGATGGCTGAGGTTGCGTTGAAACTCGAG

L12 *BnaC7.RGA*-sgRNA2

Allele1: TTACAACCCTGCTGAGCTCT-CTCTTGGCTTGATAACATGCTCACGGAGCTTAACCCACCC (deletion)

Allele2: TTACAACCCTGCTGAGCTCTATTGGGTTGA (complicated variant)

Ref: TTACAACCCTGCTGAGCTCTACTCTTGGCTTGATAACATGCTCACGGAGCTTAACCCACCCGCTGCGTCC

L13 *BnaC7.RGA*-sgRNA1

Allele1: TTGGGTTACAAGGTCAGGTCGTCGGAGATGGCTGAGGTTGCGTTGAAACTCGAG (WT)

Ref: TTGGGTTACAAGGTCAGGTCGTCGGAGATGGCTGAGGTTGCGTTGAAACTCGAG

L14 *BnaC7.RGA*-sgRNA1

Allele1: TTGGGTTACAAGGTCAGGTCGTCGGAGATGGCTGAGGTTGCGTTGAAACTCGAG (WT)

Ref: TTGGGTTACAAGGTCAGGTCGTCGGAGATGGCTGAGGTTGCGTTGAAACTCGAG

L15 *BnaC7.RGA*-sgRNA1

Allele1: TTGGGTTACAAGGTCAGGTCGTCGGAGATGGCTGAGGTTGCGTTGAAACTCGAG (WT)

Ref: TTGGGTTACAAGGTCAGGTCGTCGGAGATGGCTGAGGTTGCGTTGAAACTCGAG

L16 *BnaC7.RGA*-sgRNA1

Allele1: TTGGGTTACAAGGTCAGGTCGTCGGAGATGGCTGAGGTTGCGTTGAAACTCGAG (WT)

Ref: TTGGGTTACAAGGTCAGGTCGTCGGAGATGGCTGAGGTTGCGTTGAAACTCGAG

L17 *BnaC7.RGA*-sgRNA1

Allele1: TTGGGTTACAAGGTCAGGTCGTCGGAGATGGCTGAGGTTGCGTTGAAACTCGAG (WT)

Ref: TTGGGTTACAAGGTCAGGTCGTCGGAGATGGCTGAGGTTGCGTTGAAACTCGAG

L18 *BnaC7.RGA*-sgRNA1

Allele1: TTGGGTTACAAGGTCAGGTCGTCGGAGATGGCTGAGGTTGCGTTGAAACTCGAG (WT)

Ref: TTGGGTTACAAGGTCAGGTCGTCGGAGATGGCTGAGGTTGCGTTGAAACTCGAG

L19 *BnaC7.RGA*-sgRNA2

Allele1: GTTCATTACAACCCTGCTGAGCTCTACTCTTGGCTTGATAACATGCTCACGGAGCTTAACCCACCCGCTG (WT)

Allele2: GTTCATTACAACCCTGCTGA-------TCTTGGCTTGATAACATGCTCACGGAGCTTAACCCACCCGCTG (deletion)

Ref: GTTCATTACAACCCTGCTGAGCTCTACTCTTGGCTTGATAACATGCTCACGGAGCTTAACCCACCCGCTG

L21 *BnaC7.RGA*-sgRNA1

Allele1: TTACAACCCTGCTGAGCTCTACTCTTGGCTTGATAACATGCTCACGGAGCTTAACCCAC (WT)

Allele2: TTACAACCCTGCTGAGCTCTACTGTTGGCTTGATAACATGCTCACGGAGCTTAACCCAC (substitution)

Ref: TTACAACCCTGCTGAGCTCTACTCTTGGCTTGATAACATGCTCACGGAGCTTAACCCACCCGCTGCGTCC

L22 *BnaC7.RGA*-sgRNA1

Allele1: TTGGGTTACAAGGTCAGGTCGTCGGAGATGGCTGAGGTTGCGTTGAAACTCGAG (WT)

Ref: TTGGGTTACAAGGTCAGGTCGTCGGAGATGGCTGAGGTTGCGTTGAAACTCGAG

L23 *BnaC7.RGA*-sgRNA1

Allele1: TTGGGTTACAAGGTCAGGTCGTCGGAGATGGCTGAGGTTGCGTTGAAACTCGAG (WT)

Ref: TTGGGTTACAAGGTCAGGTCGTCGGAGATGGCTGAGGTTGCGTTGAAACTCGAG

L24 *BnaC7.RGA*-sgRNA2

Allele1: TTACAACCCTGCTGAGCTCT-----TGGCTTGATAACATGCTCACGGAGCTTAACCCACCCGCTGCGTCC (deletion)

Allele2: TTACAACCCTGCTGAGCTCTACTTCTTGGT (complicated variant)

Ref: TTACAACCCTGCTGAGCTCTACTCTTGGCTTGATAACATGCTCACGGAGCTTAACCCACCCGCTGCGTCC

L27 *BnaRGA*-sgRNA1

Allele1: CCATTCTCTCTCCGATACTG--------------------------------CCTCAAGTTCGCTCACTTCACGGCGAATCAAGCCA (deletion)

Allele2: ACTTCTACGAGACGTGCCCTTATCCTCAAGTTCGCTCACTTCACGGCGGATCAAGCCATCCTCGAGGCT (insertion)

Ref: ACTTCTACGAGACGTGCCCTTA-CCTCAAGTTCGCTCACTTCACGGCGAATCAAGCCATCCTCGAGGCTTTCGAAGG

L27 *BnaRGA*-sgRNA2

Allele1: CTTCGGCGTTGTGAAACAGATTAAACCGGTGATT-TCACGGTGGTTGAGCAAGAATCGAATCATAAC (deletion)

Allele2: CTTCGGCGTTGTGAAACAGATTAAACCGGTGATTTTCACGGTGGTTGAGCAAGAATCGAATCATAACG (WT)

Ref: CTTCGGCGTTGTGAAACAGATTAAACCGGTGATTTTCACGGTGGTTGAGCAAGAATCGAATCATAACGGT

L28 *BnaRGA*-sgRNA1

Allele1: TTCTACGAGACGTGCCCTTA--------TTCGCTCACTTCACGGCGAATCAAGCCATCCTCGAGGCTTTCG (deletion)

Allele2: TTCTACGAGACGTGCCCTTACCCTCAAGTTCGCTCACTTCACGGCGAATCAAGCCATCCTCGAGGCTTTCG (insertion)

Ref: TTCTACGAGACGTGCCCTTA-CCTCAAGTTCGCTCACTTCACGGCGAATCAAGCCATCCTCGAGGCTTTCG

L28 *BnaRGA*-sgRNA2

Allele1: TGGGATAGAGAAAGTCTTCGGCGTTGTGAAACAGATTAAACCGGTGATTTTCACGGTGGTTGAGCAAGAA (WT)

Allele2: TGGGATAGAGAAAGTCTTCGGCGTTGTGAAACAGATTAA--C--------TCACGGTGGTTGAGCAAGAA (deletion)

Ref: TGGGATAGAGAAAGTCTTCGGCGTTGTGAAACAGATTAAACCGGTGATTTTCACGGTGGTTGAGCAAGAA

L29 *BnaRGA*-sgRNA1

Allele1: TCTACGAGACGTGCCCTTA--CTCAAGTTCGCTCACTTCACGGCGAATCAAGCCATCCTCGAGGCTTTCGA (deletion)

Allele2: TCTACGAGACGTGCCCTTACCCTCAAGTTCGCTCACTTCACGGCGAATCAAGCCATCCTCGAGGCTTTCGA (insertion)

Ref: TCTACGAGACGTGCCCTTAC-CTCAAGTTCGCTCACTTCACGGCGAATCAAGCCATCCTCGAGGCTTTCGA

L30 *BnaRGA*-sgRNA1

Allele1: TTCTACGAGACGTGCCCTTA--CTCAAGTTCGCTCACTTCACGGCGAATCAAGCCATCCTCGAGGCTTTCG (deletion)

Allele2: TTCTACGAGACGTGCCCTTATCCTCAAGTTCGCTCACTTCACGGCGAATCAAGCCATCCTCGAGGCTTTCG (insertion)

Ref: TTCTACGAGACGTGCCCTTA-CCTCAAGTTCGCTCACTTCACGGCGAATCAAGCCATCCTCGAGGCTTTCG

L30 *BnaRGA*-sgRNA2

Allele1: GAGAAAGTCTTCGGCGTTGTGAAACAGATTAAACCGGTGATTTTCA---------------------------ACGGT (deletion)

Allele2: GAGAAAGTCTTCGGCGTTGTGAAACAGATTAAACCGGTGATTTTCAGCCTA (complicated variant)

Ref: GAGAAAGTCTTCGGCGTTGTGAAACAGATTAAACCGGTGATTTTCACGGTGGTTGAGCAAGAATCGAATCATAACGGT

L31 *BnaRGA*-sgRNA1

Allele1: GAGACGTGCCCTTATCCTCAAGTTCGCTCACTTCACGGCGAATCAAGCCATCCTCGAGGCTTTCG (insertion)

Allele2: GAGACGTGCCCTTAACCTCAAGTTCGCTCACTTCACGGCGAATCAAGCCATCCTCGAGGCTTTCG (insertion)

Ref: GAGACGTGCCCTTA-CCTCAAGTTCGCTCACTTCACGGCGAATCAAGCCATCCTCGAGGCTTTCG

L31 *BnaRGA*-sgRNA2

Allele1: GAGAAAGTCTTCGGCGTTGTGAAACAGATTAAACCGGTGATTTTCA---------------------------ACGGT (deletion)

Allele2: GAGAAAGTCTTCGGCGTTGTGAAACAGATTAAACCGGTGATTTTCAGCCTA (complicated variant)

Ref: GAGAAAGTCTTCGGCGTTGTGAAACAGATTAAACCGGTGATTTTCACGGTGGTTGAGCAAGAATCGAATCATAACGGT

L32 *BnaRGA*-sgRNA1

Allele1: TTCTACGAGACGTGCCCTTATCCTCAAGTTCGCTCACTTCACGGCGAATCAAGCCATCCTCGAGGCTTTCGA (insertion)

Ref: TTCTACGAGACGTGCCCTTA-CCTCAAGTTCGCTCACTTCACGGCGAATCAAGCCATCCTCGAGGCTTTCGA

L33 *BnaRGA*-sgRNA1

Allele1: TCTACGAGACGTGCCCTTA--CTCAAGTTCGCTCACTTCACGGCGAATCAAGCCATCCTCGAGGCTTTCGA (deletion)

Allele2: TCTACGAGACGTGCCCTTACCCTCAAGTTCGCTCACTTCACGGCGAATCAAGCCATCCTCGAGGCTTTCGA (insertion)

Ref: TCTACGAGACGTGCCCTTA-CCTCAAGTTCGCTCACTTCACGGCGAATCAAGCCATCCTCGAGGCTTTCGA

L34 *BnaRGA*-sgRNA1

Allele1: TTCTACGAGACGTGCCCTTA----CAAGTTCGCTCACTTCACGGCGAATCAAGCCATCCTCGAGGCTTTCG (deletion)

Allele2: TTCTACGAGACGTGCCCTTAACCTCAAGTTCGCTCACTTCACGGCGAATCAAGCCATCCTCGAGGCTTTCG (insertion)

Ref: TTCTACGAGACGTGCCCTTA-CCTCAAGTTCGCTCACTTCACGGCGAATCAAGCCATCCTCGAGGCTTTCG

L35 *BnaRGA*-sgRNA1

Allele1: ACTTCTACGAGACGTGCCCT-----CAAGTTCGCTCACTTCACGGCGAATCAAGCCATCCTCGAGGCTTT (deletion)

Allele2: ACTTCTACGAGACGTGCCCTTA--------TCGCTCACTTCACTTCACGGCGAATCAAGCCATCCTCGAGGCTTT (deletion)

Ref: ACTTCTACGAGACGTGCCCTTACCTCAAGTTCGCTCACTTCACGGCGAATCAAGCCATCCTCGAGGCTTT

L36 *BnaRGA*-sgRNA1

Allele1: TTCTACGAGACGTGCCCTTATCCTCAAGTTCGCTCACTTCACGGCGAATCAAGCCATCCTCGAGGCTTT (insertion)

Ref: TTCTACGAGACGTGCCCTTA-CCTCAAGTTCGCTCACTTCACGGCGAATCAAGCCATCCTCGAGGCTTT

L37 *BnaRGA*-sgRNA1

Allele1: TTCTACGAGACGTGCCCTTATCCTCAAGTTCGCTCACTTCACGGCGAATCAAGCCATCCTCGAGGCTTT (insertion)

Ref: TTCTACGAGACGTGCCCTTA-CCTCAAGTTCGCTCACTTCACGGCGAATCAAGCCATCCTCGAGGCTTT

L38 *BnaRGA*-sgRNA1

Allele1: TTCTACGAGACGTGCCCTTA---TTCGCTCACTTCACGGCGAATCAAGCCATCCTCGAGGCTTTCG (deletion)

Allele2: TTCTACGAGACGTGCCCTTAACCTCAAGTTCGCTCACTTCACGGCGAATCAAGCCATCCTCGAGGCTTTCG (insertion)

Ref: TTCTACGAGACGTGCCCTTA-CCTCAAGTTCGCTCACTTCACGGCGAATCAAGCCATCCTCGAGGCTTTCG

L39 *BnaRGA*-sgRNA1

Allele1: TTCTACGAGACGTGCCCTTA------AGTTCGCTCACTTCACGGCGAATCAAGCCATCCTCGAGGCTTTCG (deletion)

Allele2: TTCTACGAGACGTGCCCTTAACCTCAAGTTCGCTCACTTCACGGCGAATCAAGCCATCCTCGAGGCTTTCG (insertion)

Ref: TTCTACGAGACGTGCCCTTA-CCTCAAGTTCGCTCACTTCACGGCGAATCAAGCCATCCTCGAGGCTTTCG

L39 *BnaRGA*-sgRNA2

Allele1: GAGAAAGTCTTCGGCGTTGTGAAACAGATTAAACCGGTGATTTTCA---------------------------ACGGT (deletion)

Allele2: GAGAAAGTCTTCGGCGTTGTGAAACAGATTAAACCGGTGATTTTCAGCCTA (complicated variant)

Ref: GAGAAAGTCTTCGGCGTTGTGAAACAGATTAAACCGGTGATTTTCACGGTGGTTGAGCAAGAATCGAATCATAACGGT

L40 *BnaRGA*-sgRNA1

Allele1: GATGCACTTCTACGAGACGTGCCCTTA-CCATCAAGTTCGCTCACTTCACGGCGAAT (WT)

Allele2: GATGCACTTCTACGAGACGTGCCCTTACCCTCAAGTTCGCTCACTTCACGGCGAAT (insertion)

Ref: GATGCACTTCTACGAGACGTGCCCTTA-CCTCAAGTTCGCTCACTTCACGGCGAAT

L40 *BnaRGA*-sgRNA2

Allele1: TGGGATAGAGAAAGTCTTCGGCGTTGTGAAACAGATTAAACCGGTGATTTTCACGGTGGTTGAGCAAGAA (WT)

Allele2: TGGGATAGAGAAAGTCTTCGGCGTTGTGAAACAGATTAA--C--------TCACGGTGGTTGAGCAAGAA (deletion)

Ref: TGGGATAGAGAAAGTCTTCGGCGTTGTGAAACAGATTAAACCGGTGATTTTCACGGTGGTTGAGCAAGAA

L41 *BnaRGA*-sgRNA1

Allele1: TTCTACGAGACGTGCCCTTATCCTCAAGTTCGCTCACTTCACGGCGAAT (insertion)

Ref: TTCTACGAGACGTGCCCTTA-CCTCAAGTTCGCTCACTTCACGGCGAAT

L42 *BnaRGA*-sgRNA1

Allele1: TTCTACGAGACGTGCCCTTACCTCAAGTTCGCTCACT--ACGG (deletion)

Allele2: TTCTACGAGACGTGCCCTTACCTCAAGTTCGCTCACTGCCT (complicated variant)

Ref: TTCTACGAGACGTGCCCTTACCTCAAGTTCGCTCACTTCACGGCGAATCAAGCCATCCTCGAGGCTTTCGAAGGGAAGA

L43 *BnaRGA*-sgRNA1

Allele1: GATGCACTTCTACGAGACGTGCCCTTACCATCAAGTTCGCTCACTTCACGGCGAAT (WT)

Ref: GATGCACTTCTACGAGACGTGCCCTTACCTCAAGTTCGCTCACTTCACGGCGAAT

L44 *BnaRGA*-sgRNA1

Allele1: CTCCAGATGCACTTCTACGAGACGTGCCCTTA--CTCAAGTTCGCTCACTTCACGGCGAA (deletion)

Allele2: CTCCAGATGCACTTCTACGAGACGTGCCCTTAACCTCAAGTTCGCTCACTTCACGGCGAA (insertion)

Ref: CTCCAGATGCACTTCTACGAGACGTGCCCTTA-CCTCAAGTTCGCTCACTTCACGGCGAA

L45 *BnaRGA*-sgRNA1

Allele1: TTCTACGAGACGTGCCCTTACCTCAAGTTCGCTCACT--ACGG (deletion)

Allele2: TTCTACGAGACGTGCCCTTACCTCAAGTTCGCTCACTGCCT (complicated variant)

Ref: TTCTACGAGACGTGCCCTTACCTCAAGTTCGCTCACTTCACGGCGAATCAAGCCATCCTCGAGGCTTTCGAAGGGAAGA

L46 *BnaRGA*-sgRNA1

Allele1: TTCTACGAGACGTGCCCTTACCTCAAGTTCGCTCACT--ACGG (deletion)

Allele2: TTCTACGAGACGTGCCCTTACCTCAAGTTCGCTCACTGCCT (complicated variant)

Ref: TTCTACGAGACGTGCCCTTACCTCAAGTTCGCTCACTTCACGGCGAATCAAGCCATCCTCGAGGCTTTCGAAGGGAAGA

L46 *BnaRGA*-sgRNA2

Allele1: GGTGGGATAGAGAAAGTCTTCGGCGTTGTGAAACAGATTAAACCGGTGATTTTCACGGTGGTTGAGCAAG (WT)

Allele2: GGTGGGATAGAGAAAGTCTTCGGCGTTGTGAAACAGATTAAACCGGTGATT-TCACGGTGGTTGAGCAAG (deletion)

Ref: GGTGGGATAGAGAAAGTCTTCGGCGTTGTGAAACAGATTAAACCGGTGATTTTCACGGTGGTTGAGCAAG

L47 *BnaRGA*-sgRNA1

Allele1: TTCTACGAGACGTGCCCTTACCTCAAGTTCGCTCACT--ACGG (deletion)

Allele2: TTCTACGAGACGTGCCCTTACCTCAAGTTCGCTCACTGCCT (complicated variant)

Ref: TTCTACGAGACGTGCCCTTACCTCAAGTTCGCTCACTTCACGGCGAATCAAGCCATCCTCGAGGCTTTCGAAGGGAAGA

L47 *BnaRGA*-sgRNA2

Allele1: GTGGGATAGAGAAAGTCTTCGGCGTTGTGAAACAGATTAAACCGGTGATTTTCACGGTGGTTGAGCAAGA (WT)

Allele2: GTGGGATAGAGAAAGTCTTCGGCGTTGTGAAACAGATTAAACCGGTGA--TTCACGGTGGTTGAGCAAGA (deletion)

Ref: GTGGGATAGAGAAAGTCTTCGGCGTTGTGAAACAGATTAAACCGGTGATTTTCACGGTGGTTGAGCAAGA

L48 *BnaRGA*-sgRNA1

Allele1: TTCTACGAGACGTGCCCTTACCTCAAGTTCGCTCACT--ACGG (deletion)

Allele2: TTCTACGAGACGTGCCCTTACCTCAAGTTCGCTCACTGCCT (complicated variant)

Ref: TTCTACGAGACGTGCCCTTACCTCAAGTTCGCTCACTTCACGGCGAATCAAGCCATCCTCGAGGCTTTCGAAGGGAAGA

L48 *BnaRGA*-sgRNA2

Allele1: ATAGAGAAAGTCTTCGGCGTTGTGAAACAGATTAAACCGGTGATTTTCACGGTGGTTGAGCAAGAATCGA (WT)

Allele2: ATAGAGAAAGTCTTCGGCGTTGTGAAACAGATTAAACCGGT---------GGTGGTTGAGCAAGAATCGA (deletion)

Ref: ATAGAGAAAGTCTTCGGCGTTGTGAAACAGATTAAACCGGTGATTTTCACGGTGGTTGAGCAAGAATCGA

L49 *BnaRGA*-sgRNA1

Allele1: CTCCGATACTCTCCAGATGCACTTCTACGAGACGTGCCCTTA---------TCGCTCACTTCACGGCGAAT (deletion)

Allele2: CTCCGATACTCTCCAGATGCACTTCTACGAGACGTGCCCTTACCCTCAAGTTCGCTCACTTCACGGCGAAT (insertion)

Ref: CTCCGATACTCTCCAGATGCACTTCTACGAGACGTGCCCTTA-CCTCAAGTTCGCTCACTTCACGGCGAAT

L50 *BnaRGA*-sgRNA1

Allele1: CTTCTACGAGACGTGCCCTTACCCTCAAGTTCGCTCACTTCACGGCGAAT (insertion)

Ref: CTTCTACGAGACGTGCCCTTA-CCTCAAGTTCGCTCACTTCACGGCGAAT

L51 *BnaRGA*-sgRNA1

Allele1: TCTACGAGACGTGCCCTTAC---------GCTCACTTCACGGCGAATCA (deletion)

Allele2: TCTACGAGACGTGCCCTTAC---------------TTCACGGCGAATCAAGCCAT (deletion)

Ref: TCTACGAGACGTGCCCTTACCTCAAGTTCGCTCACTTCACGGCGAATCAAGCCATCCTCGAGGCTTTCGA

L52 *BnaRGA*-sgRNA1

Allele1: TTCTACGAGACGTGCCCTTA-----AGTTCGCTCACTTCACGGCGAATCAAGCCATCCTCGAGGCTTTCG (deletion)

Allele2: TTCTACGAGACGTGCCCTTA-------TTCGCTCACTTCACGGCGAATCAAGCCATCCTCGAGGCTTTCG (deletion)

Ref: TTCTACGAGACGTGCCCTTACCTCAAGTTCGCTCACTTCACGGCGAATCAAGCCATCCTCGAGGCTTTCG

L52 *BnaRGA*-sgRNA2

Allele1: GGTGGGATAGAGAAAGTCTTCGGCGTTGTGAAACAGATTAAACCGGTGATTTTCACGGTGGTTGAGCAAG (WT)

Allele2: GGTGGGATAGAGAAAGTCTTCGGCGTTGTGAAACAGATTAAACCGGTGA-TTTCACGGTGGTTGAGCAAG (deletion)

Ref: GGTGGGATAGAGAAAGTCTTCGGCGTTGTGAAACAGATTAAACCGGTGATTTTCACGGTGGTTGAGCAAG

L53 *BnaRGA*-sgRNA1

Allele1: CTTCTACGAGACGTGCCCTT----------TCGCTCACTTCACGGCGAATCAAGCCATCCTCGAGGCTTTC (deletion)

Allele2: CTTCTACGAGACGTGCCCTTACCCTCAAGTTCGCTCACTTCACGGCGAATCAAGCCATCCTCGAGGCTTTC (insertion)

Ref: CTTCTACGAGACGTGCCCTTA-CCTCAAGTTCGCTCACTTCACGGCGAATCAAGCCATCCTCGAGGCTTTC

L53 *BnaRGA*-sgRNA2

Allele1: TCTTGCTCAACCACCGTGAAAATCACCGGTTTAATCTGTTTCACAACGCCGAAGACTTTCTCTATCCCAC (WT)

Allele2: TCTTGCTCAACCACCGTGAAK-------GTTTAATCTGTTTCACAACGCCGAAGACTTTCTCTATCC (substitution and deletion)

Ref: TCTTGCTCAACCACCGTGAAAATCACCGGTTTAATCTGTTTCACAACGCCGAAGACTTTCTCTATCCCAC

L54 *BnaRGA*-sgRNA1

Allele1: CTTCTACGAGACGTGCCCTT----------TCGCTCACTTCACGG (deletion)

Allele2: CTTCTACGAGACGTGCCCTTACCCTCAAGTTCGCTCACTTCACGGCGAATCAAGCCATC (insertion)

Ref: CTTCTACGAGACGTGCCCTTA-CCTCAAGTTCGCTCACTTCACGGCGAATCAAGCCATCCTCGAGGCTTTC

L54 *BnaRGA*-sgRNA2

Allele1: TCTTGCTCAACCACCGTGAAAATCACCGGTTTAATCTGTTTCACAACGCCGAAGACTTTCTCTATCCCAC (WT)

Allele2: TCTTGCTCAACCACCGTGAA-------GGTTTAATCTGTTTCACAACGCCGAAGACTTTCTCTATCCCAC (deletion)

Ref: TCTTGCTCAACCACCGTGAAAATCACCGGTTTAATCTGTTTCACAACGCCGAAGACTTTCTCTATCCCAC

L55 *BnaRGA*-sgRNA1

Allele1: ACTTCTACGAGACGTGCCCT----CTCAAGTTCGCTCACTTCACGGCGAATCAAGCC (deletion)

Allele2: ACTTCTACGAGACGTGCCCTTACCCTCAAGTTCGCTCACTTCACGGCGAATCAAGCC (insertion)

Ref: ACTTCTACGAGACGTGCCCTTA-CCTCAAGTTCGCTCACTTCACGGCGAATCAAGCCATCCTCGAGGCTTT

BnaA6.RGA

L1 *BnaA6.RGA*-sgRNA1

Allele1: CTTGGGTTACAAGGTTAGGTCTTCGGAGATGGCTGAGGTTGCGTTGAAAC (WT)

Ref: CTTGGGTTACAAGGTTAGGTCTTCGGAGATGGCTGAGGTTGCGTTGAAAC

L2 *BnaA6.RGA*-sgRNA1

Allele1:	CTTGGGTTACAAGGTTAGGTCTTCGG (118-bp deletion) CTCGTGGCTTGATAA	(deletion)
Allele2:	CTTGGGTTACAAGGTTAGGTCTTCGGCAGATGGCTGAGGTTGCGTTGAAACTCGAGCAGCTTGAGAC	(insertion)
Allele3:	CTTGGGTTACAAGGTTAGGTCTTCGG-AGATGGCTGAGGTTGCGTTGAAACTCGAGCAGCTTGAGAC	(WT)
Ref:	CTTGGGTTACAAGGTTAGGTCTTCGG-AGATGGCTGAGGTTGCGTTGAAACTCGAGCAGCTTGAGACGATGATGGGT	

L3 *BnaA6.RGA*-sgRNA1

Allele1:	TTACAAGGTTAGGTCTTCGGTAGATGGCTGAGGTTGCGTTGAAACTCGAGCAGCTTGAGACGATGAT	(insertion)
Allele2:	TTACAAGGTTAGGTCTTCGG-AGATGGCTGAGGTTGCGTTGAAACTCGAGCAGCTTGAGACGATGAT	(WT)
Ref:	TTACAAGGTTAGGTCTTCGG-AGATGGCTGAGGTTGCGTTGAAACTCGAGCAGCTTGAGACGATGATGGGT	

L3 *BnaA6.RGA*-sgRNA2

Allele1:	TTCATTACAACCCCGCTGAGCTTTACCTCGTGGCTTGATAAC	(insertion)
Allele2:	TTCATTACAACCCCGCT----------TCGTGGCTTGATAAC	(deletion)
Ref:	TTCATTACAACCCCGCTGAGCTTTAC-TCGTGGCTTGATAACATGCTCACGGAGC	

L4 *BnaA6.RGA*-sgRNA1

Allele1:	CTTGGGTTACAAGGTTAGGTCTTCGGAGATGGCTGAGGTTGCGTTGAAAC	(WT)
Allele2:	CTTGGGTTACAAGGTTAGGTCT-------TGGCTGAGGTTGCGTTGAAAC	(deletion)
Allele3:	CTTGGGTTACAAGGTTAGGTC-----AGATGGCTGAGGTTGCGTTGAAACT	(deletion)
Ref:	CTTGGGTTACAAGGTTAGGTCTTCGGAGATGGCTGAGGTTGCGTTGAAAC	

L4 *BnaA6.RGA*-sgRNA2

Allele1:	TTCATTACAACCCCGCTGAGC-----TCGTGGCTTGATAACATGCTCACGGA	(deletion)
Allele2:	TTCATTACAACCCCGCTGAG------TCGTGGCTTGATAACATGCTCACGGA	(deletion)
Allele3:	TTCATTACAACCCCGCTGAGCTTTACTCGTGGCTTGATAACATGCTCACGGA	(WT)
Ref:	TTCATTACAACCCCGCTGAGCTTTACTCGTGGCTTGATAACATGCTCACGGA	

L5 *BnaA6.RGA*-sgRNA2

Allele1: ATACTGTTCATTACAACCCCGCTGAGC-----TCGTGGCTTGATAACATGCTCACGGAGCTTAA (deletion)

Allele2: ATACTGTTCATTACAACCCCGCTGAG------TCGTGGCTTGATAACATGCTCACGGAGC (deletion)

Ref: ATACTGTTCATTACAACCCCGCTGAGCTTTACTCGTGGCTTGATAACATGCTCACGGAGC

L6 *BnaA6.RGA*-sgRNA1

Allele1: TAGGAGTCTTGGGTTACAAGGTTAGGTC-----AGATGGCTGAGGTTGCGTTGAAACTCG (deletion)

Allele2: TAGGAGTCTTGGGTTACAAGGTTAGG---------ATGGCTGAGGTTGCGTTGAAACTCGAGC (deletion)

Ref: TAGGAGTCTTGGGTTACAAGGTTAGGTCTTCGGAGATGGCTGAGGTTGCGTTGAAACTCGAGCAG

L6 *BnaA6.RGA*-sgRNA2

Allele1: GACGGATACTGTTCATTACAACCCC--------------TCGTGGCTTGATAACATGCTCACGGAG (deletion)

Allele2: GACGGATACTGTTCATTACAACCCCGCTGAGCTTTACCTCGTGGCTTGATAACATGCTCAC (insertion)

Ref: GACGGATACTGTTCATTACAACCCCGCTGAGCTTTAC-TCGTGGCTTGATAACATGCTCACGGAG

L7 *BnaA6.RGA*-sgRNA1

Allele1: GCTTCTAGGAGTCTTGGGTTACAAGGTTAGGTCTTCGGAGATGGCTGAGGTTGCGTTGAAACTCG (WT)

Ref: GCTTCTAGGAGTCTTGGGTTACAAGGTTAGGTCTTCGGAGATGGCTGAGGTTGCGTTGAAACTCG

L8 *BnaA6.RGA*-sgRNA1

Allele1: GCTTCTAGGAGTCTTGGGTTACAAGGTTAGGTCTTCGGTAGATGGCTGAGGTTGCGTTGAAACTCG (insertion)

Allele2: GCTTCTAGGAGTCTTGGGTTACAAGGTTAGGTCTTCGGAAGATGGCTGAGGTTGCGTTGAAACTCG (insertion)

Ref: GCTTCTAGGAGTCTTGGGTTACAAGGTTAGGTCTTCGG-AGATGGCTGAGGTTGCGTTGAAACTCG

L9 *BnaA6.RGA*-sgRNA1

Allele1: GCTTCTAGGAGTCTTGGGTTACAAGGTTAGGTCTTCGGAGATGGCTGAGGTTGCGTTGAAACTCG (WT)

Allele2: GCTTCTAGGAGTCTTGGGTTACAAGGTTAGGTCTTCGGACATGGCTGAGGTTGCGTTGAAACTCG (substitution)

Ref: GCTTCTAGGAGTCTTGGGTTACAAGGTTAGGTCTTCGGAGATGGCTGAGGTTGCGTTGAAACTCG

L10 *BnaA6.RGA*-sgRNA1

Allele1: GCTTCTAGGAGTCTTGGGTTACAAGGTTAGGTCTTCGGAGATGGCTGAGGTTGCGTTGAAACTCG (WT)

Allele2: GCTTCTAGGAGTCTTGGGTTACAAGGTTAGGTCTTCGGACATGGCTGAGGTTGCGTTGAAACTCG (substitution)

Ref: GCTTCTAGGAGTCTTGGGTTACAAGGTTAGGTCTTCGGAGATGGCTGAGGTTGCGTTGAAACTCG

L11 *BnaA6.RGA*-sgRNA1

Allele1: GCTTCTAGGAGTCTTGGGTTACAAGGTTAGGTCTTCGGAGATGGCTGAGGTTGCGTTGAAACTCG (WT)

Ref: GCTTCTAGGAGTCTTGGGTTACAAGGTTAGGTCTTCGGAGATGGCTGAGGTTGCGTTGAAACTCG

L12 *BnaA6.RGA*-sgRNA1

Allele1: GGTCAAAGAAGAAGAAGACGACGAGCTTCTAGGAGTCTTGGGTTACAAGGTTAGGTCTTCGGAGATGG (WT)

Allele2: GGTCAAAGAAAAAAAAAACG (complicated variant)

Ref: GGTCAAAGAAGAAGAAGACGACGAGCTTCTAGGAGTCTTGGGTTACAAGGTTAGGTCTTCGGAGATGG

L13 *BnaA6.RGA*-sgRNA1

Allele1: GCTTCTAGGAGTCTTGGGTTACAAGGTTAGGTCTTCGGAGATGGCTGAGGTTGCGTTGAAACTCG (WT)

Ref: GCTTCTAGGAGTCTTGGGTTACAAGGTTAGGTCTTCGGAGATGGCTGAGGTTGCGTTGAAACTCG

L14 *BnaA6.RGA*-sgRNA1

Allele1: GCTTCTAGGAGTCTTGGGTTACAAGGTTAGGTCTTCGGAGATGGCTGAGGTTGCGTTGAAACTCG (WT)

Ref: GCTTCTAGGAGTCTTGGGTTACAAGGTTAGGTCTTCGGAGATGGCTGAGGTTGCGTTGAAACTCG

L15 *BnaA6.RGA*-sgRNA1

Allele1: GCTTCTAGGAGTCTTGGGTTACAAGGTTAGGTCTTCGGAGATGGCTGAGGTTGCGTTGAAACTCG (WT)

Ref: GCTTCTAGGAGTCTTGGGTTACAAGGTTAGGTCTTCGGAGATGGCTGAGGTTGCGTTGAAACTCG

L16 *BnaA6.RGA*-sgRNA1

Allele1: GCTTCTAGGAGTCTTGGGTTACAAGGTTAGGTCTTCGGAGATGGCTGAGGTTGCGTTGAAACTCG (WT)

Ref: GCTTCTAGGAGTCTTGGGTTACAAGGTTAGGTCTTCGGAGATGGCTGAGGTTGCGTTGAAACTCG

L17 *BnaA6.RGA*-sgRNA1

Allele1: GCTTCTAGGAGTCTTGGGTTACAAGGTTAGGTCTTCGGAGATGGCTGAGGTTGCGTTGAAACTCG (WT)

Ref: GCTTCTAGGAGTCTTGGGTTACAAGGTTAGGTCTTCGGAGATGGCTGAGGTTGCGTTGAAACTCG

L18 *BnaA6.RGA*-sgRNA1

Allele1: GCTTCTAGGAGTCTTGGGTTACAAGGTTAGGTCTTCGGAGATGGCTGAGGTTGCGTTGAAACTCG (WT)

Ref: GCTTCTAGGAGTCTTGGGTTACAAGGTTAGGTCTTCGGAGATGGCTGAGGTTGCGTTGAAACTCG

L19 *BnaA6.RGA*-sgRNA1

Allele1: GGTCAAAGAAGAAGAAGACGACGAGCTTCTAGGAGTCTTGGGTTACAAGGTTAGGTCTTCGGAGATGG (WT)

Allele2: GGTCAAAGAAAAAAAAAAACG (complicated variant)

Ref: GGTCAAAGAAGAAGAAGACGACGAGCTTCTAGGAGTCTTGGGTTACAAGGTTAGGTCTTCGGAGATGG

L20 *BnaA6.RGA*-sgRNA1

Allele1: GGTCAAAGAAGAAGAAGACGACGAGCTTCTAGGAGTCTTGGGTTACAAGGTTAGGTCTTCGGAGATGG (WT)

Allele2: GGTCAAAGAAAAAAAAAAACG (complicated variant)

Ref: GGTCAAAGAAGAAGAAGACGACGAGCTTCTAGGAGTCTTGGGTTACAAGGTTAGGTCTTCGGAGATGG

L21 *BnaA6.RGA*-sgRNA1

Allele1: GGTCAAAGAAGAAGAAGACGACGAGCTTCTAGGAGTCTTGGGTTACAAGGTTAGGTCTTCGGAGATGG (WT)

Allele2: GGTCAAAGAAAAAAAAAAACG (complicated variant)

Ref: GGTCAAAGAAGAAGAAGACGACGAGCTTCTAGGAGTCTTGGGTTACAAGGTTAGGTCTTCGGAGATGG

L22 *BnaA6.RGA*-sgRNA1

Allele1: GCTTCTAGGAGTCTTGGGTTACAAGGTTAGGTCTTCGGAGATGGCTGAGGTTGCGTTGAAACTCG (WT)

Allele2: GCTTCTAGGAGTCTTGGGTTACAAGGTTAGGTCTTCGGAAATGGCTGAGGTTGCGTTGAAACTCG (substitution)

Ref: GCTTCTAGGAGTCTTGGGTTACAAGGTTAGGTCTTCGGAGATGGCTGAGGTTGCGTTGAAACTCG

L23 *BnaA6.RGA*-sgRNA1

Allele1:	GGTCAAAGAAGAAGAAGACGACGAGCTTCTAGGAGTCTTGGGTTACAAGGTTAGGTCTTCGGAGATGG	(WT)
Allele2:	GGTCAAAGAAAAAAAAAACG	(complicated variant)
Ref:	GGTCAAAGAAGAAGAAGACGACGAGCTTCTAGGAGTCTTGGGTTACAAGGTTAGGTCTTCGGAGATGG	

L24 *BnaA6.RGA*-sgRNA1

Allele1:	GGTCAAAGAAGAAGAAGACGACGAGCTTCTAGGAGTCTTGGGTTACAAGGTTAGGTCTTCGGAGATGG	(WT)
Allele2:	GGTCAAAGAAAAAAAAAACG	(complicated variant)
Ref:	GGTCAAAGAAGAAGAAGACGACGAGCTTCTAGGAGTCTTGGGTTACAAGGTTAGGTCTTCGGAGATGG	

L25 *BnaA6.RGA*-sgRNA1

Allele1:	GGTCAAAGAAGAAGAAGACGACGAGCTTCTAGGAGTCTTGGGTTACAAGGTTAGGTCTTCGGAGATGG	(WT)
Allele2:	GGTCAAAGAAAAAAAAAACG	(complicated variant)
Ref:	GGTCAAAGAAGAAGAAGACGACGAGCTTCTAGGAGTCTTGGGTTACAAGGTTAGGTCTTCGGAGATGG	

L26 *BnaA6.RGA*-sgRNA1

Allele1:	GGTCAAAGAAGAAGAAGACGACGAGCTTCTAGGAGTCTTGGGTTACAAGGTTAGGTCTTCGGAGATGG	(WT)
Allele2:	GGTCAAAGAAAAAAAAAACG	(complicated variant)
Ref:	GGTCAAAGAAGAAGAAGACGACGAGCTTCTAGGAGTCTTGGGTTACAAGGTTAGGTCTTCGGAGATGG	

L27 *BnaRGA*-sgRNA1

Allele1:	CTTCTACGAGACTTGCCCTT---------TTCGCTCACTTCACGGCGAATCAGGCGATTCTCGAGGCTTTC	(deletion)
Allele2:	CTTCTACGAGACTTGCCCTTATCCTCAAGTTCGCTCACTTCACGGCGAATCAGGCGATTCTCGAGGCTTTC	(insertion)
Ref:	CTTCTACGAGACTTGCCCTTA-CTCAAGTTCGCTCACTTCACGGCGAATCAGGCGATTCTCGAGGCTTTC	

L28 *BnaRGA*-sgRNA1

Allele1:	ATGCACTTCTACGAGACTTG-----AC	(deletion)
Allele2:	ATGCACTTCTACGAGACTTGGC	(complicated variant)
Ref:	ATGCACTTCTACGAGACTTGCCCTTACCTCAAGTTCGCTCACTTCACGGCGAATCAGGCGATTCTCGAGG	

L29 *BnaRGA*-sgRNA1

Allele1: TTCTACGAGACTTGCCCTTAACCTCAAGTTCGCTCACTTCACGGCGAATCAGGCGATTCTCGAGG (insertion)

Ref: TTCTACGAGACTTGCCCTTA-CCTCAAGTTCGCTCACTTCACGGCGAATCAGGCGATTCTCGAGG

L30 *BnaRGA*-sgRNA1

Allele1: TTCTACGAGACTTGCCCTTATCCTCAAGTTCGCTCACTTCACGGCGAATCAGGCGATTCTCGAGG (insertion)

Allele2: TTCTACGAGACTTGCCCTTACCCTCAAGTTCGCTCACTTCACGGCGAATCAGGCGATTCTCGAGG (insertion)

Ref: TTCTACGAGACTTGCCCTTA-CCTCAAGTTCGCTCACTTCACGGCGAATCAGGCGATTCTCGAGG

L30 *BnaRGA*-sgRNA2

Allele1: TCTTGCTCAACAACCGTGAAAATCACCGGTTTAATCTGTTTCACAACGCCGAAGACTTTCTCTATCCCAC (WT)

Allele2: TCTTGCTCAACAACCGTGA--ATCACCGGTTTAATCTGTTTCACAACGCCGAAGACTTTCTCTATCCCAC (deletion)

Ref: TCTTGCTCAACAACCGTGAAAATCACCGGTTTAATCTGTTTCACAACGCCGAAGACTTTCTCTATCCCAC

L31 *BnaRGA*-sgRNA1

Allele1: TTCTACGAGACTTGCCCTTATCCTCAAGTTCGCTCACTTCACGGCGAATCAGGCGATTCTCGAGG (insertion)

Allele2: TTCTACGAGACTTGCCCTTACCCTCAAGTTCGCTCACTTCACGGCGAATCAGGCGATTCTCGAGG (insertion)

Ref: TTCTACGAGACTTGCCCTTA-CCTCAAGTTCGCTCACTTCACGGCGAATCAGGCGATTCTCGAGG

L31 *BnaRGA*-sgRNA2

Allele1: TTCTTGCTCAACAACCGTGA--ATCACCGGTTTAATCTGTTTCACAACGCCGAAGACTTTCTCTATCCCA (deletion)

Allele2: TTCTTGCTCAACAACCGTGATMAYTGGCSGRTTTW (complicated variant)

Ref: TTCTTGCTCAACAACCGTGAAAATCACCGGTTTAATCTGTTTCACAACGCCGAAGACTTTCTCTATCCCA

L32 *BnaRGA*-sgRNA1

Allele1: TTCTACGAGACTTGCCCTTA-------TTTCGCTCACTTCACGGCGAATCAGGCGATTCTCGAGGCTTTCG (insertion and deletion)

Allele2: TTCTACGAGACTTGCCCTTA---------TCGCTCACTTCACGGCGAATCAGGCGATTCTCGAGGCTTTCG (deletion)

Ref: TTCTACGAGACTTGCCCTTA-CCTCAAGTTCGCTCACTTCACGGCGAATCAGGCGATTCTCGAGGCTTTCG

L33 *BnaRGA*-sgRNA1

Allele1: CTACGAGACTTGCCCTTACCCTCAAGTTCGCTCACTTCACGGCGAATCAGGCGATTCTCGAGGCTTTCG (insertion)

Allele2: CTACGAGACTTGCCCTTA-CCTCAAGTTCGCTCACTTCACGGCGAATCAGGCGATTCTCGAGGCTTTCG (WT)

Ref: CTACGAGACTTGCCCTTA-CCTCAAGTTCGCTCACTTCACGGCGAATCAGGCGATTCTCGAGGCTTTCG

L34 *BnaRGA*-sgRNA1

Allele1: CTACGAGACTTGCCCTTACCCTCAAGTTCGCTCACTTCACGGCGAATCAGGCGATTCTCGAGGCTTTCG (insertion)

Ref: CTACGAGACTTGCCCTTA-CCTCAAGTTCGCTCACTTCACGGCGAATCAGGCGATTCTCGAGGCTTTCG

L35 *BnaRGA*-sgRNA1

Allele1: CTTCTACGAGACTTGCCCTT----CAAGTTCGCTCACTTCACGGCGAATCAGGCGATTCTCGAGGCTTTC (deletion)

Allele2: CTTCTACGAGACTTGCCCTT-----AAGTTCGCTCACTTCACGGCGAATCAGGCGATTCTCGAGGCTTTC (deletion)

Ref: CTTCTACGAGACTTGCCCTTACCTCAAGTTCGCTCACTTCACGGCGAATCAGGCGATTCTCGAGGCTTTC

L36 *BnaRGA*-sgRNA1

Allele1: TTCTACGAGACTTGCCCTTA-----AGTTCGCTCACTTCACGGCGAATCAGGCGATTCTCGAGGCTTTC (deletion)

Ref: TTCTACGAGACTTGCCCTTACCTCAAGTTCGCTCACTTCACGGCGAATCAGGCGATTCTCGAGGCTTTC

L37 *BnaRGA*-sgRNA1

Allele1: TTCTACGAGACTTGCCCTTA-TCCTCAAGTTCGCTCACTTCACGGCGAATCAGGCGATTCTCGAG (insertion and deletion)

Allele2: TTCTACGAGACTTGCCCTTA------------TCTCACTTCACGGCGAATCAGGCGATTCTCGAGGCTTTCG (deletion)

Ref: TTCTACGAGACTTGCCCTTA-CCTCAAGTTCGCTCACTTCACGGCGAATCAGGCGATTCTCGAGGCTTTCG

L38 *BnaRGA*-sgRNA1

Allele1: TTCTACGAGACTTGCCCTTA------GTTCGCTCACTTCACGGCGAATCAGGCGATTCTCGAGGCTTTCG (deletion)

Allele2: TTCTACGAGACTTGCCCTTAC-------TCGCTCACTTCACGGCGAATCAGGCGATTCTCGAGGCTTTCG (deletion)

Ref: TTCTACGAGACTTGCCCTTACCTCAAGTTCGCTCACTTCACGGCGAATCAGGCGATTCTCGAGGCTTTCG

L39 *BnaRGA*-sgRNA1

Allele1: ATGCACTTCTACGAGACTTG-----AC (deletion)

Allele2: ATGCACTTCTACGAGACTTGGC (complicated variant)

Ref: ATGCACTTCTACGAGACTTGCCCTTACCTCAAGTTCGCTCACTTCACGGCGAATCAGGCGATTCTCGAGG

L40 *BnaRGA*-sgRNA1

Allele1: TCTACGAGACTTGCCCTTA-CTCAAGTTCGCTCACTTCACGGCGAATCAGGCGATTCTCGAGG (deletion)

Ref: TCTACGAGACTTGCCCTTACCTCAAGTTCGCTCACTTCACGGCGAATCAGGCGATTCTCGAGG

L41 *BnaRGA*-sgRNA1

Allele1: ACTCTTTATCCGATACTCTC---------------------------------AAGTTCGCTCACTTCACGGCGAATCAGGCGATTCTCGA (deletion)

Allele2: ACTCTTTATCCGATACTCTCCAGATGCACTTCTACGAGACTTGCCCTT-----AAGTTCGCTC (deletion)

Ref: ACTCTTTATCCGATACTCTCCAGATGCACTTCTACGAGACTTGCCCTTACCTCAAGTTCGCTCACTTCAC

L42 *BnaRGA*-sgRNA1

Allele1: ATGCACTTCTACGAGACTTG-----AC (deletion)

Allele2: ATGCACTTCTACGAGACTTGGC (complicated variant)

Ref: ATGCACTTCTACGAGACTTGCCCTTACCTCAAGTTCGCTCACTTCACGGCGAATCAGGCGATTCTCGAGG

L43 *BnaRGA*-sgRNA1

Allele1: ATGCACTTCTACGAGACTTGCCCTTACCTCAAGTTCGCTCACTTCACGGCGAATCAGGCGATTCTCGAGG (WT)

Ref: ATGCACTTCTACGAGACTTGCCCTTACCTCAAGTTCGCTCACTTCACGGCGAATCAGGCGATTCTCGAGG

L44 *BnaRGA*-sgRNA1

Allele1: TTCTACGAGACTTGCCCTTAACCTCAAGTTCGCTCACTTCACGGCGAATCAGGCGATTCTCGAGG (insertion)

Ref: TTCTACGAGACTTGCCCTTA-CCTCAAGTTCGCTCACTTCACGGCGAATCAGGCGATTCTCGAGG

L45 *BnaRGA*-sgRNA1

Allele1: ACTTCTACGAGACTTGCCCT------CAAGTTCGCTCACTTCACGGCGAATCAGGCGATTCTCGAGGCTTT (deletion)

Allele2: ACTTCTACGAGACTTGCCCTTAGCCTCAAGTTCGCTCACTTCACGGCGAATCAGGCGATTCTCGAGGCTTT (insertion)

Ref: ACTTCTACGAGACTTGCCCTTA-CCTCAAGTTCGCTCACTTCACGGCGAATCAGGCGATTCTCGAGGCTTT

L45 *BnaRGA*-sgRNA2

Allele1: GATTAAACCGGTGATTTTCAAGGGT (insertion)

Allele2: GATTAAACCGGTGATTTTCAGCCTA (complicated variant)

Ref: GATTAAACCGGTGATTTTCACGGTTGTTGAGCAAGAATCGAATCATAACGGTCCGGTTTTCTTAGACCGG

L46 *BnaRGA*-sgRNA1

Allele1: TTCTACGAGACTTGCCCTTA------AGTTCGCTCACTTCACGGCGAATCAGGCGATTCTCGAGGCTTTCG (deletion)

Allele2: TTCTACGAGACTTGCCCTTATCCTCAAGTTCGCTCACTTCACGGCGAATCAGGCGATTCTCGAGGCTTTCG (insertion)

Ref: TTCTACGAGACTTGCCCTTA-CCTCAAGTTCGCTCACTTCACGGCGAATCAGGCGATTCTCGAGGCTTTCG

L46 *BnaRGA*-sgRNA2

Allele1: CTTGCTCAACAACCGTGA-AATCACCGGTTTAATCTGTTTCACAACGCCGAAGACTTTCTCTATCCCACC (deletion)

Allele2: CTTGCTCAACAACCGTGAAAATCCCCGGTTTAATCTGTTTCACAACGCCGAAGACTTTCTCTATCC (complicated variant)

Ref: CTTGCTCAACAACCGTGAAAATCACCGGTTTAATCTGTTTCACAACGCCGAAGACTTTCTCTATCCCACC

L47 *BnaRGA*-sgRNA1

Allele1: ATGCACTTCTACGAGACTTG-----AC (deletion)

Allele2: ATGCACTTCTACGAGACTTGGC (complicated variant)

Ref: ATGCACTTCTACGAGACTTGCCCTTACCTCAAGTTCGCTCACTTCACGGCGAATCAGGCGATTCTCGAGG

L47 *BnaRGA*-sgRNA2

Allele1: CTTGCTCAACAACCGTGA-AATCACCGGTTTAATCTGTTTCACAACGCCGAAGACTTTCTCTATCCCACC (deletion)

Allele2: CTTGCTCAACAACCGTGAAAATCCCCGGTTTAATCTGTTTCACAACGCCGAAGACTTTCTCTATCC (complicated variant)

Ref: CTTGCTCAACAACCGTGAAAATCACCGGTTTAATCTGTTTCACAACGCCGAAGACTTTCTCTATCCCACC

L48 *BnaRGA*-sgRNA1

Allele1: ATGCACTTCTACGAGACTTG-----AC (deletion)

Allele2: ATGCACTTCTACGAGACTTGGC (complicated variant)

Ref: ATGCACTTCTACGAGACTTGCCCTTACCTCAAGTTCGCTCACTTCACGGCGAATCAGGCGATTCTCGAGG

L49 *BnaRGA*-sgRNA1

Allele1:	CTTCTACGAGACTTGCCCTT-CCTCAAGTTCGCTCACTTCACGGCGAATCAGGCGATTCTCGAGGCTTTC	(deletion)
Allele2:	CTTCTACGAGACTTGCCCTT------AGTTCGCTCACTTCACGGCGAATCAGGCGATTCTCGAGGCTTTC	(deletion)
Ref:	CTTCTACGAGACTTGCCCTTACCTCAAGTTCGCTCACTTCACGGCGAATCAGGCGATTCTCGAGGCTTTC	

L50 *BnaRGA*-sgRNA1

Allele1:	TTCTACGAGACTTGCCCTTA------GTTCGCTCACTTCACGGCGAATCAGGCGATTCTCGAGGCTTTCG	(deletion)
Allele2:	TTCTACGAGACTTGCCCTTAC-------TCGCTCACTTCACGGCGAATCAGGCGATTCTCGAGGCTTTCG	(deletion)
Ref:	TTCTACGAGACTTGCCCTTACCTCAAGTTCGCTCACTTCACGGCGAATCAGGCGATTCTCGAGGCTTTCG	

L51 *BnaRGA*-sgRNA1

Allele1:	TTCTACGAGACTTGCCCTTA--TCAAGTTCGCTCACTTCACGGCGAATCAGGCGATTCTCGAGGCTTTCG	(deletion)
Ref:	TTCTACGAGACTTGCCCTTACCTCAAGTTCGCTCACTTCACGGCGAATCAGGCGATTCTCGAGGCTTTCG	

L52 *BnaRGA*-sgRNA2

Allele1:	GGCGTTGTGAAACAG------------------ACGGT	(deletion)
Allele2:	GGCGTTGTGAAACAGGCCTA (complicated variant)	
Ref:	GGCGTTGTGAAACAGATTAAACCGGTGATTTTCACGGTTGTTGAGCAAGAATCGAATCATAACGG	

L53 *BnaRGA*-sgRNA2

Allele1:	CTTGCTCAACAACCGTGA--AATCACCGGTTTAATCTGTTTCACAACGCCGAAGACTTTCTCTATCCCACC	(deletion)
Allele2:	CTTGCTCAACAACCGTGAAAAATCACCGGTTTAATCTGTTTCACAACGCCGAAGACTTTCTCTATCCCACC	(insertion)
Ref:	CTTGCTCAACAACCGTGA-AAATCACCGGTTTAATCTGTTTCACAACGCCGAAGACTTTCTCTATCCCACC	

L54 *BnaRGA*-sgRNA1

Allele1:	GCACTTCTACGAGACTTGCC-----------TTCGCTCACTTCACGGCGAATCAGGCGATTCTCGAGGCT	(deletion)
Allele2:	GCACTTCTACGAGACTTGCCCTTA--------TCGCTCACTTCACGGCGAATCAGGCGATTCTCGAGGCT	(deletion)
Ref:	GCACTTCTACGAGACTTGCCCTTACCTCAAGTTCGCTCACTTCACGGCGAATCAGGCGATTCTCGAGGCT	

L54 *BnaRGA*-sgRNA2

Allele1: CTTGCTCAACAACCGTGA-AATCACCGGTTTAATCTGTTTCACAACGCCGAAGACTTTCTCTATCCCACC (deletion)

Allele2: CTTGCTCAACAACCGTGAAAATCACCGGTTTATTC (complicated variant)

Ref: CTTGCTCAACAACCGTGAAAATCACCGGTTTAATCTGTTTCACAACGCCGAAGACTTTCTCTATCCCACC

L55 *BnaRGA*-sgRNA1

Allele1: TTCTACGAGACTTGCCCTTA-CCTCAAGTTCGCTCACTTCACGGCGAATCAGGCGATTCTCGAGGCTTTCG (WT)

Allele2: TTCTACGAGACTTGCCCTTAACCTCAAGTTCGCTCACTTCACGGCGAATCAGGCGATTCTCGAGGCTTTCG (insertion)

Ref: TTCTACGAGACTTGCCCTTA-CCTCAAGTTCGCTCACTTCACGGCGAATCAGGCGATTCTCGAGGCTTTCG

BnaA6.DA1

L1 *BnaA6.DA1*-sgRNA2

Allele1: AGTTCAACATATCCTGTATTGCGTGGCTGCACATA (WT)

Allele2: AGTTCAACATATCCTGTATTTGCTGGCTTGCCCTT (complicated variant)

Ref: AGTTCAACATATCCTGTATTGCGTGGCTGCACATAAACGAAAGCTGGCAAATTAGTATTGACTTTGGGAA

L2 *BnaA6.DA1*-sgRNA2

Allele: CTAATTTGCCAGCTTTCGTTTATGTGCAGCCACGCAATACAGGATATGTTGAACTTAACGATGGACGGAAACTTTGC (WT)

Ref: CTAATTTGCCAGCTTTCGTTTATGTGCAGCCACGCAATACAGGATATGTTGAACTTAACGATGGACGGAAACTTTGC

L3 *BnaA6.DA1*-sgRNA2

Allele1: AGTTCAACATATCCTGTATTTGCGTGGCTGCACATAAACGAAAGCTGGCAAATTAGTATTGACTTTGGGAA (insertion)

Ref: AGTTCAACATATCCTGTA-TTGCGTGGCTGCACATAAACGAAAGCTGGCAAATTAGTATTGACTTTGGGAA

L6 *BnaA6.DA1*-sgRNA2

Allele1: TAAGTTCAACATATCCTGTA-TTGCGTGGCTGCACATAAACGAAAGCTGGCAAATTAGTATTGACTTTGGG (WT)

Allele2: TAAGTTCAACATATCCTGTAATTGCGTGGCTGCACATAAACGAAAGCTGGCAAATTAGTATTGACTTTGGG (insertion)

Ref: TAAGTTCAACATATCCTGTA-TTGCGTGGCTGCACATAAACGAAAGCTGGCAAATTAGTATTGACTTTGGG

L8 *BnaA6.DA1*-sgRNA2

Allele1:	TAAGTTCAACATATCCTGTA-TTGCGTGGCTGCACATAAACGAAAGCTGGCAAATTAGTATTGACTTTGGG	(WT)
Allele2:	TAAGTTCAACATATCCTGTAATTGCGTGGCTGCACATAAACGAAAGCTGGCAAATTAGTATTGACTTTGGG	(insertion)
Ref:	TAAGTTCAACATATCCTGTA-TTGCGTGGCTGCACATAAACGAAAGCTGGCAAATTAGTATTGACTTTGGG	

L10 *BnaA6.DA1*-sgRNA2

Allele1:	AGTTCAACATATCCTGTATTGCGTGGCTGCACATA	(WT)
Allele2:	AGTTCAACATATCCTGTATTTGCTGGCTTGCCCTT	(complicated variant)
Ref:	AGTTCAACATATCCTGTATTGCGTGGCTGCACATAAACGAAAGCTGGCAAATTAGTATTGACTTTGGGAA	

L14 *BnaA6.DA1*-sgRNA2

Allele1:	AGTTCAACATATCCTGTATTGCGTGGCTGCACATAAACGAAAGCTGGCAAATTAGTATTGACTTTGGGAA	(WT)
Allele2:	AGTTCAACATATCCTGTATTTGCTGGCTTGCCCTT	(complicated variant)
Ref:	AGTTCAACATATCCTGTATTGCGTGGCTGCACATAAACGAAAGCTGGCAAATTAGTATTGACTTTGGGAA	

L15 *BnaA6.DA1*-sgRNA2

Allele1:	CTAATTTGCCAGCTTTCGTTTATGTGCAGCCACGCAATACAGGATATGTTGAACTTAACGATGGACGGAAACTTTGC	(WT)
Ref:	CTAATTTGCCAGCTTTCGTTTATGTGCAGCCACGCAATACAGGATATGTTGAACTTAACGATGGACGGAAACTTTGC	

L16 *BnaA6.DA1*-sgRNA2

Allele:	CTAATTTGCCAGCTTTCGTTTATGTGCAGCCACGCAATACAGGATATGTTGAACTTAACGATGGACGGAAACTTTGC	(WT)
Ref:	CTAATTTGCCAGCTTTCGTTTATGTGCAGCCACGCAATACAGGATATGTTGAACTTAACGATGGACGGAAACTTTGC	

L17 *BnaA6.DA1*-sgRNA2

Allele1:	AAGTTCAACATATCCTGTA-TGCGTGGCTGCACATAAACGAAAGCTGGCAAATTAGTATTGACTTTGGGAA	(deletion)
Ref:	AAGTTCAACATATCCTGTATTGCGTGGCTGCACATAAACGAAAGCTGGCAAATTAGTATTGACTTTGGGAA	

L18 *BnaA6.DA1*-sgRNA2

Allele:	CTAATTTGCCAGCTTTCGTTTATGTGCAGCCACGCAATACAGGATATGTTGAACTTAACGATGGACGGAAACTTTGC	(WT)
Ref:	CTAATTTGCCAGCTTTCGTTTATGTGCAGCCACGCAATACAGGATATGTTGAACTTAACGATGGACGGAAACTTTGC	

L19 *BnaA6.DA1*-sgRNA2

Allele1: AGTTCAACATATCCTGTATTGCGTGGCTGCACATAAACGAAAGCTGGCAAATTAGTATTGACTTTGGGAA (WT)

Allele2: AGTTCAACATATCCTGTATTTGCTGGCTTGCCCTT (complicated variant)

Ref: AGTTCAACATATCCTGTATTGCGTGGCTGCACATAAACGAAAGCTGGCAAATTAGTATTGACTTTGGGAA

L20 *BnaA6.DA1*-sgRNA2

Allele: CTAATTTGCCAGCTTTCGTTTATGTGCAGCCACGCAATACAGGATATGTTGAACTTAACGATGGACGGAAACTTTGC (WT)

Ref: CTAATTTGCCAGCTTTCGTTTATGTGCAGCCACGCAATACAGGATATGTTGAACTTAACGATGGACGGAAACTTTGC

L21 *BnaA6.DA1*-sgRNA2

Allele1: AAGTTCAACATATCCTGTA--TGCGTGGCTGCACATAAACGAAAGCTGGCAAATTAGTATTGACTTTGGGA (deletion)

Allele2: AAGTTCAACATATCCTGTATTTGCGTGGCTGCACATAAACGAAAGCTGGCAAATTAGTATTGACTTTGGGA (insertion)

Ref: AAGTTCAACATATCCTGTA-TTGCGTGGCTGCACATAAACGAAAGCTGGCAAATTAGTATTGACTTTGGGA

L22 *BnaA6.DA1*-sgRNA2

Allele1: AAGTTCAACATATCCTGTA--TGCGTGGCTGCACATAAACGAAAGCTGGCAAATTAGTATTGACTTTGGGA (deletion)

Allele2: AAGTTCAACATATCCTGTATTTGCGTGGCTGCACATAAACGAAAGCTGGCAAATTAGTATTGACTTTGGGA (insertion)

Ref: AAGTTCAACATATCCTGTA-TTGCGTGGCTGCACATAAACGAAAGCTGGCAAATTAGTATTGACTTTGGGA

L23 *BnaA6.DA1*-sgRNA2

Allele1: TAAGTTCAACATATCCTGTACTTGCGTGGCTGCACATAAACGAAAGCTGGCAAATTAGTATTGACTTTGGGA (insertion)

Allele2: TAAGTTCAACATATCCTGTAATTGCGTGGCTGCACATAAACGAAAGCTGGCAAATTAGTATTGACTTTGGGA (insertion)

Ref: TAAGTTCAACATATCCTGTA-TTGCGTGGCTGCACATAAACGAAAGCTGGCAAATTAGTATTGACTTTGGGA

L24 *BnaA6.DA1*-sgRNA2

Allele1: AAGTTCAACATATCCTGTATTGCGTGGCTGCACATAAACGAAAGCTGGCAAATTAGTATTGACTTTGGGA (WT)

Allele2: AAGTTCAACATATCCTGTA-TGCGTGGCTGCACATAAACGAAAGCTGGCAAATTAGTATTGACTTTGGGA (deletion)

Ref: AAGTTCAACATATCCTGTATTGCGTGGCTGCACATAAACGAAAGCTGGCAAATTAGTATTGACTTTGGGA

L25 *BnaA6.DA1*-sgRNA2

Allele:	CTAATTTGCCAGCTTTCGTTTATGTGCAGCCACGCAATACAGGATATGTTGAACTTAACGATGGACGGAAACTTTGC	(WT)
Ref:	CTAATTTGCCAGCTTTCGTTTATGTGCAGCCACGCAATACAGGATATGTTGAACTTAACGATGGACGGAAACTTTGC	

L26 *BnaA6.DA1*-sgRNA2

Allele1:	CATCGTTAAGTTCAACATATCCTGTATTGCGTGGCTGCACATAAACGAAAGCTGGCAAATTAGTATTGAC	(WT)
Allele2:	CATCGTTAAGTTCAACATAT (40-bp deletion) TAGTATTGACTTTGG	(deletion)
Ref:	CATCGTTAAGTTCAACATATCCTGTATTGCGTGGCTGCACATAAACGAAAGCTGGCAAATTAGTATTGAC	

L27 *BnaA6.DA1*-sgRNA2

Allele1:	AGTTCAACATATCCTGTATTGCGTGGCTGCACATAAACGAAAGCTGGCAAATTAGTATTGACTTTGGGAA	(WT)
Allele2:	AGTTCAACATATCCTGTATTTGCTGGCTTGCCCTT	(complicated variant)
Ref:	AGTTCAACATATCCTGTATTGCGTGGCTGCACATAAACGAAAGCTGGCAAATTAGTATTGACTTTGGGAA	

L28 *BnaA6.DA1*-sgRNA2

Allele1:	TAAGTTCAACATATCCTGTA--TGCGTGGCTGCACATAAACGAAAGCTGGCAAATTAGTATTGACTTTGGG	(deletion)
Allele2:	TAAGTTCAACATATCCTGTAATTGCGTGGCTGCACATAAACGAAAGCTGGCAAATTAGTATTGACTTTGGG	(insertion)
Ref:	TAAGTTCAACATATCCTGTA-TTGCGTGGCTGCACATAAACGAAAGCTGGCAAATTAGTATTGACTTTGGG	

L29 *BnaA6.DA1*-sgRNA2

Allele1:	TATGTGCAGCCACGCAATAC------------------------ACGGA	(deletion)
Allele2:	TATGTGCAGCCACGCAATAC (35-bp deletion) GCCTT	(deletion)
Ref:	TATGTGCAGCCACGCAATACAGGATATGTTGAACTTAACGATGGACGGAAACTTTGCCTTGAATGTCTGG	

L30 *BnaA6.DA1*-sgRNA2

Allele1:	TATGTGCAGCCACGCAATAC------------------------ACGGA	(deletion)
Allele2:	TATGTGCAGCCACGCAATAC (35-bp deletion) GCCTT	(deletion)
Ref:	TATGTGCAGCCACGCAATACAGGATATGTTGAACTTAACGATGGACGGAAACTTTGCCTTGAATGTCTGG	

L31 *BnaA6.DA1*-sgRNA2

Allele1: TAAGTTCAACATATCCTGTAATTGCGTGGCTGCACATAAACGAAAGCTGGCAAATTAGTATTGACTTTGGG (insertion)

Ref: TAAGTTCAACATATCCTGTA-TTGCGTGGCTGCACATAAACGAAAGCTGGCAAATTAGTATTGACTTTGGG

L32 *BnaA6.DA1*-sgRNA2

Allele1: TAAGTTCAACATATCCTGTA---GCGTGGCTGCACATAAACGAAAGCTGGCAAATTAGTATTGACTTTGGG (deletion)

Allele2: TAAGTTCAACATATCCTGTAATTGCGTGGCTGCACATAAACGAAAGCTGGCAAATTAGTATTGACTTTGGG (insertion)

Ref: TAAGTTCAACATATCCTGTA-TTGCGTGGCTGCACATAAACGAAAGCTGGCAAATTAGTATTGACTTTGGG

L33 *BnaA6.DA1*-sgRNA2

Allele1: TAAGTTCAACATATCCTGTAATTGCGTGGCTGCACATAAACGAAAGCTGGCAAATTAGTATTGACTTTGGG (insertion)

Ref: TAAGTTCAACATATCCTGTA-TTGCGTGGCTGCACATAAACGAAAGCTGGCAAATTAGTATTGACTTTGGG

L34 *BnaA6.DA1*-sgRNA2

Allele: CTAATTTGCCAGCTTTCGTTTATGTGCAGCCACGCAATACAGGATATGTTGAACTTAACGATGGACGGAAACTTTGC (WT)

Ref: CTAATTTGCCAGCTTTCGTTTATGTGCAGCCACGCAATACAGGATATGTTGAACTTAACGATGGACGGAAACTTTGC

L35 *BnaA6.DA1*-sgRNA2

Allele: CTAATTTGCCAGCTTTCGTTTATGTGCAGCCACGCAATACAGGATATGTTGAACTTAACGATGGACGGAAACTTTGC (WT)

Ref: CTAATTTGCCAGCTTTCGTTTATGTGCAGCCACGCAATACAGGATATGTTGAACTTAACGATGGACGGAAACTTTGC

L36 *BnaA6.DA1*-sgRNA2

Allele1: TATGTGCAGCCACGCAATAC------------------------ACGGA (deletion)

Allele2: TATGTGCAGCCACGCAATAC (35-bp deletion) GCCTT (deletion)

Ref: TATGTGCAGCCACGCAATACAGGATATGTTGAACTTAACGATGGACGGAAACTTTGCCTTGAATGTCTGG

BnaC5.DA1

L1 *BnaC5.DA1*-sgRNA1

Allele1: GTTAAATAACTTATCAAGAACACCTTAGGATTTGATGCTGGCTGCAATATGGAGATTGGGCATGGAAGA (insertion)

Allele2: GTTAAATAACTTATCAAGAACACCTTAGGATTTGTTGCTGGCTGCAATATGGAGATTGGGCATGGAAGA (insertion)

Ref: GTTAAATAACTTATCAAGAACACCTTAGGATTTG-TGCTGGCTGCAATATGGAGATTGGGCATGGAAGA

L1 *BnaC5.DA1*-sgRNA2

Allele1: GTCAACTTTCGTTTTTTTTTTCTTTGTAGCCACGG (WT)

Allele2: GTCAACTTTCGTTTTTTTTTTCCTTTGAACCCCCGA (complicated variant)

Ref: GTTTTTTTTTTCTTTGTAGCCACGGAATACAGGATATGTTGAACTTAACGATGGACGGAAACTTTGCCTGGAGTGTCTGGACT

L2 *BnaC5.DA1*-sgRNA1

Allele1: GTTAAATAACTTATCAAGAACACCTTAGGATTTGTGCTGGCTGCAATATGGAGATTGGGCATGGAAGA (WT)

Ref: GTTAAATAACTTATCAAGAACACCTTAGGATTTGTGCTGGCTGCAATATGGAGATTGGGCATGGAAGA

L2 *BnaC5.DA1*-sgRNA2

Allele1: GTCAACTTTCGTTTTTTTTTTCTTTGTAGCCACGG (WT)

Allele2: GTCAACTTTCGTTTTTTTTTTCCTTTGAACCCCCGA (complicated variant)

Ref: GTTTTTTTTTTCTTTGTAGCCACGGAATACAGGATATGTTGAACTTAACGATGGACGGAAACTTTGCCTGGAGTGTCTGGACT

L3 *BnaC5.DA1*-sgRNA1

Allele1: GTTAAATAACTTATCAAGAACACCTTAGGATTTGTGCTGGCTGCAATATGGAGATTGGGCATGGAAGA (WT)

Ref: GTTAAATAACTTATCAAGAACACCTTAGGATTTGTGCTGGCTGCAATATGGAGATTGGGCATGGAAGA

L3 *BnaC5.DA1*-sgRNA2

Allele1: GTCAACTTTCGTTTTTTTTTTCTTTGTAGCCACGG (WT)

Allele2: GTCAACTTTCGTTTTTTTTTTCCTTTGAACCCCCGA (complicated variant)

Ref: GTTTTTTTTTTCTTTGTAGCCACGGAATACAGGATATGTTGAACTTAACGATGGACGGAAACTTTGCCTGGAGTGTCTGGACT

L4 *BnaC5.DA1*-sgRNA1

Allele1: GTTAAATAACTTATCAAGAACACCTTAGGATTTGTGCTGGCTGCAATATGGAGATTGGGCATGGAAGA (WT)

Ref: GTTAAATAACTTATCAAGAACACCTTAGGATTTGTGCTGGCTGCAATATGGAGATTGGGCATGGAAGA

L4 *BnaC5.DA1*-sgRNA2

Allele1: GTCAACTTTCGTTTTTTTTTTCTTTGTAGCCACGG (WT)

Allele2: GTCAACTTTCGTTTTTTTTTCCTTTGAACCCCCGA (complicated variant)

Ref: GTTTTTTTTTTCTTTGTAGCCACGGAATACAGGATATGTTGAACTTAACGATGGACGGAAACTTTGCCTGGAGTGTCTGGACT

L5 *BnaC5.DA1*-sgRNA1

Allele1: GTTAAATAACTTATCAAGAACACCTTAGGATTTGTGCTGGCTGCAATATGGAGATTGGGCATGGAAGA (WT)

Ref: GTTAAATAACTTATCAAGAACACCTTAGGATTTGTGCTGGCTGCAATATGGAGATTGGGCATGGAAGA

L5 *BnaC5.DA1*-sgRNA2

Allele1: GTCAACTTTCGTTTTTTTTTTCTTTGTAGCCACGG (WT)

Allele2: GTCAACTTTCGTTTTTTTTTCCTTTGAACCCCCGA (complicated variant)

Ref: GTTTTTTTTTTCTTTGTAGCCACGGAATACAGGATATGTTGAACTTAACGATGGACGGAAACTTTGCCTGGAGTGTCTGGACT

L6 *BnaC5.DA1*-sgRNA1

Allele1: TTATCAAGAACACCTTA (159-bp deletion) ACCTT (deletion)

Allele2: TTATCAAGAACACCTTAGCGGA (complicated variant)

Ref: TTATCAAGAACACCTTAGGATTTGTGCTGGCTGCAATATGGAGATTGGGCATGGAAGATATCTGAAT

L6 *BnaC5.DA1*-sgRNA2

Allele1: GTCAACTTTCGTTTTTTTTTTCTTTGTAGCCACGG (WT)

Allele2: GTCAACTTTCGTTTTTTTTTCCTTTGAACCCCCGA (complicated variant)

Ref: GTTTTTTTTTTCTTTGTAGCCACGGAATACAGGATATGTTGAACTTAACGATGGACGGAAACTTTGCCTGGAGTGTCTGGACT

L7 *BnaC5.DA1*-sgRNA1

Allele1: GTTAAATAACTTATCAAGAACACCTTAGGATTTGTGCTGGCTGCAATATGGAGATTGGGCATGGAAGA (WT)

Ref: GTTAAATAACTTATCAAGAACACCTTAGGATTTGTGCTGGCTGCAATATGGAGATTGGGCATGGAAGA

L7 *BnaC5.DA1*-sgRNA2

Allele1:	GTCAACTTTCGTTTTTTTTTTCTTTGTAGCCACGG	(WT)
Allele2:	GTCAACTTTCGTTTTTTTTTCCTTTGAACCCCCGA	(complicated variant)
Ref:	GTTTTTTTTTTCTTTGTAGCCACGGAATACAGGATATGTTGAACTTAACGATGGACGGAAACTTTGCCTGGAGTGTCTGGACT	

L8 *BnaC5.DA1*-sgRNA1

Allele1:	GTTAAATAACTTATCAAGAACACCTTAGGATTTGTGCTGGCTGCAATATGGAGATTGGGCATGGAAGA	(WT)
Ref:	GTTAAATAACTTATCAAGAACACCTTAGGATTTGTGCTGGCTGCAATATGGAGATTGGGCATGGAAGA	

L8 *BnaC5.DA1*-sgRNA2

Allele1:	GTCAACTTTCGTTTTTTTTTTCTTTGTAGCCACGG	(WT)
Allele2:	GTCAACTTTCGTTTTTTTTTCCTTTGAACCCCCGA	(complicated variant)
Ref:	GTTTTTTTTTTCTTTGTAGCCACGGAATACAGGATATGTTGAACTTAACGATGGACGGAAACTTTGCCTGGAGTGTCTGGACT	

L9 *BnaC5.DA1*-sgRNA1

Allele1:	GTTAAATAACTTATCAAGAACACCTTAGGATTTGTGCTGGCTGCAATATGGAGATTGGGCATGGAAGA	(WT)
Ref:	GTTAAATAACTTATCAAGAACACCTTAGGATTTGTGCTGGCTGCAATATGGAGATTGGGCATGGAAGA	

L9 *BnaC5.DA1*-sgRNA2

Allele1:	GTCAACTTTCGTTTTTTTTTTCTTTGTAGCCACGG	(WT)
Allele2:	GTCAACTTTCGTTTTTTTTTCCTTTGAACCCCCGA	(complicated variant)
Ref:	GTTTTTTTTTTCTTTGTAGCCACGGAATACAGGATATGTTGAACTTAACGATGGACGGAAACTTTGCCTGGAGTGTCTGGACT	

L10 *BnaC5.DA1*-sgRNA1

Allele1:	CAAGAACACCTTAGGATTTG-TGCTGGCTGCAATATGGAGATTGGGCATGGAAGATATCTGAATTGCTTGA	(WT)
Allele2:	CAAGAACACCTTAGGATTTGATGCTGGCTGCAATATGGAGATTGGGCATGGAAGATATCTGAATTGCTTGA	(insertion)
Ref:	CAAGAACACCTTAGGATTTG-TGCTGGCTGCAATATGGAGATTGGGCATGGAAGATATCTGAATTGCTTGA	

L10 *BnaC5.DA1*-sgRNA2

Allele1: GTCAACTTTCGTTTTTTTTTTCTTTGTAGCCACGG (WT)

Allele2: GTCAACTTTCGTTTTTTTTTCCTTTGAACCCCCGA (complicated variant)

Ref: GTTTTTTTTTTCTTTGTAGCCACGGAATACAGGATATGTTGAACTTAACGATGGACGGAAACTTTGCCTGGAGTGTCTGGACT

L11 *BnaC5.DA1*-sgRNA1

Allele1: GTTAAATAACTTATCAAGAACACCTTAGGATTTGTGCTGGCTGCAATATGGAGATTGGGCATGGAAGA (WT)

Ref: GTTAAATAACTTATCAAGAACACCTTAGGATTTGTGCTGGCTGCAATATGGAGATTGGGCATGGAAGA

L11 *BnaC5.DA1*-sgRNA2

Allele1: GTCAACTTTCGTTTTTTTTTTCTTTGTAGCCACGG (WT)

Allele2: GTCAACTTTCGTTTTTTTTTCCTTTGAACCCCCGA (complicated variant)

Ref: GTTTTTTTTTTCTTTGTAGCCACGGAATACAGGATATGTTGAACTTAACGATGGACGGAAACTTTGCCTGGAGTGTCTGGACT

L12 *BnaC5.DA1*-sgRNA1

Allele1: TTATCAAGAACACCTTA (159-bp deletion) ACCTT (deletion)

Allele2: TTATCAAGAACACCTTAGCGGA (complicated variant)

Ref: TTATCAAGAACACCTTAGGATTTGTGCTGGCTGCAATATGGAGATTGGGCATGGAAGATATCTGAAT

L12 *BnaC5.DA1*-sgRNA2

Allele1: GTCAACTTTCGTTTTTTTTTTCTTTGTAGCCACGG (WT)

Allele2: GTCAACTTTCGTTTTTTTTTCCTTTGAACCCCCGA (complicated variant)

Ref: GTTTTTTTTTTCTTTGTAGCCACGGAATACAGGATATGTTGAACTTAACGATGGACGGAAACTTTGCCTGGAGTGTCTGGACT

L13 *BnaC5.DA1*-sgRNA1

Allele1: CAAGAACACCTTAGGATTTG-TGCTGGCTGCAATATGGAGATTGGGCATGGAAGATATCTGAATTGCTTGA (WT)

Allele2: CAAGAACACCTTAGGATTTGATGCTGGCTGCAATATGGAGATTGGGCATGGAAGATATCTGAATTGCTTGA (insertion)

Ref: CAAGAACACCTTAGGATTTG-TGCTGGCTGCAATATGGAGATTGGGCATGGAAGATATCTGAATTGCTTGA

L13 *BnaC5.DA1*-sgRNA2

Allele1: GTCAACTTTCGTTTTTTTTTTCTTTGTAGCCACGG (WT)

Allele2: GTCAACTTTCGTTTTTTTTTCCTTTGAACCCCCGA (complicated variant)

Ref: GTTTTTTTTTTCTTTGTAGCCACGGAATACAGGATATGTTGAACTTAACGATGGACGGAAACTTTGCCTGGAGTGTCTGGACT

L14 *BnaC5.DA1*-sgRNA1

Allele1: AGAACACCTTAGGATTTGTGCTGGCTGCAATATGG (WT)

Allele2: AGAACACCTTAGGATTTGTGGTGGGTGCCATAAGG (complicated variant)

Ref: AGAACACCTTAGGATTTGTGCTGGCTGCAATATGGAGATTGGGCATGGAAGATATCTGAATTGCTTGAAT

L14 *BnaC5.DA1*-sgRNA2

Allele1: GTCAACTTTCGTTTTTTTTTTCTTTGTAGCCACGG (WT)

Allele2: GTCAACTTTCGTTTTTTTTTCCTTTGAACCCCCGA (complicated variant)

Ref: GTTTTTTTTTTCTTTGTAGCCACGGAATACAGGATATGTTGAACTTAACGATGGACGGAAACTTTGCCTGGAGTGTCTGGACT

L15 *BnaC5.DA1*-sgRNA1

Allele1: GTTAAATAACTTATCAAGAACACCTTAGGATTTGTGCTGGCTGCAATATGGAGATTGGGCATGGAAGA (WT)

Ref: GTTAAATAACTTATCAAGAACACCTTAGGATTTGTGCTGGCTGCAATATGGAGATTGGGCATGGAAGA

L15 *BnaC5.DA1*-sgRNA2

Allele1: GTCAACTTTCGTTTTTTTTTTCTTTGTAGCCACGG (WT)

Allele2: GTCAACTTTCGTTTTTTTTTCCTTTGAACCCCCGA (complicated variant)

Ref: GTTTTTTTTTTCTTTGTAGCCACGGAATACAGGATATGTTGAACTTAACGATGGACGGAAACTTTGCCTGGAGTGTCTGGACT

L16 *BnaC5.DA1*-sgRNA1

Allele1: CAAGAACACCTTAGGATTTGATGCTGGCTGCAATATGGAGATTGGGCATGGAAGATATCTGAATTGCTTGA (insertion)

Allele2: CAAGAACACCTTAGGATTTGTTGCTGGCTGCAATATGGAGATTGGGCATGGAAGATATCTGAATTGCTTGA (insertion)

Ref: CAAGAACACCTTAGGATTTG-TGCTGGCTGCAATATGGAGATTGGGCATGGAAGATATCTGAATTGCTTGA

L16 *BnaC5.DA1*-sgRNA2

Allele1:	GTCAACTTTCGTTTTTTTTTTCTTTGTAGCCACGG	(WT)
Allele2:	GTCAACTTTCGTTTTTTTTTCCTTTGAACCCCCGA	(complicated variant)
Ref:	GTTTTTTTTTTCTTTGTAGCCACGGAATACAGGATATGTTGAACTTAACGATGGACGGAAACTTTGCCTGGAGTGTCTGGACT	

L17 *BnaC5.DA1*-sgRNA1

Allele1:	CAAGAACACCTTAGGATTTGCTGCTGGCTGCAATATGGAGATTGGGCATGGAAGATATCTGAATTGCTTGA	(insertion)
Allele2:	CAAGAACACCTTAGGATTTGTTGCTGGCTGCAATATGGAGATTGGGCATGGAAGATATCTGAATTGCTTGA	(insertion)
Ref:	CAAGAACACCTTAGGATTTG-TGCTGGCTGCAATATGGAGATTGGGCATGGAAGATATCTGAATTGCTTGA	

L17 *BnaC5.DA1*-sgRNA2

Allele1:	GTCAACTTTCGTTTTTTTTTTCTTTGTAGCCACGG	(WT)
Allele2:	GTCAACTTTCGTTTTTTTTTCCTTTGAACCCCCGA	(complicated variant)
Ref:	GTTTTTTTTTTCTTTGTAGCCACGGAATACAGGATATGTTGAACTTAACGATGGACGGAAACTTTGCCTGGAGTGTCTGGACT	

L18 *BnaC5.DA1*-sgRNA1

Allele1:	AAGAACACCTTAGGATTTG-TGCTGGCTGCAATATGGAGATTGGGCATGGAAGATATCTGAATTGCTTGAA	(WT)
Allele2:	AAGAACACCTTAGGATTTGTTGCTGGCTGCAATATGGAGATTGGGCATGGAAGATATCTGAATTGCTTGAA	(insertion)
Ref:	AAGAACACCTTAGGATTTG-TGCTGGCTGCAATATGGAGATTGGGCATGGAAGATATCTGAATTGCTTGAA	

L18 *BnaC5.DA1*-sgRNA2

Allele1:	GTCAACTTTCGTTTTTTTTTTCTTTGTAGCCACGG	(WT)
Allele2:	GTCAACTTTCGTTTTTTTTTCCTTTGAACCCCCGA	(complicated variant)
Ref:	GTTTTTTTTTTCTTTGTAGCCACGGAATACAGGATATGTTGAACTTAACGATGGACGGAAACTTTGCCTGGAGTGTCTGGACT	

L19 *BnaC5.DA1*-sgRNA1

Allele1:	AGAACACCTTAGGATTTGTGCTGGCTGCAATATGGAGATTGGGCATGGAAGATATCTGAATTGCTTGAAT	(WT)
Allele2:	AGAACACCTTAGGATTTGTGGTGGGTGCCATAAGG	(complicated variant)
Ref:	AGAACACCTTAGGATTTGTGCTGGCTGCAATATGGAGATTGGGCATGGAAGATATCTGAATTGCTTGAAT	

L19 *BnaC5.DA1*-sgRNA2

Allele1: GTCAACTTTCGTTTTTTTTTCTTTGTAGCCACGG (WT)

Allele2: GTCAACTTTCGTTTTTTTTTCCTTTGAACCCCCGA (complicated variant)

Ref: GTTTTTTTTTTCTTTGTAGCCACGGAATACAGGATATGTTGAACTTAACGATGGACGGAAACTTTGCCTGGAGTGTCTGGACT

L20 *BnaC5.DA1*-sgRNA1

Allele1: GTTAAATAACTTATCAAGAACACCTTAGGATTTGTGCTGGCTGCAATATGGAGATTGGGCATGGAAGA (WT)

Ref: GTTAAATAACTTATCAAGAACACCTTAGGATTTGTGCTGGCTGCAATATGGAGATTGGGCATGGAAGA

L20 *BnaC5.DA1*-sgRNA2

Allele1: GTCAACTTTCGTTTTTTTTTCTTTGTAGCCACGG (WT)

Allele2: GTCAACTTTCGTTTTTTTTTCCTTTGAACCCCCGA (complicated variant)

Ref: GTTTTTTTTTTCTTTGTAGCCACGGAATACAGGATATGTTGAACTTAACGATGGACGGAAACTTTGCCTGGAGTGTCTGGACT

BnaA2.DA2

L1 *BnaDA2*-sgRNA2

Allele1: ATAGATATACTTCACCTGTA-CAAATGCTTTTCATGCAACATCTTGACCTATTGAGGCTAGGATAGTACTA (WT)

Allele2: ATAGATATACTTCACCTGTAACAAATGCTTTTCATGCAACATCTTGACCTATTGAGGCTAGGATAGTACTA (insertion)

Ref: ATAGATATACTTCACCTGTA-CAAATGCTTTTCATGCAACATCTTGACCTATTGAGGCTAGGATAGTACTA

L2 *BnaDA2*-sgRNA2

Allele1: TAGATATACTTCACCTGTACAAATGCTTTTCATGCAACATCTTGACCTATTGAGGCTAGGATAGTACTAG (WT)

Allele2: TAGATATACTTCACCTGTACCAAAGGCTTTTCAGG (complicated variant)

Ref: TAGATATACTTCACCTGTACAAATGCTTTTCATGCAACATCTTGACCTATTGAGGCTAGGATAGTACTAG

L3 *BnaDA2*-sgRNA2

Allele1: ATAGATATACTTCACCTGTA-CAAATGCTTTTCATGCAACATCTTGACCTATTGAGGCTAGGATAGTACTA (WT)

Allele2: ATAGATATACTTCACCTGTAACAAATGCTTTTCATGCAACATCTTGACCTATTGAGGCTAGGATAGTACTA (insertion)

Ref: ATAGATATACTTCACCTGTA-CAAATGCTTTTCATGCAACATCTTGACCTATTGAGGCTAGGATAGTACTA

L5 *BnaDA2*-sgRNA2

Allele1: ATCCTAGCCTCAATAGGTCAAGATGTTGCATGAAAAGCATTTGTACAGGTGAAGTATATCTATCTCATCAGCTTTT (WT)

Ref: ATCCTAGCCTCAATAGGTCAAGATGTTGCATGAAAAGCATTTGTACAGGTGAAGTATATCTATCTCATCAGCTTTT

L7 *BnaDA2*-sgRNA2

Allele1: TATCCTAGCCTCAATAGGTCAAGATGTTGCATGAAAAGCATTTGTACAGGTGAAGTATATCTATCTCATCAGCTTTT (WT)

Ref: TATCCTAGCCTCAATAGGTCAAGATGTTGCATGAAAAGCATTTGTACAGGTGAAGTATATCTATCTCATCAGCTTTT

L9 *BnaDA2*-sgRNA2

Allele1: TATCCTAGCCTCAATAGGTCAAGATGTTGCATGAAAAGCATTTGTACAGGTGAAGTATATCTATCTCATCAGCTTTT (WT)

Ref: TATCCTAGCCTCAATAGGTCAAGATGTTGCATGAAAAGCATTTGTACAGGTGAAGTATATCTATCTCATCAGCTTTT

L10 *BnaDA2*-sgRNA2

Allele1: TATCCTAGCCTCAATAGGTCAAGATGTTGCATGAAAAGCATTTGTACAGGTGAAGTATATCTATCTCATCAGCTTTT (WT)

Ref: TATCCTAGCCTCAATAGGTCAAGATGTTGCATGAAAAGCATTTGTACAGGTGAAGTATATCTATCTCATCAGCTTTT

L11 *BnaDA2*-sgRNA2

Allele1: TATCCTAGCCTCAATAGGTCAAGATGTTGCATGAAAAGCATTTGTACAGGTGAAGTATATCTATCTCATCAGCTTTT (WT)

Ref: TATCCTAGCCTCAATAGGTCAAGATGTTGCATGAAAAGCATTTGTACAGGTGAAGTATATCTATCTCATCAGCTTTT

L12 *BnaDA2*-sgRNA2

Allele1: ATAGATATACTTCACCTGTA-----TGCTTTTCATGCAACATCTTGACCTATTGAGGCTAGGATAGTACTA (deletion)

Allele2: ATAGATATACTTCACCTGTAACAAATGCTTTTCATGCAACATCTTGACCTATTGAGGCTAGGATAGTACTA (insertion)

Ref: ATAGATATACTTCACCTGTA-CAAATGCTTTTCATGCAACATCTTGACCTATTGAGGCTAGGATAGTACTA

L13 *BnaDA2*-sgRNA2

Allele1: ATAGATATACTTCACCTGTA-CAAATGCTTTTCATGCAACATCTTGACCTATTGAGGCTAGGATAGTACTA (WT)

Allele2: ATAGATATACTTCACCTGTAGCAAATGCTTTTCATGCAACATCTTGACCTATTGAGGCTAGGATAGTACTA (insertion)

Ref: ATAGATATACTTCACCTGTA-CAAATGCTTTTCATGCAACATCTTGACCTATTGAGGCTAGGATAGTACTA

L14 *BnaDA2*-sgRNA2

Allele1:	TATCCTAGCCTCAATAGGTCAAGATGTTGCATGAAAAGCATTTGTACAGGTGAAGTATATCTATCTCATCAGCTTTT	(WT)
Ref:	TATCCTAGCCTCAATAGGTCAAGATGTTGCATGAAAAGCATTTGTACAGGTGAAGTATATCTATCTCATCAGCTTTT	

L16 *BnaDA2*-sgRNA2

Allele1:	TATCCTAGCCTCAATAGGTCAAGATGTTGCATGAAAAGCATTTGTACAGGTGAAGTATATCTATCTCATCAGCTTTT	(WT)
Ref:	TATCCTAGCCTCAATAGGTCAAGATGTTGCATGAAAAGCATTTGTACAGGTGAAGTATATCTATCTCATCAGCTTTT	

L17 *BnaDA2*-sgRNA2

Allele2:	ATAGATATACTTCACCTGTAACAAATGCTTTTCATGCAACATCTTGACCTATTGAGGCTAGGATAGTACTA	(insertion)
Ref:	ATAGATATACTTCACCTGTA-CAAATGCTTTTCATGCAACATCTTGACCTATTGAGGCTAGGATAGTACTA	

L18 *BnaDA2*-sgRNA2

Allele1:	TATCCTAGCCTCAATAGGTCAAGATGTTGCATGAAAAGCATTTGTACAGGTGAAGTATATCTATCTCATCAGCTTTT	(WT)
Ref:	TATCCTAGCCTCAATAGGTCAAGATGTTGCATGAAAAGCATTTGTACAGGTGAAGTATATCTATCTCATCAGCTTTT	

L19 *BnaDA2*-sgRNA2

Allele1:	ATAGATATACTTCACCTGTA-CAAATGCTTTTCATGCAACATCTTGACCTATTGAGGCTAGGATAGTACTA	(WT)
Allele2:	ATAGATATACTTCACCTGTATCAAATGCTTTTCATGCAACATCTTGACCTATTGAGGCTAGGATAGTACTA	(insertion)
Ref:	ATAGATATACTTCACCTGTA-CAAATGCTTTTCATGCAACATCTTGACCTATTGAGGCTAGGATAGTACTA	

L20 *BnaDA2*-sgRNA2

Allele1:	ATAGATATACTTCACCTGTA-CAAATGCTTTTCATGCAACATCTTGACCTATTGAGGCTAGGATAGTACTA	(WT)
Allele2:	ATAGATATACTTCACCTGTAACAAATGCTTTTCATGCAACATCTTGACCTATTGAGGCTAGGATAGTACTA	(insertion)
Ref:	ATAGATATACTTCACCTGTA-CAAATGCTTTTCATGCAACATCTTGACCTATTGAGGCTAGGATAGTACTA	

L22 *BnaDA2*-sgRNA2

Allele1:	TAGATATACTTCACCTGTACAAATGCTTTTCATGCAACATCTTGACCTATTGAGGCTAGGATAGTACTAG	(WT)
Allele2:	TAGATATACTTCACCTGTACCAAAGGCTTTTCAGG	(complicated variant)
Ref:	TAGATATACTTCACCTGTACAAATGCTTTTCATGCAACATCTTGACCTATTGAGGCTAGGATAGTACTAG	

BnaC6.DA2

L1 *BnaDA2*-sgRNA1

Allele1: TCGTAGTAACTGTCGCAGTT-GCCAGGAAGCATACT (WT)

Allele2: TCGTAGTAACTGTCGCAGTTTGCCAGGAAGCATAC (insertion)

Ref: TCGTAGTAACTGTCGCAGTT-GCCAGGAAGCATACTGTATGAAGCAACGTTGACGTTGTGATTGTGATTAG

L1 *BnaDA2*-sgRNA2

Allele1: AGCCTCAATAGATCAAGATGTTGCATGAAAAGCATTTGTTACAGGTGAAAAATTCATCAGATTTTT (insertion)

Ref: AGCCTCAATAGATCAAGATGTTGCATGAAAAGCATTTG-TACAGGTGAAAAATTCATCAGATTTTT

L2 *BnaDA2*-sgRNA1

Allele1: CGTCTTGTTCTATGTCGTAGTAACTGTCGCAGTTGCCAGGAAG (WT)

Ref: CGTCTTGTTCTATGTCGTAGTAACTGTCGCAGTTGCCAGGAAG

L2 *BnaDA2*-sgRNA2

Allele1: TTCACCTGTA-CAAATGCTTTTCATGCAACATCTTGATCTATTGAGGCTAGGATAGAACTA (WT)

Allele2: TTCACCTGTAACAAAGGCTTTTCAGGCAAC (complicated variant)

Ref: TTCACCTGTA-CAAATGCTTTTCATGCAACATCTTGATCTATTGAGGCTAGGATAGAACTA

L3 *BnaDA2*-sgRNA1

Allele1: CGTCTTGTTCTATGTCGTAGTAACTGTCGCAGTTGCCAGGAAG (WT)

Ref: CGTCTTGTTCTATGTCGTAGTAACTGTCGCAGTTGCCAGGAAG

L3 *BnaDA2*-sgRNA2

Allele1: AAAAAAAAAATCTGATGAA (270-bp deletion) GCCTT (deletion)

Allele2: AAAAAAAAAATCTGATGAAACGGA (complicated variant)

Ref: AAAAAAAAAATCTGATGAATTTTTCACCTGTACAAATGCTTTTCATGCAACATCTTGATCTATTGAGGC

L5 *BnaDA2*-sgRNA1

Allele1: CGTCTTGTTCTATGTCGTAGTAACTGTCGCAGTTGCCAGGAAG (WT)

Ref: CGTCTTGTTCTATGTCGTAGTAACTGTCGCAGTTGCCAGGAAG

L5 *BnaDA2*-sgRNA2

Allele1:	GATGAATTTTTCACCTGTACAAATGCTTTTCATGCAACATCTTGATCTATTGAG	(WT)
Allele2:	GATGAATTTTTCACCTGTACCAAAGGTTTTTCTGG	(complicated variant)
Ref:	GATGAATTTTTCACCTGTACAAATGCTTTTCATGCAACATCTTGATCTATTGAGGCTAGGATAGAACTAA	

L7 *BnaDA2*-sgRNA1

Allele1:	CGTCTTGTTCTATGTCGTAGTAACTGTCGCAGTTGCCAGGAAG	(WT)
Ref:	CGTCTTGTTCTATGTCGTAGTAACTGTCGCAGTTGCCAGGAAG	

L7 *BnaDA2*-sgRNA2

Allele1:	GATGAATTTTTCACCTGTACAAATGCTTTTCATGCAACATCTTGATCTATTGAG	(WT)
Allele2:	GATGAATTTTTCACCTGTACCAAAGGTTTTTCTGG	(complicated variant)
Ref:	GATGAATTTTTCACCTGTACAAATGCTTTTCATGCAACATCTTGATCTATTGAGGCTAGGATAGAACTAA	

L9 *BnaDA2*-sgRNA1

Allele1:	CGTCTTGTTCTATGTCGTAGTAACTGTCGCAGTTGCCAGGAAG	(WT)
Ref:	CGTCTTGTTCTATGTCGTAGTAACTGTCGCAGTTGCCAGGAAG	

L9 *BnaDA2*-sgRNA2

Allele1:	AAAAAAAAAATCTGATGAA (270-bp deletion) GCCTT	(deletion)
Allele2:	AAAAAAAAAATCTGATGAAACGGA	(complicated variant)
Ref:	AAAAAAAAAATCTGATGAATTTTTCACCTGTACAAATGCTTTTCATGCAACATCTTGATCTATTGAGGC	

L10 *BnaDA2*-sgRNA1

Allele1:	CGTCTTGTTCTATGTCGTAGTAACTGTCGCAGTTGCCAGGAAG	(WT)
Ref:	CGTCTTGTTCTATGTCGTAGTAACTGTCGCAGTTGCCAGGAAG	

L10 *BnaDA2*-sgRNA2

Allele1:	CGTCTTGTTCTATGTCGTAGTAACTGTCGCAGTTGCCAGGAAG	(WT)
Ref:	CGTCTTGTTCTATGTCGTAGTAACTGTCGCAGTTGCCAGGAAG	

L11 *BnaDA2*-sgRNA1

Allele1:	CGTCTTGTTCTATGTCGTAGTAACTGTCGCAGTTGCCAGGAAG	(WT)
Ref:	CGTCTTGTTCTATGTCGTAGTAACTGTCGCAGTTGCCAGGAAG	

L11 *BnaDA2*-sgRNA2

Allele1:	ATTTTTCACCTGTACAAATGCTTTTCATGCAACAT	(WT)
Allele2:	ATTTTTCACCTGTACAAATGGTTTTCATGGAACAT	(substitution)
Ref:	ATTTTTCACCTGTACAAATGCTTTTCATGCAACATCTTGATCTATTGAGGCTAGGATAGAACTAAAAAAA	

L12 *BnaDA2*-sgRNA1

Allele1:	CATACAGTATGCTTCCTGGC----GCGACAGTTACTACGACATAGAACAAGACGCAGA	(deletion)
Allele2:	CATACAGTATGCTTCCTGGC	(complicated variant)
Ref:	CATACAGTATGCTTCCTGGCAACTGCGACAGTTACTACGACATAGAACAAGACGCAGATGACATTGACCA	

L12 *BnaDA2*-sgRNA2

Allele1:	TGATGAATTTTTCACCTGTAACAAATGCTTTTCATGCAACATCTTGATCTATTGAGGCTAGGATAGAACTA	(insertion)
Allele2:	TGATGAATTTTTCACCTGTATCAAATGCTTTTCATGCAACATCTTGATCTATTGAGGCTAGGATAGAACTAA	(insertion)
Ref:	TGATGAATTTTTCACCTGTA-CAAATGCTTTTCATGCAACATCTTGATCTATTGAGGCTAGGATAGAACTAAAAAAA	

L13 *BnaDA2*-sgRNA1

Allele1:	CGTCTTGTTCTATGTCGTAGTAACTGTCGCAGTTGCCAGGAAG	(WT)
Ref:	CGTCTTGTTCTATGTCGTAGTAACTGTCGCAGTTGCCAGGAAG	

L13 *BnaDA2*-sgRNA2

Allele1:	TGATGAATTTTTCACCTGTA-CAAATGCTTTTCATGCAACATCTTGATCTATTGAGGCTAGGATAGAACTA	(WT)
Allele2:	TGATGAATTTTTCACCTGTAACAAATGCTTTTCATGCAACATCTTGATCTATTGAGGCTAGGATAGAACTA	(insertion)
Ref:	TGATGAATTTTTCACCTGTA-CAAATGCTTTTCATGCAACATCTTGATCTATTGAGGCTAGGATAGAACTA	

L14 *BnaDA2*-sgRNA1

Allele1:	CGTCTTGTTCTATGTCGTAGTAACTGTCGCAGTTGCCAGGAAG	(WT)
Ref:	CGTCTTGTTCTATGTCGTAGTAACTGTCGCAGTTGCCAGGAAG	

L14 *BnaDA2*-sgRNA2

Allele1: TGATGAATTTTTCACCTGTA-CAAATGCTTTTCATGCAACATCTTGATCTATTGAGGCTAGGATAGAACTA (WT)

Allele2: TGATGAATTTTTCACCTGTAACAAATGCTTTTCATGCAACATCTTGATCTATTGAGGCTAGGATAGAACTA (insertion)

Ref: TGATGAATTTTTCACCTGTA-CAAATGCTTTTCATGCAACATCTTGATCTATTGAGGCTAGGATAGAACTA

L17 *BnaDA2*-sgRNA1

Allele1: CGTCTTGTTCTATGTCGTAGTAACTGTCGCAGTTGCCAGGAAG (WT)

Ref: CGTCTTGTTCTATGTCGTAGTAACTGTCGCAGTTGCCAGGAAG

L17 *BnaDA2*-sgRNA2

Allele1: CGTCTTGTTCTATGTCGTAGTAACTGTCGCAGTTGCCAGGAAG (WT)

Ref: CGTCTTGTTCTATGTCGTAGTAACTGTCGCAGTTGCCAGGAAG

L18 *BnaDA2*-sgRNA1

Allele1: CGTCTTGTTCTATGTCGTAGTAACTGTCGCAGTTGCCAGGAAG (WT)

Ref: CGTCTTGTTCTATGTCGTAGTAACTGTCGCAGTTGCCAGGAAG

L18 *BnaDA2*-sgRNA2

Allele1: AAAAAAAAAATCTGATGAA (270-bp deletion) GCCTT (deletion)

Allele2: AAAAAAAAAATCTGATGAAACGGA (complicated variant)

Ref: AAAAAAAAAATCTGATGAATTTTTCACCTGTACAAATGCTTTTCATGCAACATCTTGATCTATTGAGGC

L19 *BnaDA2*-sgRNA1

Allele1: TTCTATGTCGTAGTAACTGTCGCAGTTGCCAGGAAGC (WT)

Allele2: TTCTATGTCGTAGTAACTGTCGCAGTTTGCCAGGAAC (complicated variant)

Ref: TTCTATGTCGTAGTAACTGTCGCAGTTGCCAGGAAGCATACTGTATGAAGCAACGTTGACGTTGTGATTGTGATTAG

L19 *BnaDA2*-sgRNA2

Allele1: AAAAAAAAAATCTGATGAA (270-bp deletion) GCCTT (deletion)

Allele2: AAAAAAAAAATCTGATGAAACGGA (complicated variant)

Ref: AAAAAAAAAATCTGATGAATTTTTCACCTGTACAAATGCTTTTCATGCAACATCTTGATCTATTGAGGC

L20 *BnaDA2*-sgRNA1

Allele1: TCGTAGTAACTGTCGCAGTTGCCAGGAAGCATACT (WT)

Allele2: TCGTAGTAACTGTCGCAGTT (complicated variant)

Ref: TCGTAGTAACTGTCGCAGTTGCCAGGAAGCATACTGTATGAAGCAACGTTGACGTTGTGATTGTGATTAG

L20 *BnaDA2*-sgRNA2

Allele1: TGATGAATTTTTCACCTGTAACAAATGCTTTTCATGCAACATCTTGATCTATTGAGGC (insertion)

Allele2: TGATGAATTTTTCACCTGTACCAAATGCTTTTCATGCAACATCTTGATCTATTGAGGC (insertion)

Ref: TGATGAATTTTTCACCTGTA-CAAATGCTTTTCATGCAACATCTTGATCTATTGAGGC

L22 *BnaDA2*-sgRNA1

Allele1: TCGTAGTAACTGTCGCAGTT-GCCAGGAAGCATACT (WT)

Allele2: TCGTAGTAACTGTCGCAGTTTGCCAGGAAGCATAC (insertion)

Ref: TCGTAGTAACTGTCGCAGTT-GCCAGGAAGCATACTGTATGAAGCAACGTTGACGTTGTGATTGTGATTAG

L22 *BnaDA2*-sgRNA2

Allele1: AAAAAAAAAATCTGATGAA (270-bp deletion) GCCTT (deletion)

Allele2: AAAAAAAAAATCTGATGAAACGGA (complicated variant)

Ref: AAAAAAAAAATCTGATGAATTTTTCACCTGTACAAATGCTTTTCATGCAACATCTTGATCTATTGAGGC

BnaC2.FUL

L3 *BnaFUL*-sgRNA1

Allele1: TTGCTCTCATCGTCTTCTCTT (complicated variant)

Ref: TTGCTCTCATCGTCTTCTCTTCCAAAGGCAAACTCTTCGAATATTCCACAGACTCTTGGTAATTAT

L3 *BnaFUL*-sgRNA2

Allele1: AGAGATGGGAAGGGGTAGGG-----------AGGATAGAGAATAAGATAAATAGGCAAGTTACTTTCTCA (deletion)

Allele2: AGAGATGGGAAGGGGTAGGGTTCAGCTG----GGATAGAGAATAAGATAAATAGGCAAGTTACTTTCTCA (deletion)

Ref: AGAGATGGGAAGGGGTAGGGTTCAGCTGAAGAGGATAGAGAATAAGATAAATAGGCAAGTTACTTTCTCA

L4 *BnaFUL*-sgRNA1

Allele1:	TTGCTCTCATCGTCTTCTCTT	(complicated variant)
Ref:	TTGCTCTCATCGTCTTCTCTTCCAAAGGCAAACTCTTCGAATATTCCACAGACTCTTGGTAATTAT	

L4 *BnaFUL*-sgRNA2

Allele1:	AGAGATGGGAAGGGGTAGGGTTCAGCT-AAGAGGATAGAGAATAAGATAAATAGGCAAG	(deletion)
Allele2:	AGAGATGGGAAGGGGTAGGGTTCAGCTGAAAG-------------GATAA	(substitution and deletion)
Ref:	AGAGATGGGAAGGGGTAGGGTTCAGCTGAAGAGGATAGAGAATAAGATAAATAGGCAAGTTACTTTCTCAAAGAGAA	

L5 *BnaFUL*-sgRNA1

Allele1:	TTGCTCTCATCGTCTTCTCTTCCAAAGGCAAACTCTTCGAATATTCCACAGACTCTTGGTAATTAT	(WT)
Ref:	TTGCTCTCATCGTCTTCTCTTCCAAAGGCAAACTCTTCGAATATTCCACAGACTCTTGGTAATTAT	

L5 *BnaFUL*-sgRNA2

Allele1:	TGGGAAGGGGTAGGGTTCAG (43-bp deletion) CAAAGAGAAGGTCTG	(deletion)
Allele2:	TGGGAAGGGGTAGGGTTCAGAAGAAAGAAA	(complicated variant)
Ref:	TGGGAAGGGGTAGGGTTCAGCTGAAGAGGATAGAGAATAAGATAAATAGGCAAGTTACTTTCTCAAAGAG	

L6 *BnaFUL*-sgRNA1

Allele1:	TTGCTCTCATCGTCTTCTCTTCCAAAGGCAAACTCTTCGAATATTCCACAGACTCTTGGTAATTAT	(WT)
Ref:	TTGCTCTCATCGTCTTCTCTTCCAAAGGCAAACTCTTCGAATATTCCACAGACTCTTGGTAATTAT	

L6 *BnaFUL*-sgRNA2

Allele1:	TGGGAAGGGGTAGGGTTCAGCTGAAAGAGGATAGAGAATAAGATAAATAGGCAAGTTACTTTCTCAAAGAG	（insertion）
Allele2:	TGGGAAGGGGTAGGGTTCAGCTG	(complicated variant)
Ref:	TGGGAAGGGGTAGGGTTCAGCTG-AAGAGGATAGAGAATAAGATAAATAGGCAAGTTACTTTCTCAAAGAG	

L7 *BnaFUL*-sgRNA1

Allele1:	TTGCTCTCATCGTCTTCTCTTCCAAAGGCAAACTCTTCGAATATTCCACAGACTCTTGGTAATTAT	(WT)
Ref:	TTGCTCTCATCGTCTTCTCTTCCAAAGGCAAACTCTTCGAATATTCCACAGACTCTTGGTAATTAT	

L7 *BnaFUL*-sgRNA2

Allele1: TGGGAAGGGGTAGGGTTCAGCTGAAAGAGGATAGAGAATAAGATAAATAGGCAAGTTACTTTCTCAAAGAG （insertion）

Allele2: TGGGAAGGGGTAGGGTTCAGCTG (complicated variant)

Ref: TGGGAAGGGGTAGGGTTCAGCTG-AAGAGGATAGAGAATAAGATAAATAGGCAAGTTACTTTCTCAAAGAG

L8 *BnaFUL*-sgRNA1

Allele1: AAGGGGTAGGGTTCAGCTGA (129bp) GCAAACTCTT (deletion)

Ref:

ATGGGAAGGGGTAGGGTTCAGCTGAAGAGGATAGAGAAtAAGATCAATAGGCAAGTTACTTTCTCAAAGAGAAGGTCTGGTTTGCTCAAGAAAGCTCATGAG
ATCTCTGTTCTCTGCGATGCTGAGGTTGCTCTCATCGTCTTCTCTTCCAAAGGCAAACTCTTCGAATATTCCACaGACTCTTG

L8 *BnaFUL*-sgRNA2

Allele1: AAGGGGTAGGGTTCAGCTGA (129bp) GCAAACTCTT

(deletion)

Ref:

AAGGGGTAGGGTTCAGCTGAAGAGGATAGAGAATAAGATAAATAGGCAAGTTACTTTCTCAAAGAGAAGGTCTGGTTTGCTCAAGAAAGCTCATGAGATCTCT
GTTCTCTGCGATGCTGAGGTTGCTCTCATCGTCTTCTCTTCCAAAGGCAAACTCTT

L9 *BnaFUL*-sgRNA1

Allele1: TTGCTCTCATCGTCTTCTCTT (complicated variant)

Ref: TTGCTCTCATCGTCTTCTCTTCCAAAGGCAAACTCTTCGAATATTCCACAGACTCTTGGTAATTAT

L9 *BnaFUL*-sgRNA2

Allele1: TGGGAAGGGGTAGGGTTCAGCTGAAGAGGATAGAGAATAAGATAAATAGGCAAGTTACTTTCTCAAAGAG (WT)

Allele2: TGGGAAGGGGTAGGGTTCAG---AAGAGGATAGAGAATAAGATAAATAGGCAAGTTACTTTCTCAAAGAG (deletion)

Ref: TGGGAAGGGGTAGGGTTCAGCTGAAGAGGATAGAGAATAAGATAAATAGGCAAGTTACTTTCTCAAAGAG

L10 *BnaFUL*-sgRNA1

Allele1: TTGCTCTCATCGTCTTCTCTT (complicated variant)

Ref: TTGCTCTCATCGTCTTCTCTTCCAAAGGCAAACTCTTCGAATATTCCACAGACTCTTGGTAATTAT

L10 *BnaFUL*-sgRNA2

Allele1:	TGGGAAGGGGTAGGGTTCAGCTGAAGAGGATAGAGAATAAGATAAATAGGCAAGTTACTTTCTCAAAGAG	(WT)
Allele2:	TGGGAAGGGGTAGGGTTCAG---AAGAGGATAGAGAATAAGATAAATAGGCAAGTTACTTTCTCAAAGAG	(deletion)
Ref:	TGGGAAGGGGTAGGGTTCAGCTGAAGAGGATAGAGAATAAGATAAATAGGCAAGTTACTTTCTCAAAGAG	

L11 *BnaFUL*-sgRNA1

Allele1:	TTGCTCTCATCGTCTTCTCTTCCAAAGGCAAACTCTTCGAATATTCCACAGACTCTTGGTAATTAT	(WT)
Ref:	TTGCTCTCATCGTCTTCTCTTCCAAAGGCAAACTCTTCGAATATTCCACAGACTCTTGGTAATTAT	

L11 *BnaFUL*-sgRNA2

Allele1:	TGGGAAGGGGTAGGGTTCAGCTGAAAGAGGATAGAGAATAAGATAAATAG	(insertion)
Ref:	TGGGAAGGGGTAGGGTTCAGCTG-AAGAGGATAGAGAATAAGATAAATAG	

L12 *BnaFUL*-sgRNA1

Allele1:	TTGCTCTCATCGTCTTCTCTT	(complicated variant)
Ref:	TTGCTCTCATCGTCTTCTCTTCCAAAGGCAAACTCTTCGAATATTCCACAGACTCTTGGTAATTAT	

L12 *BnaFUL*-sgRNA2

Allele1:	GGGAAGGGGTAGGGTTCAGCTGAAAGAGGATAGAG	(insertion)
Allele2:	GGGAAGGGGTAGGGTTCAGCTGAA (10-bp del) AAA	(complicated variant)
Ref:	GGGAAGGGGTAGGGTTCAGCTG-AAGAGGATAGAGAATAAGATAAATAGGCAAGTTACTTTCTCAAAGAGAAGGT	

L16 *BnaFUL*-sgRNA1

Allele1:	TTGCTCTCATCGTCTTCTCTT	(complicated variant)
Ref:	TTGCTCTCATCGTCTTCTCTTCCAAAGGCAAACTCTTCGAATATTCCACAGACTCTTGGTAATTAT	

L16 *BnaFUL*-sgRNA2

Allele1:	GAAGGGGTAGGGTTCAGCTGAAGAGGATAGAGAATAAGATA	(WT)
Allele2:	GAAGGGGTAGGGTTCAGCTG-----GATAGAGAAT	(deletion)
Ref:	GAAGGGGTAGGGTTCAGCTGAAGAGGATAGAGAATAAGATAAATAGGCAAGTTACTTTCTCAAAGAGAAG	

L17 *BnaFUL*-sgRNA1

Allele1:	TTGCTCTCATCGTCTTCTCTTCCAAAGGCAAACTCTTCGAATATTCCACAGACTCTTGGTAATTAT	(WT)
Ref:	TTGCTCTCATCGTCTTCTCTTCCAAAGGCAAACTCTTCGAATATTCCACAGACTCTTGGTAATTAT	

L17 *BnaFUL*-sgRNA2

Allele1:	TGGGAAGGGGTAGGGTTCAGCTGAAGAGGATAGAGAATAAGATAAATAGGCAA	(WT)
Ref:	TGGGAAGGGGTAGGGTTCAGCTGAAGAGGATAGAGAATAAGATAAATAGGCAA	

L18 *BnaFUL*-sgRNA1

Allele1:	TTGCTCTCATCGTCTTCTCTT	(complicated variant)
Ref:	TTGCTCTCATCGTCTTCTCTTCCAAAGGCAAACTCTTCGAATATTCCACAGACTCTTGGTAATTAT	

L18 *BnaFUL*-sgRNA2

Allele1:	AGGGGTAGGGTTCAGCTGAAAGAGGATAGAG	(insertion)
Allele2:	AGGGGTAGGGTTCAGCTGAA(18-bp del)	(complicated variant)
Ref:	AGGGGTAGGGTTCAGCTG-AAGAGGATAGAGAATAAGATAAATAGGCAAGTTACTTTCTCAAAGAGAAGGT	

L19 *BnaFUL*-sgRNA1

Allele1:	TTGCTCTCATCGTCTTCTCTTCCAAAGGCAAACTCTTCGAATATTCCACAGACTCTTGGTAATTAT	(WT)
Ref:	TTGCTCTCATCGTCTTCTCTTCCAAAGGCAAACTCTTCGAATATTCCACAGACTCTTGGTAATTAT	

L19 *BnaFUL*-sgRNA2

Allele1:	TGGGAAGGGGTAGGGTTCAGCTGAAGAGGATAGAGAATAAGATAAATAGGCAA	(WT)
Ref:	TGGGAAGGGGTAGGGTTCAGCTGAAGAGGATAGAGAATAAGATAAATAGGCAA	

L22 *BnaFUL*-sgRNA1

Allele1:	CATCGTCTTCTCTTCCAAAGGCAAACTCTTCGAATATTCCACAGACTCTTGGTAATTATTATCTTTTTAT	(WT)
Allele2:	CATCGTCTTCTCTTCCAAAG--AAACTCTTCGAATATTCCACAGACTCTTGGTAATTATTAT	(deletion)
Ref:	CATCGTCTTCTCTTCCAAAGGCAAACTCTTCGAATATTCCACAGACTCTTGGTAATTATTATCTTTTTAT	

L22 *BnaFUL*-sgRNA2

Allele1: TGGGAAGGGGTAGGGTTCAGCTGTAAGAGGATAGAGAATAAGATAAATAG (insertion)

Ref: TGGGAAGGGGTAGGGTTCAGCTG-AAGAGGATAGAGAATAAGATAAATAG

L24 *BnaFUL*-sgRNA1

Allele1: CATCGTCTTCTCTTCCAAAG--AAACTCTTCGAATATTCCACAGACTCTTGGTAATTATTAT (deletion)

Allele2: CATCGTCTTCTCTTCCAAAGGGCAACCCCT (complicated variant)

Ref: CATCGTCTTCTCTTCCAAAGGCAAACTCTTCGAATATTCCACAGACTCTTGGTAATTATTATCTTTTTAT

L24 *BnaFUL*-sgRNA2

Allele1: TGGGAAGGGGTAGGGTTCAGCTGTAAGAGGATAGAGAATAAGATAAATAG (insertion)

Ref: TGGGAAGGGGTAGGGTTCAGCTG-AAGAGGATAGAGAATAAGATAAATAG

BnaA9.FUL

L3 *BnaFUL*-sgRNA1

Allele1: CTCGTTGTCTTCTCTTCCA (complicated variant)

Ref: CTCGTTGTCTTCTCTTCCAAAGGCAAACTCTTCGAATATTCCACTGACTCTAG

L3 *BnaFUL*-sgRNA2

Allele1: GAAGGGGTAGGGTTCAGCTG----AGGATAGAGAACAAG (deletion)

Allele2: GAAGGGGTAGGGTTCAGCTGTAAAAGGATAGAGAA (complicated variant)

Ref: GAAGGGGTAGGGTTCAGCTG-AAGAGGATAGAGAACAAGATCAATAGGCAAGTTACTTTCTCAAAGAGAAG

L4 *BnaFUL*-sgRNA1

Allele1: CTCGTTGTCTTCTCTTCCA (complicated variant)

Ref: CTCGTTGTCTTCTCTTCCAAAGGCAAACTCTTCGAATATTCCACTGACTCTAG

L4 *BnaFUL*-sgRNA2

Allele1: AGATGGGAAGGGGTAGGGTTCAGCTG---AGGAT (deletion)

Ref: AGATGGGAAGGGGTAGGGTTCAGCTGAAGAGGATAGAGAACAAGATCAATAGGCAAGTTACTTTCTCAAA

L5 *BnaFUL*-sgRNA2

Allele1: AGATGGGAAGGGGTAGGGTT (complicated variant)

Ref: GAGAGATGGGAAGGGGTAGGGTTCAGCTGAAGAGGATAGAGAACAAGATCAATAGGCAAGTTACTTTCTC

L6 *BnaFUL*-sgRNA1

Allele1: CTCGTTGTCTTCTCTTCCA (complicated variant)

Ref: CTCGTTGTCTTCTCTTCCAAAGGCAAACTCTTCGAATATTCCACTGACTCTAG

L6 *BnaFUL*-sgRNA2

Allele1: AGATGGGAAGGGGTAGGGTT---------GAGGATAGAGAACAA (deletion)

Allele2: AGATGGGAAGGGGTAGGGTTCAGCTGTAAGAGGAT (insertion)

Ref: AGATGGGAAGGGGTAGGGTTCAGCTG-AAGAGGATAGAGAACAAGATCAATAGGCAAGTTACTTTCTCAAA

L7 *BnaFUL*-sgRNA1

Allele1: CTCGTTGTCTTCTCTTCCA (complicated variant)

Ref: CTCGTTGTCTTCTCTTCCAAAGGCAAACTCTTCGAATATTCCACTGACTCTAG

L7 *BnaFUL*-sgRNA2

Allele1: GAGAGATGGGAAGGGGTAGGGTTCAGCTGAAGAGGATAGAGAACAAGATCAATAGGCAAGTTACTTTCTC (WT)

Allele2: GAGAGATGGGAAGGGGTAGGAATAAGATACAAAAC (complicated variant)

Ref: GAGAGATGGGAAGGGGTAGGGTTCAGCTGAAGAGGATAGAGAACAAGATCAATAGGCAAGTTACTTTCTC

L8 *BnaFUL*-sgRNA1

Allele1: CGTTGTCTTCTCTTCCAAAG-CAAACTCTTCGAATATTCCACTGACTCTAG (deletion)

Ref: CGTTGTCTTCTCTTCCAAAGGCAAACTCTTCGAATATTCCACTGACTCTAG

L8 *BnaFUL*-sgRNA2

Allele1: GGGAAGGGGTAGGGTTCAGC--AAGAGGATAGAGAAC (deletion)

Ref: GGGAAGGGGTAGGGTTCAGCTGAAGAGGATAGAGAAC

L9 *BnaFUL*-sgRNA1

Allele1: CTCGTTGTCTTCTCTTCCAAAGGCAAACTCTTCGAATATTCCACTGACTCTAG (WT)

Ref: CTCGTTGTCTTCTCTTCCAAAGGCAAACTCTTCGAATATTCCACTGACTCTAG

L9 *BnaFUL*-sgRNA2

Allele1: AGATGGGAAGGGGTAGGGTT------AAGAGGATAGAGAAC (deletion)

Ref: AGATGGGAAGGGGTAGGGTTCAGCTGAAGAGGATAGAGAAC

L10 *BnaFUL*-sgRNA1

Allele1: CTCGTTGTCTTCTCTTCCAAAGGCAAACTCTTCGAATATTCCACTGACTCTAG (WT)

Ref: CTCGTTGTCTTCTCTTCCAAAGGCAAACTCTTCGAATATTCCACTGACTCTAG

L10 *BnaFUL*-sgRNA2

Allele1: AGATGGGAAGGGGTAGGGTT------AAGAGGATAGAGAAC (deletion)

Ref: AGATGGGAAGGGGTAGGGTTCAGCTGAAGAGGATAGAGAAC

L11 *BnaFUL*-sgRNA1

Allele1: CTCGTTGTCTTCTCTTCCA (complicated variant)

Ref: CTCGTTGTCTTCTCTTCCAAAGGCAAACTCTTCGAATATTCCACTGACTCTAG

L11 *BnaFUL*-sgRNA2

Allele1: AGATGGGAAGGGGTAGGGTT---------AGGATAGAGAACAAGATCAATAG (deletion)

Allele2: AGATGGGAAGGGGTAGGGTTCAGCTGAAAGAGGATAGAGAACAAGATCAATAG (insertion)

Ref: AGATGGGAAGGGGTAGGGTTCAGCTG-AAGAGGATAGAGAACAAGATCAATAGGCAAGTTACTTTCTCAAA

L12 *BnaFUL*-sgRNA1

Allele1: CTCGTTGTCTTCTCTTCCA (complicated variant)

Ref: CTCGTTGTCTTCTCTTCCAAAGGCAAACTCTTCGAATATTCCACTGACTCTAG

L12 *BnaFUL*-sgRNA2

Allele2: AGGGGTAGGGTTCAGCTGAA (complicated variant)

Ref: AGGGGTAGGGTTCAGCTGAAGAGGATAGAGAACAAGATCAATAGGCAAGTTACTTTCTCAAAGAGAAGGT

L15 *BnaFUL*-sgRNA1

Allele1:	GTTGTCTTCTCTTCCAAAG-GCAAACTCTTCGAATATTCCACTGACTCTAGGTAATTATTATCTATTGGTT	(WT)
Allele2:	GTTGTCTTCTCTTCCAAAGGGCAAACTCTTCGAATATTCCACTGACTCTAGGTAATTATTATCTATTGGTT	(insertion)
Ref:	GTTGTCTTCTCTTCCAAAG-GCAAACTCTTCGAATATTCCACTGACTCTAGGTAATTATTATCTATTGGTT	

L15 *BnaFUL*-sgRNA2

Allele1:	GGAAGGGGTAGGGTTCAGCT--AAGAGGATAGAGAAC	(deletion)
Allele2:	GGAAGGGGTAGGGTTCAGCTGTAAGAGGATAGAGA	(insertion)
Ref:	GGAAGGGGTAGGGTTCAGCTG-AAGAGGATAGAGAACAAGATCAATAGGCAAGTTACTTTCTCAAAGAGAA	

L16 *BnaFUL*-sgRNA1

Allele1:	CTCGTTGTCTTCTCTTCCA	(complicated variant)
Ref:	CTCGTTGTCTTCTCTTCCAAAGGCAAACTCTTCGAATATTCCACTGACTCTAG	

L16 *BnaFUL*-sgRNA2

Allele2:	AGGGGTAGGGTTCAGCTGAA	(complicated variant)
Ref:	AGGGGTAGGGTTCAGCTGAAGAGGATAGAGAACAAGATCAATAGGCAAGTTACTTTCTCAAAGAGAAGGT	

L17 *BnaFUL*-sgRNA1

Allele1:	CTCGTTGTCTTCTCTTCCA	(complicated variant)
Ref:	CTCGTTGTCTTCTCTTCCAAAGGCAAACTCTTCGAATATTCCACTGACTCTAG	

L17 *BnaFUL*-sgRNA2

Allele1:	AGGGGTAGGGTTCAGCTGAAGAGGATAGAG	(WT)
Allele2:	AGGGGTAGGGTTCAGCTGAA(39-bp del)AAAGA	(complicated variant)
Ref:	AGGGGTAGGGTTCAGCTGAAGAGGATAGAGAACAAGATCAATAGGCAAGTTACTTTCTCAAAGAGAAGGT	

L18 *BnaFUL*-sgRNA1

Allele1:	CTCGTTGTCTTCTCTTCCA	(complicated variant)
Ref:	CTCGTTGTCTTCTCTTCCAAAGGCAAACTCTTCGAATATTCCACTGACTCTAG	

L18 *BnaFUL*-sgRNA2

Allele2: AGGGGTAGGGTTCAGCTGAA (complicated variant)

Ref: AGGGGTAGGGTTCAGCTGAAGAGGATAGAGAACAAGATCAATAGGCAAGTTACTTTCTCAAAGAGAAGGT

L19 *BnaFUL*-sgRNA1

Allele1: CTCGTTGTCTTCTCTTCCAAAGGCAAACTCTTCGAATATTCCACTGACTCTAG (WT)

Ref: CTCGTTGTCTTCTCTTCCAAAGGCAAACTCTTCGAATATTCCACTGACTCTAG

L19 *BnaFUL*-sgRNA2

Allele1: AGATGGGAAGGGGTAGGGTTCAGCTGAAGAGGATAGAGAAC (WT)

Ref: AGATGGGAAGGGGTAGGGTTCAGCTGAAGAGGATAGAGAAC

L21 *BnaFUL*-sgRNA1

Allele1: CTCGTTGTCTTCTCTTCCAAAGGCAAACTCTTCGAATATTCCACTGACTCTAG (WT)

Ref: CTCGTTGTCTTCTCTTCCAAAGGCAAACTCTTCGAATATTCCACTGACTCTAG

L21 *BnaFUL*-sgRNA2

Allele1: AGATGGGAAGGGGTAGGGTTCAGCTGAAGAGGATAGAGAAC (WT)

Ref: AGATGGGAAGGGGTAGGGTTCAGCTGAAGAGGATAGAGAAC

L22 *BnaFUL*-sgRNA1

Allele1: CTCGTTGTCTTCTCTTCCA (complicated variant)

Ref: CTCGTTGTCTTCTCTTCCAAAGGCAAACTCTTCGAATATTCCACTGACTCTAG

L22 *BnaFUL*-sgRNA2

Allele2: AGGGGTAGGGTTCAGCTGAA (complicated variant)

Ref: AGGGGTAGGGTTCAGCTGAAGAGGATAGAGAACAAGATCAATAGGCAAGTTACTTTCTCAAAGAGAAGGT

L23 *BnaFUL*-sgRNA1

Allele1: CTCGTTGTCTTCTCTTCCA (complicated variant)

Ref: CTCGTTGTCTTCTCTTCCAAAGGCAAACTCTTCGAATATTCCACTGACTCTAG

L23 *BnaFUL*-sgRNA2

Allele1: GGAAGGGGTAGGGTTCAGCT-AAGAGGATAGAGAAC (deletion)

Allele2: GGAAGGGGTAGGGTTCAGCTG------------------------------GTTACTTTCTCAAA (deletion)

Ref: GGAAGGGGTAGGGTTCAGCTGAAGAGGATAGAGAACAAGATCAATAGGCAAGTTACTTTCTCAAAGAGAA

L24 *BnaFUL*-sgRNA1

Allele1: CTCGTTGTCTTCTCTTCCA (complicated variant)

Ref: CTCGTTGTCTTCTCTTCCAAAGGCAAACTCTTCGAATATTCCACTGACTCTAG

L24 *BnaFUL*-sgRNA2

Allele1: GGAAGGGGTAGGGTTCAGCT------------------------------AGTTACTTTCTCAAA (deletion)

Allele2: GGAAGGGGTAGGGTTCAGCTG-AGAGGATAGAGAACAAGATCAATAGGCAAGTTAC (deletion)

Ref: GGAAGGGGTAGGGTTCAGCTGAAGAGGATAGAGAACAAGATCAATAGGCAAGTTACTTTCTCAAAGAGAA

BnaC7.FUL

L3 *BnaFUL*-sgRNA1

Allele1: CATCTTCTCTTCCA (complicated variant)

Ref: CATCTTCTCTTCCAAAGGCAAACTCTTCGAATATTCCACCGACTCTTGGTAAT

L3 *BnaFUL*-sgRNA2

Allele1: GAGGAGAGAGAGAGAGAGACATGGGAAGGGGTAGGGTTCAGCTGAAGAGGATAGAAAACAAGATCAATAG (WT)

Allele2: GAGGAGAGAGAGAGAGAGACATGG-------------------GAAGAGGATAG (deletion)

Ref: GAGGAGAGAGAGAGAGAGACATGGGAAGGGGTAGGGTTCAGCTGAAGAGGATAGAAAACAAGATCAATAG

L4 *BnaFUL*-sgRNA1

Allele1: CATCTTCTCTTCCA (complicated variant)

Ref: CATCTTCTCTTCCAAAGGCAAACTCTTCGAATATTCCACCGACTCTTGGTAAT

L4 *BnaFUL*-sgRNA2

Allele1: AGAGAGAGAGACATGGGAAGGGGTAGGGTTCAGCTGAAAGAGGATAGAAAACAAGATCAATAG (insertion)

Allele2: AGAGAGAGAGACATGGGAAGGGGTAGGGTTCAGCTGTAAGAGGATAGAAAACAAGATCAATAG (insertion)

Ref: AGAGAGAGAGACATGGGAAGGGGTAGGGTTCAGCTG-AAGAGGATAGAAAACAAGATCAATAG

L5 *BnaFUL*-sgRNA1

Allele1: CATCTTCTCTTCCA (complicated variant)

Ref: CATCTTCTCTTCCAAAGGCAAACTCTTCGAATATTCCACCGACTCTTGGTAAT

L5 *BnaFUL*-sgRNA2

Allele1: AGAGAGAGAGACATGGGAAGGGGTAGGGTTCAGCTGAAAGAGGATAGAAAACAAGATCAATAG (insertion)

Allele2: AGAGAGAGAGACATGGGAAGGGGTAGGGTTCAGCTGTAAGAGGATAGAAAACAAGATCAATAG (insertion)

Ref: AGAGAGAGAGACATGGGAAGGGGTAGGGTTCAGCTG-AAGAGGATAGAAAACAAGATCAATAG

L6 *BnaFUL*-sgRNA1

Allele1: CATCTTCTCTTCCA (complicated variant)

Ref: CATCTTCTCTTCCAAAGGCAAACTCTTCGAATATTCCACCGACTCTTGGTAAT

L6 *BnaFUL*-sgRNA2

Allele1: AGAGAGAGAGACATGGGAAGGGGTAGGGTTCAGCTGAAAGAGGATAGAAAACAAGATCAATAG (insertion)

Allele2: AGAGAGAGAGACATGGGAAGGGGTAGGGTTCAGCTGTAAGAGGATAGAAAACAAGATCAATAG (insertion)

Ref: AGAGAGAGAGACATGGGAAGGGGTAGGGTTCAGCTG-AAGAGGATAGAAAACAAGATCAATAG

L7 *BnaFUL*-sgRNA1

Allele1: CATCTTCTCTTCCA (complicated variant)

Ref: CATCTTCTCTTCCAAAGGCAAACTCTTCGAATATTCCACCGACTCTTGGTAAT

L7 *BnaFUL*-sgRNA2

Allele1: AGAGAGAGAGACATGGGAAGGGGTAGGGTTCAGCTGAAAGAGGATAGAAAACAAGATCAATAG (insertion)

Allele2: AGAGAGAGAGACATGGGAAGGGGTAGGGTTCAGCTGTAAGAGGATAGAAAACAAGATCAATAG (insertion)

Ref: AGAGAGAGAGACATGGGAAGGGGTAGGGTTCAGCTG-AAGAGGATAGAAAACAAGATCAATAG

L8 *BnaFUL*-sgRNA1

Allele1: CATCTTCTCTTCCA (complicated variant)

Ref: CATCTTCTCTTCCAAAGGCAAACTCTTCGAATATTCCACCGACTCTTGGTAAT

L8 *BnaFUL*-sgRNA2

Allele1: AGAGAGAGAGACATGGGAAGGGGTAGGGTTCAGC (complicated variant)

Ref: AGAGAGAGAGACATGGGAAGGGGTAGGGTTCAGCTG-AAGAGGATAGAAAACAAGATCAATAG

L9 *BnaFUL*-sgRNA1

Allele1: CATCTTCTCTTCCAAAGGCAAACTCTTCGAATATTCCACCGACTCTTGGTAAT (WT)

Ref: CATCTTCTCTTCCAAAGGCAAACTCTTCGAATATTCCACCGACTCTTGGTAAT

L9 *BnaFUL*-sgRNA2

Allele1: AGAGAGAGAGACATGGGAAGGGGTAGGGTTCAGCTGAAGAGGATAGAAAACAAGATCAATAG (WT)

Ref: AGAGAGAGAGACATGGGAAGGGGTAGGGTTCAGCTGAAGAGGATAGAAAACAAGATCAATAG

L10 *BnaFUL*-sgRNA1

Allele1: CATCTTCTCTTCCAAAGGCAAACTCTTCGAATATTCCACCGACTCTTGGTAAT (WT)

Ref: CATCTTCTCTTCCAAAGGCAAACTCTTCGAATATTCCACCGACTCTTGGTAAT

L10 *BnaFUL*-sgRNA2

Allele1: AGAGAGAGAGACATGGGAAGGGGTAGGGTTCAGCTGAAAGAGGATAGAAAACAAGATCAATAG (insertion)

Ref: AGAGAGAGAGACATGGGAAGGGGTAGGGTTCAGCTG-AAGAGGATAGAAAACAAGATCAATAG

L11 *BnaFUL*-sgRNA1

Allele1: CATCTTCTCTTCCA (complicated variant)

Ref: CATCTTCTCTTCCAAAGGCAAACTCTTCGAATATTCCACCGACTCTTGGTAAT

L11 *BnaFUL*-sgRNA2

Allele1: AGAGAGAGAGACATGGGAAGGGGTAGGGTTCAGC (complicated variant)

Ref: AGAGAGAGAGACATGGGAAGGGGTAGGGTTCAGCTG-AAGAGGATAGAAAACAAGATCAATAG

L12 *BnaFUL*-sgRNA1

Allele1: CATCTTCTCTTCCA (complicated variant)

Ref: CATCTTCTCTTCCAAAGGCAAACTCTTCGAATATTCCACCGACTCTTGGTAAT

L12 *BnaFUL*-sgRNA2

Allele1: AGAGAGAGAGACATGGGAAGGGGTAGGGTTCAGCTGAAAGAGGATAGAAAACAAGATCAATAG (insertion)

Ref: AGAGAGAGAGACATGGGAAGGGGTAGGGTTCAGCTG-AAGAGGATAGAAAACAAGATCAATAG

L15 *BnaFUL*-sgRNA1

Allele1: CATCTTCTCTTCCA (complicated variant)

Ref: CATCTTCTCTTCCAAAGGCAAACTCTTCGAATATTCCACCGACTCTTGGTAAT

L15 *BnaFUL*-sgRNA2

Allele1: GACATGGGAAGGGGTAGGGT-----------GGATAGAAAACAAGA (deletion)

Allele2: GACATGGGAAGGGGTAGGGTTCAGCTG (43-bp del) AGAGAAGG (deletion)

Ref: GACATGGGAAGGGGTAGGGTTCAGCTGAAGAGGATAGAAAACAAGATCAATAGGCAAGTTACTTTCTCTA

L16 *BnaFUL*-sgRNA1

Allele1: CATCTTCTCTTCCA (complicated variant)

Ref: CATCTTCTCTTCCAAAGGCAAACTCTTCGAATATTCCACCGACTCTTGGTAAT

L16 *BnaFUL*-sgRNA2

Allele1: AGAGAGAGAGACATGGGAAGGGGTAGGGTTCAGC (complicated variant)

Ref: AGAGAGAGAGACATGGGAAGGGGTAGGGTTCAGCTG-AAGAGGATAGAAAACAAGATCAATAG

L17 *BnaFUL*-sgRNA1

Allele1: CATCTTCTCTTCCAAAGGCAAACTCTTCGAATATTCCACCGACTCTTGGTAAT (WT)

Ref: CATCTTCTCTTCCAAAGGCAAACTCTTCGAATATTCCACCGACTCTTGGTAAT

L17 *BnaFUL*-sgRNA2

Allele1: AGAGAGAGAGACATGGGAAGGGGTAGGGTTCAGCTGAAGAGGATAGAAAACAAGATCAATAG (WT)

Ref: AGAGAGAGAGACATGGGAAGGGGTAGGGTTCAGCTGAAGAGGATAGAAAACAAGATCAATAG

L18 *BnaFUL*-sgRNA1

Allele1: CATCTTCTCTTCCA (complicated variant)

Ref: CATCTTCTCTTCCAAAGGCAAACTCTTCGAATATTCCACCGACTCTTGGTAAT

L18 *BnaFUL*-sgRNA2

Allele1: AGAGAGAGAGACATGGGAAGGGGTAGGGTTCAGCTGAAAGAGGATAGAAAACAAGATCAATAG (insertion)

Ref: AGAGAGAGAGACATGGGAAGGGGTAGGGTTCAGCTG-AAGAGGATAGAAAACAAGATCAATAG

L19 *BnaFUL*-sgRNA1

Allele1: CATCTTCTCTTCCAAAGGCAAACTCTTCGAATATTCCACCGACTCTTGGTAAT (WT)

Ref: CATCTTCTCTTCCAAAGGCAAACTCTTCGAATATTCCACCGACTCTTGGTAAT

L19 *BnaFUL*-sgRNA2

Allele1: AGAGAGAGAGACATGGGAAGGGGTAGGGTTCAGCTGAAGAGGATAGAAAACAAGATCAATAG (WT)

Ref: AGAGAGAGAGACATGGGAAGGGGTAGGGTTCAGCTGAAGAGGATAGAAAACAAGATCAATAG

L21 *BnaFUL*-sgRNA1

Allele1: CATCTTCTCTTCCAAAGGCAAACTCTTCGAATATTCCACCGACTCTTGGTAAT (WT)

Ref: CATCTTCTCTTCCAAAGGCAAACTCTTCGAATATTCCACCGACTCTTGGTAAT

L21 *BnaFUL*-sgRNA2

Allele1: AGAGAGAGAGACATGGGAAGGGGTAGGGTTCAGCTGAAGAGGATAGAAAACAAGATCAATAG (WT)

Ref: AGAGAGAGAGACATGGGAAGGGGTAGGGTTCAGCTGAAGAGGATAGAAAACAAGATCAATAG

L23 *BnaFUL*-sgRNA1

Allele1: CATCTTCTCTTCCA (complicated variant)

Ref: CATCTTCTCTTCCAAAGGCAAACTCTTCGAATATTCCACCGACTCTTGGTAAT

L23 *BnaFUL*-sgRNA2

Allele1: ATGGGAAGGGGTAGGGTTCA--------AGAGGATAGAAAACAAGA (deletion)

Allele2: ATGGGAAGGGGTAGGGTTCAGCTGAAAAGAGGATAGAAAACAAGATCAATAGGCAA (insertion)

Ref: ATGGGAAGGGGTAGGGTTCAGCTG--AAGAGGATAGAAAACAAGATCAATAGGCAAGTTACTTTCTCTAAGA

L24 *BnaFUL*-sgRNA1

Allele1:	CATCTTCTCTTCCA	(complicated variant)
Ref:	CATCTTCTCTTCCAAAGGCAAACTCTTCGAATATTCCACCGACTCTTGGTAAT	

L24 *BnaFUL*-sgRNA2

Allele1:	AGAGAGAGAGACATGGGAAGGGGTAGGGTTCAGCTGAAAGAGGATAGAAAACAAGATCAATAG	(insertion)
Allele2:	AGAGAGAGAGACATGGGAAGGGGTAGGGTTCAGCTGTAAGAGGATAGAAAACAAGATCAATAG	(insertion)
Ref:	AGAGAGAGAGACATGGGAAGGGGTAGGGTTCAGCTG-AAGAGGATAGAAAACAAGATCAATAG	
